# Bifurcated synthesis of methylene-lactone- and methylene-lactam-fused spirolactams via electrophilic amide allylation of γ-phenylthio-functionalized γ-lactams

**DOI:** 10.3762/bjoc.16.227

**Published:** 2020-11-13

**Authors:** Tetsuya Sengoku, Koki Makino, Ayumi Iijima, Toshiyasu Inuzuka, Hidemi Yoda

**Affiliations:** 1 Department of Applied Chemistry, Faculty of Engineering, Shizuoka University, 3-5-1 Johoku, Naka-ku, Hamamatsu 432-8561, Japan; 2 Division of Instrumental Analysis, Life Science Research Center, Gifu University, 1-1 Yanagido, Gifu 501-1193, Japan

**Keywords:** bifurcated synthesis, electrophilic amide allylation, α-methylene-γ-butyrolactam, α-methylene-γ-butyrolactone, spirolactams

## Abstract

New synthetic methods for spirolactams bearing an α-methylene-γ-butyrolactone or its analogous methylene-lactam have been developed. The allylation of γ-phenylthio-functionalized γ-lactams with 2-(acetoxy)methyl acrylamides was accomplished by using 2.5 equivalents of NaH to give the corresponding adducts in excellent yields. The remaining phenylthio group was substituted with a hydroxy group by treatment with CuBr, and the resulting γ-hydroxyamides were cyclized under acidic conditions to afford the corresponding methylene-lactam-fused spirolactams in high yields. On the other hand, methylene-lactone-fused spirolactams could be delivered from the allyl adducts in high yields through a sequential *N*-Boc protection/desulfinative lactonization.

## Introduction

**Scheme 1:**
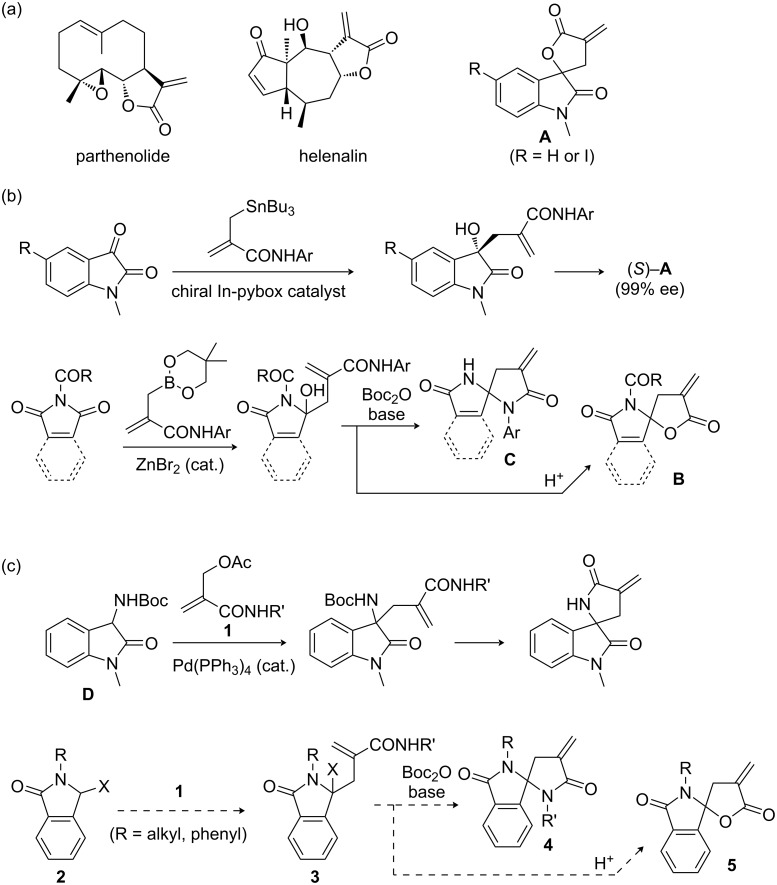
Examples of (a) bioactive compounds bearing an α-methylene-γ-butyrolactone structure, (b) syntheses of spirocyclic compounds through nucleophilic amide allylation, and (c) syntheses of spirocyclic compounds through electrophilic amide allylation.

α-Methylene-γ-butyrolactone is a pharmaceutically important motif which is found in a wide variety of bioactive molecules including natural products (Scheme 1a) [1–3]. For example, sesquiterpene lactones represented by parthenolide and helenalin have attracted interest because of their useful biological activities, and many synthetic efforts have been made so far [4–6]. On the other hand, syntheses and biological evaluation of nonnatural methylene-lactones also have been reported [4,6–8]. For instance, Heindel and his co-worker synthesized methylene-lactone derivatives spiro-fused to an oxindole (**A**), one of which exhibited a potent cytotoxic activity [9]. Our research group succeeded the enantiopure synthesis of these compounds, in which enantioselective allylation of an isatin derivative with an amido-functionalized allylstannane was employed as a key reaction (Scheme 1b) [10–11]. More recently, an amide-functionalized allyl boronate was developed as an alternative reagent for the allylstannane [12–16], which led to the syntheses of not only cytotoxic methylene-lactones spiro-fused to a lactam ring (**B**) but also their analogous methylene-lactams (**C**) through zinc-catalyzed addition to *N*-carbonyl imides [13–14,16].

Meanwhile, we also developed an umpolung electrophilic allylation of 3-heterosubstituted oxindole **D** for the synthesis of lactam analog of **A** (Scheme 1c) [17]. The oxindole **D** readily reacted with 2-(acetoxy)methyl acrylamides **1** in the presence of a catalytic amount of Pd(PPh_3_)_4_ to give the corresponding adducts which could be delivered into an methylene-lactam-fused oxindole. On the basis of this umpolung strategy, spirolactams **4** and **5**, which are *N*-alkyl or *N*-phenyl-substituted analogs of **B** and **C** and unable to be obtained under the conditions depicted in Scheme 1b, seems to be accessible from the common precursor **3** prepared through the reaction between **1** and 3-heterosubstituted isoindolinones **2** followed by cyclization. Considering that **2** is estimated to be less reactive compared with the oxindole derivative **D** which show excellent nucleophilicity arising from the oxindole–hydroxyindole tautomerization [18–19], this synthetic approach is undoubtedly attractive because it could complement our previous one using nucleophilic amido-functionalized allyl boronates in terms of the structural diversity as well as understanding of the structure–cytotoxicity relationship [14]. Therefore, we were motivated to develop a new synthetic methodology for *N*-alkyl and *N*-phenyl derivatives **4** and **5** starting from **2** with **1**.

## Results and Discussion

Our studies began with an exploration of a promising substrate for the desired electrophilic allylation. We initially chose *N*-phenyl-3-(acetoxy)isoindolinone (**2a**) as a reactant. However, **2a** showed no reactivity under the reaction conditions determined for the palladium-catalyzed allylation in our previous work and was recovered in 98% yield [17] (Table 1, entry 1). The use of strong bases such as potassium *tert*-butoxide or sodium hydride resulted in no formation of the desired product **3a** because **2a** was decomposed under the harsh reaction conditions (Table 1, entries 2 and 3). Thus we opted to employ another substrate **2b** bearing a phenylthio group which polarizes the α-C–H bond to lead the corresponding stabilized anion [20] and is readily transformed into a hydroxy group by treatment with copper bromide according to our previous work [21]. Allylation of **2b** with **1a** at room temperature proceeded in the presence of a catalytic amount of Pd(PPh_3_)_4_ by using potassium *tert*-butoxide or sodium hydride, affording the corresponding adduct in 50% and 81% yields, respectively (Table 1, entries 6 and 7). Surprisingly, the desired allylation underwent even in the absence of palladium catalyst, probably due to high nucleophilicity of the deprotonated intermediate, to give **3b** in 87% yield (Table 1, entry 8). Optimization studies were conducted by screening solvents, reagent amount, and reaction temperature, showing that **3b** was produced in the highest yield of 97% when the reaction was carried out with 2.5 equivalents of sodium hydride in THF at −10 °C (Table 1, entries 9–13) [22].

**Table 1:** Screening of reaction conditions for electrophilic amide allylation.

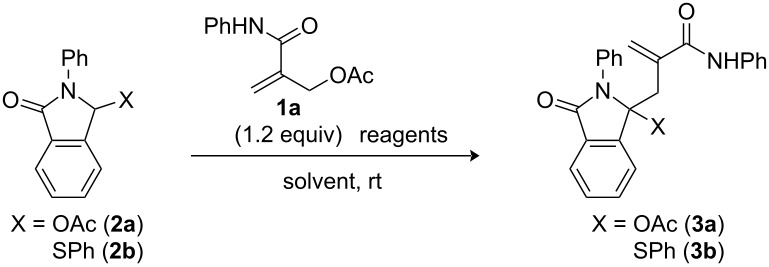					

entry	**2**	reagents (equiv)	solvent	time (h)	**3** (%)

1	**2a**	Et_3_N (2.5) Pd(PPh_3_)_4_ (0.1)	THF	24	**3a** (0)
2	**2a**	*t*-BuOK (2.5) Pd(PPh_3_)_4_ (0.1)	THF	24	**3a** (0)
3	**2a**	NaH (2.5) Pd(PPh_3_)_4_ (0.1)	THF	24	**3a** (0)
4	**2b**	Pyridine (2.5) Pd(PPh_3_)_4_ (0.1)	THF	24	**3b** (0)
5	**2b**	Et_3_N (2.5) Pd(PPh_3_)_4_ (0.1)	THF	24	**3b** (0)
6	**2b**	*t*-BuOK (2.5) Pd(PPh_3_)_4_ (0.1)	THF	24	**3b** (50)
7	**2b**	NaH (2.5) Pd(PPh_3_)_4_ (0.1)	THF	1	**3b** (81)
8	**2b**	NaH (2.5)	THF	1	**3b** (87)
9	**2b**	NaH (1.0)	THF	2	**3b** (62)
10	**2b**	NaH (2.5)	toluene	1	**3b** (74)
11	**2b**	NaH (2.5)	DMF	1	**3b** (18)
12^a^	**2b**	NaH (2.5)	THF	1	**3b** (97)
13^b^	**2b**	NaH (2.5)	THF	1	**3b** (87)

^a^The reaction was performed at −10 °C. ^b^The reaction was performed at −20 °C.

**Figure 1:**
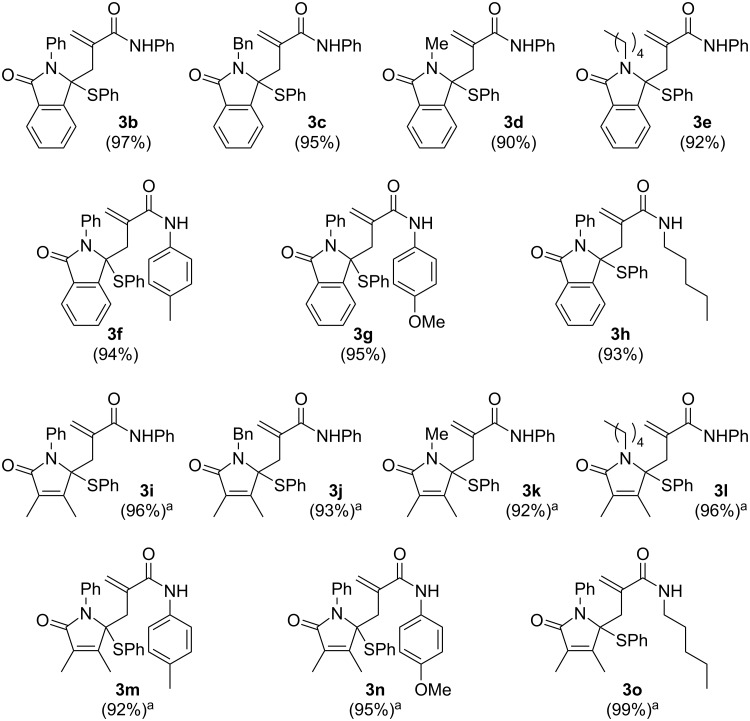
Syntheses of **3b**–**o** via electrophilic amide allylation of γ-phenylthio lactams. Reactions were carried out with **1** (1.2 equiv), **2** (1.0 equiv), and NaH (2.5 equiv) in THF (0.2 M for **2**) at −10 °C for 1 h. ^a^Reactions were performed at −20 °C.

With the optimal reaction conditions for electrophilic allylation of **2b** with **1a** in hand, we turned to demonstrate the versatility of this method. In our previous work relating to nucleophilic allylation of imide derivatives, we succeeded the syntheses of *N*-carbonyl-functionalized γ-hydroxy amides bearing an amide side chain [14]. Therefore, we here examined reactions using *N*-alkyl derivatives in order to gain the structural diversity. Reactions of 3-(phenylthio)isoindolinone derivatives bearing an *N*-benzyl (**2c**), *N*-methyl (**2d**), and *N*-(*n*-pentyl) group (**2d**) with **1a** readily proceeded under the optimized reaction conditions for **3b** to afford the corresponding products **3c**–**e** in 90–95% yields (Figure 1). As for the substituents on the methacrylamide side chain, not only aryl groups (*p*-tolyl and *p*-anisyl groups) but also an *n*-pentyl group were tolerated, providing **3f**–**h** in excellent yields. On the other hand, a reaction of a nonaromatic lactam derivative prepared from *N*-phenyl-2,3-dimethylmaleimide was accompanied by the formation of structure-unidentified side products, resulting in relatively low product yield (**3i**, 78% yield). This problem was overcome by carrying out the reaction at −20 °C. Under these conditions, **3i**–**o** were predominantly formed in 92–99% yields. Thus, we could obtain a wide variety of 3-phenylthio lactams bearing an amide functionality by electrophilic allylation with **1**.

**Scheme 2:**
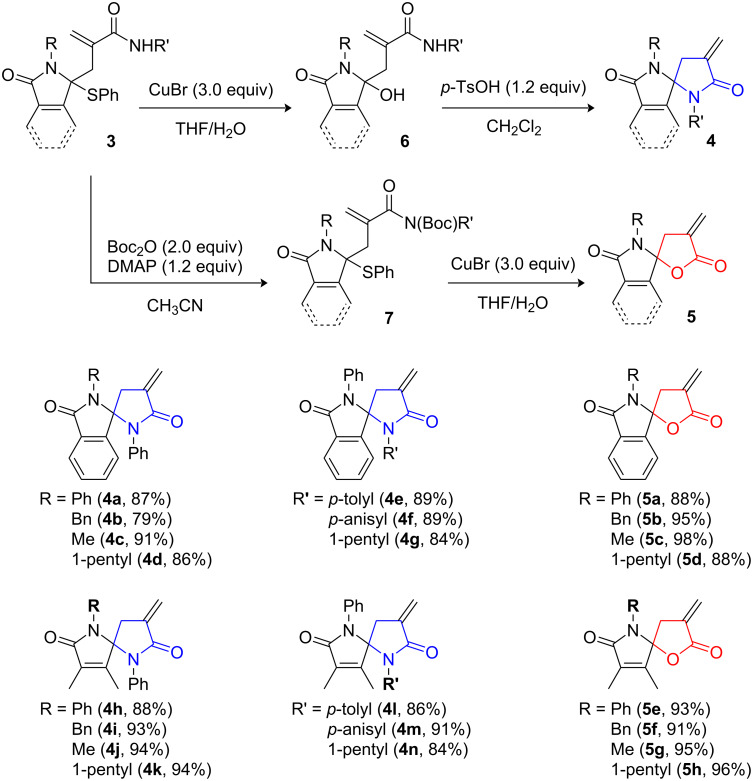
Syntheses of *N*-phenyl and *N*-alkyl-substituted spirolactams (two-step yields from **3**).

Having completed the installation of an acrylamide side chain into 3-phenylthio lactams, we subsequently proceeded to the construction of the spiro skeleton of **4** and **5**. For this purpose, we initially attempted to transform **3b** to **5a** through the reaction sequence consisting of copper-mediated hydroxylation and acid-mediated lactonization based on our previous reports [10–11,14,21]. Substitution of the phenylthio group of **3b** with a hydroxy group was readily achieved by treatment with CuBr in aqueous media (THF/H_2_O) to afford the corresponding hydroxylactam in 90% yield (Scheme 2). However, the subsequent spirolactonization with *p*-toluenesulfonic acid (*p*-TsOH) was unsuccessful, leading to predominant production of bislactam derivative **4a** (97%) [23–25]. Although acidic spirolactamization was contrary to our initial expectations, a new type of *N*-phenyl spirobislactam, which could not be obtained by our previous method, became accessible through this reaction. Therefore, we decided to evaluate its applicability to other substrates. Reactions of isoindolinone derivatives **3c**–**h** under the comparable conditions used for **3b** were successful, furnishing the corresponding *N*-alkyl and *N*-phenyl bislactams **4b**–**g** in 79–91% two-step yields. Similarly, non-aromatic substrates **3i**–**o** were also converted into **4h**–**n** in 84–94% yields, indicating widespread applicability of this sequential transformation.

Since the cyclization under acidic conditions led to the predominant formation of the methylene-lactam-fused spirolactam, we set another route toward **5** via cyclization of *N*-Boc-functionalized γ-hydroxymethacrylamide [26]. We initially treated the hydroxylactam prepared through CuBr-mediated hydroxylation of **3b** with di-*tert*-butyl dicarbonate (Boc_2_O) in the presence of *N*,*N*-dimethyl-4-aminopyridine (DMAP), but a mixture of structurally unidentified products was formed. Meanwhile, when the reaction was carried out with 2.0 equivalents of *n*-butyllithium and 1.1 equivalents of Boc_2_O in THF at −78 °C, **5a** was obtained as a mixture including unreacted starting material (<11% yield), indicating that installation of an *N*-Boc group on the terminus amide should trigger the desired lactonization. Therefore, we next examined the transposed reaction sequence, *N*-Boc protection followed by hydroxylation (Scheme 2). *N*-Boc amides were readily obtained by treatment of **3b**–**o** with Boc_2_O and DMAP, which were successively subjected to hydroxylation in the presence of CuBr. Expectedly, the desired lactonization occurred spontaneously under the reaction conditions and led to methylene-lactone-fused spirolactams **5a**–**h** in excellent yields without exception (88–98% two-step yields). Thus, we established the bifurcated synthetic routes toward lactams spiro-fused to α-methylene-γ-butyrolactone or α-methylene-γ-butyrolactam by using 3-phenylthiolactams bearing an acrylamide side chain as a common intermediate.

**Figure 2:**
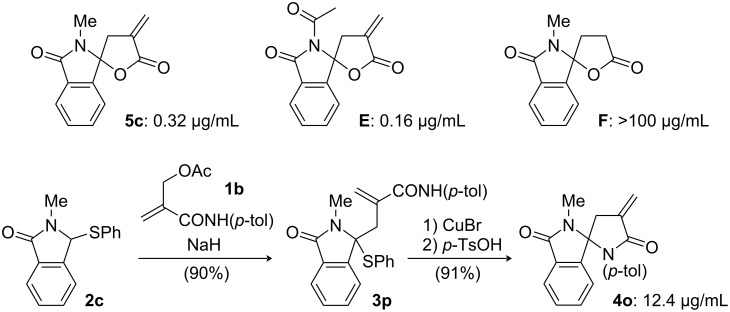
Cytotoxicity of spirolactams on P388 cells (IC_50_ values).

Finally, we subjected methylene-lactone-fused spirolactams to the assay of cytotoxicity on P388 cells (Figure 2). *N*-Methyl-substituted spirolactam **5c** exhibited potent cytotoxicity (IC_50_ 0.32 μg/mL) which was comparable to that of the *N*-acetyl analog **E** (0.16 μg/mL) [14]. It should be noted that the IC_50_ values of our recently reported non-conjugated lactone **F** [27] was >100 μg/mL. In addition to **5c**, we tested the cytotoxicity of methylene-lactam-based compound **4o**, which exhibited weak activity (IC_50_ 12.4 μg/mL) against the P388 cell line. These results suggest that cytotoxic activity of this type of spirolactams against P388 cells is simply related to the methylene lactone structure [28].

## Conclusion

In conclusion, we have achieved a new bifurcated syntheses of *N*-alkyl and *N*-phenyl-substituted spirolactams bearing a methylene-lactone or methylene-lactam structure. The key intermediates were synthesized by electrophilic allylation of γ-phenylthio-functionalized γ-lactam derivatives with 2-(acetoxy)methyl acrylamides in the absence of any metal catalyst and delivered into each type of spirolactams through desulfinative hydroxylation/lactamization or *N*-Boc protection/desulfinative lactonization. The increase of diversity of structure accessible led to further understanding of the structure–cytotoxicity relationship of spirolactams on P388 cells.

## Supporting Information

File 1 Experimental procedures and characterization data.

File 2 Copies of ^1^H and ^13^C NMR spectra of all new compounds.
