# Supplementary material for: Bifurcated synthesis of methylene-lactone- and methylene-lactam-fused spirolactams via electrophilic amide allylation of γ-phenylthio-functionalized γ-lactams
Source: Beilstein J Org Chem. 2020 Nov 13;16:2769–75. doi: 10.3762/bjoc.16.227 (PMC7670114; doi:10.3762/bjoc.16.227)
Supplement: File 1 — Experimental procedures and characterization data. [file Beilstein_J_Org_Chem-16-2769-s001.pdf]

BEILSTEIN JOURNAL OF ORGANIC CHEMISTRY

Supporting Information

for

Bifurcated synthesis of methylene-lactone- and methylene-lactam-fused spirolactams via electrophilic amide allylation of γ-phenylthio-functionalized γ-lactams

Tetsuya Sengoku, Koki Makino, Ayumi Iijima, Toshiyasu Inuzuka and Hidemi Yoda

Beilstein J. Org. Chem. **2020,** *16,* 2769–2775. doi:10.3762/bjoc.16.227

Experimental procedures and characterization data

License and Terms: This is a supporting information file under the terms of the Creative Commons Attribution License (https://creativecommons.org/licenses/by/4.0). Please note that the reuse, redistribution and reproduction in particular requires that the authors and source are credited. The license is subject to the *Beilstein Journal of Organic Chemistry* terms and conditions: (https://www.beilstein-journals.org/bjoc)

The following data are included in this material:

General methods S2

Experimental procedures and characterization data S2–S31

General methods. All solvents and reagents were of reagent grade quality, and used without further purification unless otherwise stated. Chloroform, acetonitrile, ethanol, toluene, dichloromethane and 1,2-dichloroethane were dried over MS 4 Å or MS 3 Å prior to use, respectively. Tetrahydrofuran was dried over Na wire under a nitrogen atmosphere. The ^{1}H and ^{13}C nuclear magnetic resonance (NMR) spectra operating at the frequencies of 300 and 75 MHz, respectively, on a JEOL JNM-AL300 spectrometer were recorded in chloroform–*d* ($CDCl_3$) unless otherwise noted. Chemical shifts are reported in parts per million (ppm) relative to TMS and the solvent used as internal standards, and the coupling constants are reported in hertz (Hz). Reactions were monitored by thin layer chromatography (TLC) using Merck TLC silica gel $60F_{254}$, visualized by irradiation with UV light and/or by treatment with phosphomolybdic acid or *p*-anisaldehyde stain followed by heating. Column chromatography was performed using silica gel 60N (spherical neutral) from Kanto Chemical Co. and eluting with the indicated solvent system. Fourier transform infrared (FTIR) spectra were recorded on a JASCO FT/IR-4100 spectrometer. Elemental analyses were performed by JSL Model JM 10 instruments. Allylating reagent **1a** was prepared according to the literature procedure [1].

Experimental procedures and characterization data.

Synthesis and characterization of 2a. To a solution of *N*-phenylphthalimide (639 mg, 2.86 mmol) in methanol (13 mL) was added sodium borohydride (130 mg, 3.43 mmmol) at 0 °C. After stirring the solution at room temperature for 2 hours, the reaction was quenched by addition of water (10 mL), and the resulting solution was concentrated under reduced pressure. The resulting mixture was extracted with ethyl acetate (2 × 20 mL). The combined organic extracts were washed with brine (10 mL), dried over Na_2SO_4, filtered, and concentrated in vacuo to provide a crude material (639 mg) which was used in the next step without further purification. R_f = 0.30 (silica gel, hexane/EtOAc = 2/1).

To a solution of the crude material (639 mg) in dichloromethane (5.7 mL) were added triethylamine

(0.59 mL, 3.4 mmol) and acetic anhydride (0.32 mL, 3.4 mmol) at room temperature. After stirring the solution at the same temperature for 2 days, the reaction mixture was concentrated in vacuo to provide a crude material. This material was purified by column chromatography (silica gel, hexane/EtOAc = 4/1 to 2/1) followed by recrystallization (hexane/EtOAc) to give **2a** [2] (301 mg, 1.13 mmol, 40% for 2 steps) as a white solid. R_f = 0.33 (silica gel, hexane/EtOAc = 4/1); m.p. 97–100 °C; IR (KBr) 1719 (C=O) cm^{-1}; ^{1}H NMR (300 MHz, $CDCl_3$) δ 7.92 (m, 1H), 7.65–7.58 (m, 5H), 7.59 (s, 1H), 7.45 (t, *J* = 7.5 Hz, 2H), 7.27 (m, 1H), 2.06 (s, 3H); ^{13}C NMR (75 MHz, $CDCl_3$) δ 170.9, 166.7, 140.3, 136.2, 133.0, 131.9, 130.6, 129.2, 126.0, 124.1, 123.8, 122.7, 81.4, 21.0. Anal. Calcd for $C_{16}H_{13}NO_3$: C, 71.90; H, 4.90; N, 5.24. Found: C, 71.56; H, 5.05; N, 5.07.

Synthesis and characterization of 2b. To a solution of *N*-phenylphthalimide (617 mg, 2.76 mmol) in methanol (14 mL) was added sodium borohydride (125 mg, 3.31 mmmol) at 0 °C. After stirring the solution at room temperature for 2 hours, the reaction was quenched by addition of water (10 mL), and the resulting solution was concentrated under reduced pressure. The resulting mixture was extracted with ethyl acetate (2 × 20 mL). The combined organic extracts were washed with brine (10 mL), dried over Na_2SO_4, filtered, and concentrated in vacuo to provide a crude material (623 mg) which was used in the next step without further purification. R_f = 0.30 (silica gel, hexane/EtOAc = 2/1).

To a solution of the crude material (623 mg) in dichloromethane (14 mL) were added thiophenol (365 mL, 3.31 mmol) and boron trifluoride-diethyl ether complex (392 mg, 2.76 mmol) at room temperature. After stirring the solution at the same temperature for 3 hours, the reaction mixture was quenched with sat. $NaHCO_3$ aq (20 mL). The resulting mixture was extracted with dichloromethane (2 × 20 mL). The combined organic extracts were washed with brine (10 mL), dried over Na_2SO_4, filtered, and concentrated in vacuo to provide a crude material. This material was purified by column chromatography (silica gel, hexane/EtOAc = 5/1 to 2/1) followed by recrystallization (hexane/EtOAc) to give **2b** [3] (470 mg, 1.48 mmol, 54% for 2 steps) as a white solid. R_f = 0.30 (silica gel, hexane/EtOAc = 3/1); m.p. 130–131 °C; IR (KBr) 1686 (C=O) cm^{-1}; ^{1}H

NMR (300 MHz, $CDCl_3$) δ 7.73–7.57 (m, 5H), 7.47 (t, *J* = 7.5 Hz, 2H), 7.39 (t, *J* = 7.5 Hz, 1H), 7.27 (t, *J* = 7.2 Hz, 1H), 7.14 (t, *J* = 7.2 Hz, 1H), 7.00 (t, *J* = 8.1 Hz, 2H), 6.88 (d, *J* = 8.1 Hz, 2H), 6.32 (s, 1H). ^{13}C NMR (75 MHz, $CDCl_3$) δ 166.2, 142.3, 136.6, 135.9, 132.1, 131.7, 129.2, 128.9, 128.4, 127.4, 125.4, 123.7, 123.5, 123.3, 66.8. Anal. Calcd for $C_{20}H_{15}NOS$: C, 75.68; H, 4.76; N, 4.41. Found: C, 75.37; H, 4.94; N, 4.39.

Preparation and characterization of 2c. According to the synthetic procedure of **2b**, **2c** was prepared from *N*-benzylphthalimide (1.21 g, 5.10 mmol). The crude product was purified by column chromatography (silica gel, hexane/EtOAc = 5/1 to 2/1) to give **2c** (1.54 g, 4.65 mmol, 91% for 2 steps) as a white solid. R_f = 0.30 (silica gel, hexane/EtOAc = 3/1); m.p. 89–90 °C; IR (KBr) 1686 (C=O) cm^{-1}; ^1H NMR (300 MHz, $CDCl_3$) δ 7.62–7.50 (m, 3H), 7.37–7.25 (m, 6H), 7.20–7.13 (m, 1H), 7.10–7.04 (m, 4H), 5.50 (s, 1H), 5.45 (d, *J* = 14.7 Hz, 1H), 4.54 (d, *J* = 14.7 Hz, 1H); ^{13}C NMR (75 MHz, $CDCl_3$) δ 167.2, 142.8, 136.7, 135.2, 131.6, 131.5, 129.0, 128.7, 128.6, 128.4, 127.9, 127.6, 123.7, 123.2, 65.7, 43.1. Anal. Calcd for $C_{21}H_{17}NOS$: C, 76.10; H, 5.17; N, 4.23. Found: C, 76.06; H, 4.94; N, 4.24.

Preparation and characterization of 2d. According to the synthetic procedure of **2b**, **2d** was prepared from *N*-methylphthalimide (2.52 g, 15.6 mmol). The crude product was purified by column chromatography (silica gel, hexane/EtOAc = 3/1) to give **2d** (2.94 g, 11.5 mmol, 73% for 2 steps) as a white solid. R_f = 0.40 (silica gel, hexane/EtOAc = 1/1); m.p. 87–88 °C; IR (KBr) 1692 (C=O) cm^{-1}; ^1H NMR (300 MHz, $CDCl_3$) δ 7.63 (d, *J* = 7.5 Hz, 1H), 7.53 (q, *J* = 6.9 Hz, 2H), 7.33 (t, *J* = 7.5 Hz, 1H), 7.14 (m, 1H), 7.09–7.05 (m, 4H), 5.61 (s, 1H), 3.25 (s, 3H); ^{13}C NMR (75 MHz, $CDCl_3$) δ 167.4, 142.6, 135.2, 131.8, 131.4, 129.0, 128.6, 128.0, 123.5, 122.9, 68.5, 27.2. Anal. Calcd for $C_{15}H_{13}NOS$: C, 70.56; H, 5.13; N, 5.49. Found: C, 70.73; H, 5.12; N, 5.45.

Preparation and characterization of 2e. According to the synthetic procedure of **2b**, **2e** was prepared from *N*-pentylphthalimide (0.892 g, 4.12 mmol). The crude product was purified by column chromatography (silica gel, hexane/EtOAc = 5/1 to 2/1) to give **2e** (1.18 g, 3.79 mmol, 92% for 2 steps) as a colorless oil. R_f = 0.40 (silica gel, hexane/EtOAc = 3/1); IR (KBr) 1690 (C=O)

cm^{-1}; ^{1}H NMR (300 MHz, $CDCl_3$) δ 7.64 (d, *J* = 7.5 Hz, 1H), 7.54 (q, *J* = 7.5 Hz, 2H), 7.33 (t, *J* = 7.5 Hz, 1H), 7.15 (m, 1H), 7.09–7.03 (m, 4H), 5.73 (s, 1H), 4.05 (m, 1H), 3.53 (m, 1H), 1.71–1.57 (m, 2H), 1.39–1.28 (m, 4H), 0.89 (t, *J* = 6.6 Hz, 3H); ^{13}C NMR (75 MHz, $CDCl_3$) δ 167.1, 142.6, 135.1, 131.8, 131.3, 128.8, 128.4, 128.0, 123.5, 122.9, 66.2, 39.3, 28.9, 27.7, 22.2, 13.9. Anal. Calcd for $C_{19}H_{21}NOS$: C, 73.27; H, 6.80; N, 4.50. Found: C, 72.98; H, 6.46; N, 4.49.

Synthesis and characterization of 2f. To a solution of 2,3-dimethyl-*N*-phenylmaleimide (757 mg, 3.76 mmol) in methanol (19 mL) was added sodium borohydride (213 mg, 5.64 mmmol) at 0 °C. After stirring the solution at room temperature for 3 hours, the reaction was quenched by addition of water (10 mL), and the resulting solution was concentrated under reduced pressure. The resulting mixture was extracted with chloroform (2 × 20 mL). The combined organic extracts were washed with brine (10 mL), dried over Na_2SO_4, filtered, and concentrated in vacuo to provide a crude material (651 mg) which was used in the next step without further purification. R_f = 0.31 (silica gel, hexane/EtOAc = 2/1).

To a solution of the crude material (651 mg) in dichloromethane (14 mL) were added thiophenol (387 mg, 3.52 mmol) and boron trifluoride-diethyl ether complex (454 mg, 3.20 mmol) at room temperature. After stirring the solution at the same temperature for 18 hours, the reaction mixture was quenched with sat. $NaHCO_3$ aq (20 mL). The resulting mixture was extracted with dichloromethane (2 × 20 mL). The combined organic extracts were washed with brine (10 mL), dried over Na_2SO_4, filtered, and concentrated in vacuo to provide a crude material. This material was purified by column chromatography (silica gel, hexane/EtOAc = 8/1 to 4/1 to 2/1) followed by recrystallization (hexane/EtOAc) to give **2f** (545 mg, 1.84 mmol, 49% for 2 steps) as a white solid. R_f = 0.21 (silica gel, hexane/EtOAc = 3/1); m.p. 127–128 °C; IR (KBr) 1678 (C=O) cm^{-1}; ^{1}H NMR (300 MHz, $CDCl_3$) δ 7.61 (d, *J* = 7.8 Hz, 2H), 7.42 (t, *J* = 7.5 Hz, 2H), 7.28 (m, 1H), 7.19 (t, *J* = 7.5 Hz, 3H), 7.08 (d, *J* = 8.1 Hz, 2H), 5.51 (s, 1H), 2.12 (s, 3H), 1.61 (s, 3H); ^{13}C NMR (75 MHz, $CDCl_3$) δ 169.7, 146.5, 136.9, 135.5, 130.5, 129.3, 128.8, 128.5, 127.8, 124.4, 121.4, 69.4, 12.6, 8.3. Anal. Calcd for $C_{18}H_{17}NOS$: C, 73.19; H, 5.80; N, 4.74. Found: C, 72.85; H, 5.63; N, 4.64.

Preparation and characterization of 2g. According to the synthetic procedure of **2f**, **2g** was prepared from 2,3-dimethyl-*N*-benzylmaleimide (3.14 g, 14.6 mmol). The crude product was purified by column chromatography (silica gel, hexane/EtOAc = 8/1 to 4/1) to give **2g** (4.28 g, 13.8 mmol, 95% for 2 steps) as a colorless oil. R_f = 0.21 (silica gel, hexane/EtOAc = 3/1); IR (KBr) 1685 (C=O) cm^{-1}; ^1H NMR (300 MHz, $CDCl_3$) δ 7.33–7.23 (m, 10H), 5.26 (d, *J* = 14.7 Hz, 1H), 4.74 (s, 1H), 4.14 (d, *J* = 14.7 Hz, 1H), 1.95 (s, 3H), 1.59 (s, 3H); ^{13}C NMR (75 MHz, $CDCl_3$) δ 170.8, 146.6, 137.3, 134.5, 130.0, 128.9, 128.8, 128.7, 128.3, 127.4, 69.2, 43.2, 12.5, 8.4. Anal. Calcd for $C_{19}H_{19}NOS$: C, 73.75; H, 6.19; N, 4.53. Found: C, 73.39; H, 6.49; N, 4.60.

Preparation and characterization of 2h. According to the synthetic procedure of **2f**, **2h** was prepared from 2,3-dimethyl-*N*-methylmaleimide (644 mg, 4.77 mmol). The crude product was purified by column chromatography (silica gel, hexane/EtOAc = 4/1 to 1/1) to give **2h** (1.08 g, 4.64 mmol, 97% for 2 steps) as a white solid. R_f = 0.32 (silica gel, hexane/EtOAc = 1/1); IR (KBr) 1683 (C=O) cm^{-1}; ^1H NMR (300 MHz, $CDCl_3$) δ 7.30–7.20 (m, 5H), 4.84 (s, 1H), 3.11 (s, 3H), 1.99 (s, 3H), 1.56 (s, 3H); ^{13}C NMR (75 MHz, $CDCl_3$) δ 170.9, 145.8, 134.5, 130.2, 128.8, 128.7, 128.6, 71.9, 26.9, 12.3, 8.3. Anal. Calcd for $C_{13}H_{15}NOS$: C, 66.92; H, 6.48; N, 6.00. Found: C, 66.54; H, 6.59; N, 5.72.

Preparation and characterization of 2i. According to the synthetic procedure of **2f**, **2i** was prepared from 2,3-dimethyl-*N*-pentylmaleimide (3.42 g, 15.9 mmol). The crude product was purified by column chromatography (silica gel, hexane/EtOAc = 2/1 to 1/1) to give **2i** (3.95 g, 13.6 mmol, 86% for 2 steps) as a yellow oil. R_f = 0.21 (silica gel, hexane/EtOAc = 3/1); m.p. 57–58 °C; IR (KBr) 1686 (C=O) cm^{-1}; ^1H NMR (300 MHz, $CDCl_3$) δ 7.28–7.19 (m, 5H), 4.94 (s, 1H), 3.87 (m, 1H), 3.45 (m, 1H), 1.99 (s, 3H), 1.56 (s, 3H), 1.55–1.46 (m, 2H), 1.37–1.25 (m, 4H), 0.89 (t, *J* = 6.6 Hz, 3H); ^{13}C NMR (75 MHz, $CDCl_3$) δ 170.8, 145.7, 134.5, 130.3, 128.93, 128.85, 128.7, 69.9, 39.5, 29.0, 28.2, 22.3, 13.9, 12.4, 8.3. Anal. Calcd for $C_{17}H_{23}NOS$: C, 70.55; H, 8.01; N, 4.84. Found: C, 70.38; H, 7.63; N, 4.86.

General procedure for the synthesis of 3b-o. All the experiments for the synthesis of **3b-o** were

carried out as described in the following typical procedure. The reaction of **2b** with **1a** for the synthesis of 3b was exemplified as follows.

Synthesis and characterization of 3b. To a stirred solution of **2b** (31.7 mg, 0.100 mmol) in tetrahydrofuran (0.5 mL) was added sodium hydride (60% oil suspension, 10.0 mg, 0.250 mmol) at -10 °C under an argon atmosphere. After stirring the solution at the same temperature for 10 min, **1a** (26.3 mg, 0.120 mmol) was added. The mixture was stirred for additional 1 hour, and then the reaction was quenched by addition of water (10 mL). The resulting mixture was extracted with dichloromethane (2 × 20 mL). The combined organic extracts were washed with brine (10 mL), dried over Na_2SO_4, filtered, and concentrated in vacuo to provide a crude material. This material was purified by column chromatography (silica gel, hexane/EtOAc = 4/1 to 2/1) to give **3b** (49.4 mg, 0.0971 mmol, 97%) as a white solid. R_f = 0.30 (silica gel, hexane/EtOAc = 2/1); m.p. 182–183 °C; IR (KBr) 3312 (N–H), 1695 (C=O), 1672 (C=O) cm^{-1}; ^1H NMR (300 MHz, $CDCl_3$) δ 8.30 (d, *J* = 7.8 Hz, 2H), 7.71 (d, *J* = 6.0 Hz, 1H), 7.49 (t, *J* = 7.8 Hz, 2H), 7.43–7.26 (m, 3H), 7.17–7.02 (m, 7H), 6.95 (d, *J* = 3.6 Hz, 4H), 5.45 (s, 1H), 4.82 (s, 1H), 3.95 (d, *J* = 14.4 Hz, 1H), 3.44 (d, *J* = 14.4 Hz, 1H); ^{13}C NMR (75 MHz, $CDCl_3$) δ 167.7, 167.0, 145.1, 139.6, 137.8, 137.0, 135.9, 132.1, 129.9, 129.2, 128.83, 128.78, 128.34, 128.32, 128.26, 126.2, 124.6, 124.3, 123.8, 122.8, 122.4, 119.7, 79.2, 35.6. Anal. Calcd for $C_{30}H_{24}N_2O_2S$: C, 75.61; H, 5.08; N, 5.88. Found: C, 75.23; H, 5.31; N, 5.80.

Synthesis and characterization of 3c. According to the synthetic procedure of **3b**, **3c** was synthesized from **2c** (66.3 mg, 0.200 mmol) and **1a** (52.6 mg, 0.240 mmol). The crude product was purified by column chromatography (silica gel, hexane/EtOAc = 4/1 to 3/1) to give **3c** (93.7 mg, 0.191 mmol, 95%) as a white solid. R_f = 0.37 (silica gel, hexane/EtOAc = 2/1); m.p. 120–121 °C; IR (KBr) 3296 (N–H), 1686 (C=O), 1670 (C=O) cm^{-1}; ^1H NMR (300 MHz, $CDCl_3$) δ 7.64 (t, *J* = 8.4 Hz, 3H), 7.46 (d, *J* = 6.9 Hz, 1H), 7.37–7.30 (m, 4H), 7.28–7.21 (m, 2H), 7.16 (t, *J* = 5.7 Hz, 3H), 7.12–7.03 (m, 2H), 6.95 (t, *J* = 7.5 Hz, 2H), 6.78 (d, *J* = 7.2 Hz, 2H), 6.47 (brs, 1H), 5.15 (d, *J* = 15.0 Hz, 1H), 5.01 (s, 1H), 4.81 (d, *J* = 15.0 Hz, 1H), 3.98 (s, 1H), 3.67 (d, *J* = 14.4 Hz, 1H), 3.08

(d, *J* = 14.4 Hz, 1H); ^{13}C NMR (75 MHz, $CDCl_3$) δ 168.4, 166.9, 144.9, 139.4, 138.3, 137.3, 135.5, 131.5, 130.6, 129.8, 129.2, 128.51, 128.46, 128.43, 128.39, 128.2, 127.5, 124.3, 124.1, 122.4, 121.2, 119.9, 79.5, 44.0, 36.7. Anal. Calcd for $C_{31}H_{26}N_2O_2S$: C, 75.89; H, 5.34; N, 5.71. Found: C, 76.07; H, 5.73; N, 5.64.

Synthesis and characterization of 3d. According to the synthetic procedure of **3b**, **3d** was synthesized from **2d** (51.1 mg, 0.200 mmol) and **1a** (52.6 mg, 0.240 mmol). The crude product was purified by column chromatography (silica gel, hexane/EtOAc = 4/1 to 2/1) to give **3d** (74.0 mg, 0.179 mmol, 90%) as a white solid. R_f = 0.37 (silica gel, hexane/EtOAc = 1/2); m.p. 155–156 °C; IR (KBr) 3300 (N–H), 1689 (C=O), 1669 (C=O) cm^{-1}; ^{1}H NMR (300 MHz, $CDCl_3$) δ 7.67 (d, *J* = 7.8 Hz, 1H), 7.38 (t, *J* = 7.5 Hz, 1H), 7.28 (t, *J* = 7.5 Hz, 1H), 7.24–7.15 (m, 4H), 7.11 (m, 1H), 7.08–7.02 (m, 2H), 6.96 (t, *J* = 7.8 Hz, 2H), 6.86 (d, *J* = 6.9 Hz, 2H), 5.44 (s, 1H), 5.12 (s, 1H), 3.63 (d, *J* = 13.8 Hz, 1H), 3.26 (d, *J* = 13.8 Hz, 1H), 3.19 (s, 3H); ^{13}C NMR (75 MHz, $CDCl_3$) δ 167.7, 166.7, 144.7, 140.4, 137.2, 135.7, 131.5, 129.3, 128.6, 128.53, 128.47, 128.39, 124.4 , 124.0, 122.3, 121.0, 120.2, 78.2, 36.8, 25.2. Anal. Calcd for $C_{25}H_{22}N_2O_2S$: C, 72.44; H, 5.35; N, 6.76. Found: C, 72.08; H, 5.40; N, 6.56.

Synthesis and characterization of 3e. According to the synthetic procedure of **3b**, **3e** was synthesized from **2e** (97.1 mg, 0.312 mmol) and **1a** (82.0 mg, 0.374 mmol). The crude product was purified by column chromatography (silica gel, hexane/EtOAc = 4/1 to 2/1) to give **3e** (135 mg, 0.287 mmol, 92%) as a white solid. R_f = 0.37 (silica gel, hexane/EtOAc = 2/1); m.p. 158–160 °C; IR (KBr) 3355 (N–H), 1686 (C=O), 1662 (C=O) cm^{-1}; ^{1}H NMR (300 MHz, $CDCl_3$) δ 7.53 (d, *J* = 7.5 Hz, 1H), 7.44 (d, *J* = 7.5 Hz, 1H), 7.34 (t, *J* = 7.5 Hz, 1H), 7.25 (d, *J* = 4.2 Hz, 3H), 7.13 (m, 3H), 7.02–6.91 (m, 5H), 5.47 (s, 1H), 5.11 (s, 1H), 3.65 (d, *J* = 14.4 Hz, 1H), 3.59 (m, 2H), 3.26 (d, *J* = 14.4 Hz, 1H), 1.94–1.80 (m, 2H), 1.40 (brs, 4H), 0.92 (t, *J* = 6.9 Hz, 3H); ^{13}C NMR (75 MHz, $CDCl_3$) δ 168.3, 166.8, 144.9, 140.4, 137.3, 134.9, 131.6, 131.5, 129.5, 129.0, 128.7, 128.6, 128.4, 124.4, 124.1, 122.4, 121.3, 120.1, 78.5, 41.3, 37.6, 29.8, 28.4, 22.4, 14.0. Anal. Calcd for $C_{29}H_{30}N_2O_2S$: C, 74.01; H, 6.43; N, 5.95. Found: C, 73.69; H, 6.58; N, 5.81.

Synthesis and characterization of 3f. According to the synthetic procedure of **3b**, **3f** was synthesized from **2b** (66.1 mg, 0.208 mmol) and **1b** [4] (54.8 mg, 0.250 mmol). The crude product was purified by column chromatography (silica gel, hexane/EtOAc = 4/1 to 2/1) to give **3f** (95.1 mg, 0.195 mmol, 94%) as a white solid. R_f = 0.33 (silica gel, hexane/EtOAc = 2/1); m.p. 178–179 °C; IR (KBr) 3316 (N–H), 1698 (C=O), 1664 (C=O) cm^{-1}; ^{1}H NMR (300 MHz, $CDCl_3$) δ 8.30–8.27 (m, 2H), 7.69 (m, 1H), 7.52–7.24 (m, 5H), 7.13–7.08 (m, 2H), 7.01–6.95 (m, 8H), 5.39 (s, 1H), 4.79 (s, 1H), 3.89 (d, *J* = 14.4 Hz, 1H), 3.43 (d, *J* = 14.4 Hz, 1H), 2.27 (s, 3H); ^{13}C NMR (75 MHz, $CDCl_3$) δ 167.6, 166.8, 145.1, 140.0, 137.0, 135.9, 135.0, 133.6, 132.2, 130.1, 129.3, 129.0, 128.8, 128.4, 126.2, 124.7, 124.2, 122.7, 121.9, 119.9, 79.1, 35.8, 20.8. Anal. Calcd for $C_{31}H_{26}N_2O_2S$: C, 75.89; H, 5.34; N, 5.71. Found: C, 75.73; H, 5.73; N, 5.67.

Synthesis and characterization of 3g. According to the synthetic procedure of **3b**, **3g** was synthesized from **2a** (64.3 mg, 0.203 mmol) and **1c** [4] (60.8 mg, 0.244 mmol). The crude product was purified by column chromatography (silica gel, hexane/EtOAc = 4/1 to 2/1) to give **3g** (97.8 mg, 0.193 mmol, 95%) as a white solid. R_f = 0.30 (silica gel, hexane/EtOAc = 2/1); m.p. 164–165 °C; IR (KBr) 3327 (N–H), 1699 (C=O), 1678 (C=O), 1662 (C=O) cm^{-1}; ^{1}H NMR (300 MHz, $CDCl_3$) δ 8.29 (d, *J* = 7.8 Hz, 2H), 7.70 (m, 1H), 7.52–7.40 (m, 3H), 7.32 (t, *J* = 7.2 Hz, 1H), 7.20–7.07 (m, 4H), 7.03–6.92 (m, 5H), 6.73–6.69 (m, 2H), 5.40 (s, 1H), 4.78 (s, 1H), 3.90 (d, *J* = 14.4 Hz, 1H), 3.76 (s, 3H), 3.43 (d, *J* = 14.4 Hz, 1H); ^{13}C NMR (75 MHz, $CDCl_3$) δ 167.7, 166.8, 156.1, 145.2, 140.0, 137.0, 135.9, 132.2, 130.7, 130.1, 129.3, 128.9, 128.8, 128.4, 126.2, 124.7, 124.3, 122.8, 121.9, 121.6, 113.6, 79.1, 55.3, 35.9. Anal. Calcd for $C_{31}H_{26}N_2O_3S$: C, 74.33; H, 6.02; N, 5.98. Found: C, 74.47; H, 6.15; N, 5.87.

Synthesis and characterization of 3h. According to the synthetic procedure of **3b**, **3h** was synthesized from **2a** (65.7 mg, 0.207 mmol) and **1d** (52.9 mg, 0.248 mmol). The crude product was purified by column chromatography (silica gel, hexane/EtOAc = 4/1 to 2/1) to give **3h** (91.1 mg, 0.193 mmol, 93%) as a white solid. R_f = 0.26 (silica gel, hexane/EtOAc = 2/1); m.p. 155–156 °C; IR (KBr) 3356 (N–H), 1699 (C=O), 1658 (C=O) cm^{-1}; ^{1}H NMR (300 MHz, $CDCl_3$) δ 8.24 (d, *J* =

7.8 Hz, 2H), 7.69 (d, *J* = 7.8 Hz, 1H), 7.57–7.22 (m, 6H), 7.15–7.08 (m, 1H), 6.99–6.93 (m, 4H), 5.16 (s, 1H), 5.00 (brs, 1H), 4.60 (s, 1H), 3.83 (d, *J* = 14.4 Hz, 1H), 3.36 (d, *J* = 14.4 Hz, 1H), 2.92–2.67 (m, 2H), 1.28–1.01 (m, 6H), 0.84 (t, *J* = 6.9 Hz, 3H); ^{13}C NMR (75 MHz, $CDCl_3$) δ 168.7, 167.5, 145.3, 140.0, 137.0, 135.9, 131.9, 130.4, 129.4, 128.83, 128.77, 128.4, 128.3, 126.1, 124.8, 124.3, 122.5, 121.0, 78.9, 39.5, 36.0, 28.9, 28.7, 22.2, 13.9. Anal. Calcd for $C_{29}H_{30}N_2O_2S$: C, 74.01; H, 6.43; N, 5.95. Found: C, 73.66; H, 6.25; N, 5.80.

Synthesis and characterization of 3i. According to the synthetic procedure of **3b**, **3i** was synthesized from **2f** (72.6 mg, 0.246 mmol) and **1a** (64.6 mg, 0.295 mmol). The crude product was purified by column chromatography (silica gel, hexane/EtOAc = 4/1 to 2/1) to give **3i** (107 mg, 0.235 mmol, 96%) as a white solid. R_f = 0.37 (silica gel, hexane/EtOAc = 2/1); m.p. 153–154 °C; IR (KBr) 3314 (N–H), 1692 (C=O), 1673 (C=O) cm^{-1}; ^{1}H NMR (300 MHz, $CDCl_3$) δ 8.23 (dd, *J* = 9.0, 1.5 Hz, 2H), 7.46–7.36 (m, 4H), 7.32-7.25 (m, 4H), 7.24–7.16 (m, 4H), 7.10 (m, 1H), 5.38 (s, 1H), 4.73 (s, 1H), 3.45 (dd, *J* = 14.4, 1.2 Hz, 1H), 3.17 (d, *J* = 14.4 Hz, 1H), 2.02 (q, *J* = 1.2 Hz, 3H), 1.29 (q, *J* = 1.2 Hz, 3H); ^{13}C NMR (75 MHz, $CDCl_3$) δ 170.6, 166.4, 150.4, 139.7, 137.6, 135.3, 130.0, 129.6, 129.4, 128.8, 128.71, 128.66, 125.2, 124.4, 123.3, 121.9, 120.0, 81.1, 34.0, 11.6, 8.0. Anal. Calcd for $C_{28}H_{26}N_2O_2S$: C, 73.98; H, 5.77; N, 6.16. Found: C, 74.35; H, 5.92; N, 6.35.

Synthesis and characterization of 3j. According to the synthetic procedure of **3b**, **3j** was synthesized from **2g** (72.3 mg, 0.234 mmol) and **1a** (61.6 mg, 0.281 mmol). The crude product was purified by column chromatography (silica gel, hexane/EtOAc = 4/1 to 2/1) to give **3j** (102 mg, 0.218 mmol, 93%) as a white solid. R_f = 0.33 (silica gel, hexane/EtOAc = 2/1); IR (KBr) 3424 (N–H), 1682 (C=O) cm^{-1}; ^{1}H NMR (300 MHz, $CDCl_3$) δ 7.54 (dd, *J* = 7.5, 1.8 Hz, 2H), 7.39 (dd, *J* = 8.7, 1.2 Hz, 2H), 7.32–7.24 (m, 6H), 7.18 (t, *J* = 7.8 Hz, 2H), 7.11 (d, *J* = 7.5 Hz, 1H), 7.07-7.03 (m, 2H), 6.86 (brs, 1H), 5.08 (s, 1H), 4.99 (d, *J* = 14.7 Hz, 1H), 4.63 (d, *J* = 14.7 Hz, 1H), 4.53 (s, 1H), 3.25 (d, *J* = 15.9 Hz, 1H), 2.83 (d, *J* = 15.9 Hz, 1H), 1.92 (s, 3H), 1.40 (s, 3H); ^{13}C NMR (75 MHz, $CDCl_3$) δ 171.9, 166.6, 149.6, 140.0, 138.5, 137.4, 135.2, 130.0, 129.8, 129.4, 129.2, 128.9,

128.6, 128.3, 127.4, 124.4, 119.8, 81.7, 44.4, 34.7, 11.4, 8.1. Anal. Calcd for $C_{29}H_{28}N_2O_2S$: C, 74.33; H, 6.02; N, 5.98. Found: C, 74.20; H, 6.41; N, 6.36.

Synthesis and characterization of 3k. According to the synthetic procedure of **3b**, **3k** was synthesized from **2h** (99.0 mg, 0.424 mmol) and **1a** (112 mg, 0.509 mmol). The crude product was purified by column chromatography (silica gel, hexane/EtOAc = 4/1 to 2/1) to give **3k** (153 mg, 0.390 mmol, 92%) as a white solid. R_f = 0.33 (silica gel, hexane/EtOAc = 1/1); m.p. 177–178 °C; IR (KBr) 3291 (N–H), 1685 (C=O), 1670 (C=O) cm^{-1}; ^{1}H NMR (300 MHz, $CDCl_3$) δ 7.46 (brs, 1H), 7.43 (d, J = 8.4 Hz, 2H), 7.31–7.25 (m, 3H), 7.20 (t, J = 7.8 Hz, 2H), 7.10 (t, J = 7.2 Hz, 3H), 5.57 (s, 1H), 5.33 (s, 1H), 3.31 (d, J = 14.1 Hz, 1H), 3.09 (s, 3H), 2.89 (d, J = 14.1 Hz, 1H), 1.95 (s, 3H), 1.30 (s, 3H); ^{13}C NMR (75 MHz, $CDCl_3$) δ 170.9, 166.5, 148.9, 140.8, 137.6, 135.0, 131.0, 129.3, 128.7, 128.6, 124.4, 120.6, 120.3, 80.4, 35.4, 25.5, 11.4, 8.1. Anal. Calcd for $C_{23}H_{24}N_2O_2S$: C, 70.38; H, 6.16; N, 7.14. Found: C, 70.04; H, 6.07; N, 6.93.

Synthesis and characterization of 3l. According to the synthetic procedure of **3b**, **3l** was synthesized from **2i** (57.9 mg, 0.200 mmol) and **1a** (52.6 mg, 0.240 mmol). The crude product was purified by column chromatography (silica gel, hexane/EtOAc = 4/1 to 2/1) to give **3l** (86.1 mg, 0.192 mmol, 96%) as a white solid. R_f = 0.35 (silica gel, hexane/EtOAc = 2/1); m.p. 156–157 °C; IR (KBr) 3344 (N–H), 1679 (C=O), 1664 (C=O) cm^{-1}; ^{1}H NMR (300 MHz, $CDCl_3$) δ 7.47 (dd, J = 8.7, 1.2 Hz, 2H), 7.40 (brs, 1H), 7.31 (t, J = 7.5 Hz, 2H), 7.26–7.22 (m, 2H), 7.20 (t, J = 1.8 Hz, 1H), 7.19–7.17 (m, 2H), 7.12 (m, 1H), 5.60 (s, 1H), 5.32 (s, 1H), 3.52 (m, 1H), 3.37 (m, 1H), 3.30 (dd, J = 15.0, 0.9 Hz, 1H), 2.95 (d, J = 15.0 Hz, 1H), 1.84 (q, J = 0.9 Hz, 3H), 1.82–1.74 (m, 2H), 1.40 (q, J = 0.9 Hz, 3H), 1.37–1.31 (m, 4H), 0.89 (t, J = 6.9 Hz, 3H); ^{13}C NMR (75 MHz, $CDCl_3$) δ 171.5, 166.4, 148.9, 140.8, 137.4, 133.8, 131.2, 130.5, 128.92, 128.86, 128.5, 124.6, 120.5, 120.1, 80.7, 41.5, 35.4, 29.7, 28.2, 22.3, 14.0, 11.4, 8.1. Anal. Calcd for $C_{27}H_{32}N_2O_2S$: C, 72.29; H, 7.19; N, 6.24. Found: C, 72.38; H, 6.84; N, 6.12.

Synthesis and characterization of 3m. According to the synthetic procedure of **3b**, **3m** was synthesized from **2f** (59.7 mg, 0.202 mmol) and **1b** (56.5 mg, 0.242 mmol). The crude product was

purified by column chromatography (silica gel, hexane/EtOAc = 4/1 to 2/1) to give **3m** (87.0 mg, 0.186 mmol, 92%) as a white solid. R_f = 0.33 (silica gel, hexane/EtOAc = 2/1); m.p. 170–171 °C; IR (KBr) 3325 (N–H), 1696 (C=O), 1660 (C=O) cm^{-1}; ^{1}H NMR (300 MHz, $CDCl_3$) δ 8.25–8.22 (m, 2H), 7.46–7.41 (m, 2H), 7.32–7.16 (m, 8H), 7.09 (d, *J* = 8.4 Hz, 2H), 5.36 (s, 1H), 4.70 (s, 1H), 3.45 (d, *J* = 14.4 Hz, 1H), 3.16 (d, *J* = 14.4 Hz, 1H), 2.30 (s, 3H), 2.02 (s, 3H), 1.31 (s, 3H); ^{13}C NMR (75 MHz, $CDCl_3$) δ 170.6, 166.4, 150.4, 139.8, 137.6, 135.3, 134.9, 134.1, 130.0, 129.6, 129.4, 129.3, 128.7, 128.6, 125.2, 123.3, 121.6, 120.1, 81.0, 34.1, 20.8, 11.6, 8.0. Anal. Calcd for $C_{29}H_{28}N_2O_2S$: C, 74.33; H, 6.02; N, 5.98. Found: C, 74.47; H, 6.15; N, 5.87.

Synthesis and characterization of 3n. According to the synthetic procedure of **3b**, **3n** was synthesized from 2f (59.5 mg, 0.201 mmol) and **1c** (60.1 mg, 0.241 mmol). The crude product was purified by column chromatography (silica gel, hexane/EtOAc = 4/1 to 2/1) to give **3n** (92.6 mg, 0.191 mmol, 95%) as a white solid. R_f = 0.33 (silica gel, hexane/EtOAc = 2/1); m.p. 153–154 °C; IR (KBr) 3328 (N–H), 1684 (C=O), 1660 (C=O) cm^{-1}; ^{1}H NMR (300 MHz, $CDCl_3$) δ 8.24–8.21 (m, 2H), 7.54–7.39 (m, 2H), 7.31–7.16 (m, 8H), 6.83–6.78 (m, 2H), 5.37 (s, 1H), 4.70 (s, 1H), 3.77 (s, 3H), 3.45 (d, *J* = 14.7 Hz, 1H), 3.15 (d, *J* = 14.7 Hz, 1H), 2.02 (d, *J* = 0.9 Hz, 3H), 1.30 (d, *J* = 0.9 Hz, 3H); ^{13}C NMR (75 MHz, $CDCl_3$) δ 170.7, 166.4, 156.5, 150.4, 139.8, 137.6, 135.3, 130.5, 129.9, 129.6, 129.4, 128.7, 128.6, 125.2, 123.3, 121.9, 121.5, 114.0, 81.0, 55.4, 34.2, 11.6, 8.0. Anal. Calcd for $C_{29}H_{28}N_2O_3S$: C, 71.88; H, 5.82; N, 5.78. Found: C, 72.01; H, 5.83; N, 5.63.

Synthesis and characterization of 3o. According to the synthetic procedure of **3b**, **3o** was synthesized from **2f** (61.8 mg, 0.209 mmol) and **1d** (53.5 mg, 0.251 mmol). The crude product was purified by column chromatography (silica gel, hexane/EtOAc = 4/1 to 2/1) to give **3o** (92.9 mg, 0.207 mmol, 99%) as a white solid. R_f = 0.37 (silica gel, hexane/EtOAc = 2/1); m.p. 110–111 °C; IR (KBr) 3397 (N–H), 1684 (C=O), 1661 (C=O) cm^{-1}; ^{1}H NMR (300 MHz, $CDCl_3$) δ 8.19–8.17 (m, 2H), 7.42 (t, *J* = 7.5 Hz, 2H), 7.31–7.16 (m, 6H), 5.49 (t, *J* = 5.4 Hz, 1H), 5.17 (s, 1H), 4.55 (s, 1H), 3.39 (d, *J* = 14.4 Hz, 1H), 3.13–3.05 (m, 3H), 2.04 (d, *J* = 0.6 Hz, 3H), 1.45–1.16 (m, 6H), 1.40 (d, *J* = 0.6 Hz, 3H), 0.88 (t, *J* = 7.2 Hz, 3H); ^{13}C NMR (75 MHz, $CDCl_3$) δ 170.6, 168.3, 150.3, 139.7,

137.6, 135.4, 129.6, 129.5, 128.6, 125.1, 123.2, 120.7, 81.0, 39.6, 34.1, 29.2, 28.9, 22.2, 13.9, 11.5, 8.0. Anal. Calcd for $C_{27}H_{32}N_2O_2S$: C, 72.29; H, 7.19; N, 6.24. Found: C, 72.58; H, 7.20; N, 6.15.

Synthesis and characterization of 3p. According to the synthetic procedure of **3b**, **3p** was synthesized from **2d** (255 mg, 1.00 mmol) and **1b** (280 mg, 1.20 mmol). The crude product was purified by column chromatography (silica gel, hexane/EtOAc = 2/1 to 1/2) to give **3p** (364 mg, 0.849 mmol, 85%) as a white solid. R_f = 0.33 (silica gel, hexane/EtOAc = 1/2); m.p. 185–186 °C; IR (KBr) 3278 (N–H) 1696 (C=O) cm^{-1}; ^{1}H NMR (300 MHz, $CDCl_3$) δ 7.67 (m, 1H), 7.44–7.37 (m, 2H), 7.19–6.95 (m, 8H), 6.89–6.85 (m, 2H), 6.73 (brs, 1H), 5.39 (s, 1H), 5.09 (s, 1H), 3.59 (dd, *J* = 14.1, 0.6 Hz, 1H), 3.27 (d, *J* = 14.1 Hz, 1H), 3.20 (s, 3H), 2.28 (s, 3H); ^{13}C NMR (75 MHz, $CDCl_3$) δ 167.6, 166.6, 144.7, 140.4, 135.7, 134.6, 134.0, 131.4, 129.3, 129.1, 128.5, 128.3, 124.0, 122.2, 120.9, 120.4, 78.3, 36.7, 25.1, 20.8. Anal. Calcd for $C_{26}H_{24}N_2O_2S$: C, 72.87; H, 5.65; N, 6.54. Found: C, 72.96; H, 5.71; N, 6.21.

General procedure for the synthesis of 4a–n. All the experiments for the synthesis of **4a–n** were carried out as described in the following typical procedure. The reaction of **3b** for the synthesis of **4a** was exemplified as follows.

Synthesis and characterization of 4a. To a stirred solution of **3b** (47.7 mg, 0.100 mmol) in tetrahydrofuran/water (1/1, 1.0 mL) was added copper (I) bromide (43.2 mg, 0.301 mmol) at room temperature. After stirring the solution at the same temperature for 4 hours, water (1.0 mL) was added to the solution and the resulting mixture was extracted with ethyl acetate (2 × 10 mL). The combined organic extracts were washed with brine (10 mL), dried over Na_2SO_4, filtered, and concentrated in vacuo to provide a crude material. This material was purified by column chromatography (silica gel, CH_2Cl_2/EtOAc = 4/1) to give **6a** (34.7 mg, 0.0903 mmol, 90%) as a white solid. R_f = 0.30 (silica gel, hexane/EtOAc = 1/1); m.p. 177–178 °C; IR (KBr) 3444 (O–H), 3366 (N–H), 1664 (C=O) cm^{-1}; ^{1}H NMR (300 MHz, $CDCl_3$/CD_3OD = 1/1) δ 7.81 (d, *J* = 7.5 Hz, 1H), 7.71 (d, *J* = 7.5 Hz, 2H), 7.64 (d, *J* = 7.5 Hz, 2H), 7.55 (m, 1H), 7.49–7.45 (m, 3H), 7.39–7.32 (m, 3H), 7.27 (t, *J* = 7.5 Hz, 1H), 7.09 (m, 1H), 5.79 (s, 1H), 5.18 (s, 1H), 3.38 (d, *J* = 14.7 Hz, 1H),

2.89 (d, J = 14.7 Hz, 1H); ^{13}C NMR (75 MHz, $CDCl_3/CD_3OD$ = 1/1) δ 167.7, 167.1, 145.4, 138.9, 137.2, 134.9, 132.0, 130.0, 129.2, 128.3, 128.0, 126.8, 126.6, 124.0, 123.9, 123.2, 122.6, 120.2, 91.6, 39.2. Anal. Calcd for $C_{24}H_{20}N_2O_3$: C, 74.98; H, 5.24; N, 7.29. Found: C, 74.66; H, 5.41; N, 7.10.

To a solution of **6a** (38.9 mg, 0.101 mmol) in dichloromethane (1.0 mL) was added *p*-toluenesulfonic acid monohydrate (21.4 mg, 0.121 mmol) at room temperature. After stirring the solution at the same temperature for 24 hours, the reaction was quenched with *sat.* $NaHCO_3$ aq (1.0 mL). The resulting mixture was extracted with dichloromethane (2 × 10 mL). The combined organic extracts were washed with brine (10 mL), dried over Na_2SO_4, filtered, and concentrated in vacuo to provide a crude material. This material was purified by column chromatography (silica gel, hexane/EtOAc = 1/1) to give **4a** (36.0 mg, 0.0982 mmol, 97%) as a white solid. R_f = 0.32 (silica gel, hexane/EtOAc = 1/1); m.p. 218–219 °C; IR (KBr) 1714 (C=O) cm^{-1}; ^{1}H NMR (300 MHz, $CDCl_3$) δ 7.85 (d, J = 8.1 Hz, 1H), 7.56 (m, 1H), 7.50–7.32 (m, 7H), 7.23–7.10 (m, 5H), 6.10 (t, J = 2.7 Hz, 1H), 5.33 (t, J = 2.1 Hz, 1H), 3.37 (dt, J = 17.4, 2.7 Hz, 1H), 3.20 (dt, J = 17.4, 2.1 Hz, 1H); ^{13}C NMR (75 MHz, $CDCl_3$) δ 167.2, 166.6, 144.7, 135.8, 135.4, 134.6, 133.3, 131.2, 130.0, 129.6, 129.0, 127.9, 127.3, 127.0, 125.3, 124.1, 121.8, 118.2, 83.0, 37.5. Anal. Calcd for $C_{24}H_{18}N_2O_2$: C, 78.67; H, 4.95; N, 7.65. Found: C, 78.27; H, 4.94; N, 7.52.

Synthesis and characterization of 4b. According to the synthetic procedure of **6a**, **6b** was synthesized from **3c** (48.9 mg, 0.100 mmol). The crude product was purified by column chromatography (silica gel, hexane/EtOAc = 2/1 to $CHCl_3$/MeOH = 10/1) to give **6b** (35.2 mg, 0.0886 mmol, 89%) as a white solid. R_f = 0.23 (silica gel, hexane/EtOAc = 1/1); m.p. 89–90 °C; IR (KBr) 3302 (O–H), 1684 (C=O) cm^{-1}; ^{1}H NMR (300 MHz, $CDCl_3$) δ 7.90 (s, 1H), 7.73 (d, J = 6.9 Hz, 1H), 7.47–7.13 (m, 13H), 5.95 (s, 1H), 5.78 (s, 1H), 5.08 (s, 1H), 4.78 (t, J = 15.6 Hz, 1H), 4.65 (d, J = 15.6 Hz, 1H), 3.21 (d, J = 13.8 Hz, 1H), 2.60 (d, J = 13.8 Hz, 1H); ^{13}C NMR (75 MHz, $CDCl_3$) δ 167.6, 167.4, 145.1, 137.4, 136.1, 134.4, 132.7, 131.1, 130.0, 129.0, 128.6, 127.9, 127.8, 127.7, 126.7, 123.7, 121.8, 118.9, 82.5, 42.5, 36.5. Anal. Calcd for $C_{25}H_{22}N_2O_3$: C, 75.36; H, 5.57;

N, 7.03. Found: C, 75.26; H, 5.38; N, 6.92.

According to the synthetic procedure of **4a**, **4b** was synthesized from **6b** (39.7 mg, 0.100 mmol). The crude product was purified by column chromatography (silica gel, hexane/EtOAc = 2/1) to give **4b** (35.2 mg, 0.0886 mmol, 89%) as a white solid. R_f = 0.33 (silica gel, hexane/EtOAc = 1/1); m.p. 210–211 °C; IR (KBr) 1714 (C=O), 1698 (C=O) cm^{-1}; ^{1}H NMR (300 MHz, $CDCl_3$) δ 7.76 (dd, *J* = 6.3, 1.2 Hz, 1H), 7.57 (m, 1H), 7.46 (t, *J* = 7.8 Hz, 2H), 7.38–7.26 (m, 5H), 7.18–7.13 (m, 3H), 6.81–6.77 (m, 2H), 6.35 (t, *J* = 2.4 Hz, 1H), 5.40 (t, *J* = 2.4 Hz, 1H), 5.23 (d, *J* = 15.6 Hz, 1H), 4.15 (d, *J* = 15.6 Hz, 1H), 3.23 (dt, *J* = 17.7, 2.4 Hz, 1H), 2.93 (dt, *J* = 17.7, 2.4 Hz, 1H); ^{13}C NMR (75 MHz, $CDCl_3$) δ 167.5, 167.4, 145.0, 137.3, 136.1, 134.4, 132.7, 131.1, 130.0, 129.0, 128.6, 127.9, 127.8, 127.7, 126.6, 123.7, 121.8, 118.9, 82.5, 42.4, 36.4. Anal. Calcd for $C_{25}H_{20}N_2O_2$: C, 78.93; H, 5.30; N, 7.36. Found: C, 78.74; H, 5.16; N, 7.38.

Synthesis and characterization of 4c. According to the synthetic procedure of **6a**, **6c** was synthesized from **3d** (41.5 mg, 0.100 mmol). The crude product was purified by column chromatography (silica gel, hexane/EtOAc = 1/1) to give **6c** (30.8 mg, 0.0955 mmol, 95%) as a white solid. R_f = 0.23 (silica gel, hexane/EtOAc = 1/3); m.p. 176–177 °C; IR (KBr) 3303 (O–H), 1691 (C=O), 1664 (C=O) cm^{-1}; ^{1}H NMR (300 MHz, $CDCl_3/CD_3OD$ = 1/1) δ 7.69 (d, *J* = 7.5 Hz, 1H), 7.61 (m, 1H), 7.51 (m, 1H), 7.41 (m, 1H), 7.35–7.24 (m, 4H), 7.08 (m, 1H), 5.63 (s, 1H), 5.21 (s, 1H), 3.34 (d, *J* = 12.6 Hz, 1H), 3.26 (d, *J* = 13.5 Hz, 1H), 3.05 (s, 3H); ^{13}C NMR (75 MHz, $CDCl_3/CD_3OD$ = 1/1) δ 167.6, 167.4, 145.6, 139.1, 137.1, 131.4, 130.7, 128.9, 127.9, 123.9, 122.5, 122.3, 121.9, 120.5, 89.6, 37.9, 22.9. Anal. Calcd for $C_{19}H_{18}N_2O_3$: C, 70.79; H, 5.63; N, 8.69. Found: C, 70.99; H, 5.78; N, 8.52.

According to the synthetic procedure of **4a**, **4c** was synthesized from **6c** (32.2 mg, 0.100 mmol). The crude product was purified by column chromatography (silica gel, hexane/EtOAc = 1/1 to 1/2) to give **4c** (29.3 mg, 0.0962 mmol, 96%) as a white solid. R_f = 0.33 (silica gel, hexane/EtOAc = 1/3); m.p. 232–233 °C; IR (KBr) 1697 (C=O), 1658 (C=O) cm^{-1}; ^{1}H NMR (300 MHz, $CDCl_3$) δ 7.72 (d, *J* = 7.5 Hz, 1H), 7.57 (t, *J* = 7.5 Hz, 1H), 7.46 (q, *J* = 6.3 Hz, 2H), 7.20–7.11 (m, 3H),

6.84–6.81 (m, 2H), 6.40 (t, J = 2.7 Hz, 1H), 5.68 (t, J = 2.1 Hz, 1H), 3.40 (dt, J = 17.7, 2.7 Hz, 1H), 3.19 (dt, J = 17.7, 2.1 Hz, 1H), 3.03 (s, 3H); ^{13}C NMR (75 MHz, $CDCl_3$) δ 167.3, 166.6, 144.9, 136.1, 134.5, 132.5, 131.4, 129.9, 129.0, 127.9, 126.4, 123.5, 121.8, 118.8, 81.7, 36.1, 24.1. Anal. Calcd for $C_{19}H_{16}N_2O_2$: C, 74.98; H, 5.30; N, 9.20. Found: C, 74.72; H, 5.69; N, 9.10.

Synthesis and characterization of 4d. According to the synthetic procedure of **6a**, **6d** was synthesized from **3e** (47.1 mg, 0.100 mmol). The crude product was purified by column chromatography (silica gel, hexane/EtOAc = 2/1 to $CHCl_3$/MeOH = 10/1) to give **6d** (34.8 mg, 0.0919 mmol, 92%) as a white solid. R_f = 0.32 (silica gel, hexane/EtOAc = 1/1); m.p. 170–171 °C; IR (KBr) 3358 (N–H), 3211 (O–H), 1657 (C=O) cm^{-1}; ^{1}H NMR (300 MHz, $CDCl_3$/CD_3OD = 1/1) δ 7.68 (d, J = 7.2 Hz, 1H), 7.59 (t, J = 9.9 Hz, 1H), 7.49 (t, J = 7.5 Hz, 1H), 7.41 (dd, J = 7.2, 0.9 Hz, 1H), 7.37–7.34 (m, 2H), 7.27 (t, J = 7.5 Hz, 2H), 7.09 (m, 1H), 5.67 (s, 1H), 5.18 (s, 1H), 3.63 (m, 1H), 3.37 (d, J = 14.1 Hz, 1H), 3.37–3.27 (m, 1H), 3.09 (d, J = 14.1 Hz, 1H), 1.87–1.69 (m, 2H), 1.40–1.35 (m, 4H), 0.92 (t, J = 6.6 Hz, 3H); ^{13}C NMR (75 MHz, $CDCl_3$/CD_3OD = 1/1) δ 167.6, 167.5, 145.5, 139.1, 137.2, 131.3, 130.9, 128.9, 127.9, 123.8, 122.6, 122.5, 121.8, 120.4, 90.4, 39.0, 38.4, 28.9, 28.1, 21.7, 13.0. Anal. Calcd for $C_{23}H_{26}N_2O_3$: C, 72.99; H, 6.92; N, 7.40. Found: C, 72.93; H, 6.64; N, 7.23.

According to the synthetic procedure of **4a**, **4d** was synthesized from **6d** (37.8 mg, 0.100 mmol). The crude product was purified by column chromatography (silica gel, hexane/EtOAc = 1/1) to give **4d** (33.8 mg, 0.0938 mmol, 94%) as a white solid. R_f = 0.37 (silica gel, hexane/EtOAc = 1/1); m.p. 98–100 °C; IR (KBr) 1713 (C=O), 1697 (C=O) cm^{-1}; ^{1}H NMR (300 MHz, $CDCl_3$) δ 7.70 (d, J = 7.8 Hz, 1H), 6.93 (t, J = 7.2 Hz, 1H), 7.44 (dd, J = 6.6, 5.1 Hz, 2H), 7.18–7.08 (m, 3H), 6.84–6.81 (m, 2H), 6.39 (t, J = 2.7 Hz, 1H), 5.68 (t, J = 2.1 Hz, 1H), 3.58 (m, 1H), 3.44 (dt, J = 17.4, 2.7 Hz, 1H), 3.25 (dt, J = 17.4, 2.1 Hz, 1H), 3.18 (m, 1H), 1.82 (m, 1H), 1.62 (m, 1H), 1.40–1.26 (m, 4H), 0.90 (t, J = 6.9 Hz, 3H); ^{13}C NMR (75 MHz, $CDCl_3$) δ 167.4, 167.0, 144.8, 136.4, 134.7, 132.5, 131.6, 130.0, 128.9, 127.7, 126.2, 123.4, 121.7, 118.5, 82.2, 39.8, 37.5, 29.5, 28.5, 22.2, 13.9. Anal. Calcd for $C_{23}H_{24}N_2O_2$: C, 76.64; H, 6.71; N, 7.77. Found: C, 76.35; H, 6.79; N, 7.92.

Synthesis and characterization of 4e. According to the synthetic procedure of **6a**, **6e** was synthesized from **3f** (94.8 mg, 0.194 mmol). The crude product was purified by column chromatography (silica gel, hexane/EtOAc = 3/1 to $CHCl_3$/MeOH = 30/1) to give **6e** (69.5 mg, 0.174 mmol, 90%) as a white solid. R_f = 0.27 (silica gel, hexane/EtOAc = 1/1); m.p. 194–195 °C; IR (KBr) 3325 (N–H), 1691 (C=O), 1661 (C=O) cm^{-1}; ^1H NMR (300 MHz, $CDCl_3/CD_3OD$=1/1) δ 7.81 (m, 1H), 7.71–7.67 (m, 2H), 7.63–7.44 (m, 5H), 7.36 (m, 1H), 7.25–7.20 (m, 2H), 7.08 (d, J = 8.1 Hz, 2H), 5.82 (s, 1H), 5.18 (s, 1H), 3.34 (d, J = 14.1 Hz, 1H), 2.83 (d, J = 14.1 Hz, 1H), 2.30 (s, 3H); ^{13}C NMR (75 MHz, $CDCl_3/CD_3OH$ = 1/1) δ 167.6, 167.1, 145.4, 138.8, 134.9, 134.6, 133.6, 132.0, 130.0, 129.2, 128.5, 128.4, 126.8, 126.7, 124.1, 123.3, 122.7, 120.3, 91.6, 39.4, 19.9. Anal. Calcd for $C_{25}H_{22}N_2O_3$: C, 75.36; H, 5.57; N, 7.03. Found: C, 75.19; H, 5.51; N, 6.91.

According to the synthetic procedure of **4a**, **4e** was synthesized from **6e** (56.4 mg, 0.142 mmol). The crude product was purified by column chromatography (silica gel, hexane/EtOAc = 3/1 to 1/1) to give **4e** (53.5 mg, 0.141 mmol, 99%) as a white solid. R_f = 0.33 (silica gel, hexane/EtOAc = 1/1); m.p. 206–207 °C; IR (KBr) 1705 (C=O), 1671 (C=O) cm^{-1}; ^1H NMR (300 MHz, $CDCl_3$) δ 7.84 (d, J = 7.5 Hz, 1H), 7.59–7.31 (m, 8H), 7.03–6.96 (m, 4H), 6.09 (t, J = 2.7 Hz, 1H), 5.32 (t, J = 2.7 Hz, 1H), 3.36 (dt, J = 17.4, 2.7 Hz, 1H), 3.19 (dt, J = 17.4, 2.7 Hz, 1H), 2.21 (s, 3H); ^{13}C NMR (75 MHz, $CDCl_3$) δ 167.2, 166.6, 144.8, 137.1, 135.8, 134.7, 133.2, 132.6, 131.1, 129.9, 129.5, 127.7, 126.8, 125.2, 124.0, 121.8, 117.9, 83.0, 37.3, 20.8. Anal. Calcd for $C_{25}H_{20}N_2O_2$: C, 78.93; H, 5.30; N, 7.36. Found: C, 79.26; H, 5.37; N, 7.39.

Synthesis and characterization of 4f. According to the synthetic procedure of **6a**, **6f** was synthesized from **3g** (50.7 mg, 0.100 mmol). The crude product was purified by column chromatography (silica gel, hexane/EtOAc = 2/1 to $CHCl_3$/MeOH = 10/1) to give **6f** (37.4 mg, 0.0902 mmol, 90%) as a white solid. R_f = 0.25 (silica gel, hexane/EtOAc = 1/1); m.p. 177–178 °C; IR (KBr) 3337 (N–H), 3302 (O–H), 1665 (C=O), 1657 (C=O) cm^{-1}; ^1H NMR (300 MHz, $CDCl_3/CD_3OD$=1/1) δ 7.81 (m, 1H), 7.73-7.70 (m, 2H), 7.65-7.45 (m, 5H), 7.36 (m, 1H), 7.25-7.20 (m, 2H), 6.85-6.79 (m, 2H), 5.76 (s, 1H), 5.16 (s, 3H), 3.79 (s, 3H), 3.37 (d, J = 13.8 Hz, 1H), 2.88

(d, J = 13.8 Hz, 1H) ; ^{13}C NMR (75 MHz, $CDCl_3/CD_3OH$ = 1/1) δ 167.5, 167.0, 156.0, 145.4, 138.8, 134.9, 131.9, 130.2, 130.0, 129.1, 128.2, 126.7, 126.6, 123.6, 123.2, 122.6, 122.0, 113.1, 91.6, 54.5, 39.1. Anal. Calcd for $C_{25}H_{22}N_2O_4$: C, 72.45; H, 5.35; N, 6.76. Found: C, 72.73; H, 5.42; N, 6.73.

According to the synthetic procedure of **4a**, **4f** was synthesized from **6f** (37.4 mg, 0.0902 mmol). The crude product was purified by column chromatography (silica gel, hexane/EtOAc = 2/1 to 1/1) to give **4f** (35.5 mg, 0.0895 mmol, 99%) as a white solid. R_f = 0.32 (silica gel, hexane/EtOAc = 1/1); m.p. 91–92 °C; IR (KBr) 1704 (C=O), 1670 (C=O) cm^{-1}; ^{1}H NMR (300 MHz, $CDCl_3$) δ 7.83 (d, J = 2.4 Hz, 1H), 7.58 (m, 1H), 7.50-7.31 (m, 7H), 7.05-7.00 (m, 2H), 6.73-6.67 (m, 2H), 6.11 (t, J = 2.7 Hz, 1H), 5.34 (t, J = 2.1 Hz, 1H), 3.69 (s, 3H), 3.37 (dt, J = 17.7, 2.7 Hz, 1H), 3.20 (dt, J = 17.7, 2.1 Hz, 1H); ^{13}C NMR (75 MHz, $CDCl_3$) δ 167.3, 166.6, 158.3, 144.9, 135.9, 134.8, 133.2, 131.1, 129.9, 129.6, 127.8, 127.6, 126.9, 126.6, 124.0, 121.8, 117.9, 114.2, 83.0, 55.2, 36.9. Anal. Calcd for $C_{25}H_{20}N_2O_3$: C, 75.74; H, 5.09; N, 7.07. Found: C, 75.40; H, 4.94; N, 6.98.

Synthesis and characterization of 4g. According to the synthetic procedure of **6a**, **6g** was synthesized from **3h** (120 mg, 0.255 mmol). The crude product was purified by column chromatography (silica gel, hexane/EtOAc = 3/2) to give **6g** (89.2 mg, 0.236 mmol, 93%) as a white solid. R_f = 0.26 (silica gel, hexane/EtOAc = 1/1); m.p. 170–171 °C; IR (KBr) 3357 (N–H), 3223 (O–H), 1691 (C=O), 1652 (C=O) cm^{-1}; ^{1}H NMR (300 MHz, $CDCl_3/CD_3OD$=1/1) δ 7.80 (m, 1H), 7.69–7.44 (m, 7H), 7.36 (m, 1H), 5.64 (s, 1H), 5.05 (s, 1H), 3.26 (d, J = 13.8 Hz, 1H), 3.06–2.88 (m, 2H), 2.76 (d, J = 13.8 Hz, 1H), 1.41–1.16 (m, 6H), 0.90 (t, J = 6.9 Hz, 3H); ^{13}C NMR (75 MHz, $CDCl_3/CD_3OH$ = 1/1) δ 169.3, 166.9, 145.6, 138.3, 134.9, 131.7, 129.9, 129.0, 128.2, 126.6, 123.2, 122.5, 91.3, 39.4, 39.0, 28.4, 28.0, 21.6, 12.9. Anal. Calcd for $C_{23}H_{26}N_2O_3$: C, 72.99; H, 6.92; N, 7.40. Found: C, 73.27; H, 7.20; N, 7.00.

According to the synthetic procedure of **4a**, **4g** was synthesized from **6g** (61.1 mg, 0.161 mmol). The crude product was purified by column chromatography (silica gel, hexane/EtOAc = 2/1) to give **4g** (52.3 mg, 0.145 mmol, 90%) as a white solid. R_f = 0.38 (silica gel, hexane/EtOAc = 1/1); m.p. 115–116 °C; IR (KBr) 1719 (C=O), 1698 (C=O) cm^{-1}; ^{1}H NMR (300 MHz, $CDCl_3$) δ 7.96 (m, 1H),

7.70–7.58 (m, 2H), 7.49–7.30 (m, 4H), 7.17–7.13 (m, 2H), 5.94 (t, *J* = 2.7 Hz, 1H), 5.20 (t, *J* = 2.4 Hz, 1H), 3.44 (m, 1H), 3.23 (dt, *J* = 17.7, 2.7 Hz, 1H), 3.04 (dt, *J* = 17.7, 2.4 Hz, 1H), 2.76 (m, 1H), 1.49 (m, 1H), 1.25–0.92 (m, 5H), 0.72 (t, *J* = 6.9 Hz, 3H); ^{13}C NMR (75 MHz, $CDCl_3$) δ 167.9, 166.7, 145.1, 135.8, 134.2, 132.9, 131.1, 130.3, 129.6, 128.0, 127.3, 124.2, 122.4, 116.4, 82.5, 40.6, 36.0, 29.0, 27.8, 21.8, 13.6. Anal. Calcd for $C_{23}H_{24}N_2O_2$: C, 76.64; H, 6.71; N, 7.77. Found: C, 76.73; H, 6.42; N, 7.66.

Synthesis and characterization of 4h. According to the synthetic procedure of **6a**, **6h** was synthesized from **3i** (50.9 mg, 0.112 mmol). The crude product was purified by column chromatography (silica gel, hexane/EtOAc = 1/1) to give **6h** (36.8 mg, 0.102 mmol, 91%) as a white solid. R_f = 0.37 (silica gel, hexane/EtOAc = 1/1); m.p. 188–189 °C; IR (KBr) 3386 (O–H), 3303 (N–H), 1685 (C=O), 1679 (C=O), 1655 (C=O) cm^{-1}; ^{1}H NMR (300 MHz, $CDCl_3/CD_3OD$ = 1/1) δ 7.75 (dd, *J* = 9.0, 1.5 Hz, 2H), 7.51 (dd, *J* = 9.0, 1.5 Hz, 2H), 7.39 (dd, *J* = 7.2, 1.8 Hz, 1H), 7.36–7.29 (m, 3H), 7.24 (m, 1H), 7.12 (m, 1H), 5.63 (s, 1H), 5.01 (s, 1H), 3.17 (dd, *J* = 14.1, 0.9 Hz, 1H), 2.83 (d, *J* = 14.1, 0.9 Hz, 1H), 1.99 (d, *J* = 0.9 Hz, 3H), 1.73 (d, *J* = 0.9 Hz, 3H); ^{13}C NMR (75 MHz, $CDCl_3/CD_3OH$ = 1/1) δ 170.3, 167.4, 152.2, 138.9, 137.4, 135.8, 128.2, 128.1, 127.9, 125.4, 124.7, 123.9, 122.6, 120.2, 92.4, 36.7, 9.7, 7.2. Anal. Calcd for $C_{22}H_{22}N_2O_3$: C, 72.91; H, 6.12; N, 7.73. Found: C, 72.56; H, 5.72; N, 7.64.

According to the synthetic procedure of **4a**, **4h** was synthesized from **6h** (36.9 mg, 0.102 mmol). The crude product was purified by column chromatography (silica gel, hexane/EtOAc = 3/2) to give **4h** (34.2 mg, 0.0993 mmol, 97%) as a white solid. R_f = 0.27 (silica gel, hexane/EtOAc = 1/1); m.p. 151–152 °C; IR (KBr) 1703 (C=O), 1661 (C=O) cm^{-1}; ^{1}H NMR (300 MHz, $CDCl_3$) δ 7.40–7.24 (m, 10H), 6.05 (t, *J* = 2.4 Hz, 1H), 5.29 (t, *J* = 2.4 Hz, 1H), 3.06 (dt, *J* = 17.4, 2.4 Hz, 1H), 2.86 (dt, *J* = 17.4, 2.4 Hz, 1H), 1.81 (s, 3H), 1.77 (s, 3H); ^{13}C NMR (75 MHz, $CDCl_3$) δ 169.8, 167.3, 148.3, 135.7, 135.5, 135.2, 131.7, 129.5, 129.1, 127.3, 127.1, 125.9, 124.7, 118.2, 84.3, 33.6, 10.0, 8.6. Anal. Calcd for $C_{22}H_{20}N_2O_2$: C, 76.72; H, 5.85; N, 8.13. Found: C, 76.41; H, 5.52; N, 7.94.

Synthesis and characterization of 4i. According to the synthetic procedure of **6a**, **6i** was

synthesized from **3j** (93.3 mg, 0.200 mmol). The crude product was purified by column chromatography (silica gel, hexane/EtOAc = 2/1 to $CHCl_3$/MeOH = 10/1) to give **6i** (71.1 mg, 0.189 mmol, 94%) as a white solid. R_f = 0.37 (silica gel, hexane/EtOAc = 1/1); m.p. 158–159 °C; IR (KBr) 3412 (O–H), 3296 (N–H), 1686 (C=O), 1656 (C=O) cm^{-1}; ^1H NMR (300 MHz, $CDCl_3/CD_3OD$ = 1/1) δ 7.52 (dd, *J* = 8.7, 1.2 Hz, 2H), 7.40 (dd, *J* = 8.7, 1.2 Hz, 2H), 7.35–7.27 (m, 3H), 7.26–7.18 (m, 2H), 7.12 (m, 1H), 5.65 (s, 1H), 5.23 (s, 1H), 4.64 (d, *J* = 15.3 Hz, 1H), 4.44 (d, *J* = 15.3 Hz, 1H), 3.11 (dd, *J* = 14.1, 0.9 Hz, 1H), 2.94 (d, *J* = 14.1 Hz, 1H), 1.91 (d, *J* = 1.5 Hz, 3H), 1.66 (d, *J* = 1.2 Hz, 3H); ^{13}C NMR (75 MHz, $CDCl_3/CD_3OH$ = 1/1) δ 171.2, 167.4, 151.7, 139.4, 137.8, 137.4, 128.5, 128.1, 127.6, 127.5, 126.3, 123.9, 121.8, 120.3, 91.4, 42.4, 36.9, 9.4, 7.1. Anal. Calcd for $C_{23}H_{24}N_2O_3$: C, 73.38; H, 6.43; N, 7.44. Found: C, 73.01; H, 6.27; N, 7.32.

According to the synthetic procedure of **4a**, **4i** was synthesized from **6i** (75.3 mg, 0.200 mmol). The crude product was purified by column chromatography (silica gel, hexane/EtOAc = 2/1 to 1/1) to give **4i** (71.3 mg, 0.199 mmol, 99%) as a white solid. R_f = 0.30 (silica gel, hexane/EtOAc = 1/1); m.p. 103–104 °C; IR (KBr) 1692 (C=O), 1664 (C=O) cm^{-1}; ^1H NMR (300 MHz, $CDCl_3$) δ 7.33–7.21 (m, 8H), 6.99 (d, *J* = 6.9 Hz, 2H), 6.26 (t, *J* = 2.7 Hz, 1H), 5.33 (t, *J* = 2.4 Hz, 1H), 5.02 (d, *J* = 15.6 Hz, 1H), 4.06 (d, *J* = 15.6 Hz, 1H), 2.89 (dt, *J* = 17.7, 2.7 Hz, 1H), 2.60 (dt, *J* = 17.7, 2.4 Hz, 1H), 1.76 (s, 3H); ^{13}C NMR (75 MHz, $CDCl_3$) δ 170.4, 167.4, 147.6, 137.4, 136.0, 134.8, 131.3, 128.9, 128.3, 127.8, 127.5, 127.4, 125.3, 118.5, 83.7, 42.5, 33.0, 9.9, 8.5. Anal. Calcd for $C_{23}H_{22}N_2O_2$: C, 77.07; H, 6.19; N, 7.82. Found: C, 77.05; H, 6.23; N, 7.54.

Synthesis and characterization of 4j. According to the synthetic procedure of **6a**, **6j** was synthesized from **3k** (78.5 mg, 0.200 mmol). The crude product was purified by column chromatography (silica gel, hexane/EtOAc = 2/1 to $CHCl_3$/MeOH = 10/1) to give **6j** (58.0 mg, 0.193 mmol, 96%) as a white solid. R_f = 0.22 (silica gel, hexane/EtOAc = 1/3); m.p. 167–168 °C; IR (KBr) 3370 (O–H), 3294 (N–H), 1686 (C=O), 1675 (C=O), 1656 (C=O) cm^{-1}; ^1H NMR (300 MHz, $CDCl_3/CD_3OD$ = 1/1) δ 7.50 (dd, *J* = 8.7, 1.2 Hz, 2H), 7.31 (t, *J* = 7.2 Hz, 2H), 7.11 (m, 1H), 5.67 (s, 1H), 5.42 (s, 1H), 3.05 (dd, *J* = 13.8, 0.9 Hz, 1H), 2.90 (d, *J* = 13.8 Hz, 1H), 2.88 (s, 3H),

1.93 (d, J = 0.9 Hz, 3H), 1.64 (d, J = 0.9 Hz, 3H); ^{13}C NMR (75 MHz, $CDCl_3/CD_3OH$ = 1/1) δ 171.0, 167.3, 151.4, 139.8, 137.3, 128.4, 128.0, 123.9, 121.0, 120.4, 90.3, 36.4, 23.0, 9.2, 7.1. Anal. Calcd for $C_{17}H_{20}N_2O_3$: C, 67.98; H, 6.71; N, 9.33. Found: C, 67.64; H, 6.35; N, 9.15.

According to the synthetic procedure of **4a**, **4j** was synthesized from **6j** (60.1 mg, 0.200 mmol). The crude product was purified by column chromatography (silica gel, hexane/EtOAc = 2/1 to 1/1) to give **4j** (55.3 mg, 0.196 mmol, 98%) as a white solid. R_f = 0.23 (silica gel, hexane/EtOAc = 1/2); m.p. 136–137 °C; IR (KBr) 1691 (C=O), 1666 (C=O) cm^{-1}; ^{1}H NMR (300 MHz, $CDCl_3$) δ 7.34–7.22 (m, 3H), 7.02 (d, J = 8.4 Hz, 2H), 6.30 (t, J = 2.4 Hz, 1H), 5.61 (t, J = 2.1 Hz, 1H), 3.08 (dt, J = 17.7, 2.4 Hz, 1H), 2.88 (s, 3H), 2.85 (dt, J = 17.7, 2.1 Hz, 1H), 1.79 (s, 3H), 1.72 (s, 3H); ^{13}C NMR (75 MHz, $CDCl_3$) δ 169.8, 167.2, 147.4, 136.1, 135.0, 131.7, 129.1, 127.6, 125.3, 118.5, 83.1, 32.7, 24.1, 9.9, 8.5. Anal. Calcd for $C_{17}H_{18}N_2O_2$: C, 72.32; H, 6.43; N, 9.92. Found: C, 72.14; H, 6.34; N, 9.71.

Synthesis and characterization of 4k. According to the synthetic procedure of **6a**, **6k** was synthesized from **3l** (90.5 mg, 0.202 mmol). The crude product was purified by column chromatography (silica gel, hexane/EtOAc = 2/1 to 1/1) to give **6k** (71.8 mg, 0.201 mmol, 99%) as a white solid. R_f = 0.39 (silica gel, hexane/EtOAc = 1/1); m.p. 148–149 °C; IR (KBr) 3289 (O–H), 3138 (N–H), 1693 (C=O), 1676 (C=O), 1650 (C=O) cm^{-1}; ^{1}H NMR (300 MHz, $CDCl_3/CD_3OD$ = 1/1) δ 7.50 (dd, J = 8.4, 0.9 Hz, 2H), 7.31 (t, J = 7.5 Hz, 2H), 7.12 (m, 1H), 5.67 (s, 1H), 5.38 (s, 1H), 3.45 (m, 1H), 3.13 (m, 1H), 3.09 (d, J = 13.8 Hz, 1H), 2.90 (d, J = 13.8 Hz, 1H), 1.90 (d, J = 1.2 Hz, 3H), 1.77–1.56 (m, 2H), 1.63 (d, J = 1.2 Hz, 3H), 1.40–1.27 (m, 4H), 0.91 (t, J = 6.9 Hz, 3H); ^{13}C NMR (75 MHz, $CDCl_3/CD_3OH$ = 1/1) δ 171.0, 167.3, 151.1, 139.7, 137.4, 128.6, 128.1, 123.9, 121.3, 120.3, 91.3, 39.2, 36.4, 28.8, 28.2, 21.7, 13.0, 9.2, 7.1. Anal. Calcd for $C_{21}H_{28}N_2O_3$: C, 67.98; H, 6.71; N, 9.33. Found: C, 67.64; H, 6.35; N, 9.15.

According to the synthetic procedure of **4a**, **4k** was synthesized from **6k** (47.4 mg, 0.133 mmol). The crude product was purified by column chromatography (silica gel, hexane/EtOAc = 1/1 to 1/2) to give **4k** (42.6 mg, 0.126 mmol, 95%) as a white solid. R_f = 0.30 (silica gel, hexane/EtOAc = 1/1);

IR (KBr) 1697 (C=O) cm^{-1}; ^{1}H NMR (300 MHz, $CDCl_3$) δ 7.33–7.21 (m, 3H), 7.05 (d, *J* = 7.5 Hz, 2H), 6.32 (t, *J* = 2.4 Hz, 1H), 5.61 (t, *J* = 2.1 Hz, 1H), 3.36 (m, 1H), 3.12 (m, 1H), 3.10 (dt, *J* = 17.7, 2.4 Hz, 1H), 2.89 (dt, *J* = 17.7, 2.1 Hz, 1H), 1.73 (s, 6H), 1,71–1.52 (m, 2H), 1.31–1.24 (m, 4H), 0.86 (t, *J* = 6.9 Hz, 3H); ^{13}C NMR (75 MHz, $CDCl_3$) δ 170.3, 167.3, 147.1, 136.4, 135.2, 131.7, 128.9, 127.3, 125.0, 118.3, 83.5, 39.8, 34.0, 29.3, 28.4, 22.1, 13.8, 9.9, 8.4. Anal. Calcd for $C_{21}H_{26}N_2O_2$: C, 74.53; H, 7.74; N, 8.28. Found: C, 74.13; H, 7.73; N, 8.46.

Synthesis and characterization of 4l. According to the synthetic procedure of **6a**, **6l** was synthesized from **3m** (65.7 mg, 0.140 mmol). The crude product was purified by column chromatography (silica gel, $CHCl_3$/MeOH = 40/1) to give **6l** (47.7 mg, 0.127 mmol, 91%) as a white solid. R_f = 0.42 (silica gel, hexane/EtOAc = 1/1); m.p. 200–201 °C; IR (KBr) 3327 (N–H), 3267 (O–H), 1687 (C=O), 1655 (C=O) cm^{-1}; ^{1}H NMR (300 MHz, $CDCl_3/CD_3OD$ = 1/1) δ 7.76–7.72 (m, 2H), 7.42–7.36 (m, 4H), 7.24 (m, 1H), 7.15–7.11 (m, 2H), 5.61 (s, 1H), 5.00 (s, 1H), 3.16 (d, *J* = 14.1 Hz, 1H), 2.81 (d, *J* = 14.1 Hz, 1H), 2.32 (s, 3H), 1.98 (d, *J* = 1.2 Hz, 3H), 1.73 (d, *J* = 1.2 Hz, 3H); ^{13}C NMR (75 MHz, $CDCl_3/CD_3OH$ = 1/1) δ 170.3, 167.3, 152.3, 139.0, 135.8, 134.8, 133.6, 128.6, 128.2, 127.9, 125.4, 124.7, 122.5, 120.3, 92.4, 36.9, 19.8, 9.7, 7.3. Anal. Calcd for $C_{23}H_{24}N_2O_3$: C, 73.38; H, 6.43; N, 7.44. Found: C, 73.44; H, 6.38; N, 7.63.

According to the synthetic procedure of **4a**, **4l** was synthesized from **6l** (44.5 mg, 0.118 mmol). The crude product was purified by column chromatography (silica gel, hexane/EtOAc = 2/1 to 1/1) to give **4l** (39.7 mg, 0.111 mmol, 94%) as a white solid. R_f = 0.30 (silica gel, hexane/EtOAc = 1/1); m.p. 141–142 °C; IR (KBr) 1699 (C=O), 1657 (C=O) cm^{-1}; ^{1}H NMR (300 MHz, $CDCl_3$) δ 7.39–7.11 (m, 9H), 6.04 (t, *J* = 2.4 Hz, 1H), 5.28 (t, *J* = 2.4 Hz, 1H), 3.04 (dt, *J* = 17.7, 3.0 Hz, 1H), 2.85 (dt, *J* = 17.7, 2.4 Hz, 1H), 2.33 (s, 3H), 1.80 (d, *J* = 1.2 Hz, 3H), 1.77 (d, *J* = 1.2 Hz, 3H); ^{13}C NMR (75 MHz, $CDCl_3$) δ 169.8, 167.2, 148.3, 137.1, 135.8, 135.2, 132.7, 131.5, 129.6, 129.4, 126.9, 125.8, 124.5, 117.9, 84.2, 33.4, 20.9, 10.0, 8.5. Anal. Calcd for $C_{23}H_{22}N_2O_2$: C, 78.93; H, 5.30; N, 7.36. Found: C, 78.57; H, 5.56; N, 7.72.

Synthesis and characterization of 4m. According to the synthetic procedure of **6a**, **6m** was

synthesized from **3n** (48.9 mg, 0.101 mmol). The crude product was purified by column chromatography (silica gel, hexane/EtOAc = 2/1 to $CHCl_3$/MeOH = 10/1) to give **6m** (36.1 mg, 0.0920 mmol, 92%) as a white solid. R_f = 0.30 (silica gel, hexane/EtOAc = 1/1); m.p. 196–197 °C; IR (KBr) 3386 (N–H), 3264 (O–H), 1672 (C=O), 1656 (C=O) cm^{-1}; ^{1}H NMR (300 MHz, $CDCl_3$/CD_3OD=1/1) δ 7.74 (d, *J* = 7.8 Hz, 2H), 7.42–7.35 (m, 4H), 7.23 (t, *J* = 7.2 Hz, 1H), 6.87 (d, *J* = 8.7 Hz, 2H), 5.60 (s, 1H), 4.98 (s, 1H), 3.79 (s, 3H), 3.15 (d, *J* = 14.1 Hz, 1H), 2.81 (d, *J* = 13.2 Hz, 1H), 1.98 (s, 3H), 1.73 (s, 3H); ^{13}C NMR (75 MHz, $CDCl_3$/CD_3OH = 1/1) δ 170.3, 167.3, 156.0, 152.3, 138.9, 135.9, 130.5, 128.1, 127.9, 125.4, 124.7, 122.4, 122.0, 113.3, 92.4, 54.6, 36.9, 9.7, 7.3. Anal. Calcd for $C_{23}H_{24}N_2O_4$: C, 70.39; H, 6.16; N, 7.14. Found: C, 70.30; H, 5.76; N, 7.06.

According to the synthetic procedure of **4a**, **4m** was synthesized from **6m** (36.1 mg, 0.0920 mmol). The crude product was purified by column chromatography (silica gel, hexane/EtOAc = 2/1 to 1/1) to give **4m** (34.1 mg, 0.0911 mmol, 99%) as a white solid. R_f = 0.30 (silica gel, hexane/EtOAc = 1/1); m.p. 159–160 °C; IR (KBr) 1700 (C=O), 1665 (C=O) cm^{-1}; ^{1}H NMR (300 MHz, $CDCl_3$) δ 7.40-7.21 (m, 7H), 6.89-6.84 (m, 2H), 6.06 (t, *J* = 2.7 Hz, 1H), 5.29 (t, *J* = 2.1 Hz, 1H), 3.79 (s, 3H), 3.04 (dt, *J* = 17.7, 2.7 Hz, 1H), 2.86 (d, *J* = 17.7, 2.1 Hz, 1H), 1.79 (s, 3H), 1.78 (s, 3H); ^{13}C NMR (75 MHz, $CDCl_3$) δ 169.7, 167.2, 158.3, 148.3, 135.7, 135.2, 131.4, 129.3, 127.9, 126.8, 126.1, 125.4, 117.8, 114.2, 84.2, 55.2, 33.0, 9.9, 8.4. Anal. Calcd for $C_{23}H_{22}N_2O_3$: C, 75.74; H, 5.09; N, 7.07. Found: C, 76.04; H, 5.27; N, 7.37.

Synthesis and characterization of 4n. According to the synthetic procedure of **6a**, **6n** was synthesized from **3o** (50.5 mg, 0.113 mmol). The crude product was purified by column chromatography (silica gel, $CHCl_3$/MeOH = 50/1 to 30/1) to give **6n** (39.4 mg, 0.111 mmol, 98%) as a white solid. R_f = 0.23 (silica gel, hexane/EtOAc = 1/1); m.p. 162–163 °C; IR (KBr) 3357 (N–H), 3281 (O–H), 1682 (C=O), 1652 (C=O) cm^{-1}; ^{1}H NMR (300 MHz, $CDCl_3$/CD_3OD = 1/1) δ 7.71–7.67 (m, 2H), 7.41–7.35 (m, 2H), 7.24 (m, 1H), 5.46 (s, 1H), 4.90 (s, 1H), 3.15–3.04 (m, 3H), 2.75 (d, *J* = 14.1 Hz, 1H), 1.98 (d, *J* = 1.2 Hz, 3H), 1.81 (d, *J* = 1.2 Hz, 3H), 1.54–1.44 (m, 2H), 1.40–1.24 (m, 4H), 0.92 (t, *J* = 6.9 Hz, 3H); ^{13}C NMR (75 MHz, $CDCl_3$/CD_3OH = 1/1) δ 170.2,

169.1, 152.3, 138.6, 135.9, 127.8, 127.6, 125.3, 124.6, 121.9, 92.3, 39.1, 36.8, 28.43, 28.38, 21.6, 12.9, 9.6, 7.2. Anal. Calcd for $C_{23}H_{28}N_2O_3$: C, 70.76; H, 7.92; N, 7.86. Found: C, 70.45; H, 7.73; N, 7.95.

According to the synthetic procedure of **4a**, **4n** was synthesized from **6n** (53.7 mg, 0.151 mmol). The crude product was purified by column chromatography (silica gel, hexane/EtOAc = 2/1) to give **4n** (43.9 mg, 0.130 mmol, 86%) as a white solid. R_f = 0.30 (silica gel, hexane/EtOAc = 1/1); m.p. 80–81 °C; IR (KBr) 1701 (C=O) cm^{-1}; ^1H NMR (300 MHz, $CDCl_3$) δ 7.38–7.13 (m, 5H), 6.00 (t, J = 2.7 Hz, 1H), 5.24 (t, J = 2.1 Hz, 1H), 3.32 (m, 1H), 2.86 (dt, J = 18.0, 2.7 Hz, 1H), 2.96 (m, 1H), 2.77 (dt, J = 18.0, 2.7 Hz, 1H), 1.95 (d, J = 1.2 Hz, 3H), 1.87 (d, J = 0.9 Hz, 3H), 1.67 (m, 1H), 1.44–1.20 (m, 5H), 0.87 (t, J = 7.2 Hz, 3H); ^{13}C NMR (75 MHz, $CDCl_3$) δ 170.1, 167.9, 149.1, 136.0, 135.2, 130.6, 129.3, 126.7, 125.2, 116.5, 83.6, 40.2, 32.0, 29.3, 27.4, 22.0, 13.7, 10.2, 8.7. Anal. Calcd for $C_{21}H_{26}N_2O_3$: C, 76.64; H, 6.71; N, 7.77. Found: C, 76.78; H, 6.64; N, 7.99.

Synthesis and characterization of 4o. According to the synthetic procedure of **6a**, **6o** was synthesized from **3p** (364 mg, 0.849 mmol). The crude product was purified by column chromatography (silica gel, hexane/EtOAc = 1/1 to 1/2) to give **6o** (256 mg, 0.761 mmol, 90%) as a white solid. R_f = 0.23 (silica gel, hexane/EtOAc = 1/3); m.p. 168–169 °C; IR (KBr) 3361 (N–H), 3230 (O–H), 1663 (C=O), 1649 (C=O) cm^{-1}; ^1H NMR (300 MHz, $CDCl_3/CD_3OD$ = 1/1) δ 7.69 (m, 1H), 7.60 (m, 1H), 7.51 (m, 1H), 7.41 (m, 1H), 7.22–7.18 (m, 2H), 7.07 (d, J = 8.1 Hz, 2H), 5.62 (s, 1H), 5.19 (s, 1H), 3.33 (d, J = 13.2 Hz, 1H), 3.10 (d, J = 13.5 Hz, 1H), 3.34 (s, 3H), 2.30 (s, 3H); ^{13}C NMR (75 MHz, $CDCl_3/CD_3OH$ = 1/1) δ 167.6, 167.3, 145.6, 139.1, 134.5, 133.6, 131.4, 130.7, 128.9, 128.4, 122.5, 122.3, 121.9, 120.6, 89.6, 38.1, 22.9, 19.9. Anal. Calcd for $C_{20}H_{20}N_2O_3$: C, 71.41; H, 5.99; N, 8.33. Found: C, 71.19; H, 5.90; N, 7.98.

According to the synthetic procedure of **4a**, **4o** was synthesized from **6o** (256 mg, 0.761 mmol). The crude product was purified by column chromatography (silica gel, hexane/EtOAc = 1/1 to 1/3) to give **4o** (220 mg, 0.691 mmol, 91%) as a white solid. R_f = 0.30 (silica gel, hexane/EtOAc = 1/3); m.p. 191–192 °C; IR (KBr) 1702 (C=O), 1662 (C=O) cm^{-1}; ^1H NMR (300 MHz, $CDCl_3$) δ 7.71 (d,

J = 7.5 Hz, 1H), 7.60–7.43 (m, 3H), 6.95 (d, *J* = 8.4 Hz, 2H), 6.71–6.66 (m, 2H), 6.38 (t, *J* = 2.7 Hz, 1H), 5.66 (t, *J* = 2.4 Hz, 1H), 3.39 (dt, *J* = 17.7, 2.7 Hz, 1H), 3.17 (dt, *J* = 17.7, 2.4 Hz, 1H), 3.02 (s, 3H), 2.20 (s, 3H); ^{13}C NMR (75 MHz, $CDCl_3$) δ 167.3, 166.5, 145.0, 137.7, 136.1, 132.4, 131.7, 131.4, 129.8, 129.6, 126.2, 123.4, 121.7, 118.4, 81.7, 35.8, 24.0, 20.8. Anal. Calcd for $C_{20}H_{18}N_2O_2$: C, 74.45; H, 5.70; N, 8.80. Found: C, 75.20; H, 5.87; N, 8.72.

General procedure for the synthesis of 5a–n. All the experiments for the synthesis of **5a–n** were carried out as described in the following typical procedure. The reaction of **3b** for the synthesis of **5a** was exemplified as follows.

Synthesis and characterization of 5a. To a stirred solution of **3b** (95.3 mg, 0.200 mmol) in acetonitrile (2.0 mL) were added *N*,*N*-dimethyl-4-aminopyridine (29.3 mg, 0.240 mmol) and di-*tert*-butyl dicarbonate (Boc_2O, 87.3 mg, 0.400 mmol) at room temperature. After stirring the solution at the same temperature for 2 hours, the reaction was quenched with sat. NH_4Cl aq (2.0 mL). The resulting mixture was extracted with ethyl acetate (2 × 20 mL). The combined organic extracts were washed with brine (10 mL), dried over Na_2SO_4, filtered, and concentrated in vacuo to provide a crude material. This material was purified by column chromatography (silica gel, hexane/EtOAc = 2/1) to give **7a** (106 mg, 0.184 mmol, 92%) as a white solid. R_f = 0.35 (silica gel, hexane/EtOAc = 2/1); m.p. 165–166 °C; IR (KBr) 1752 (C=O), 1712 (C=O), 1684 (C=O) cm^{-1}; ^{1}H NMR (300 MHz, $CDCl_3$) δ 8.18 (d, *J* = 7.5 Hz, 2H), 7.63 (d, *J* = 7.5 Hz, 1H), 7.54–7.42 (m, 4H), 7.32–7.24 (m, 5H), 7.14 (m, 1H), 7.01–6.96 (m, 4H), 6.63 (m, 2H), 5.31 (s, 1H), 4.68 (s, 1H), 3.89 (d, *J* = 15.0 Hz, 1H), 3.40 (d, *J* = 15.0 Hz, 1H), 1.24 (s, 9H); ^{13}C NMR (75 MHz, $CDCl_3$) δ 171.5, 167.5, 151.9, 144.6, 138.7, 138.3, 137.0, 136.1, 131.8, 131.5, 129.4, 129.1, 128.8, 128.7 128.4, 128.1, 127.5, 126.3, 125.7, 124.0, 123.9, 122.6, 83.0, 78.8, 37.6, 27.6. Anal. Calcd for $C_{35}H_{32}N_2O_4S$: C, 72.89; H, 5.59; N, 4.86. Found: C, 72.62; H, 5.67; N, 4.77.

To a stirred solution of **7a** (84.3 mg, 0.146 mmol) in tetrahydrofuran/water (9/1, 1.0 mL) was added copper (I) bromide (62.8 mg, 0.438 mmol) at room temperature. After stirring the suspension at the same temperature for 12 hours, the reaction mixture was diluted with water (1.0 mL). The resulting

solution was extracted with ethyl acetate (2 × 10 mL). The combined organic extracts were washed with brine (10 mL), dried over Na_2SO_4, filtered, and concentrated in vacuo to provide a crude material. This material was purified by column chromatography (silica gel, hexane/EtOAc = 1/1) to give **5a** (40.9 mg, 0.140 mmol, 96%) as a white solid. R_f = 0.35 (silica gel, hexane/EtOAc = 1/1); m.p. 125–126 °C; IR (KBr) 1771 (C=O), 1710 (C=O) cm^{-1}; ^{1}H NMR (300 MHz, $CDCl_3$) δ 7.93 (m, 1H), 7.71–7.59 (m, 2H), 7.53–7.34 (m, 6H), 6.21 (t, *J* = 2.7 Hz, 1H), 5.58 (t, *J* = 2.7 Hz, 1H), 3.39 (dt, *J* = 18.0, 2.7 Hz, 1H), 3.22 (dt, *J* = 18.0, 2.7 Hz, 1H); ^{13}C NMR (75 MHz, $CDCl_3$) δ 167.7, 166.4, 143.7, 133.4, 133.2, 132.8, 130.7, 130.0, 129.7, 128.8, 128.7, 124.0, 123.1, 121.6, 95.1, 35.8. Anal. Calcd for $C_{18}H_{13}NO_3$: C, 74.22; H, 4.50; N, 4.81. Found: C, 74.28; H, 4.63; N, 4.69.

Synthesis and characterization of 5b. According to the synthetic procedure of **7a**, **7b** was synthesized from **3c** (390 mg, 0.795 mmol). The crude product was purified by column chromatography (silica gel, hexane/EtOAc = 2/1) to give **7b** (466 mg, 0.789 mmol, 99%) as a white solid. R_f = 0.35 (silica gel, hexane/EtOAc = 2/1); m.p. 161–162 °C; IR (KBr) 1748 (C=O), 1708 (C=O), 1689 (C=O) cm^{-1}; ^{1}H NMR (300 MHz, $CDCl_3$) δ 7.68 (d, *J* = 8.4 Hz, 1H), 7.56–7.53 (m, 2H), 7.46 (t, *J* = 6.6 Hz, 2H), 7.31–7.22 (m, 7H), 7.13 (t, *J* = 7.5 Hz, 1H), 6.97 (t, *J* = 7.5 Hz, 2H), 6.79 (t, *J* = 8.4 Hz, 4H), 5.23 (d, *J* = 15.0 Hz, 1H), 5.22 (s, 1H), 4.79 (d, *J* = 15.0 Hz, 1H), 4.32 (s, 1H), 3.60 (d, *J* = 15.0 Hz, 1H), 2.96 (d, *J* = 15.0 Hz, 1H), 1.27 (s, 9H); ^{13}C NMR (75 MHz, $CDCl_3$) δ 172.0, 168.4, 152.0, 144.5, 138.8, 138.5, 138.4, 136.0, 131.8, 131.1, 129.4, 129.2, 128.8, 128.6, 128.3, 127.6, 127.3, 125.0, 124.9, 122.5, 82.9, 80.0, 43.8, 38.9, 27.6. Anal. Calcd for $C_{36}H_{34}N_2O_4S$: C, 73.20; H, 5.80; N, 4.74. Found: C, 72.83; H, 5.74; N, 4.68.

According to the synthetic procedure of **5a**, **5b** was synthesized from **7b** (150 mg, 0.254 mmol). The crude product was purified by column chromatography (silica gel, hexane/EtOAc = 2/1) to give **5b** (74.7 mg, 0.245 mmol, 96%) as a white solid. R_f = 0.40 (silica gel, hexane/EtOAc = 1/1); m.p. 170–172 °C; IR (KBr) 1789 (C=O), 1716 (C=O) cm^{-1}; ^{1}H NMR (300 MHz, $CDCl_3$) δ 7.89 (m, 1H), 7.66–7.56 (m, 2H), 7.44 (m, 1H), 7.33–7.25 (m, 5H), 6.42 (t, *J* = 3.0 Hz, 1H), 5.67 (t, *J* = 3.0 Hz, 1H), 5.03 (d, *J* = 15.9 Hz, 1H), 4.31 (d, *J* = 15.9 Hz, 1H), 3.27 (dt, *J* = 18.0, 2.7 Hz, 1H), 3.05 (dt, *J*

= 18.0, 2.7 Hz, 1H); ^{13}C NMR (75 MHz, $CDCl_3$) δ 167.8, 167.4, 144.1, 137.0, 133.2, 133.1, 130.7, 130.2, 128.7, 127.7, 127.5, 123.9, 121.4, 94.8, 42.2, 35.7. Anal. Calcd for $C_{19}H_{15}NO_3$: C, 74.74; H, 4.95; N, 4.59. Found: C, 74.38; H, 4.72; N, 4.49.

Synthesis and characterization of 5c. According to the synthetic procedure of **7a**, **7c** was synthesized from **3d** (41.5 mg, 0.100 mmol). The crude product was purified by column chromatography (silica gel, hexane/EtOAc = 2/1 to 1/1) to give **7c** (51.0 mg, 0.0991 mmol, 99%) as a white solid. R_f = 0.40 (silica gel, hexane/EtOAc = 1/1); m.p. 127–128 °C; IR (KBr) 1717 (C=O), 1696 (C=O) cm^{-1}; ^{1}H NMR (300 MHz, $CDCl_3$) δ 7.67 (d, J = 6.9 Hz, 1H), 7.49 (t, J = 7.5 Hz, 1H), 7.39 (d, J = 7.5 Hz, 1H), 7.33–7.23 (m, 4H), 7.12 (t, J = 7.5 Hz, 1H), 6.98 (t, J = 8.1 Hz, 2H), 6.84 (m, 4H), 5.45 (s, 1H), 5.02 (s, 1H), 3.60 (d, J = 15.3 Hz, 1H), 3.32 (d, J = 15.3 Hz, 1H), 3.22 (s, 3H), 1.33 (s, 9H); ^{13}C NMR (75 MHz, $CDCl_3$) δ 171.7, 167.5, 152.4, 144.8, 139.1, 138.4, 136.1, 131.9, 131.2, 129.4, 128.9, 128.4, 128.3, 127.6, 127.4, 123.4, 123.3, 122.5, 83.2, 78.2, 36.6, 27.7, 24.9. Anal. Calcd for $C_{30}H_{30}N_2O_4S$: C, 70.02; H, 5.88; N, 5.44. Found: C, 70.24; H, 5.84; N, 5.36.

According to the synthetic procedure of **5a**, **5c** was synthesized from **7c** (145 mg, 0.251 mmol). The crude product was purified by column chromatography (silica gel, hexane/EtOAc = 3/1 to 2/1) to give **5c** (57.4 mg, 0.250 mmol, 99%) as a white solid. R_f = 0.40 (silica gel, hexane/EtOAc = 1/2); m.p. 163–164 °C; IR (KBr) 1771 (C=O), 1709 (C=O) cm^{-1}; ^{1}H NMR (300 MHz, $CDCl_3$) δ 7.82 (m, 1H), 7.65–7.54 (m, 2H), 7.47 (m, 1H), 6.50 (t, J = 3.0 Hz, 1H), 5.90 (t, J = 3.0 Hz, 1H), 3.44 (dt, J = 18.0, 3.0 Hz, 1H), 3.28 (dt, J = 18.0, 3.0 Hz, 1H), 2.99 (s, 3H); ^{13}C NMR (75 MHz, $CDCl_3$) δ 167.8, 166.6, 143.6, 133.1, 132.9, 130.6, 130.5, 123.9, 123.5, 121.4, 94.4, 35.0, 23.5. Anal. Calcd for $C_{13}H_{11}NO_3$: C, 68.11; H, 4.84; N, 6.11. Found: C, 68.03; H, 4.85; N, 6.03.

Synthesis and characterization of 5d. According to the synthetic procedure of **7a**, **7d** was synthesized from **3e** (55.4 mg, 0.118 mmol). The crude product was purified by column chromatography (silica gel, hexane/EtOAc = 2/1) to give **7d** (65.0 mg, 0.114 mmol, 97%) as a colorless oil. R_f = 0.32 (silica gel, hexane/EtOAc = 2/1); IR (KBr) 1724 (C=O), 1702 (C=O) cm^{-1}; ^{1}H NMR (300 MHz, $CDCl_3$) δ 7.62 (d, J = 7.5 Hz, 1H), 7.45 (t, J = 7.5 Hz, 2H), 7.37–7.23 (m, 4H),

7.13 (t. *J* = 7.5 Hz, 1H), 6.98 (t, *J* = 7.5 Hz, 4H), 6.85 (dd, *J* = 8.1, 1.2 Hz, 2H), 5.55 (s, 1H), 4.99 (s, 1H), 3.76–3.63 (m, 2H), 3.63 (d, *J* = 15.9 Hz, 1H), 3.23 (d, *J* = 15.9 Hz, 1H), 1.92 (m, 1H), 1.69 (m, 1H), 1.45–1.39 (m, 4H), 1.36 (s, 9H), 0.92 (t, *J* = 6.9 Hz, 3H); ^{13}C NMR (75 MHz, $CDCl_3$) δ 172.3, 167.8, 152.6, 144.9, 140.2, 138.4, 136.0, 131.6, 131.3, 129.3, 128.9, 128.6, 128.3, 127.7, 127.6, 123.7, 122.9, 122.4, 83.3, 78.7, 40.8, 37.8, 29.8, 28.3, 27.7, 22.4, 14.0. Anal. Calcd for $C_{34}H_{38}N_2O_4S$: C, 71.55; H, 6.71; N, 4.91. Found: C, 71.84; H, 6.62; N, 4.76.

According to the synthetic procedure of **5a**, **5d** was synthesized from **7d** (115 mg, 0.201 mmol). The crude product was purified by column chromatography (silica gel, hexane/EtOAc = 2/1) to give **5d** (52.0 mg, 0.182 mmol, 91%) as a colorless oil. R_f = 0.39 (silica gel, hexane/EtOAc = 1/1); IR (KBr) 1783 (C=O), 1706 (C=O) cm^{-1}; ^{1}H NMR (300 MHz, $CDCl_3$) δ 7.82 (m, 1H), 7.64–7.53 (m, 2H), 7.44 (m, 1H), 6.50 (t, *J* = 2.7 Hz, 1H), 5.88 (t, *J* = 2.7 Hz, 1H), 3.55 (m, 1H), 3.45 (dt, *J* = 18.3, 2.7 Hz, 1H), 3.25 (m, 1H), 3.29 (dt, *J* = 18.3, 2.7 Hz, 1H), 1.76–1.63 (m, 2H), 1.37–1.30 (m, 4H), 0.90 (t, *J* = 6.9 Hz, 3H); ^{13}C NMR (75 MHz, $CDCl_3$) δ 167.9, 167.0, 143.9, 133,3, 132.9, 130.60, 130.55, 123.7, 123.5, 121.3, 95.0, 39.3, 36.0, 29.2, 28.7, 22.2, 13.9. Anal. Calcd for $C_{17}H_{19}NO_3$: C, 71.56; H, 6.71; N, 4.91. Found: C, 71.19; H, 6.73; N, 4.83.

Synthesis and characterization of 5e. According to the synthetic procedure of **7a**, **7e** was synthesized from **3f** (95.3 mg, 0.210 mmol). The crude product was purified by column chromatography (silica gel, hexane/EtOAc = 3/1) to give **7e** (111 mg, 0.200 mmol, 95%) as a colorless oil. R_f = 0.32 (silica gel, hexane/EtOAc = 2/1); m.p. 149–150 °C; IR (KBr) 1727 (C=O), 1702 (C=O) cm^{-1}; ^{1}H NMR (300 MHz, $CDCl_3$) δ 8.10 (dd, *J* = 9.0, 1.5 Hz, 2H), 7.43–7.19 (m, 11H), 6.96 (dd, *J* = 9.0, 1.5 Hz, 2H), 5.43 (s, 1H), 4.83 (s, 1H), 3.31 (d, *J* = 16.5 Hz, 1H), 3.25 (d, *J* = 16.5 Hz, 1H), 2.01 (d, *J* = 1.2 Hz, 3H), 1.45 (d, *J* = 1.2 Hz, 3H), 1.29 (s, 9H); ^{13}C NMR (75 MHz, $CDCl_3$) δ 171.5, 170.5, 152.4, 148.5, 139.0, 138.4, 137.2, 135.6, 130.2, 129.6, 129.5, 128.9, 128.5, 127.6, 127.4, 125.3, 124.1, 123.1, 83.2, 81.3, 33.6, 27.5, 11.2, 8.0. Anal. Calcd for $C_{33}H_{34}N_2O_4S$: C, 71.45; H, 6.18; N, 5.05. Found: C, 71.18; H, 6.11; N, 4.91.

According to the synthetic procedure of **5a**, **5e** was synthesized from **7e** (194 mg, 0.350 mmol). The

crude product was purified by column chromatography (silica gel, hexane/EtOAc = 2/1) to give **5e** (92.4 mg, 0.343 mmol, 98%) as a white solid. R_f = 0.26 (silica gel, hexane/EtOAc = 1/1); m.p. 138–139 °C; IR (KBr) 1766 (C=O), 1716 (C=O) cm^{-1}; ^1H NMR (300 MHz, $CDCl_3$) δ 7.43–7.25 (m, 5H), 6.17 (t, *J* = 2.7 Hz, 1H), 5.54 (t, *J* = 2.7 Hz, 1H), 3.06 (dt, *J* = 18.0, 2.7 Hz, 1H), 2.94 (dt, *J* = 18.0, 2.7 Hz, 1H), 1.93 (s, 3H), 1.92 (s, 3H); ^{13}C NMR (75 MHz, $CDCl_3$) δ 169.7, 168.0, 147.4, 133.9, 133.0, 130.2, 129.6, 128.0, 123.1, 96.7, 32.8, 9.5, 8.5. Anal. Calcd for $C_{16}H_{15}NO_3$: C, 71.36; H, 5.61; N, 5.20. Found: C, 71.21; H, 5.66; N, 5.09.

Synthesis and characterization of 5f. According to the synthetic procedure of **7a**, **7f** was synthesized from **3g** (94.6 mg, 0.203 mmol). The crude product was purified by column chromatography (silica gel, hexane/EtOAc = 4/1) to give **7f** (113 mg, 0.192 mmol, 95%) as a colorless oil. R_f = 0.30 (silica gel, hexane/EtOAc = 2/1); IR (KBr) 1749 (C=O), 1737 (C=O), 1692 (C=O) cm^{-1}; ^1H NMR (300 MHz, $CDCl_3$) δ 7.49 (d, *J* = 6.9 Hz, 2H), 7.36–7.16 (m, 9H), 7.06 (d, *J* = 8.1 Hz, 2H), 6.96 (d, *J* = 8.1 Hz, 2H), 5.22 (s, 1H), 4.95 (d, *J* = 15.0 Hz, 1H), 4.64 (d, *J* = 15.0 Hz, 1H), 4.56 (s, 1H), 3.09 (d, *J* = 16.8 Hz, 1H), 3.01 (d, *J* = 16.8 Hz, 1H), 1.94 (s, 3H), 1.48 (s, 3H), 1.33 (s, 9H); ^{13}C NMR (75 MHz, $CDCl_3$) δ 171.9, 171.7, 152.5, 148.5, 138.6, 138.5, 137.9, 135.5, 129.9, 129.5, 129.4, 129.1, 128.8, 128.3, 128.1, 127.4, 127.3, 127.0, 122.5, 82.9, 82.0, 44.3, 33.8, 27.5, 11.1, 8.0. Anal. Calcd for $C_{34}H_{36}N_2O_4S$: C, 71.80; H, 6.38; N, 4.93. Found: C, 71.45; H, 6.29; N, 4.80.

According to the synthetic procedure of **5a**, **5f** was synthesized from **7f** (128 mg, 0.225 mmol). The crude product was purified by column chromatography (silica gel, hexane/EtOAc = 5/1 to 2/1) to give **5f** (61.0 mg, 0.215 mmol, 96%) as a white solid. R_f = 0.32 (silica gel, hexane/EtOAc = 1/1); IR (KBr) 1773 (C=O), 1709 (C=O) cm^{-1}; ^1H NMR (300 MHz, $CDCl_3$) δ 7.31–7.21 (m, 5H), 6.31 (t, *J* = 2.7 Hz, 1H), 5.60 (t, *J* = 2.7 Hz, 1H), 4.76 (d, *J* = 16.2 Hz, 1H), 4.25 (d, *J* = 16.2 Hz, 1H), 2.94 (dt, *J* = 18.6, 2.7 Hz, 1H), 2.78 (dt, *J* = 18.6, 2.7 Hz, 1H), 1.88 (d, *J* = 0.9 Hz, 3H), 1.84 (d, *J* = 0.9 Hz, 3H); ^{13}C NMR (75 MHz, $CDCl_3$) δ 170.7, 168.0, 147.2, 137.3, 133.1, 130.3, 128.6, 127.6, 123.7, 123.6, 96.2, 42.3, 32.6, 9.3, 8.6. Anal. Calcd for $C_{17}H_{17}NO_3$: C, 72.07; H, 6.05; N, 4.94. Found: C,

71.75; H, 6.14; N, 4.87.

Synthesis and characterization of 5g. According to the synthetic procedure of **7a**, **7g** was synthesized from **3h** (78.5 mg, 0.200 mmol). The crude product was purified by column chromatography (silica gel, hexane/EtOAc = 3/1 to 2/1) to give **7g** (94.5 mg, 0.192 mmol, 96%) as a colorless oil. R_f = 0.40 (silica gel, hexane/EtOAc = 1/1); m.p. 105–106 °C; IR (KBr) 1731 (O–H), 1691 (C=O) cm^{-1}; ^{1}H NMR (300 MHz, $CDCl_3$) δ 7.37 (t, *J* = 6.9 Hz, 2H), 7.29 (t, *J* = 6.9 Hz, 2H), 7.22 (t, *J* = 8.1 Hz, 2H), 7.09 (m, 4H), 5.53 (s, 1H), 5.17 (s, 1H), 3.15 (d, *J* = 16.2 Hz, 1H), 3.08 (s, 3H), 3.04 (d, *J* = 16.2 Hz, 1H), 1.97 (s, 3H), 1.41 (s, 3H), 1.37 (s, 9H); ^{13}C NMR (75 MHz, $CDCl_3$) δ 171.8, 170.9, 152.6, 147.7, 139.6, 138.5, 135.6, 130.6, 129.5, 129.2, 129.0, 128.5, 127.7, 127.6, 121.3, 83.3, 80.7, 33.7, 27.7, 25.1, 11.0, 8.1. Anal. Calcd for $C_{28}H_{32}N_2O_4S$: C, 68.27; H, 6.55; N, 5.69. Found: C, 67.92; H, 6.27; N, 5.46.

According to the synthetic procedure of **5a**, **5g** was synthesized from **7g** (81.7 mg, 0.166 mmol). The crude product was purified by column chromatography (silica gel, hexane/EtOAc = 2/1 to 1/2) to give **5g** (34.1 mg, 0.165 mmol, 99%) as a white solid. R_f = 0.35 (silica gel, hexane/EtOAc = 1/2); IR (KBr) 1772 (C=O), 1706 (C=O) cm^{-1}; ^{1}H NMR (300 MHz, $CDCl_3$) δ 6.41 (t, *J* = 2.7 Hz, 1H), 5.83 (t, *J* = 2.7 Hz, 1H), 3.11 (dt, *J* = 18.0, 2.7 Hz, 1H), 2.99 (dt, *J* = 18.0, 2.7 Hz, 1H), 2.84 (s, 3H), 1.88 (d, *J* = 0.9 Hz, 3H), 1.84 (d, *J* = 0.9 Hz, 3H); ^{13}C NMR (75 MHz, $CDCl_3$) δ 169.9, 168.0, 146.5, 133.1, 130.5, 123.7, 95.8, 31.9, 23.4, 9.1, 8.4. Anal. Calcd for $C_{11}H_{13}NO_3$: C, 63.76; H, 6.32; N, 6.76. Found: C, 63.37; H, 6.06; N, 6.95.

Synthesis and characterization of 5h. According to the synthetic procedure of **7a**, **7h** was synthesized from **3i** (88.8 mg, 0.198 mmol). The crude product was purified by column chromatography (silica gel, hexane/EtOAc = 4/1 to 2/1) to give **7h** (108 mg, 0.197 mmol, 99%) as a white solid. R_f = 0.32 (silica gel, hexane/EtOAc = 2/1); IR (KBr) 1739 (C=O), 1690 (C=O), 1684 (C=O) cm^{-1}; ^{1}H NMR (300 MHz, $CDCl_3$) δ 7.39 (t, *J* = 7.2 Hz, 2H), 7.31 (t, *J* = 7.2 Hz, 2H), 7.23 (t, *J* = 7.2 Hz, 2H), 7.15–7.06 (m, 4H), 5.49 (s, 1H), 3.58–3.45 (m, 2H), 3.18 (d, *J* = 17.4 Hz, 1H), 3.05 (d, *J* = 17.4 Hz, 1H), 1.94 (d, *J* = 0.9 Hz, 3H), 1.74 (m, 1H), 1.60 (m, 1H), 1.43 (d, *J* = 0.9 Hz, 3H),

1.38 (s, 9H), 1.36–1.31 (m, 4H), 0.89 (t, *J* = 7.2 Hz, 3H); ^{13}C NMR (75 MHz, $CDCl_3$) δ 172.5, 171.2, 152.8, 148.1, 140.5, 138.3, 135.8, 129.7, 129.5, 129.2, 128.9, 128.3, 127.6, 127.4, 119.0, 83.2, 81.2, 40.8, 32.9, 29.6, 28.1, 27.5, 22.2, 13.9, 10.7, 7.8. Anal. Calcd for $C_{32}H_{40}N_2O_4S$: C, 70.04; H, 7.35; N, 5.11. Found: C, 70.14; H, 7.07; N, 4.94.

According to the synthetic procedure of **5a**, **5h** was synthesized from **7h** (100 mg, 0.182 mmol). The crude product was purified by column chromatography (silica gel, hexane/EtOAc = 4/1 to 2/1) to give **5h** (44.2 mg, 0.176 mmol, 97%) as a white solid. R_f = 0.30 (silica gel, hexane/EtOAc = 2/1); IR (KBr) 1693 (C=O) cm^{-1}; ^{1}H NMR (300 MHz, $CDCl_3$) δ 6.41 (t, *J* = 2.7 Hz, 1H), 5.82 (t, *J* = 2.7 Hz, 1H), 3.44 (m, 1H), 3.12 (dt, *J* = 18.3, 2.7 Hz, 1H), 3.06 (m, 1H), 3.01 (dt, *J* = 18.3, 2.7 Hz, 1H), 1.86 (d, *J* = 1.2 Hz, 3H), 1.83 (d, *J* = 1.2 Hz, 3H), 1.62–1.53 (m, 2H), 1.34–1.22 (m, 4H), 0.88 (t, *J* = 6.9 Hz, 3H); ^{13}C NMR (75 MHz, $CDCl_3$) δ 170.3, 168.0, 146.5, 133.3, 130.3, 123.4, 96.3, 39.1, 32.6, 29.0, 28.6, 22.1, 13.8, 9.1, 8.3. Anal. Calcd for $C_{15}H_{21}NO_3$: C, 68.42; H, 8.04; N, 5.32. Found: C, 68.75; H, 8.37; N, 5.59.

Cytotoxicity Test. P388 cells were seeded in 96-well plates (Iwaki) and cultured overnight in RPMI1640 medium (Sigma) supplemented with 10% fetal bovine serum (FBS) (Cellgro), and antibiotics (100 U/mL penicillin and 100 μg/mL streptomycin, Nakarai Tesque) at 37 °C in 5% CO_2-humidified incubator. Then 0.1 μg/mL, 1 μg/mL, 10 μg/mL and 100 μg/mL of samples were added and incubated for 4 days under the same condition. The cell proliferation was measured by using the cell counting kit-8 (Dojin Kagaku).

References

1. Sengoku, T.; Hayashi, D.; Takahashi, M.; Yoda, H. *Eur. J. Org. Chem.* **2018**, 1813–1820.

2. Ukhin, L. Y.; Suponitsky, K. Y.; Belousova, L. V.; Orlova, Z. I. *Russ. Chem. Bull.* **2009**, *58*, 2478–2487.

3. Kobayashi, K.; Hashimoto, H.; Suzuki, T.; Konishi, H. *Helv. Chim. Acta* **2011**, *94*, 2002–2009.

4. Li, X.; Sun, B.; Zhou, J.; Jin, C.; Yu, C. *Eur. J. Org. Chem.* **2019**, 2635–2638.
