# Supplementary material for: Bifurcated synthesis of methylene-lactone- and methylene-lactam-fused spirolactams via electrophilic amide allylation of γ-phenylthio-functionalized γ-lactams
Source: Beilstein J Org Chem. 2020 Nov 13;16:2769–75. doi: 10.3762/bjoc.16.227 (PMC7670114; doi:10.3762/bjoc.16.227)
Supplement: File 2 — Copies of 1H and 13C NMR spectra of all new compounds. [file Beilstein_J_Org_Chem-16-2769-s002.pdf]

BEILSTEIN JOURNAL OF ORGANIC CHEMISTRY

Supporting Information

for

Bifurcated synthesis of methylene-lactone- and methylene-lactam-fused spirolactams via electrophilic amide allylation of γ-phenylthio-functionalized γ-lactams

Tetsuya Sengoku, Koki Makino, Ayumi Iijima, Toshiyasu Inuzuka and Hidemi Yoda

Beilstein J. Org. Chem. **2020,** *16,* 2769–2775. doi:10.3762/bjoc.16.227

Copies of ^{1}H and ^{13}C NMR spectra of all new compounds

License and Terms: This is a supporting information file under the terms of the Creative Commons Attribution License (https://creativecommons.org/licenses/by/4.0). Please note that the reuse, redistribution and reproduction in particular requires that the authors and source are credited.
The license is subject to the *Beilstein Journal of Organic Chemistry* terms and conditions: (https://www.beilstein-journals.org/bjoc)

The following data are included in this material:

Copies of ^{1}H and ^{13}C NMR spectra S2–S141

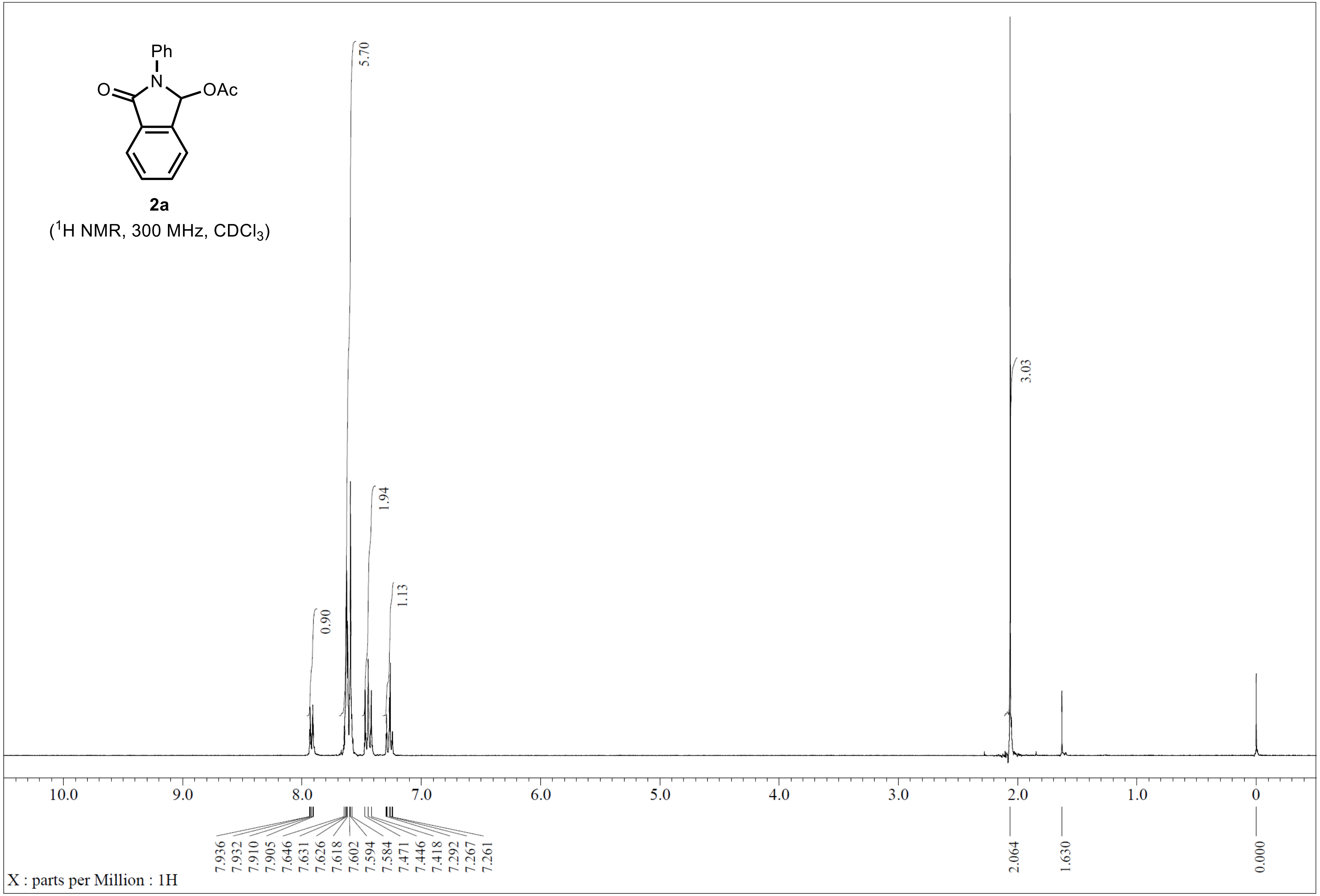

Ph
N
O
OAc
2a
(1H NMR, 300 MHz, CDCl3)
5.70
3.03
1.94
1.13
0.90
10.0
9.0
8.0
7.0
6.0
5.0
4.0
3.0
2.0
1.0
0
7.936
7.932
7.910
7.905
7.646
7.631
7.626
7.618
7.602
7.594
7.584
7.471
7.446
7.418
7.292
7.267
7.261
2.064
1.630
0.000
X : parts per Million : 1H

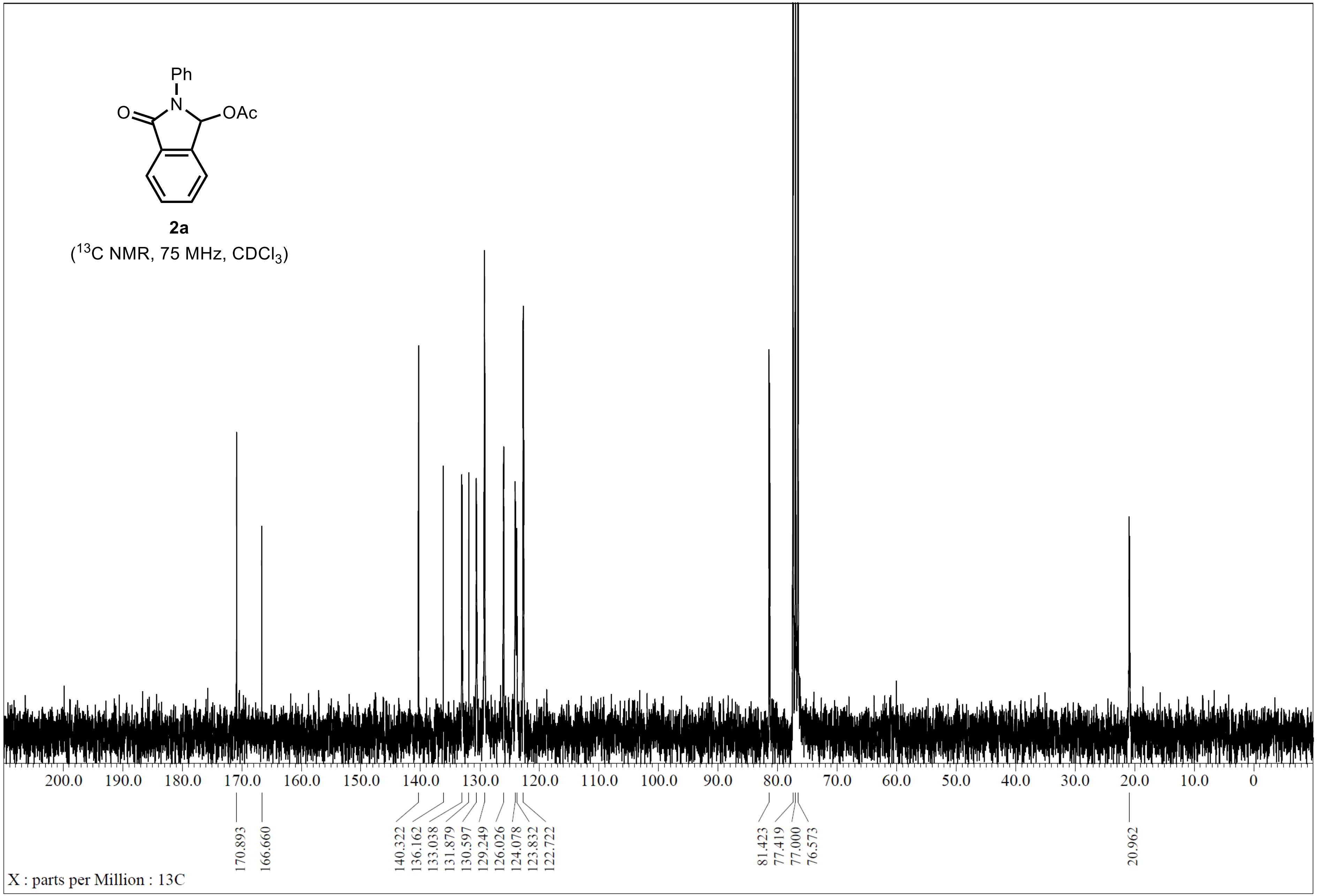
Ph
N
O
OAc
2a
(¹³C NMR, 75 MHz, CDCl3)
200.0
190.0
180.0
170.0
160.0
150.0
140.0
130.0
120.0
110.0
100.0
90.0
80.0
70.0
60.0
50.0
40.0
30.0
20.0
10.0
0
170.893
166.660
140.322
136.162
133.038
131.879
130.597
129.249
126.026
124.078
123.832
122.722
81.423
77.419
77.000
76.573
20.962
X : parts per Million : 13C

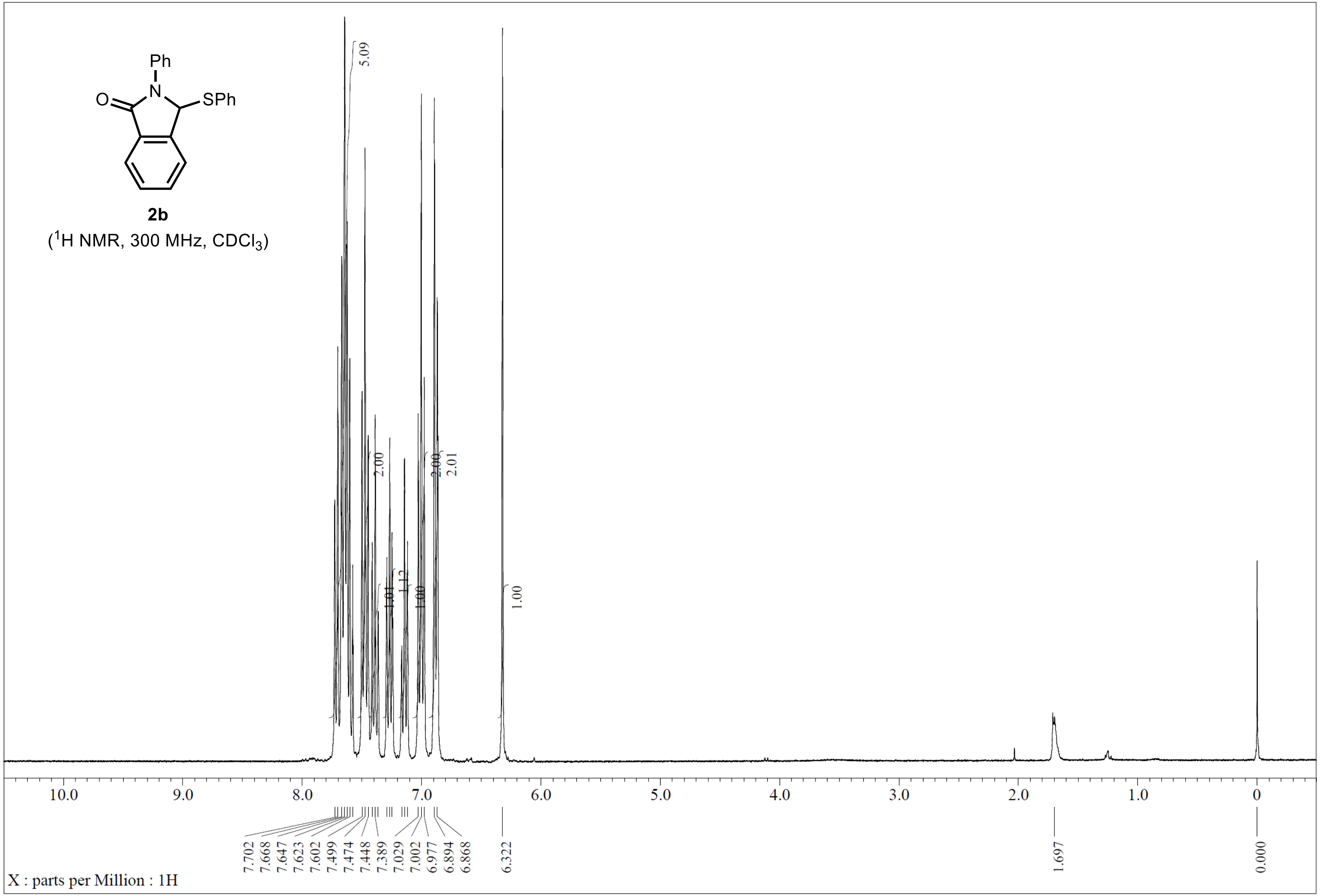
Ph
N
O
SPh
2b
(^{1}H NMR, 300 MHz, CDCl$_3$)
5.09
2.00
2.00
2.01
1.01
1.12
1.00
1.00
10.0
9.0
8.0
7.0
6.0
5.0
4.0
3.0
2.0
1.0
0
7.702
7.668
7.647
7.623
7.602
7.499
7.474
7.448
7.389
7.029
7.002
6.977
6.894
6.868
6.322
1.697
0.000
X : parts per Million : 1H

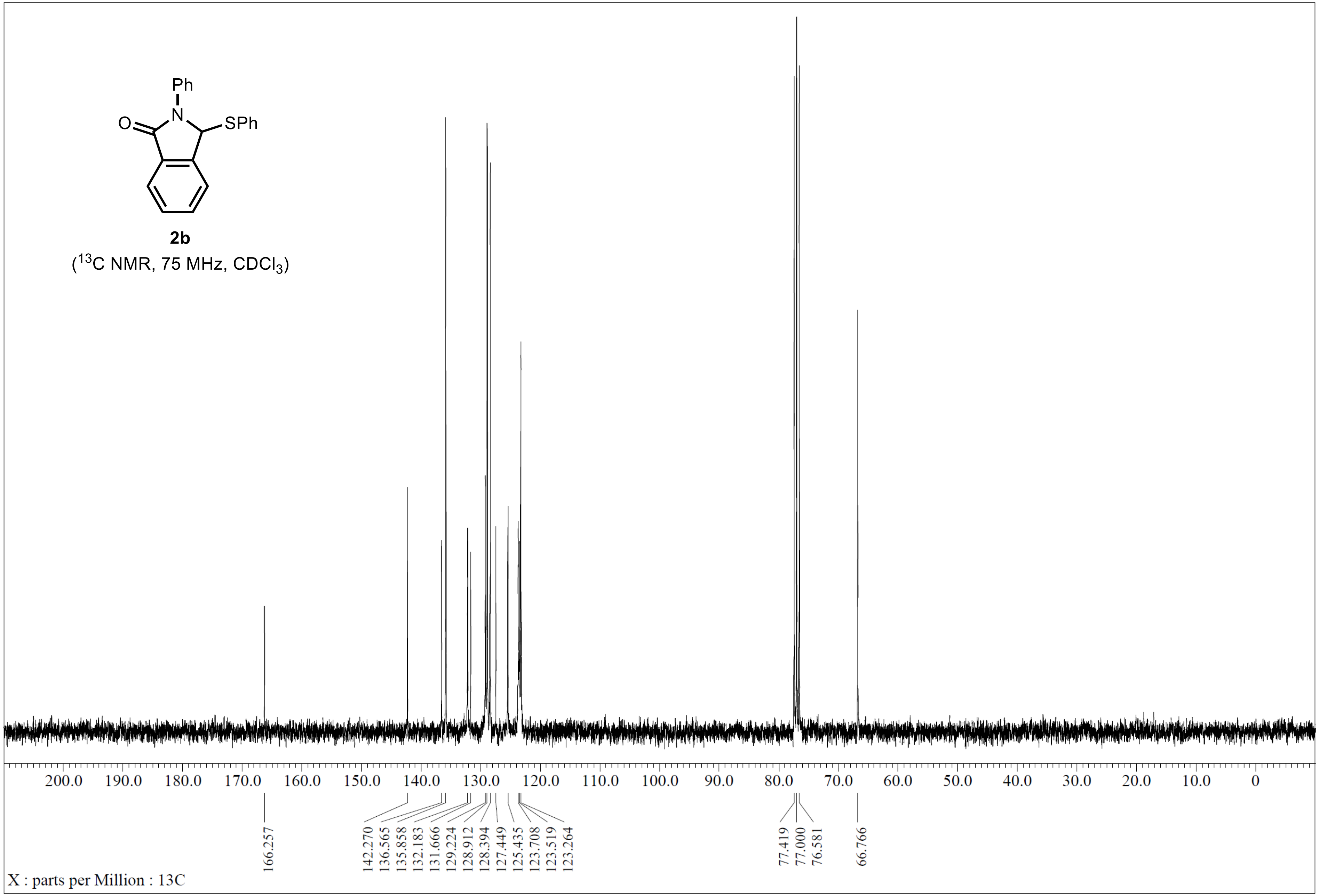
Ph
N
O
SPh
2b
(^{13}C NMR, 75 MHz, CDCl$_3$)
200.0 190.0 180.0 170.0 160.0 150.0 140.0 130.0 120.0 110.0 100.0 90.0 80.0 70.0 60.0 50.0 40.0 30.0 20.0 10.0 0
166.257
142.270
136.565
135.858
132.183
131.666
129.224
128.912
128.394
127.449
125.435
123.708
123.519
123.264
77.419
77.000
76.581
66.766
X : parts per Million : 13C

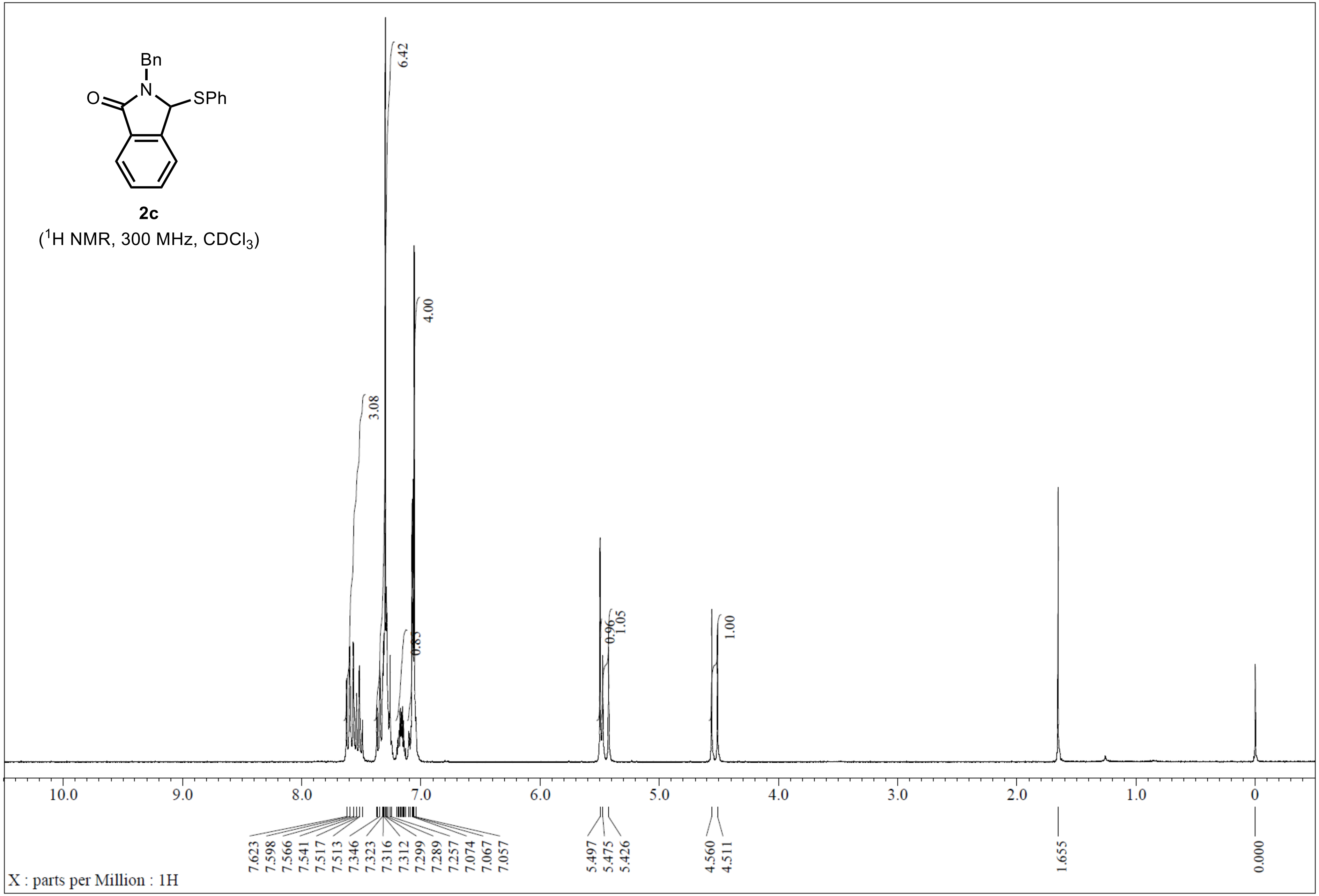
Bn
O
N
SPh
2c
(1H NMR, 300 MHz, CDCl3)
6.42
4.00
3.08
0.83
0.96
1.05
1.00
10.0
9.0
8.0
7.0
6.0
5.0
4.0
3.0
2.0
1.0
0
7.623
7.598
7.566
7.541
7.517
7.513
7.346
7.323
7.316
7.312
7.299
7.289
7.257
7.074
7.067
7.057
5.497
5.475
5.426
4.560
4.511
1.655
0.000
X : parts per Million : 1H

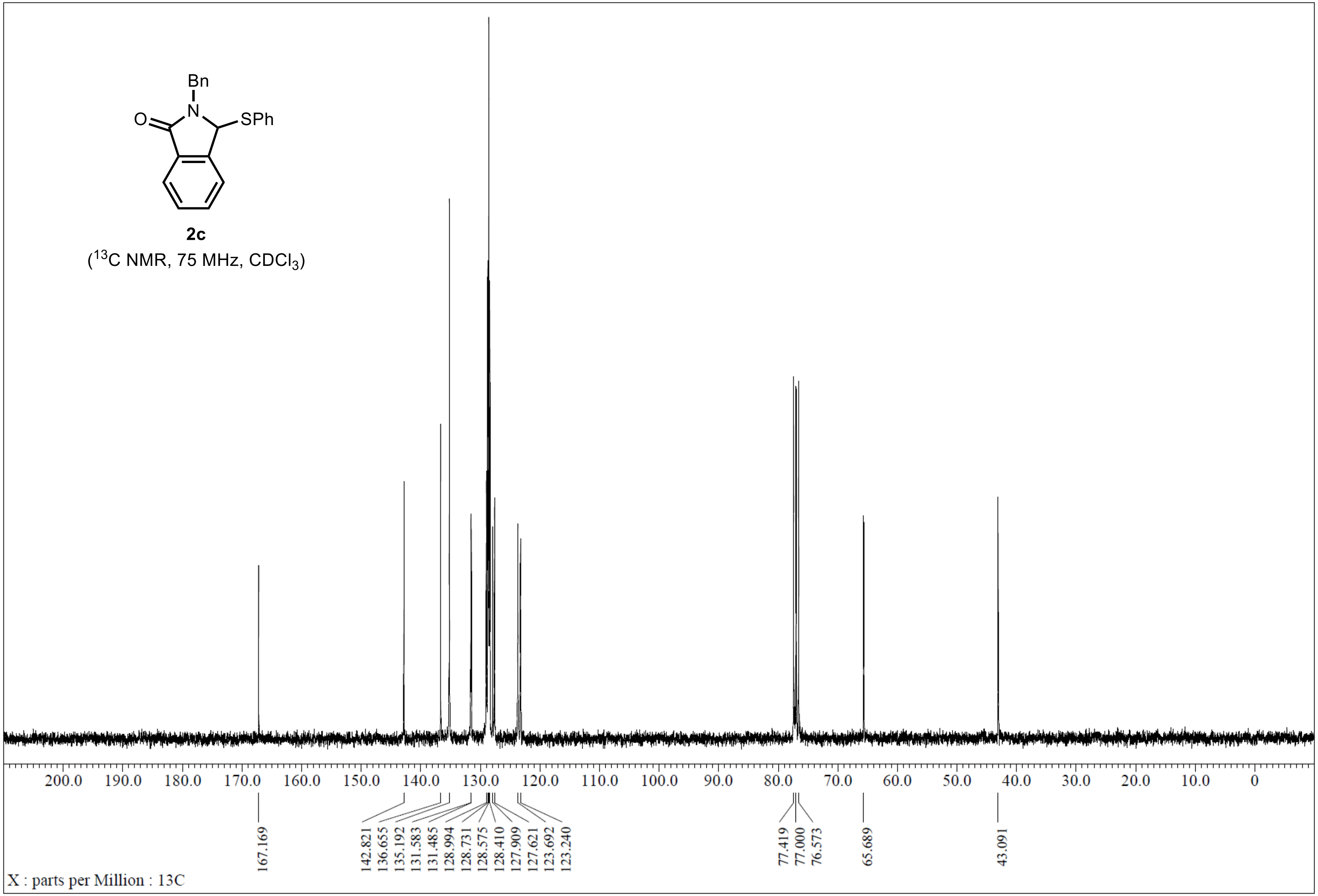
Bn
N
O
SPh
2c
(13C NMR, 75 MHz, CDCl3)
200.0 190.0 180.0 170.0 160.0 150.0 140.0 130.0 120.0 110.0 100.0 90.0 80.0 70.0 60.0 50.0 40.0 30.0 20.0 10.0 0
167.169
142.821
136.655
135.192
131.583
131.485
128.994
128.731
128.575
128.410
127.909
127.621
123.692
123.240
77.419
77.000
76.573
65.689
43.091
X : parts per Million : 13C

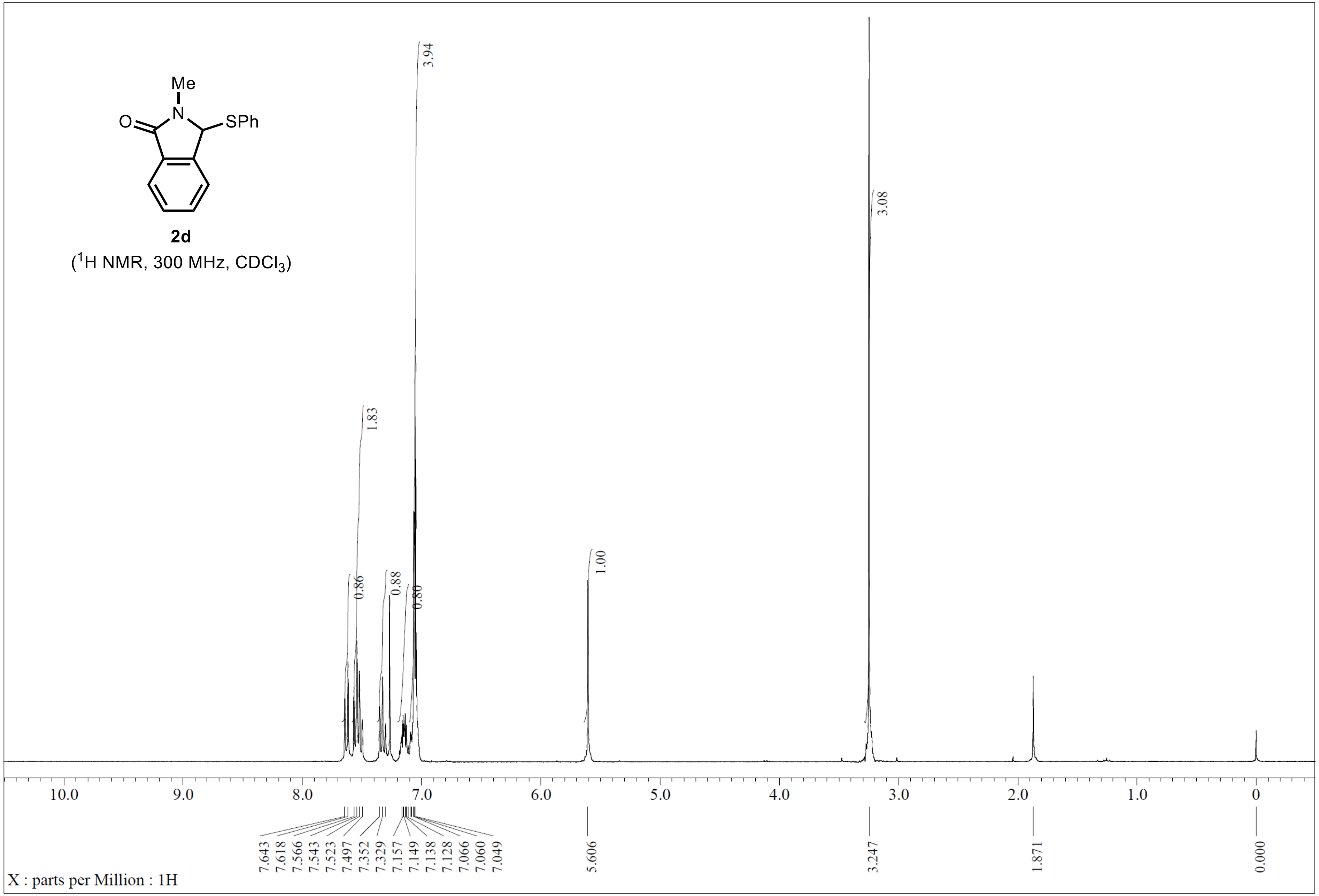
Me
N
O
SPh
2d
(1H NMR, 300 MHz, CDCl3)
3.94
1.83
0.86
0.88
0.80
1.00
3.08
10.0
9.0
8.0
7.0
6.0
5.0
4.0
3.0
2.0
1.0
0
7.643
7.618
7.566
7.543
7.523
7.497
7.352
7.329
7.157
7.149
7.138
7.128
7.066
7.060
7.049
5.606
3.247
1.871
0.000
X : parts per Million : 1H

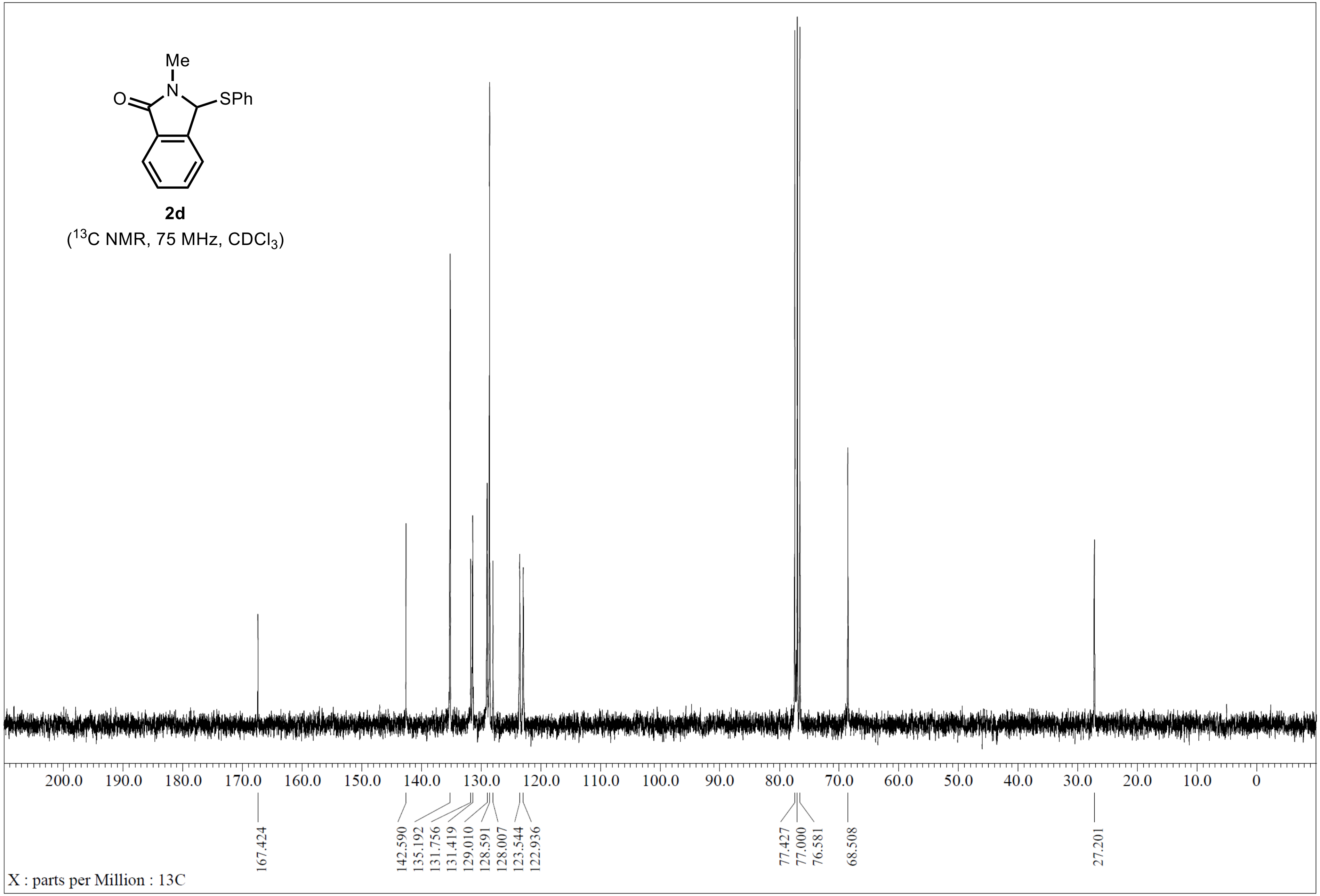
Me
O
N
SPh
2d
(^{13}C NMR, 75 MHz, CDCl$_3$)
200.0 190.0 180.0 170.0 160.0 150.0 140.0 130.0 120.0 110.0 100.0 90.0 80.0 70.0 60.0 50.0 40.0 30.0 20.0 10.0 0
167.424
142.590
135.192
131.756
131.419
129.010
128.591
128.007
123.544
122.936
77.427
77.000
76.581
68.508
27.201
X : parts per Million : 13C

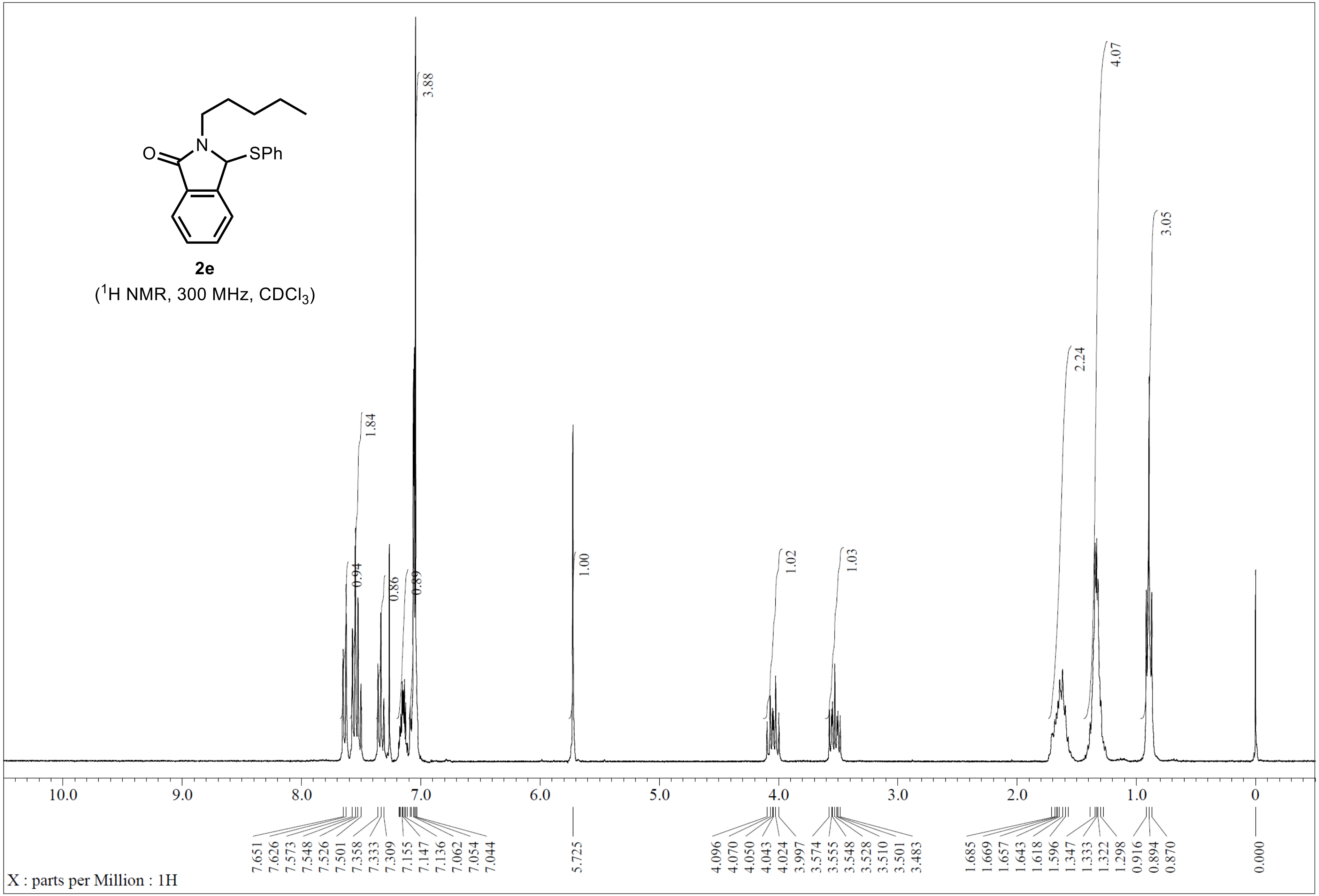
O
N
SPh
2e
(¹H NMR, 300 MHz, CDCl3)
3.88
4.07
3.05
2.24
1.84
0.94
0.86
0.89
1.00
1.02
1.03
10.0
9.0
8.0
7.0
6.0
5.0
4.0
3.0
2.0
1.0
0
7.651
7.626
7.573
7.548
7.526
7.501
7.358
7.333
7.309
7.155
7.147
7.136
7.062
7.054
7.044
5.725
4.096
4.070
4.050
4.043
4.024
3.997
3.574
3.555
3.548
3.528
3.510
3.501
3.483
1.685
1.669
1.657
1.643
1.618
1.596
1.347
1.333
1.322
1.298
0.916
0.894
0.870
0.000
X : parts per Million : 1H

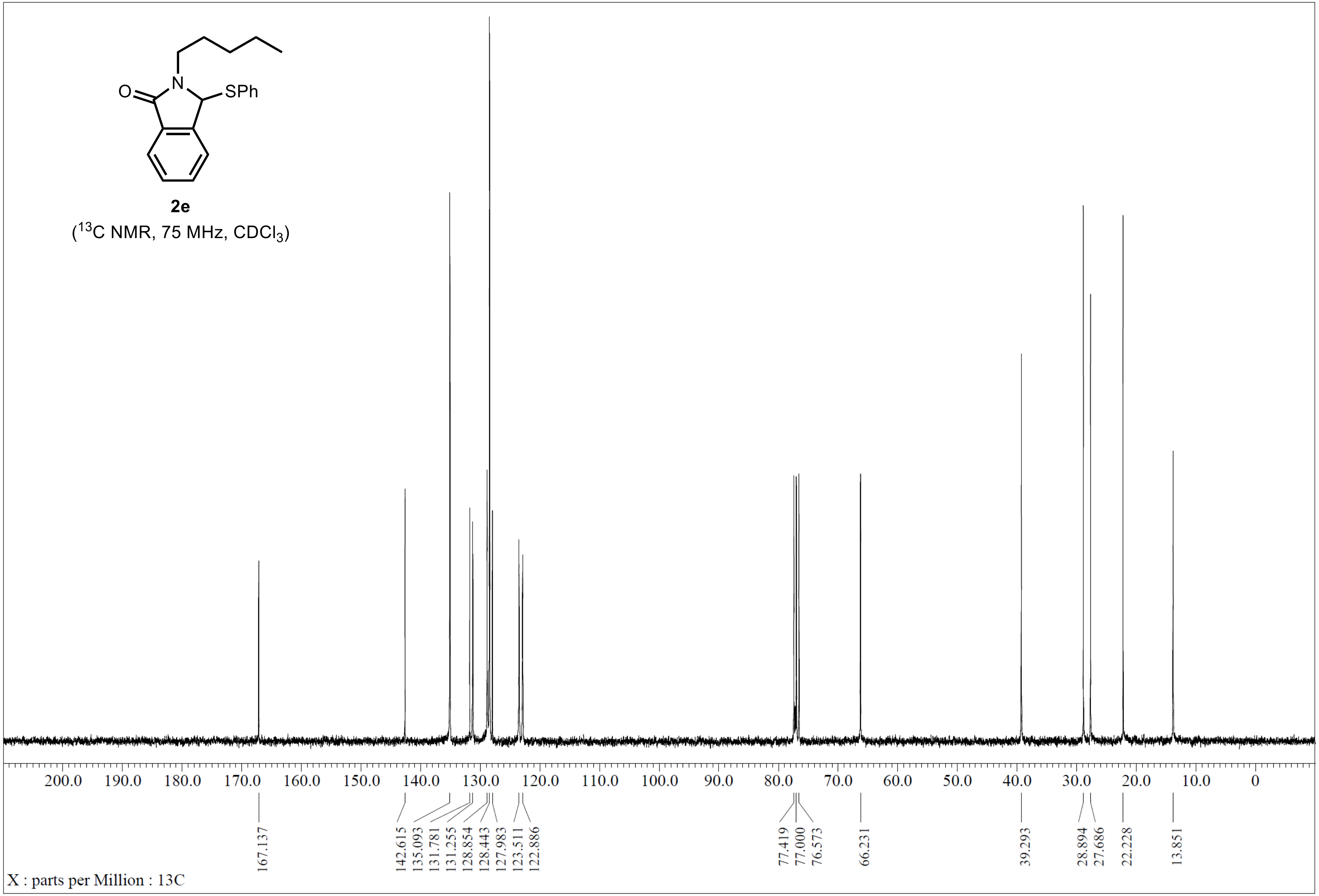

O
N
SPh
2e
(13C NMR, 75 MHz, CDCl3)
200.0 190.0 180.0 170.0 160.0 150.0 140.0 130.0 120.0 110.0 100.0 90.0 80.0 70.0 60.0 50.0 40.0 30.0 20.0 10.0 0
167.137
142.615
135.093
131.781
131.255
128.854
128.443
127.983
123.511
122.886
77.419
77.000
76.573
66.231
39.293
28.894
27.686
22.228
13.851
X : parts per Million : 13C

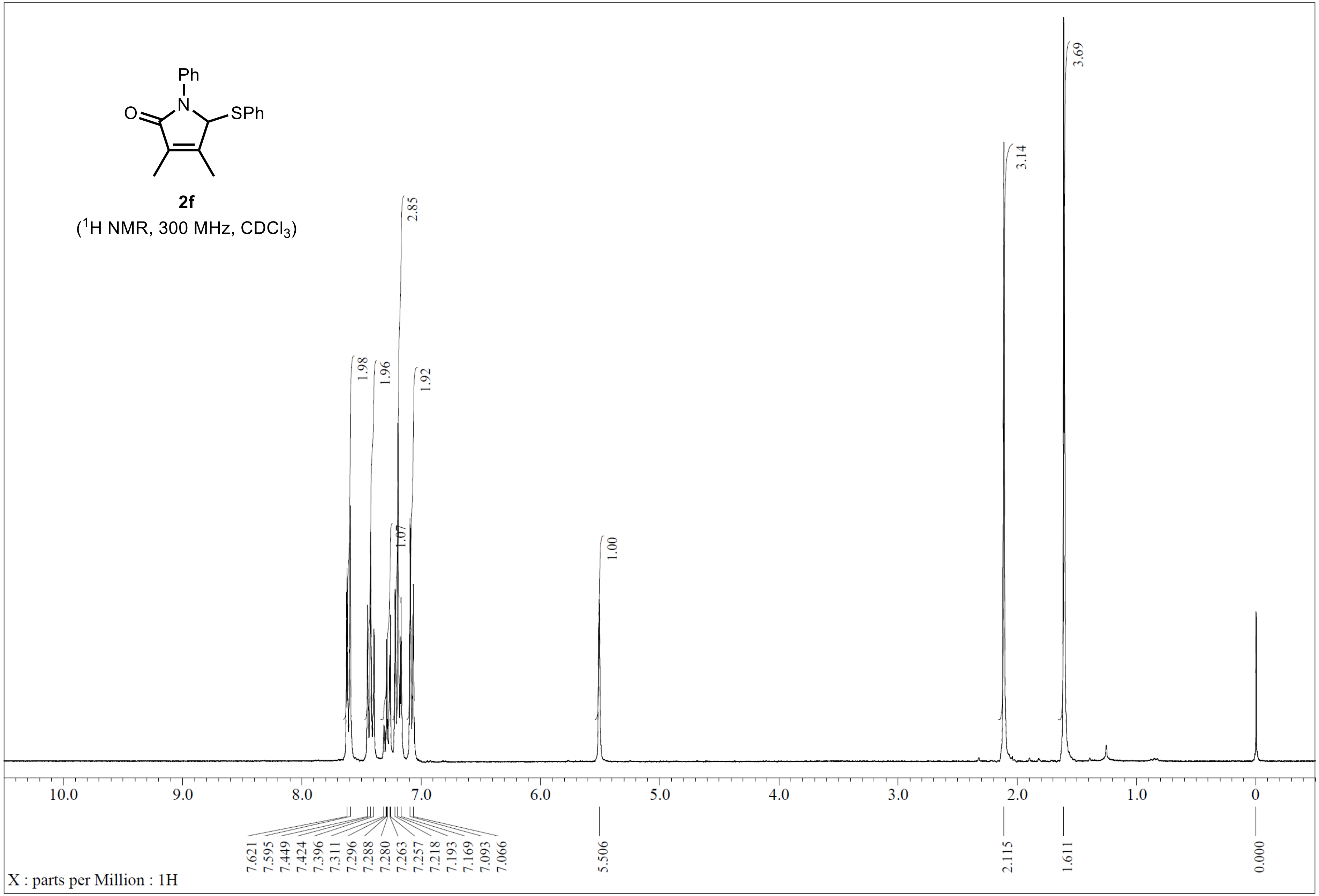
Ph
N
O
SPh
2f
(1H NMR, 300 MHz, CDCl3)
3.69
3.14
2.85
1.98
1.96
1.92
1.07
1.00
10.0
9.0
8.0
7.0
6.0
5.0
4.0
3.0
2.0
1.0
0
7.621
7.595
7.449
7.424
7.396
7.311
7.296
7.288
7.280
7.263
7.257
7.218
7.193
7.169
7.093
7.066
5.506
2.115
1.611
0.000
X : parts per Million : 1H

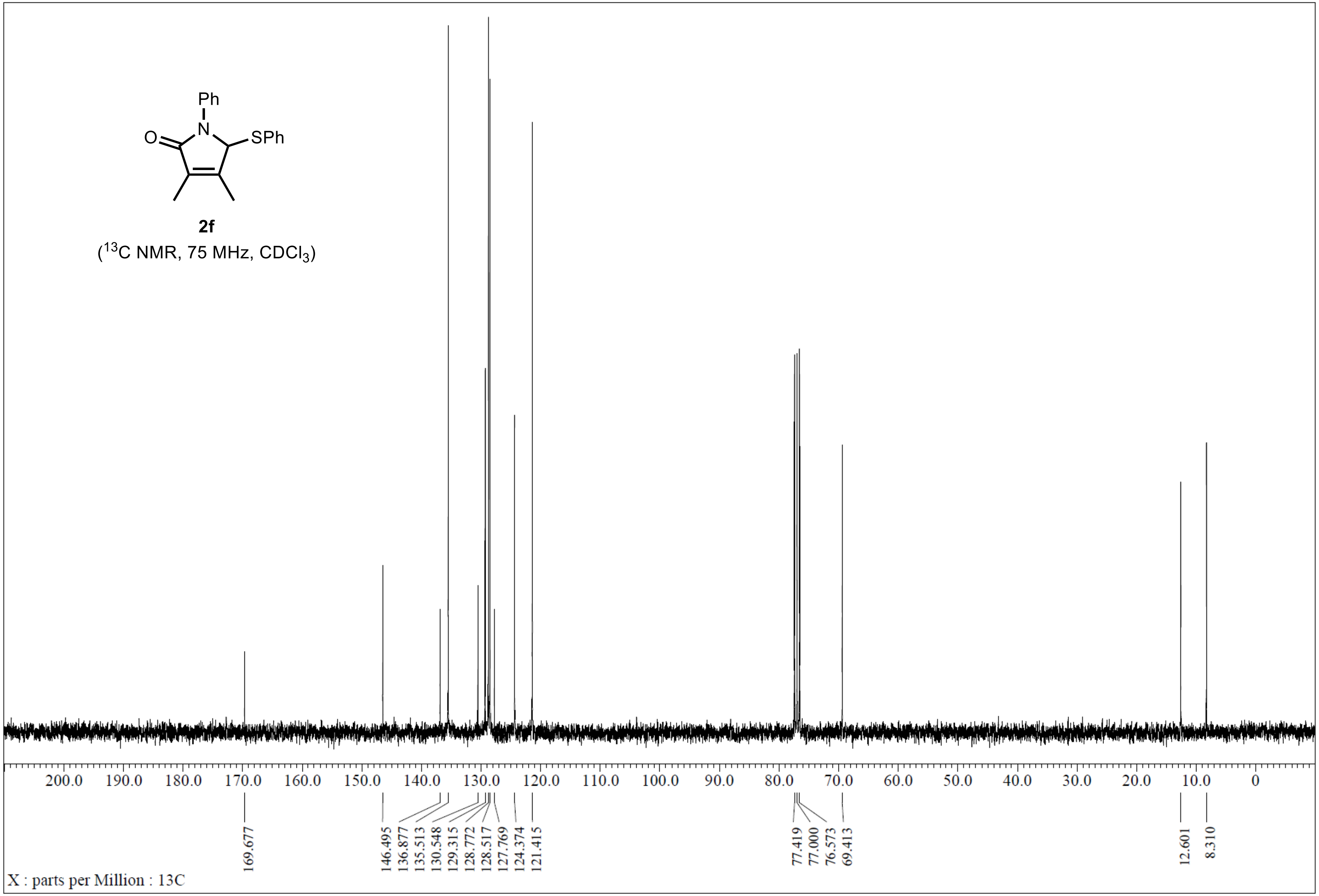
Ph
O
N
SPh
2f
(13C NMR, 75 MHz, CDCl3)
200.0 190.0 180.0 170.0 160.0 150.0 140.0 130.0 120.0 110.0 100.0 90.0 80.0 70.0 60.0 50.0 40.0 30.0 20.0 10.0 0
169.677
146.495
136.877
135.513
130.548
129.315
128.772
128.517
127.769
124.374
121.415
77.419
77.000
76.573
69.413
12.601
8.310
X : parts per Million : 13C

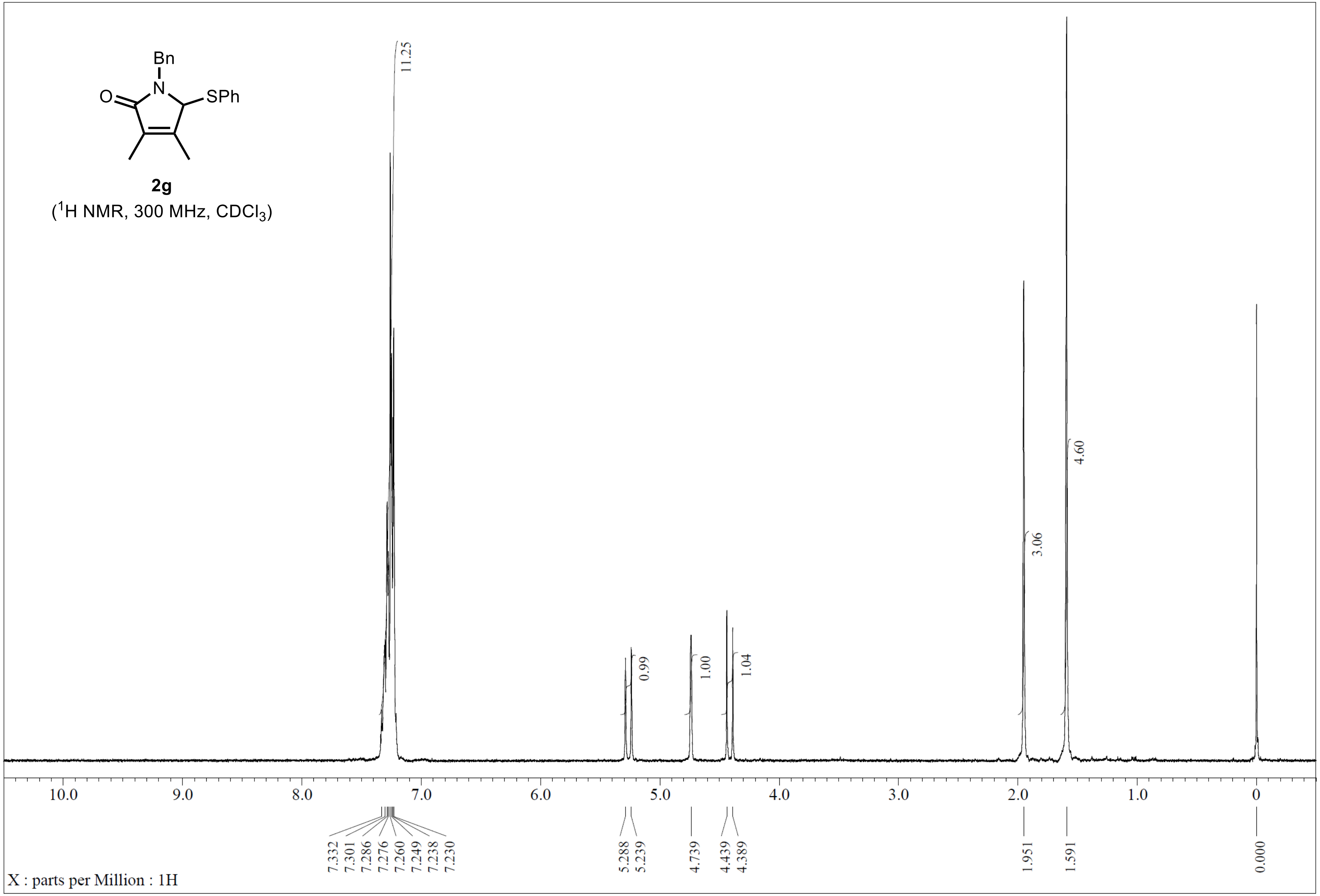
Bn
N
O
SPh
2g
(1H NMR, 300 MHz, CDCl3)
11.25
4.60
3.06
0.99
1.00
1.04
10.0
9.0
8.0
7.0
6.0
5.0
4.0
3.0
2.0
1.0
0
7.332
7.301
7.286
7.276
7.260
7.249
7.238
7.230
5.288
5.239
4.739
4.439
4.389
1.951
1.591
0.000
X : parts per Million : 1H

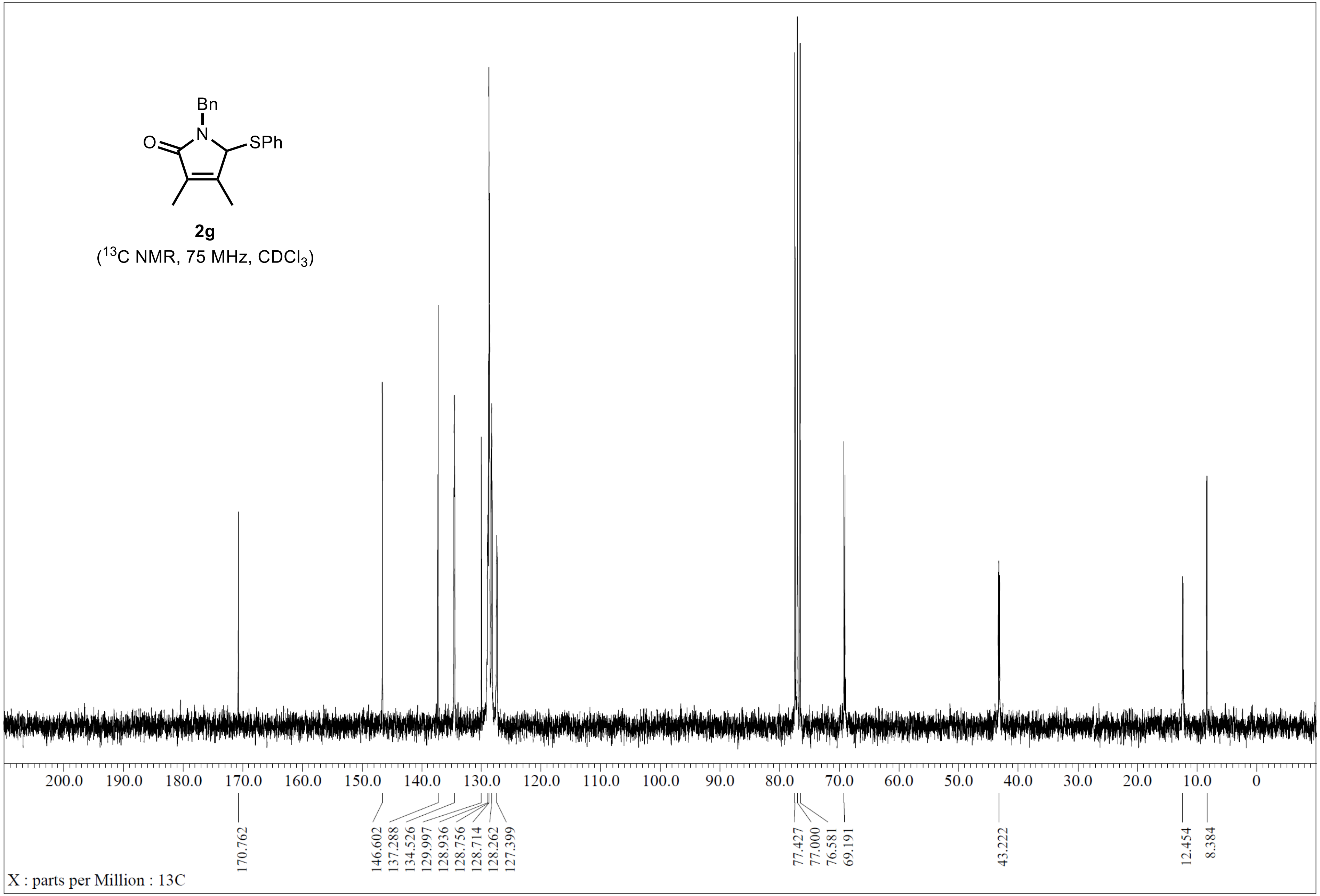
Bn
N
O
SPh
2g
(13C NMR, 75 MHz, CDCl3)
200.0
190.0
180.0
170.0
160.0
150.0
140.0
130.0
120.0
110.0
100.0
90.0
80.0
70.0
60.0
50.0
40.0
30.0
20.0
10.0
0
170.762
146.602
137.288
134.526
129.997
128.936
128.756
128.714
128.262
127.399
77.427
77.000
76.581
69.191
43.222
12.454
8.384
X : parts per Million : 13C

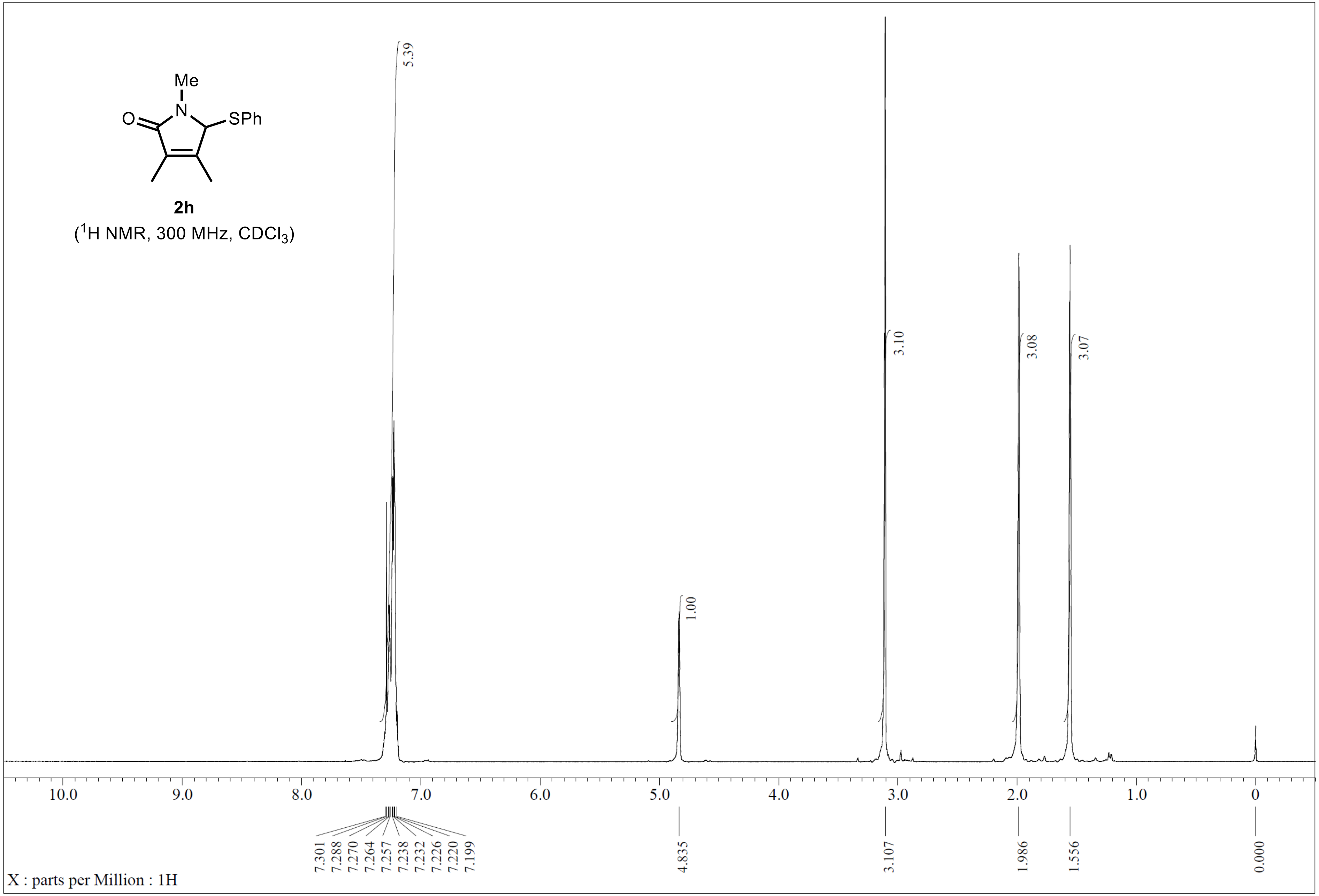
Me
N
O
SPh
2h
(¹H NMR, 300 MHz, CDCl₃)
5.39
3.10
3.08
3.07
1.00
10.0 9.0 8.0 7.0 6.0 5.0 4.0 3.0 2.0 1.0 0
7.301 7.288 7.270 7.264 7.257 7.238 7.232 7.226 7.220 7.199
4.835
3.107
1.986
1.556
0.000
X : parts per Million : 1H

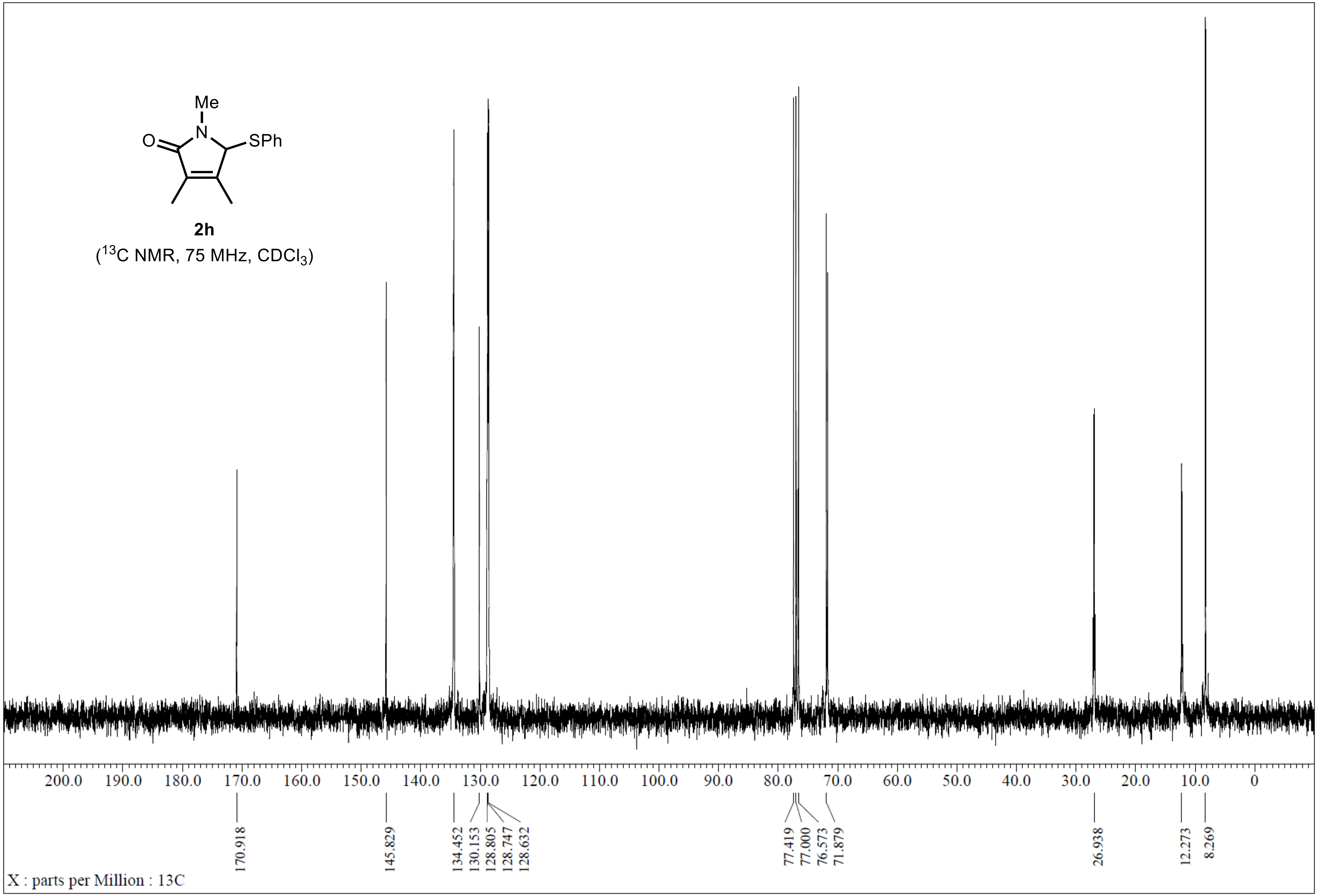
Me
N
O
SPh
2h
(^{13}C NMR, 75 MHz, CDCl$_3$)
200.0 190.0 180.0 170.0 160.0 150.0 140.0 130.0 120.0 110.0 100.0 90.0 80.0 70.0 60.0 50.0 40.0 30.0 20.0 10.0 0
170.918
145.829
134.452
130.153
128.805
128.747
128.632
77.419
77.000
76.573
71.879
26.938
12.273
8.269
X : parts per Million : 13C

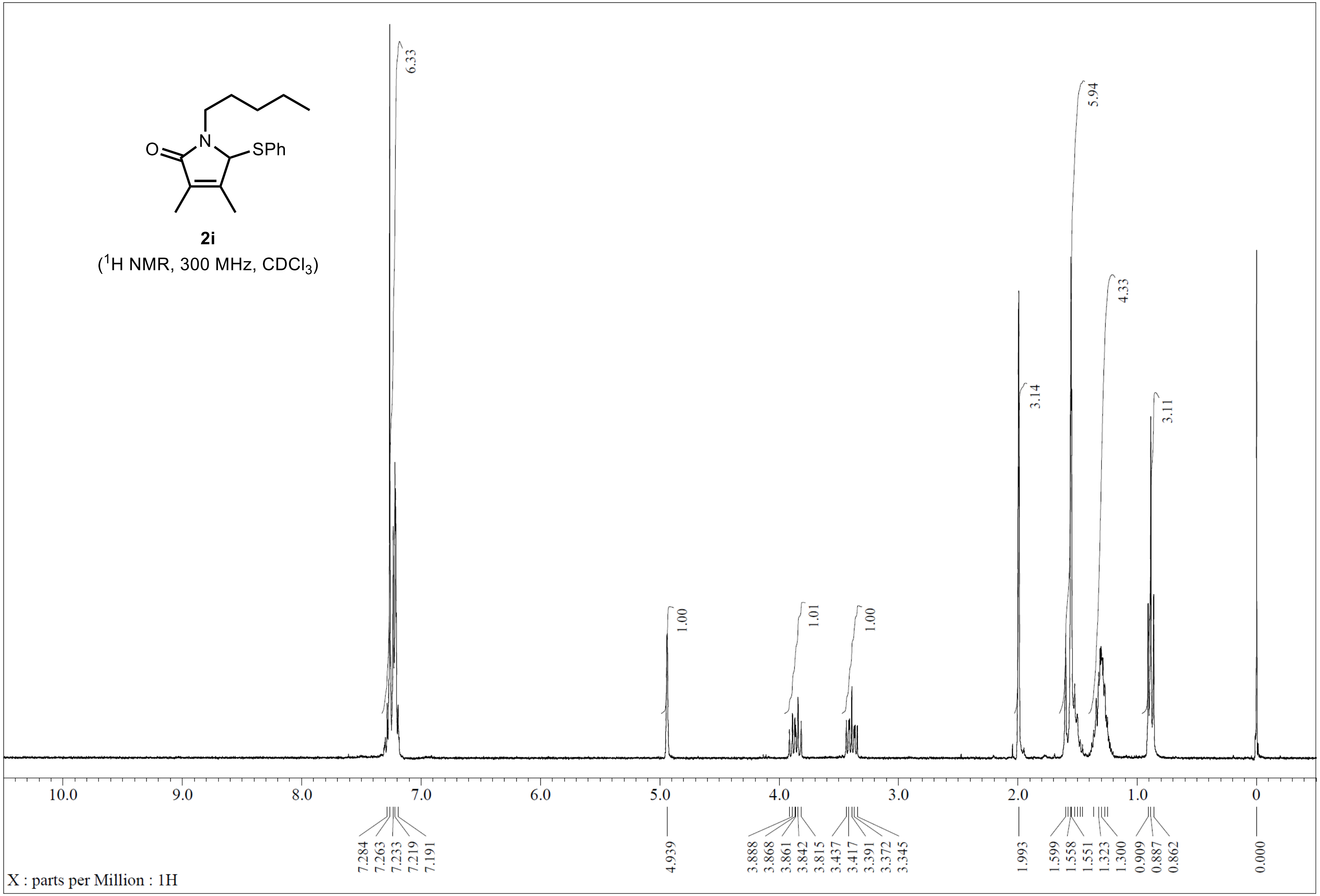

O
N
SPh
2i
(1H NMR, 300 MHz, CDCl3)
6.33
5.94
4.33
3.14
3.11
1.00
1.01
1.00
10.0
9.0
8.0
7.0
6.0
5.0
4.0
3.0
2.0
1.0
0
7.284
7.263
7.233
7.219
7.191
4.939
3.888
3.868
3.861
3.842
3.815
3.437
3.417
3.391
3.372
3.345
1.993
1.599
1.558
1.551
1.323
1.300
0.909
0.887
0.862
0.000
X : parts per Million : 1H

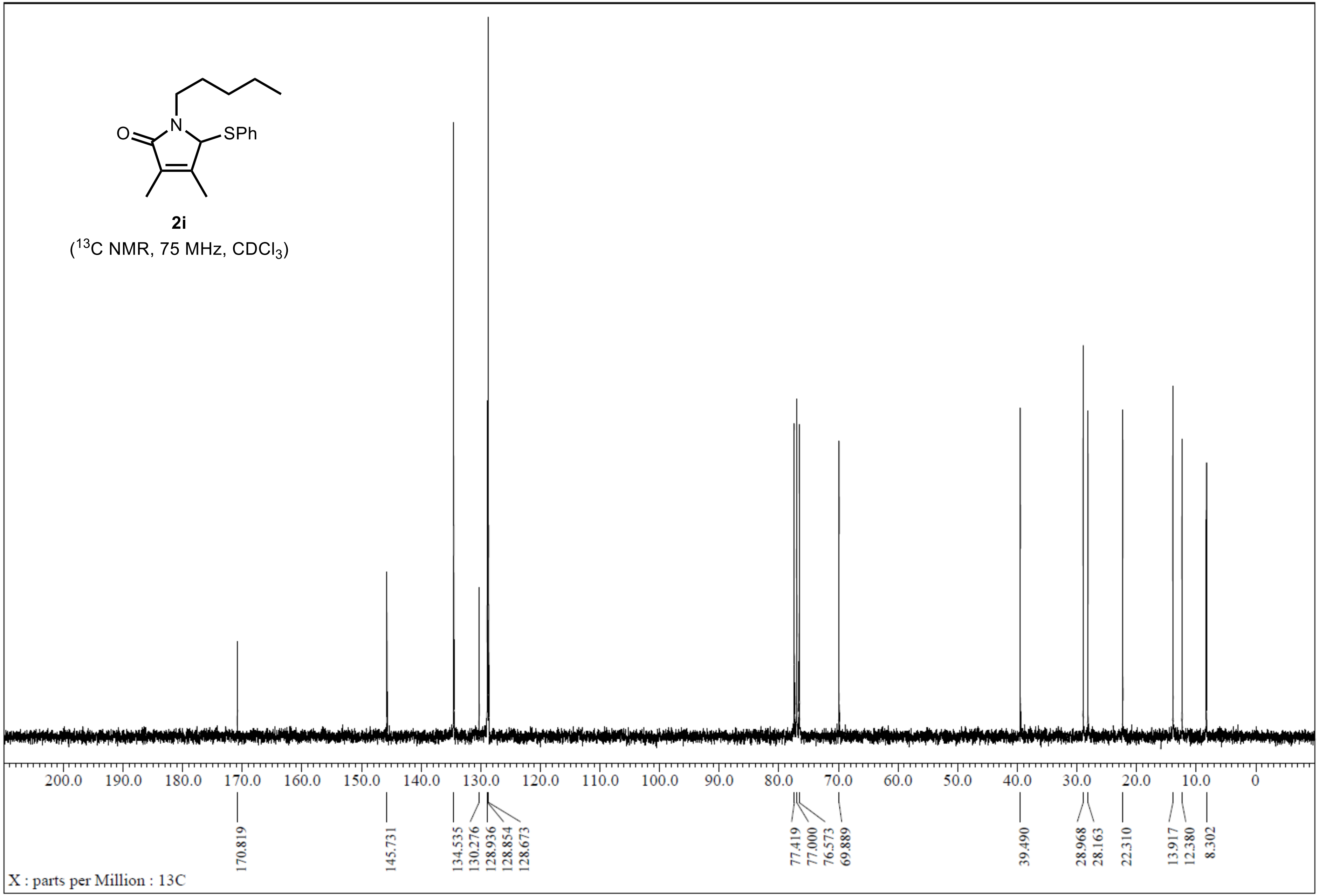
O
N
SPh
2i
(13C NMR, 75 MHz, CDCl3)
200.0 190.0 180.0 170.0 160.0 150.0 140.0 130.0 120.0 110.0 100.0 90.0 80.0 70.0 60.0 50.0 40.0 30.0 20.0 10.0 0
170.819
145.731
134.535
130.276
128.936
128.854
128.673
77.419
77.000
76.573
69.889
39.490
28.968
28.163
22.310
13.917
12.380
8.302
X : parts per Million : 13C

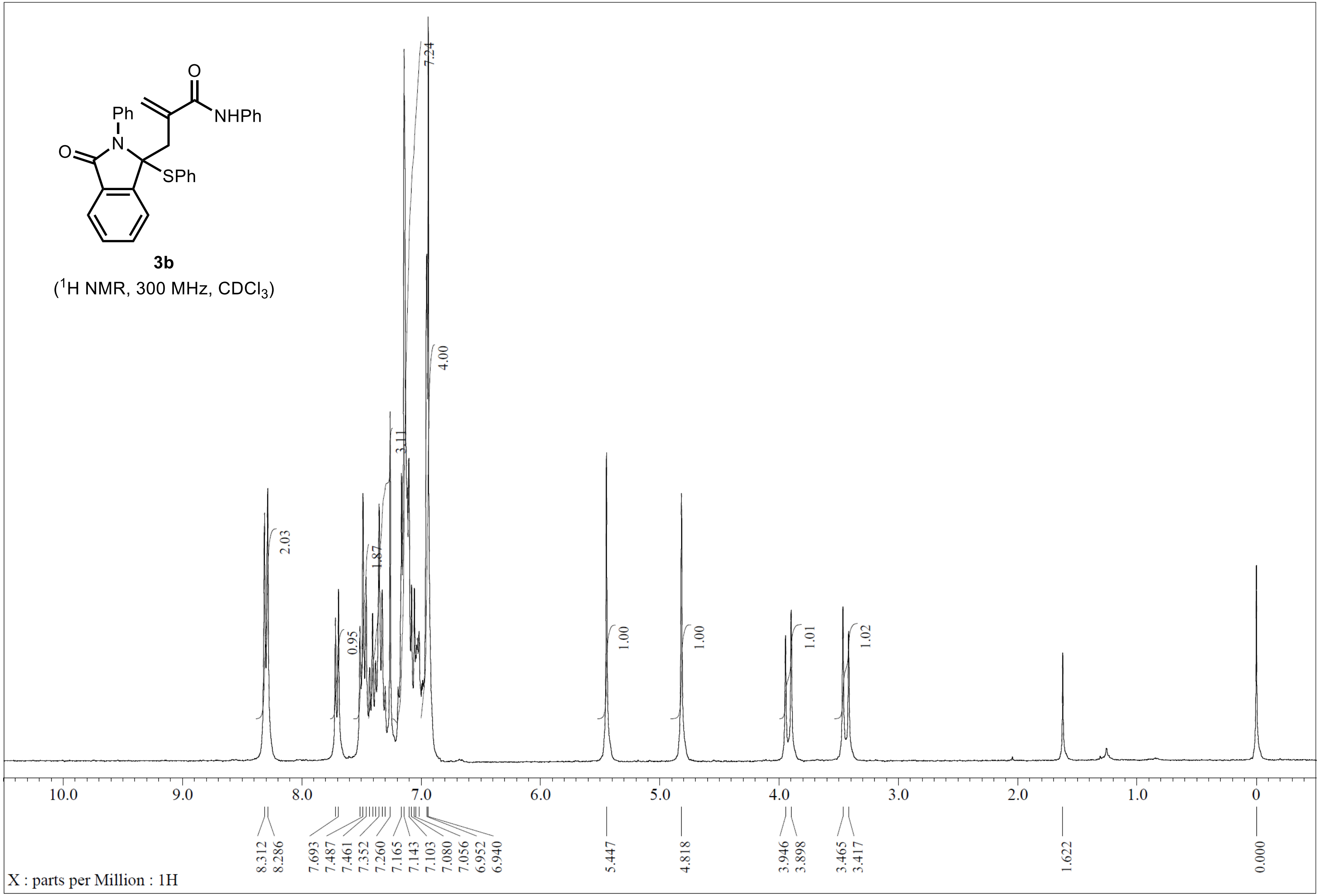
Ph
O
N
SPh
O
NHPh
3b
(1H NMR, 300 MHz, CDCl3)
2.03
0.95
1.87
3.11
7.24
4.00
1.00
1.00
1.01
1.02
10.0
9.0
8.0
7.0
6.0
5.0
4.0
3.0
2.0
1.0
0
8.312
8.286
7.693
7.487
7.461
7.352
7.260
7.165
7.143
7.103
7.080
7.056
6.952
6.940
5.447
4.818
3.946
3.898
3.465
3.417
1.622
0.000
X : parts per Million : 1H

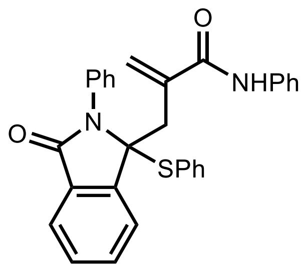

3b

(^{13}C NMR, 75 MHz, $CDCl_3$)

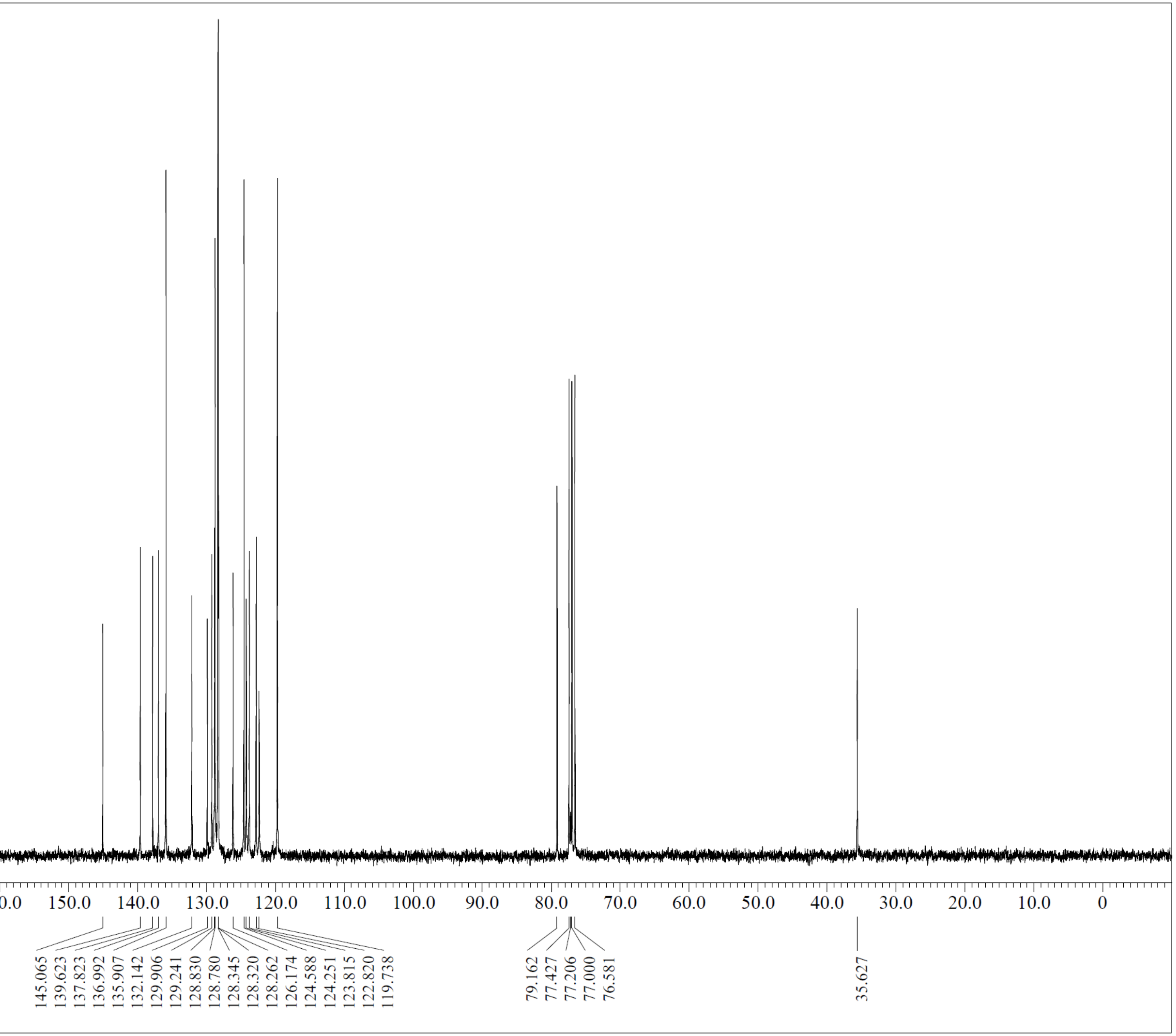

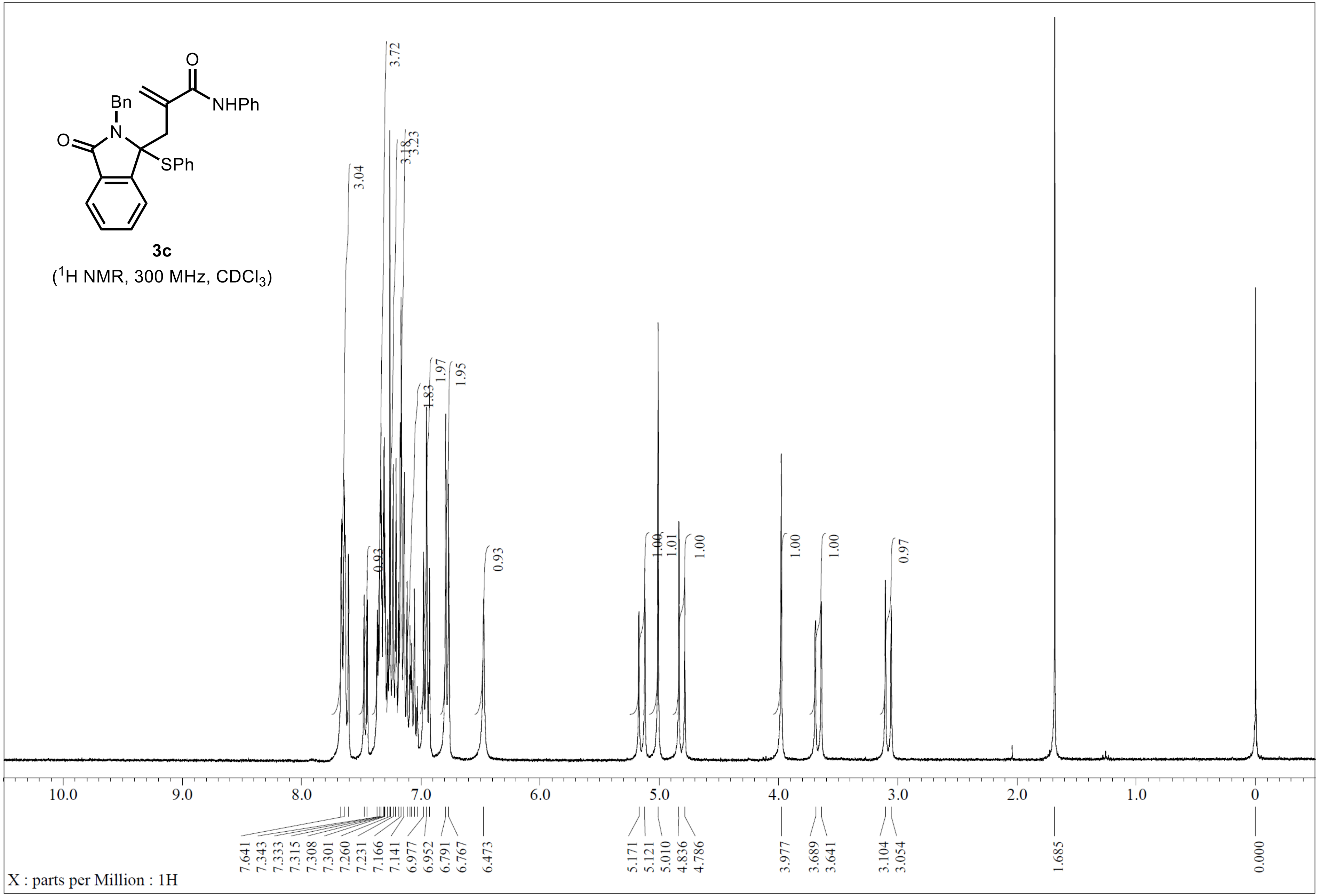

O
Bn
N
O
NHPh
SPh
3c
(^{1}H NMR, 300 MHz, $CDCl_3$)
3.72
3.04
3.18
3.23
1.83
1.97
1.95
0.93
0.93
1.00
1.01
1.00
1.00
1.00
0.97
10.0
9.0
8.0
7.0
6.0
5.0
4.0
3.0
2.0
1.0
0
7.641
7.343
7.333
7.315
7.308
7.301
7.260
7.231
7.166
7.141
6.977
6.952
6.791
6.767
6.473
5.171
5.121
5.010
4.836
4.786
3.977
3.689
3.641
3.104
3.054
1.685
0.000
X : parts per Million : 1H

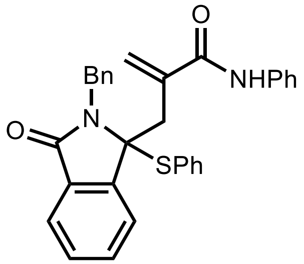

3c

(^{13}C NMR, 75 MHz, $CDCl_3$)

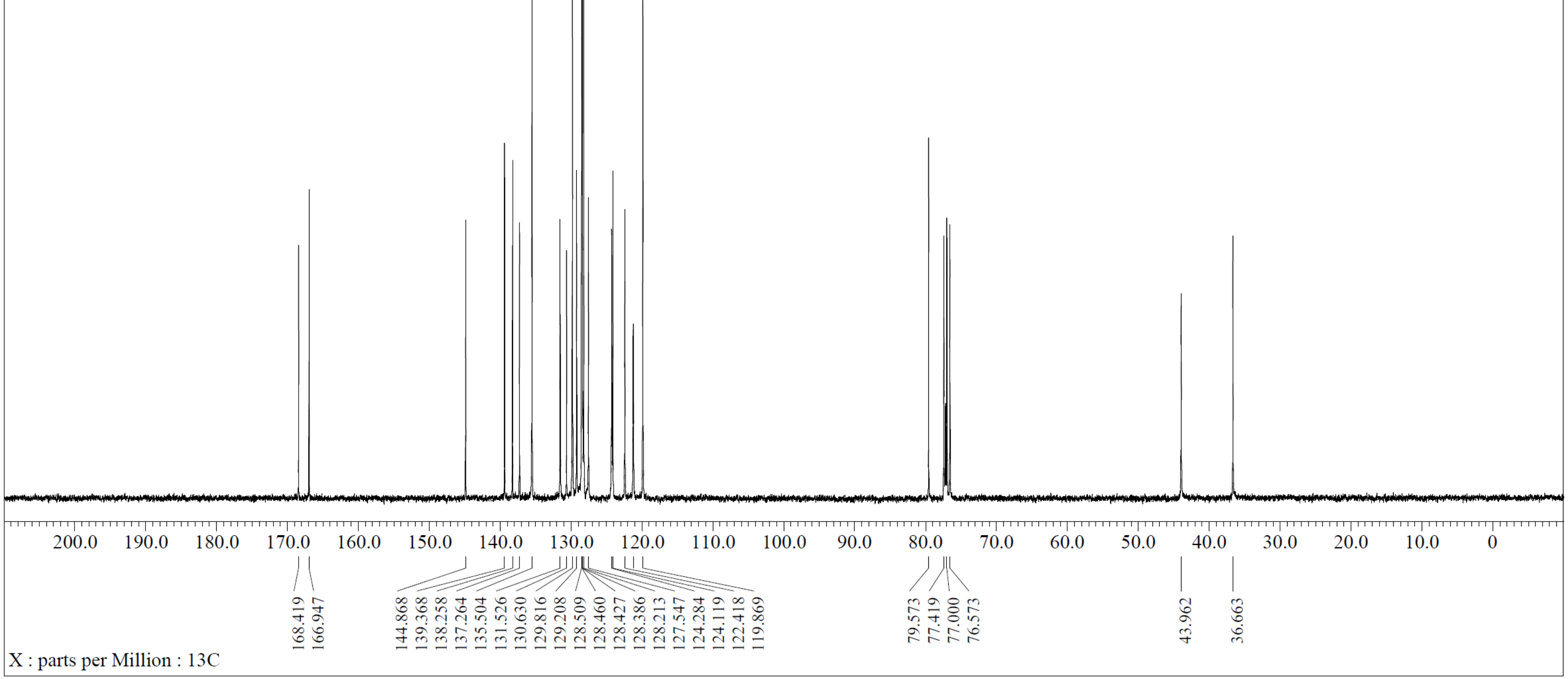

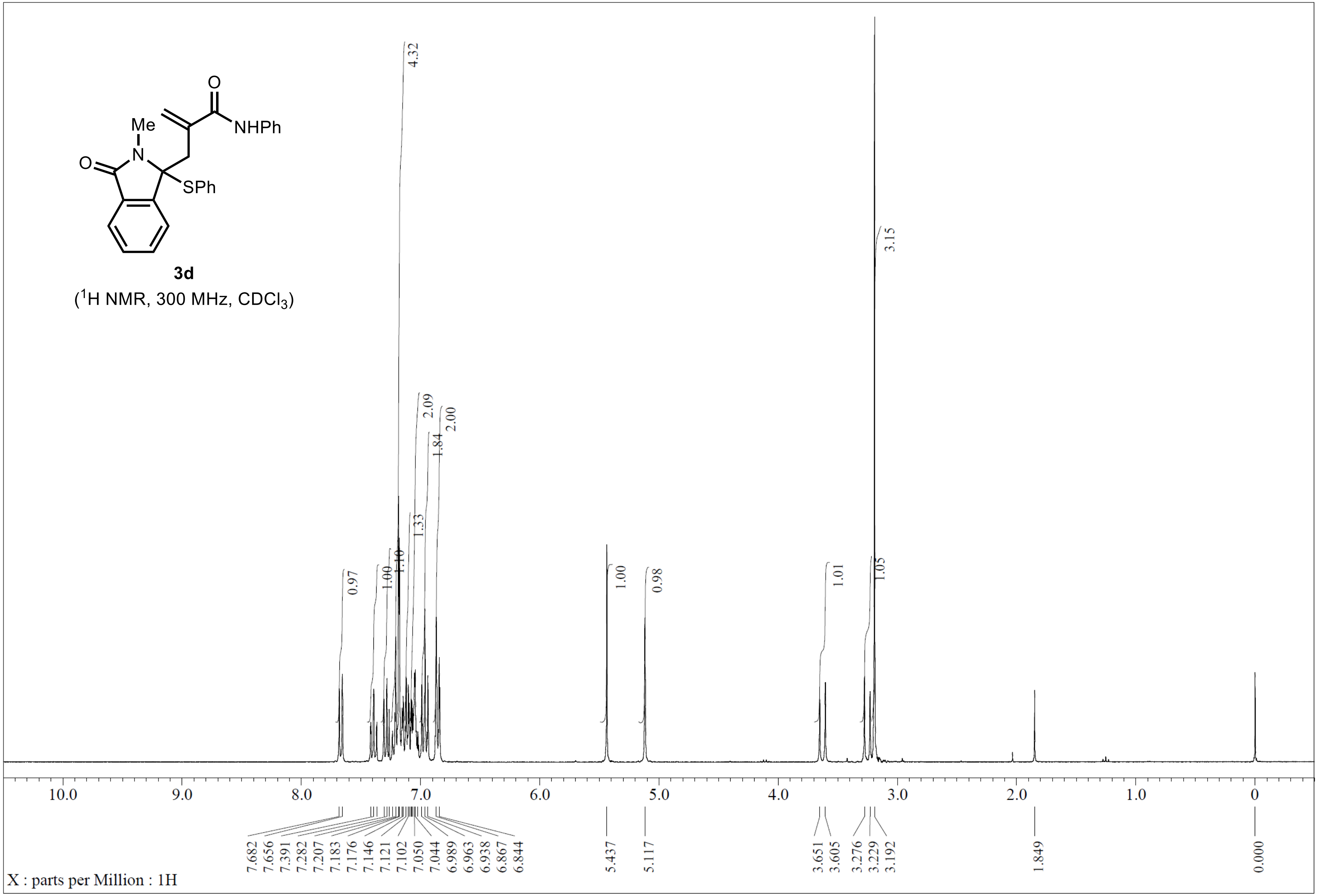

3d

(^{1}H NMR, 300 MHz, $CDCl_3$)

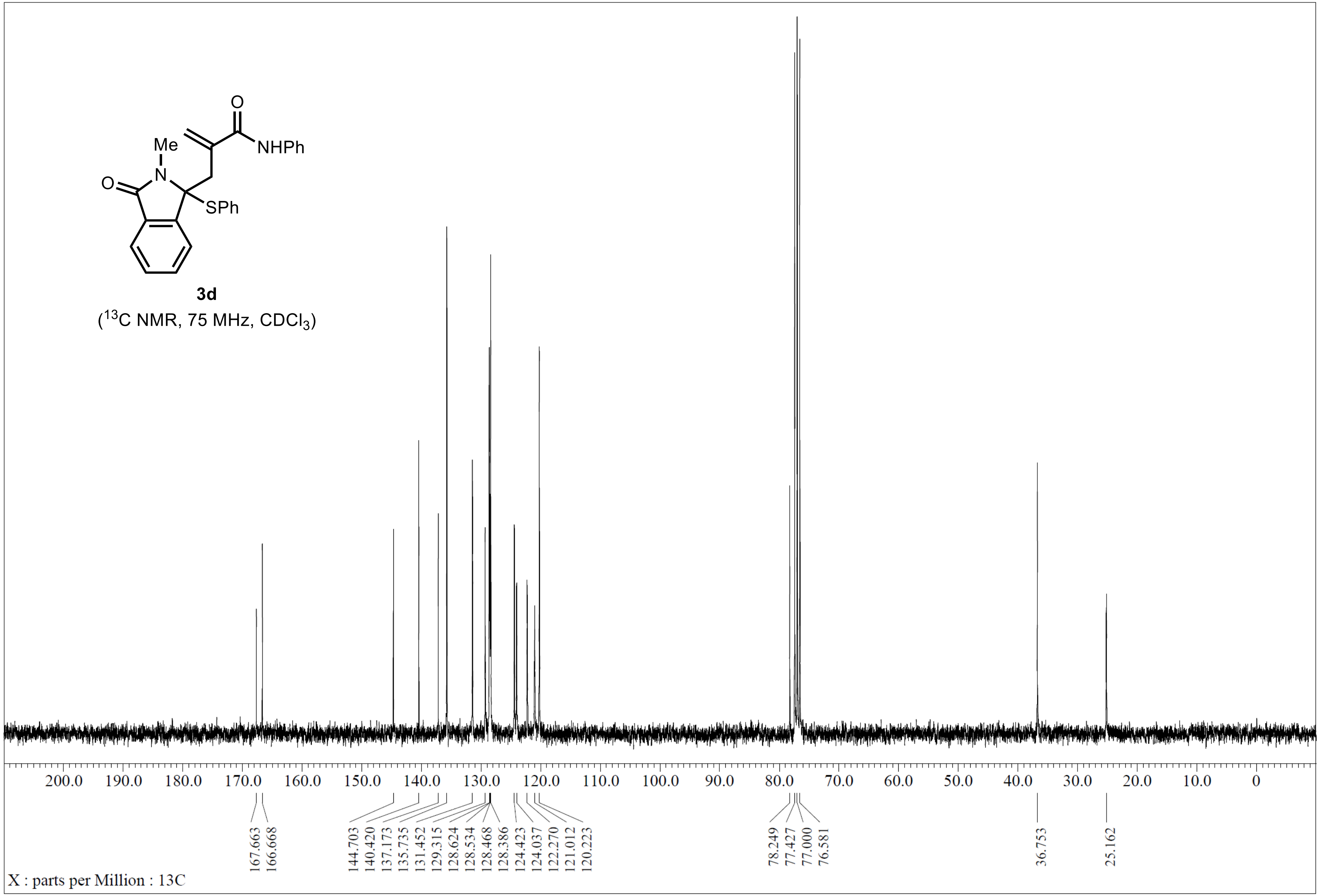

Me
O
N
O
NHPh
SPh
3d
(13C NMR, 75 MHz, CDCl3)
200.0 190.0 180.0 170.0 160.0 150.0 140.0 130.0 120.0 110.0 100.0 90.0 80.0 70.0 60.0 50.0 40.0 30.0 20.0 10.0 0
167.663
166.668
144.703
140.420
137.173
135.735
131.452
129.315
128.624
128.534
128.468
128.386
124.423
124.037
122.270
121.012
120.223
78.249
77.427
77.000
76.581
36.753
25.162
X : parts per Million : 13C

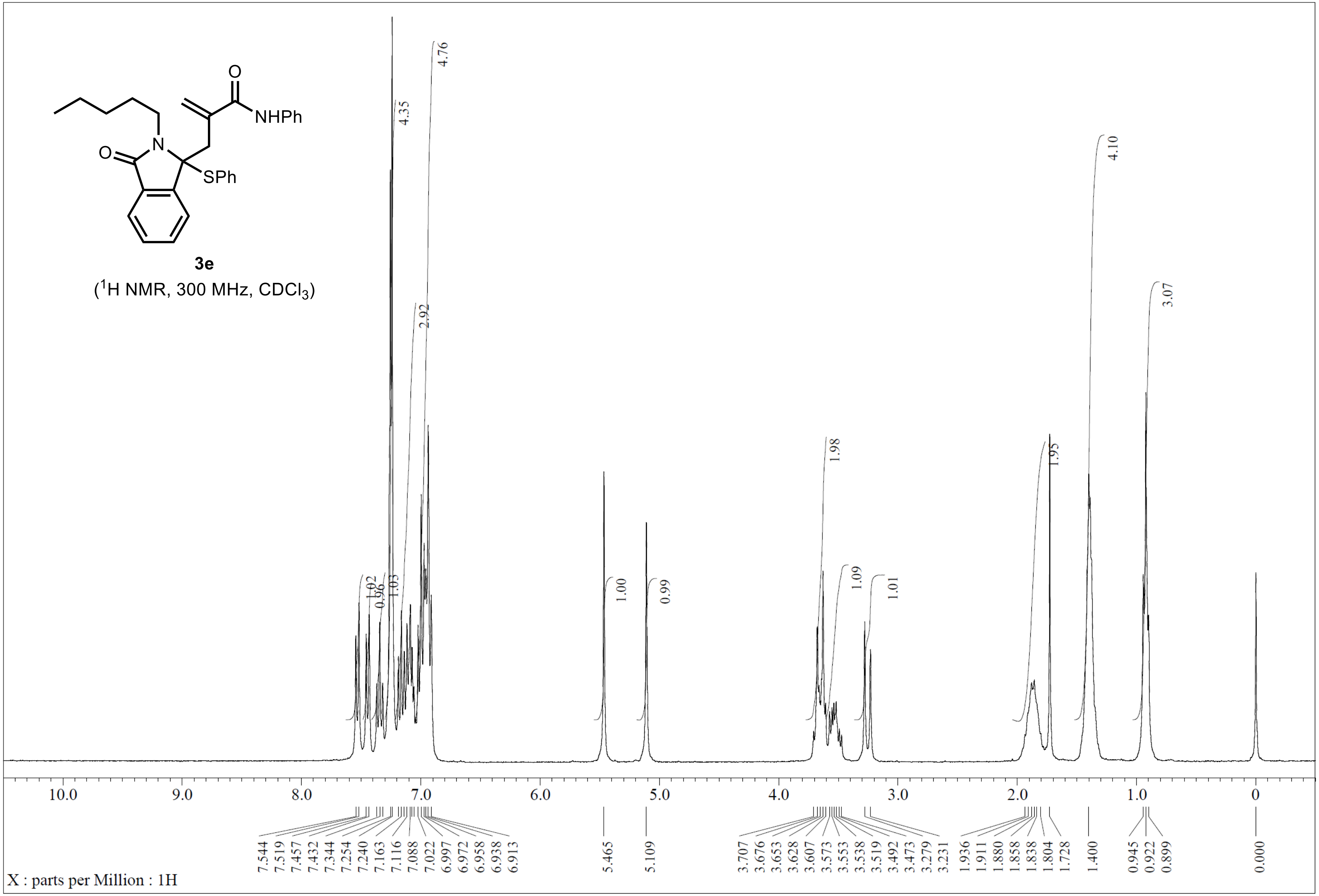
3e
(1H NMR, 300 MHz, CDCl3)
X : parts per Million : 1H

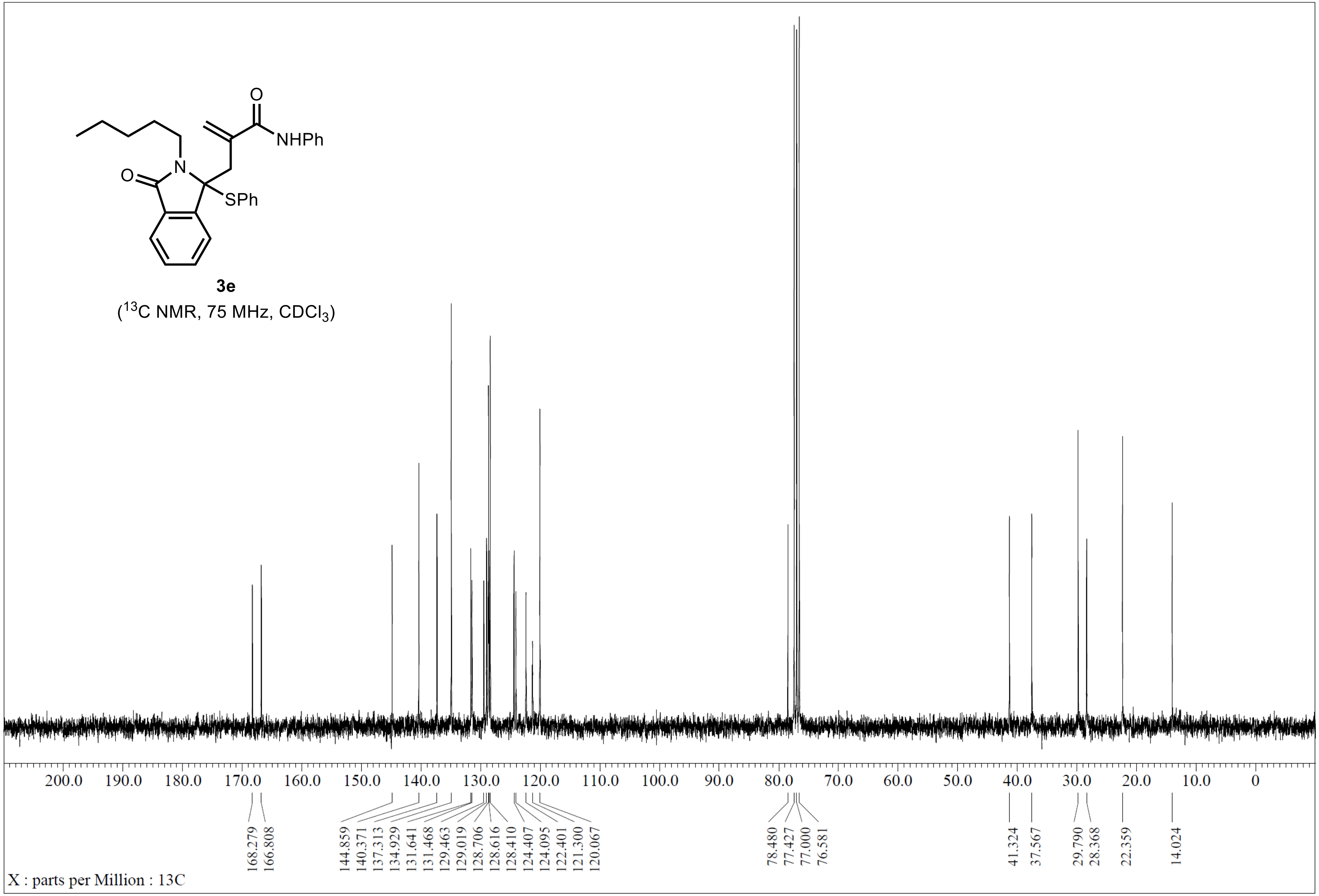
O
NHPh
N
O
SPh
3e
(13C NMR, 75 MHz, CDCl3)
200.0 190.0 180.0 170.0 160.0 150.0 140.0 130.0 120.0 110.0 100.0 90.0 80.0 70.0 60.0 50.0 40.0 30.0 20.0 10.0 0
168.279
166.808
144.859
140.371
137.313
134.929
131.641
131.468
129.463
129.019
128.706
128.616
128.410
124.407
124.095
122.401
121.300
120.067
78.480
77.427
77.000
76.581
41.324
37.567
29.790
28.368
22.359
14.024
X : parts per Million : 13C

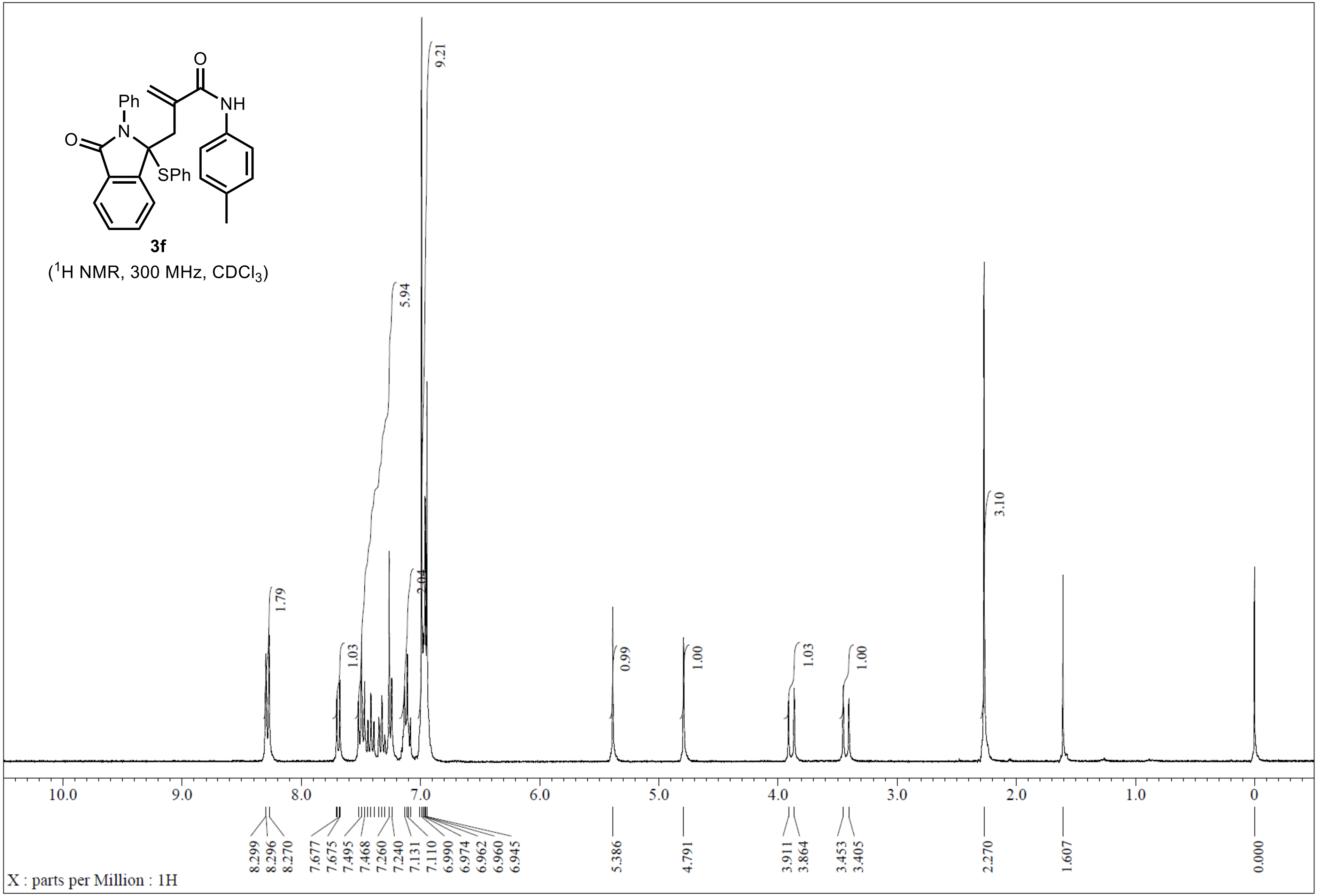
O
Ph
NH
N
O
SPh
3f
(¹H NMR, 300 MHz, CDCl₃)
9.21
5.94
1.79
1.03
2.04
0.99
1.00
1.03
1.00
3.10
10.0
9.0
8.0
7.0
6.0
5.0
4.0
3.0
2.0
1.0
0
8.299
8.296
8.270
7.677
7.675
7.495
7.468
7.260
7.240
7.131
7.110
6.990
6.974
6.962
6.960
6.945
5.386
4.791
3.911
3.864
3.453
3.405
2.270
1.607
0.000
X : parts per Million : 1H

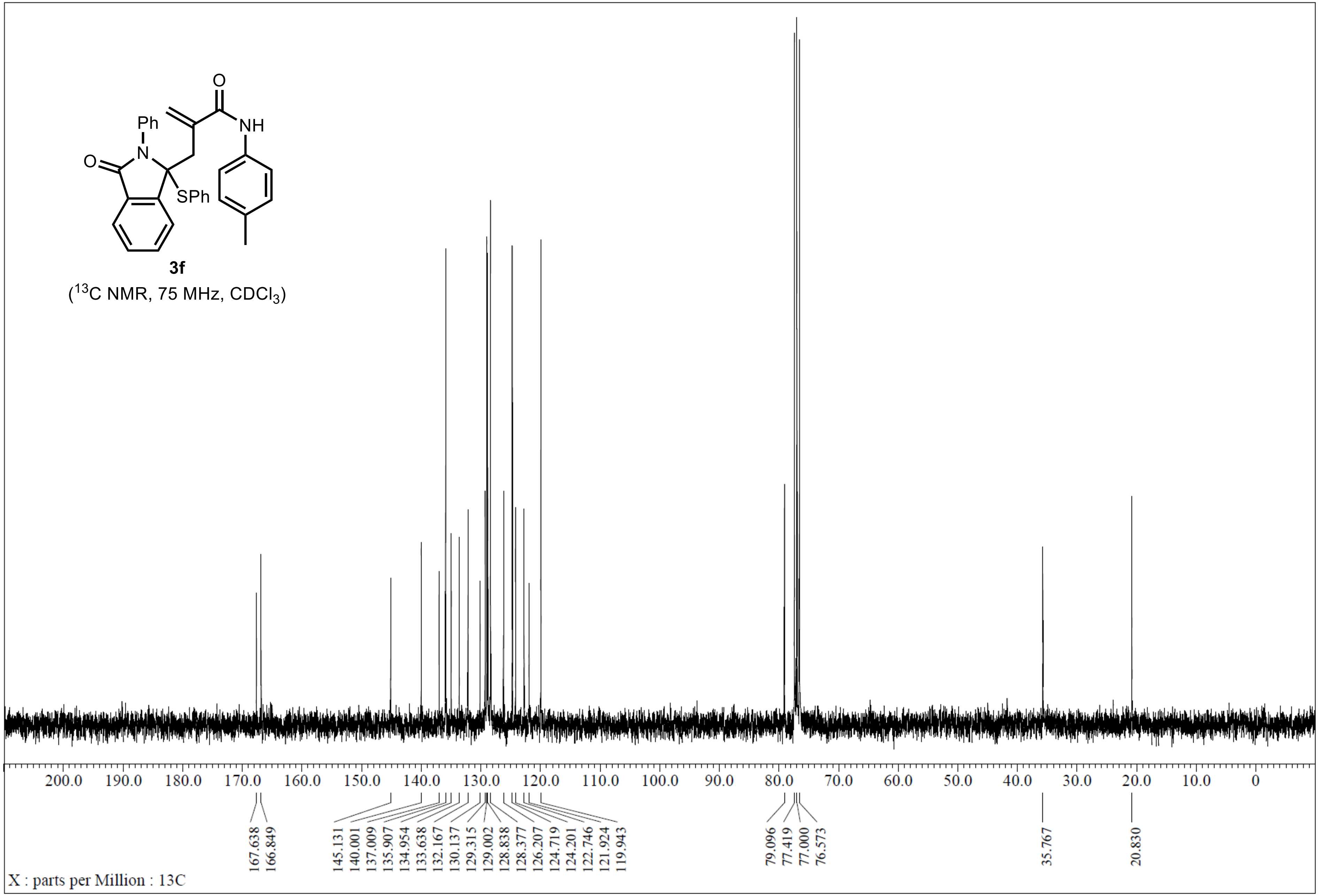
O
NH
Ph
N
O
SPh
3f
(^{13}C NMR, 75 MHz, $CDCl_3$)
200.0 190.0 180.0 170.0 160.0 150.0 140.0 130.0 120.0 110.0 100.0 90.0 80.0 70.0 60.0 50.0 40.0 30.0 20.0 10.0 0
167.638
166.849
145.131
140.001
137.009
135.907
134.954
133.638
132.167
130.137
129.315
129.002
128.838
128.377
126.207
124.719
124.201
122.746
121.924
119.943
79.096
77.419
77.000
76.573
35.767
20.830
X : parts per Million : 13C

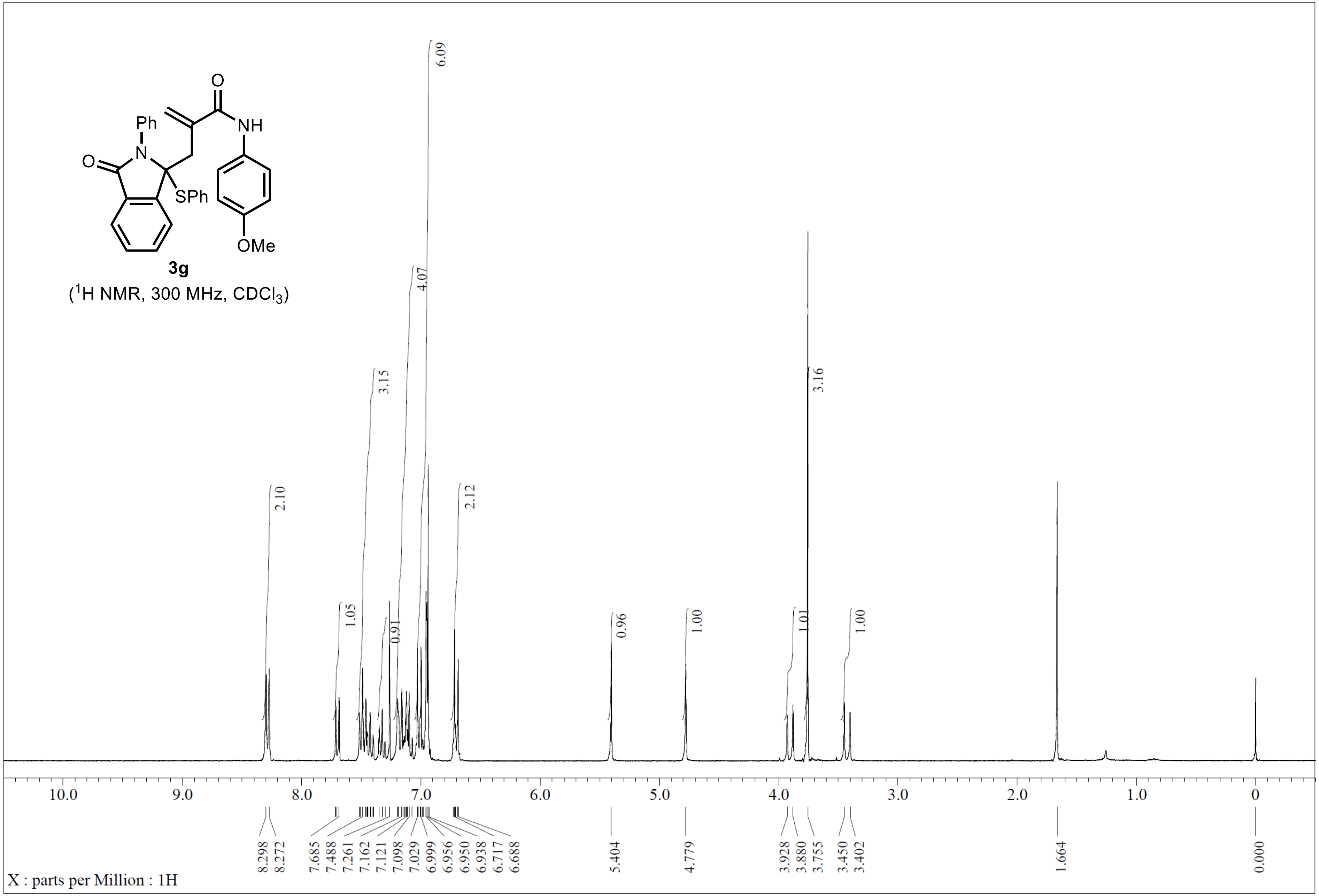
Ph
N
O
O
NH
SPh
OMe
3g
(1H NMR, 300 MHz, CDCl3)
6.09
4.07
3.15
3.16
2.10
2.12
1.05
0.91
0.96
1.00
1.01
1.00
10.0
9.0
8.0
7.0
6.0
5.0
4.0
3.0
2.0
1.0
0
8.298
8.272
7.685
7.488
7.261
7.162
7.121
7.098
7.029
6.999
6.956
6.950
6.938
6.717
6.688
5.404
4.779
3.928
3.880
3.755
3.450
3.402
1.664
0.000
X : parts per Million : 1H

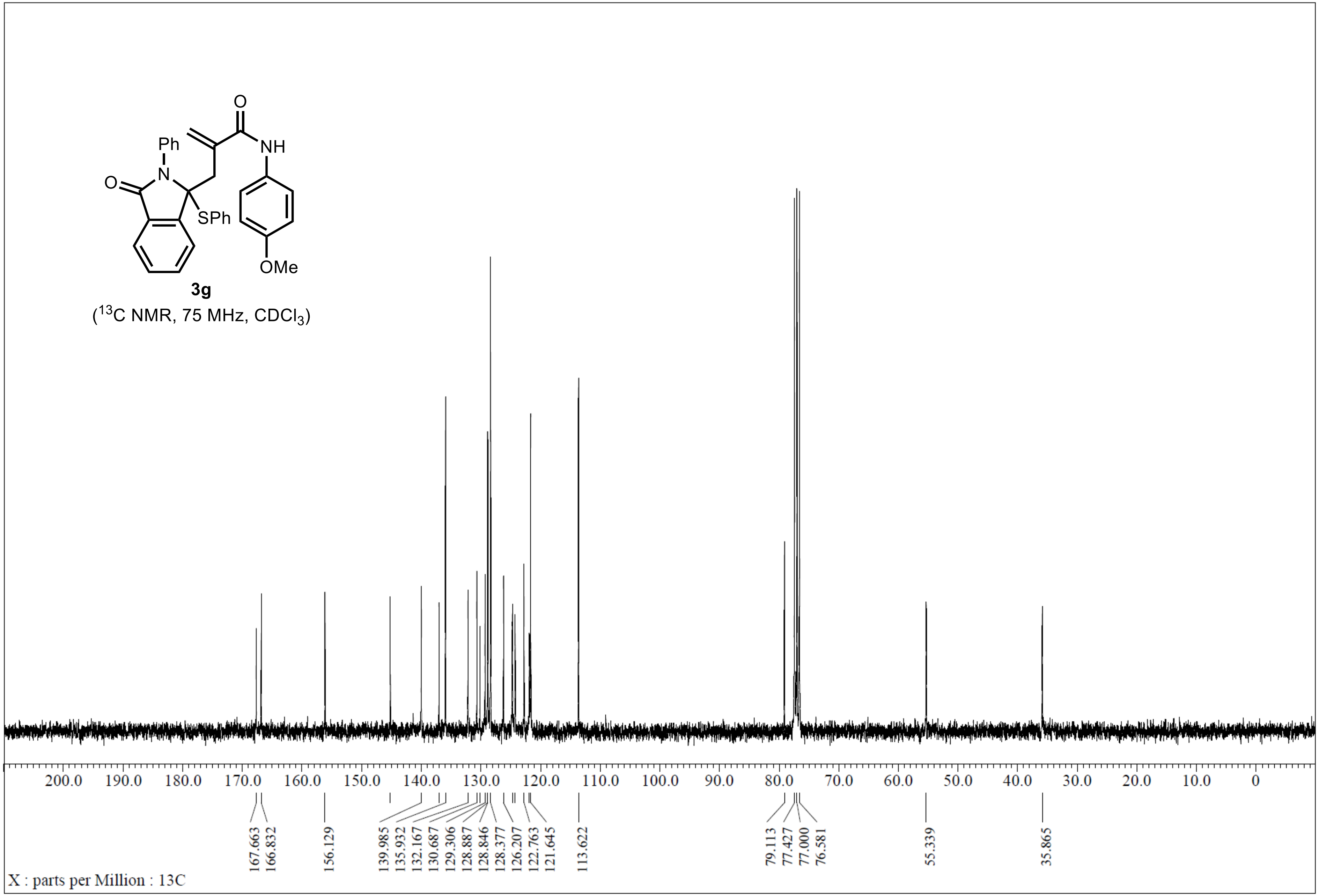

3g
(13C NMR, 75 MHz, CDCl3)
200.0
190.0
180.0
170.0
160.0
150.0
140.0
130.0
120.0
110.0
100.0
90.0
80.0
70.0
60.0
50.0
40.0
30.0
20.0
10.0
0
167.663
166.832
156.129
139.985
135.932
132.167
130.687
129.306
128.887
128.846
128.377
126.207
122.763
121.645
113.622
79.113
77.427
77.000
76.581
55.339
35.865
X : parts per Million : 13C

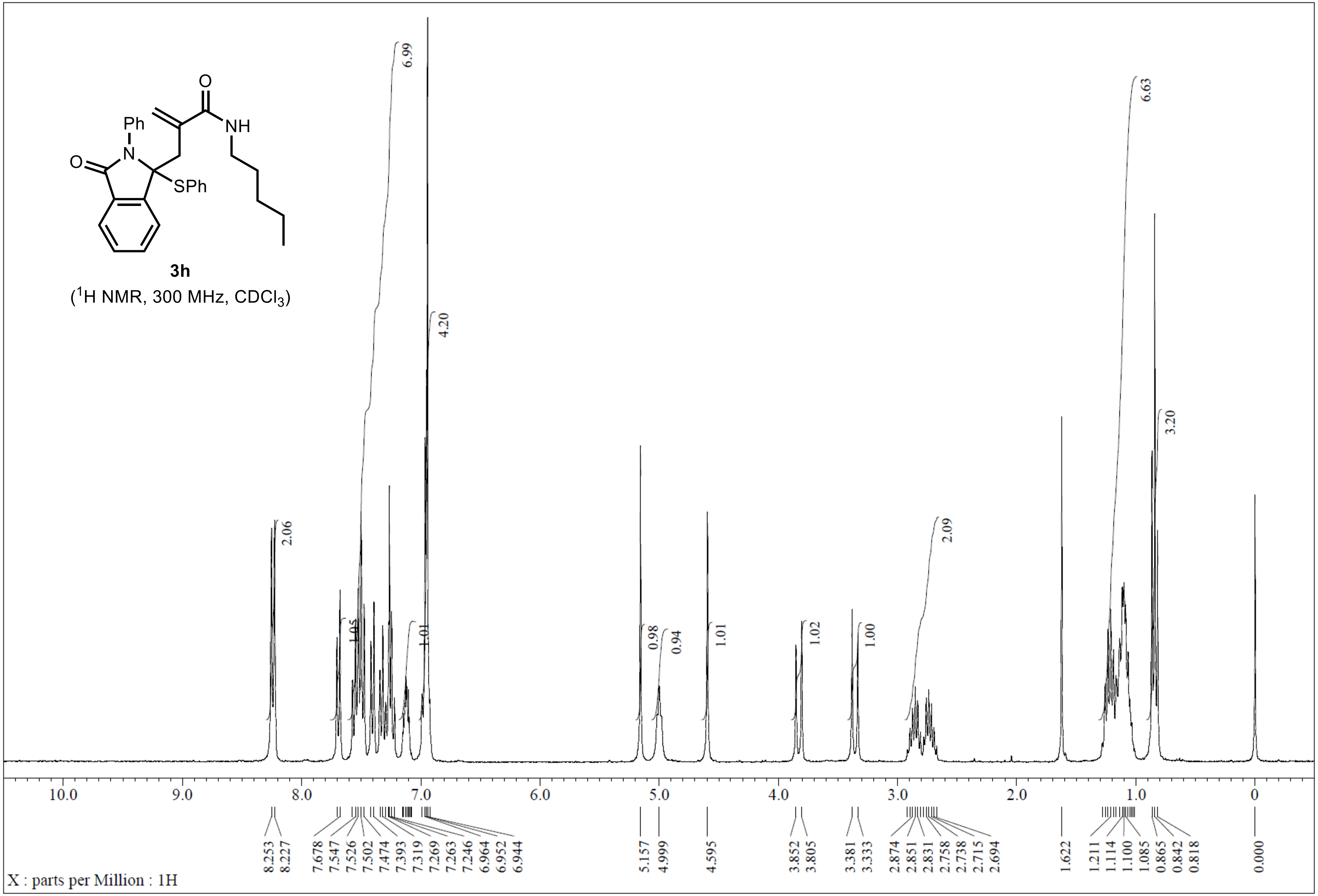
O
Ph
NH
N
O
SPh
3h
(1H NMR, 300 MHz, CDCl3)
6.99
4.20
6.63
3.20
2.06
1.05
1.01
0.98
0.94
1.01
1.02
1.00
2.09
10.0
9.0
8.0
7.0
6.0
5.0
4.0
3.0
2.0
1.0
0
8.253
8.227
7.678
7.547
7.526
7.502
7.474
7.393
7.319
7.269
7.263
7.246
6.964
6.952
6.944
5.157
4.999
4.595
3.852
3.805
3.381
3.333
2.874
2.851
2.831
2.758
2.738
2.715
2.694
1.622
1.211
1.114
1.100
1.085
0.865
0.842
0.818
0.000
X : parts per Million : 1H

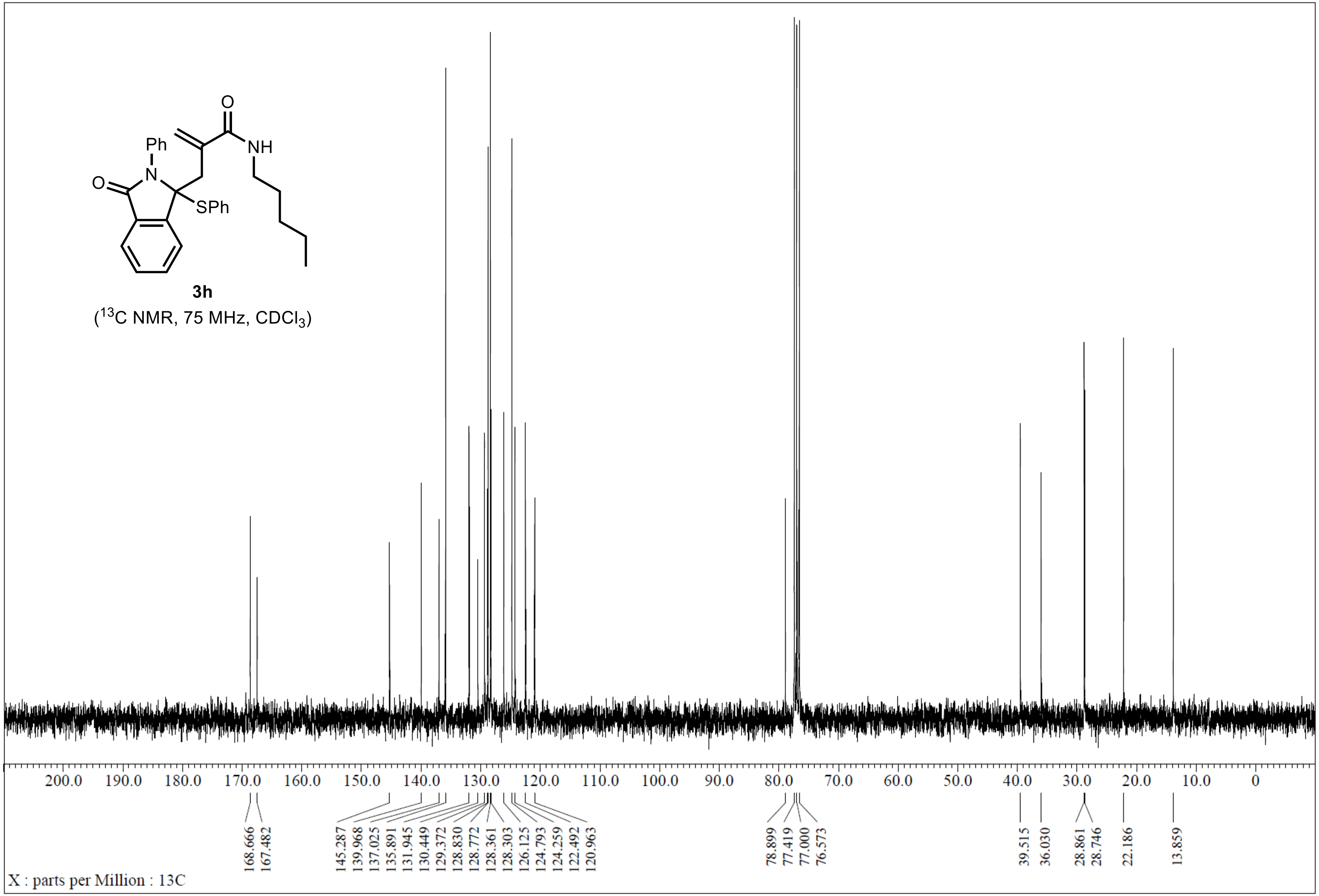

Ph
N
O
SPh
O
NH
3h
(13C NMR, 75 MHz, CDCl3)
200.0 190.0 180.0 170.0 160.0 150.0 140.0 130.0 120.0 110.0 100.0 90.0 80.0 70.0 60.0 50.0 40.0 30.0 20.0 10.0 0
168.666
167.482
145.287
139.968
137.025
135.891
131.945
130.449
129.372
128.830
128.772
128.361
128.303
126.125
124.793
124.259
122.492
120.963
78.899
77.419
77.000
76.573
39.515
36.030
28.861
28.746
22.186
13.859
X : parts per Million : 13C

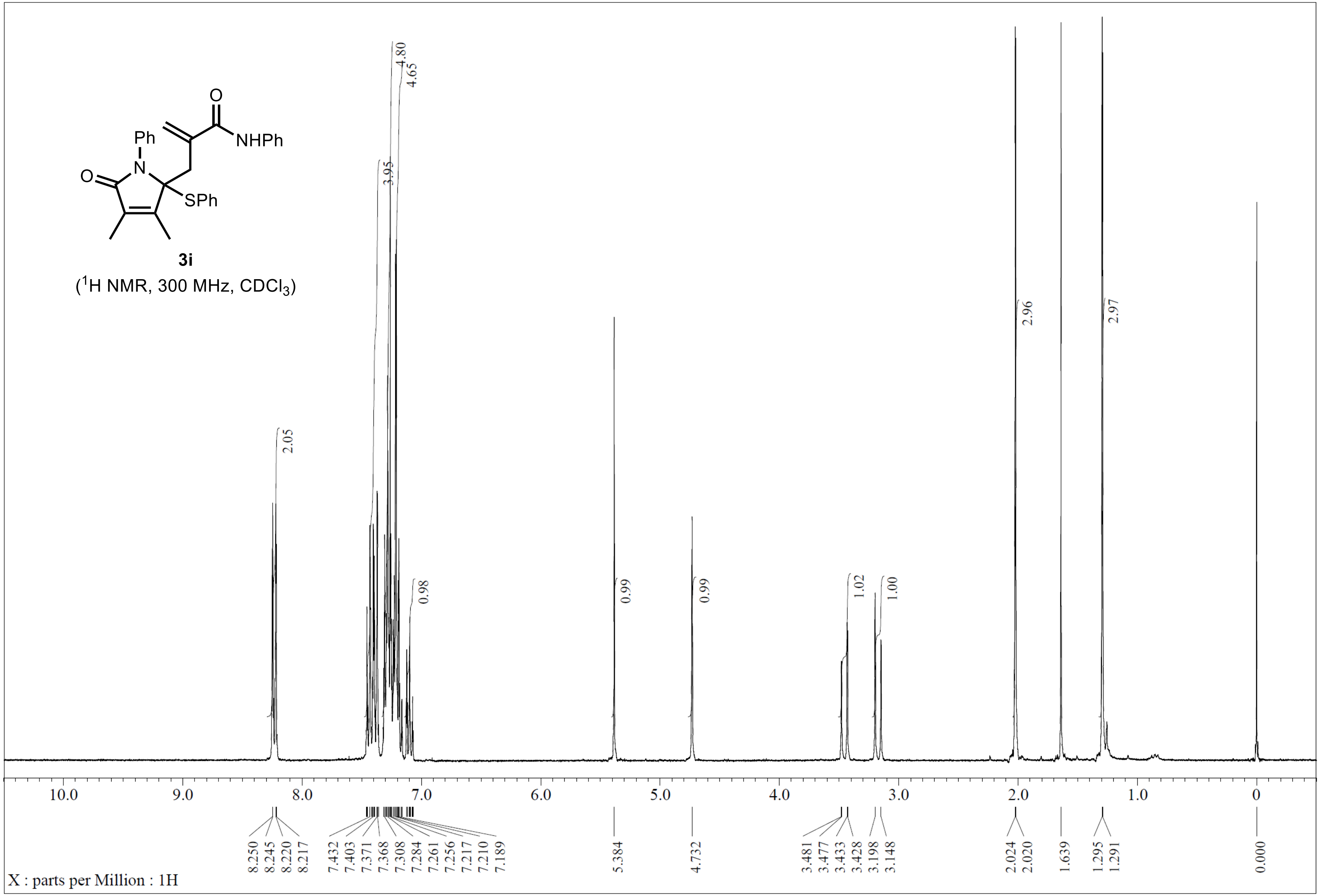

O
Ph
NHPh
O
N
SPh
3i
(1H NMR, 300 MHz, CDCl3)
4.80
4.65
3.95
2.05
0.98
0.99
0.99
1.02
1.00
2.96
2.97
10.0
9.0
8.0
7.0
6.0
5.0
4.0
3.0
2.0
1.0
0
8.250
8.245
8.220
8.217
7.432
7.403
7.371
7.368
7.308
7.284
7.261
7.256
7.217
7.210
7.189
5.384
4.732
3.481
3.477
3.433
3.428
3.198
3.148
2.024
2.020
1.639
1.295
1.291
0.000
X : parts per Million : 1H

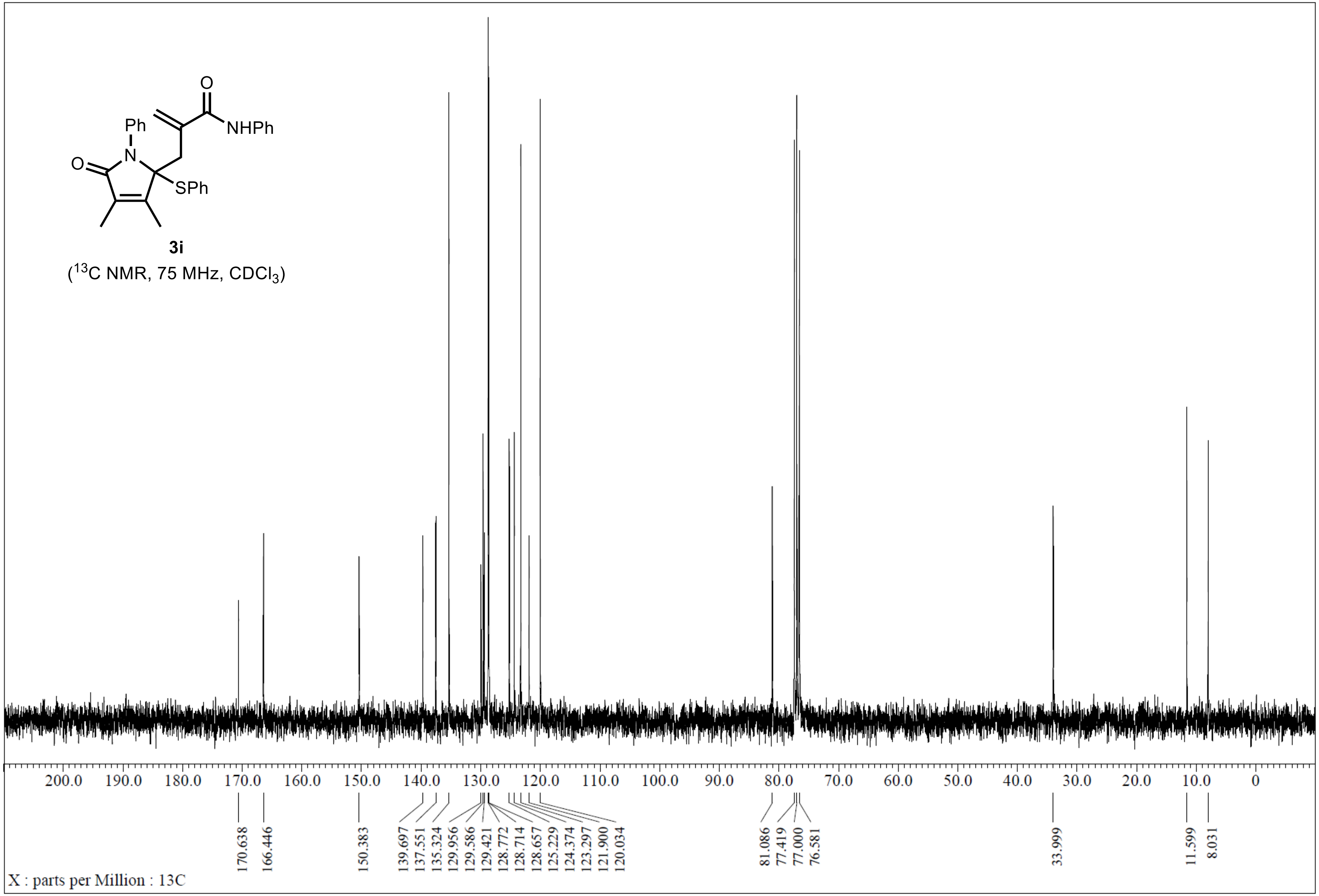

O
Ph
N
O
NHPh
SPh
3i
(^{13}C NMR, 75 MHz, CDCl$_3$)
200.0 190.0 180.0 170.0 160.0 150.0 140.0 130.0 120.0 110.0 100.0 90.0 80.0 70.0 60.0 50.0 40.0 30.0 20.0 10.0 0
170.638
166.446
150.383
139.697
137.551
135.324
129.956
129.586
129.421
128.772
128.714
128.657
125.229
124.374
123.297
121.900
120.034
81.086
77.419
77.000
76.581
33.999
11.599
8.031
X : parts per Million : 13C

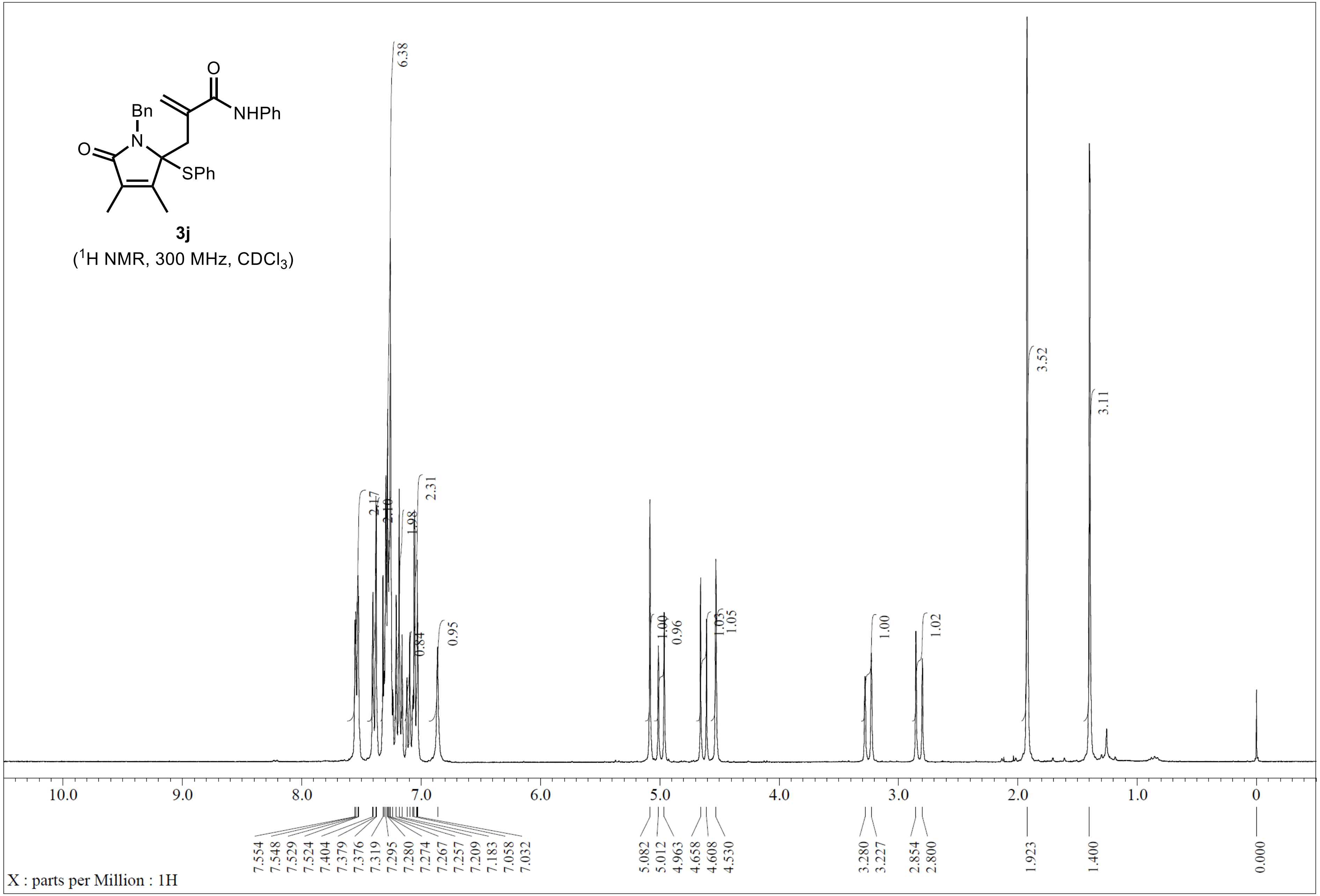

Bn
O
NHPh
SPh
3j
(1H NMR, 300 MHz, CDCl3)
6.38
2.17
2.10
1.98
2.31
0.84
0.95
1.00
0.96
1.05
1.05
1.00
1.02
3.52
3.11
10.0
9.0
8.0
7.0
6.0
5.0
4.0
3.0
2.0
1.0
0
7.554
7.548
7.529
7.524
7.404
7.379
7.376
7.319
7.295
7.280
7.274
7.267
7.257
7.209
7.183
7.058
7.032
5.082
5.012
4.963
4.658
4.608
4.530
3.280
3.227
2.854
2.800
1.923
1.400
0.000
X : parts per Million : 1H

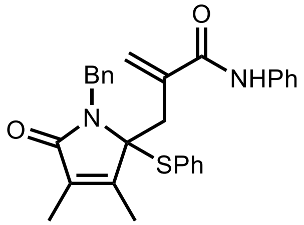

3j

(^{13}C NMR, 75 MHz, $CDCl_3$)

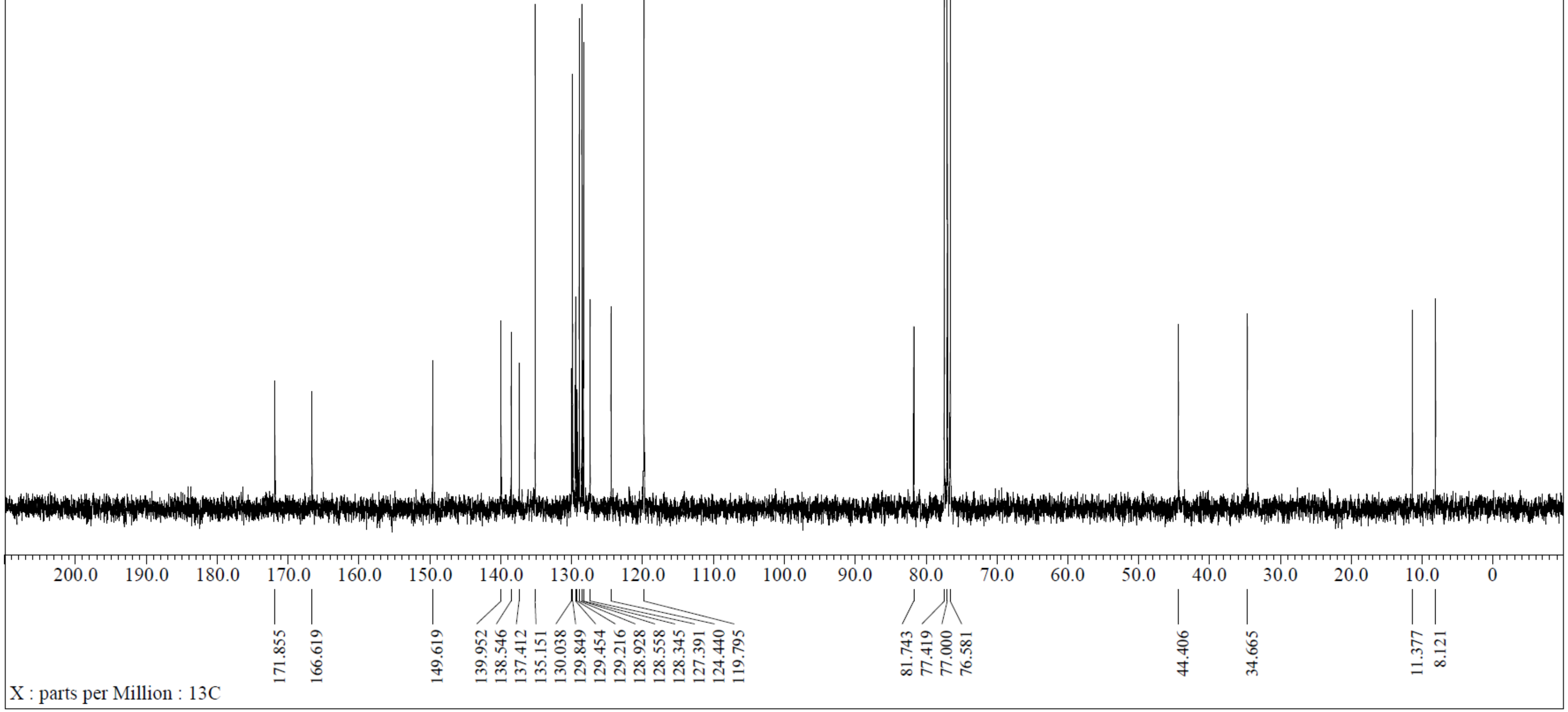

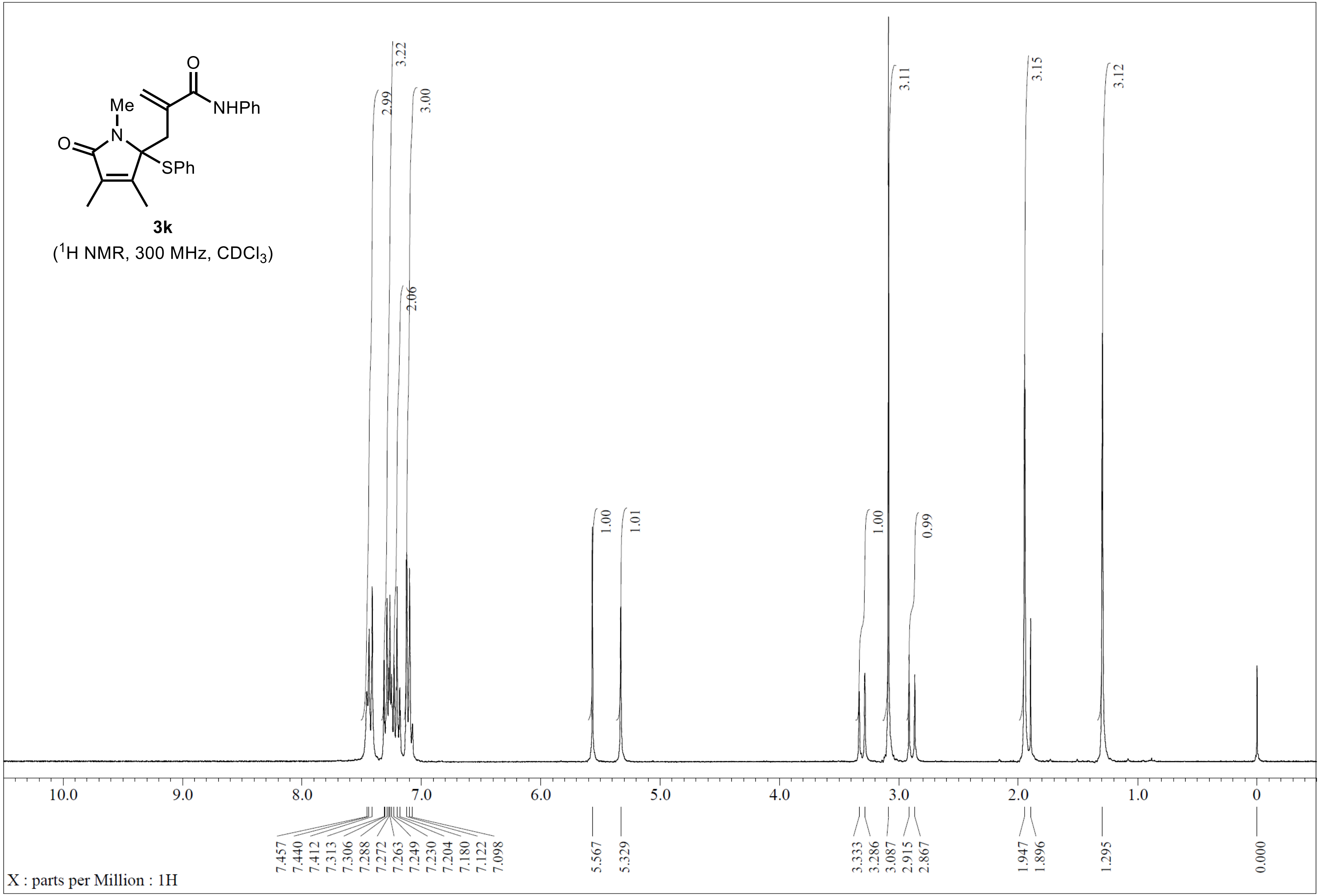
Me
N
O
O
NHPh
SPh
3k
(¹H NMR, 300 MHz, CDCl₃)
2.99
3.22
3.00
2.06
1.00
1.01
1.00
3.11
0.99
3.15
3.12
10.0
9.0
8.0
7.0
6.0
5.0
4.0
3.0
2.0
1.0
0
7.457
7.440
7.412
7.313
7.306
7.288
7.272
7.263
7.249
7.230
7.204
7.180
7.122
7.098
5.567
5.329
3.333
3.286
3.087
2.915
2.867
1.947
1.896
1.295
0.000
X : parts per Million : 1H

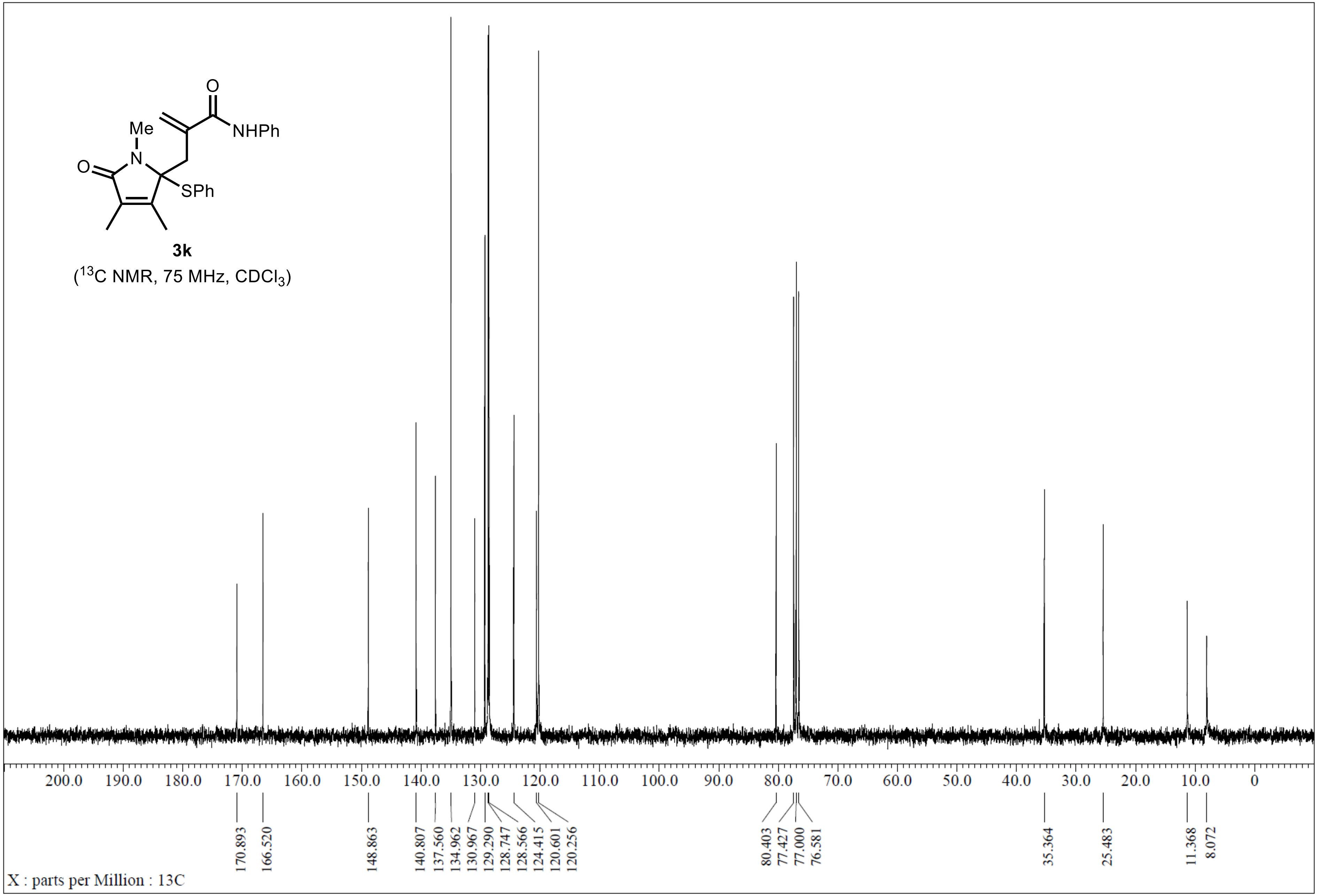

Me
N
O
SPh
O
NHPh
3k
(13C NMR, 75 MHz, CDCl3)
200.0
190.0
180.0
170.0
160.0
150.0
140.0
130.0
120.0
110.0
100.0
90.0
80.0
70.0
60.0
50.0
40.0
30.0
20.0
10.0
0
170.893
166.520
148.863
140.807
137.560
134.962
130.967
129.290
128.747
128.566
124.415
120.601
120.256
80.403
77.427
77.000
76.581
35.364
25.483
11.368
8.072
X : parts per Million : 13C

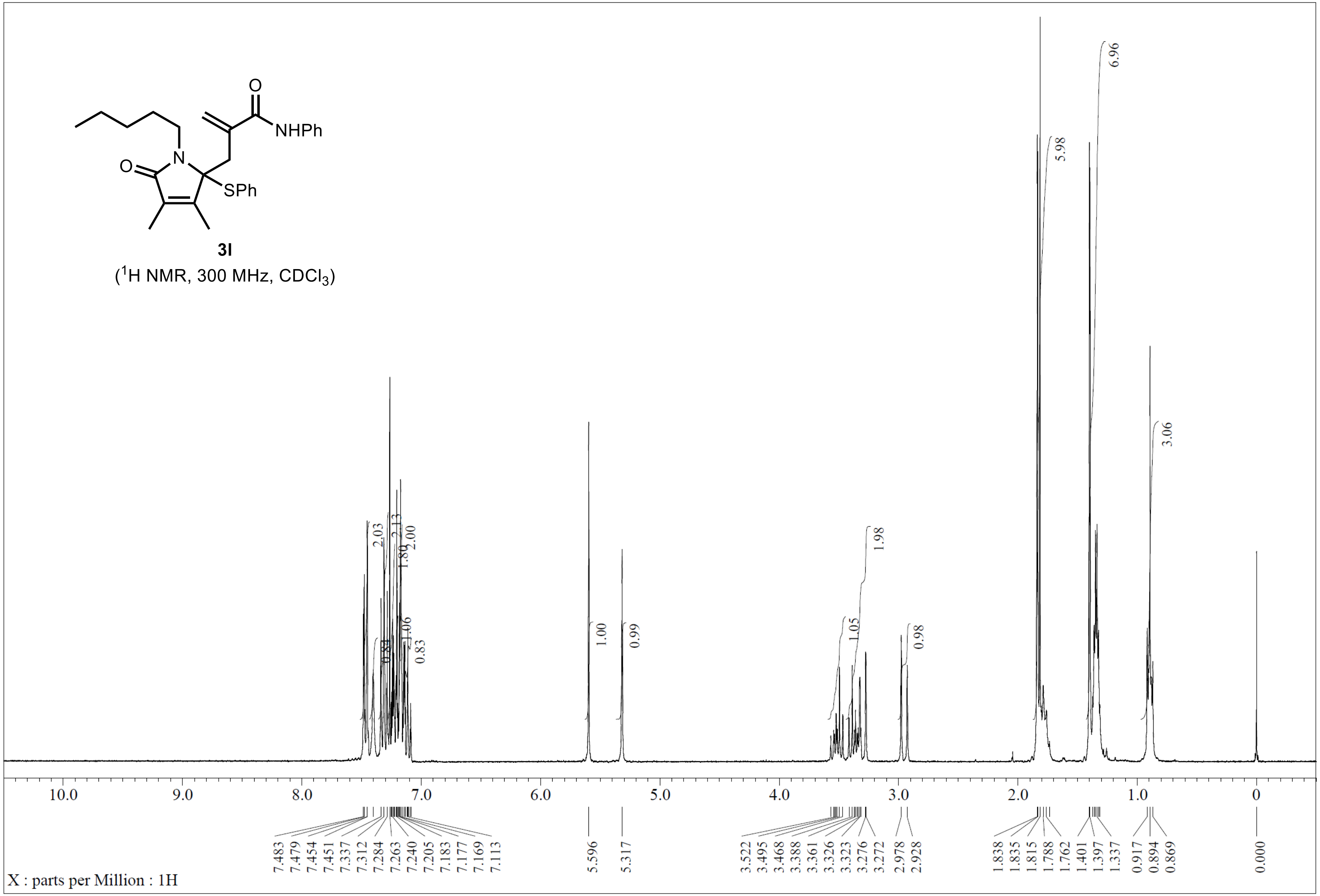
NHPh
SPh
3l
(1H NMR, 300 MHz, CDCl3)
X : parts per Million : 1H

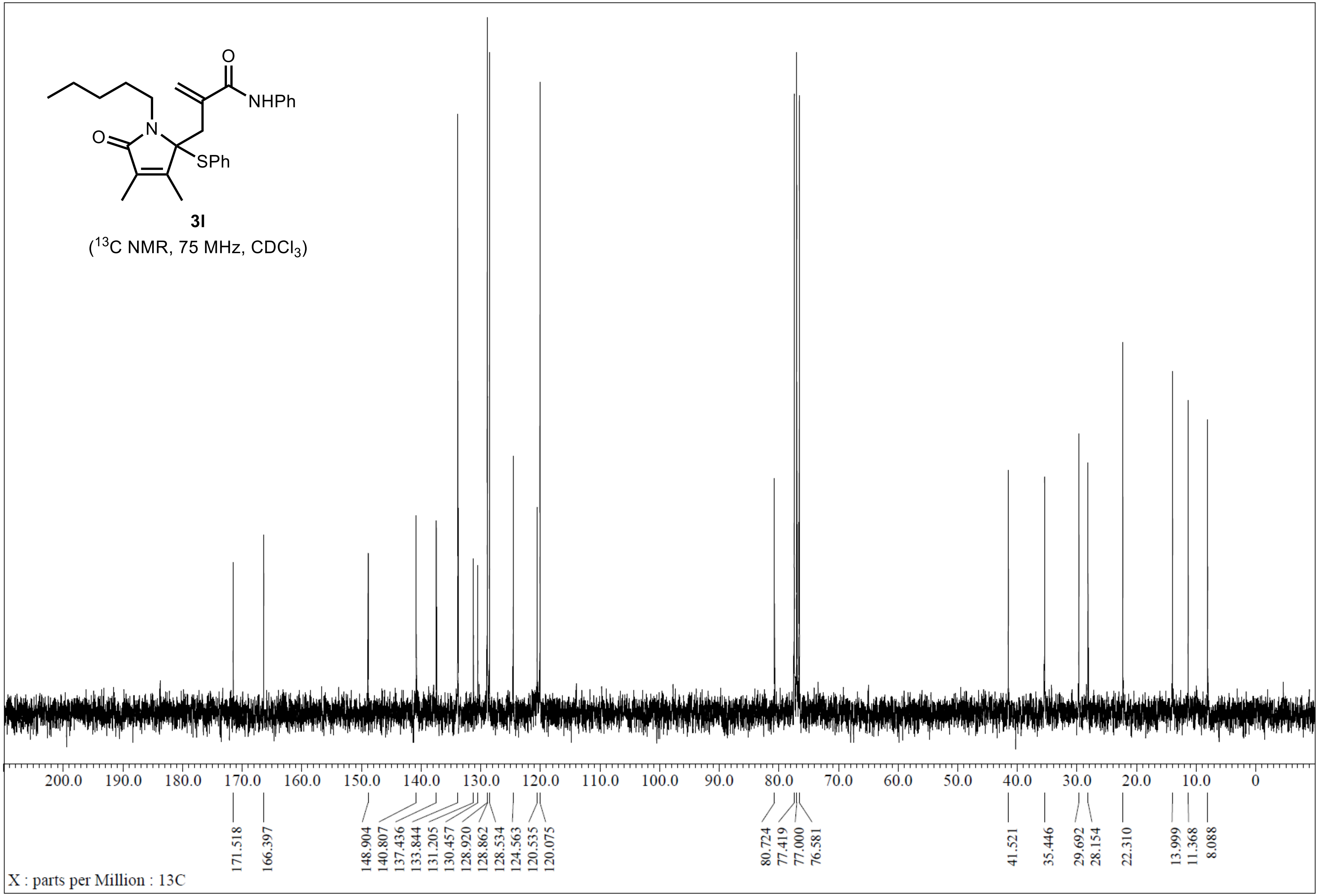

O
NHPh
O
N
SPh
3l
(13C NMR, 75 MHz, CDCl3)
200.0 190.0 180.0 170.0 160.0 150.0 140.0 130.0 120.0 110.0 100.0 90.0 80.0 70.0 60.0 50.0 40.0 30.0 20.0 10.0 0
171.518
166.397
148.904
140.807
137.436
133.844
131.205
130.457
128.920
128.862
128.534
124.563
120.535
120.075
80.724
77.419
77.000
76.581
41.521
35.446
29.692
28.154
22.310
13.999
11.368
8.088
X : parts per Million : 13C

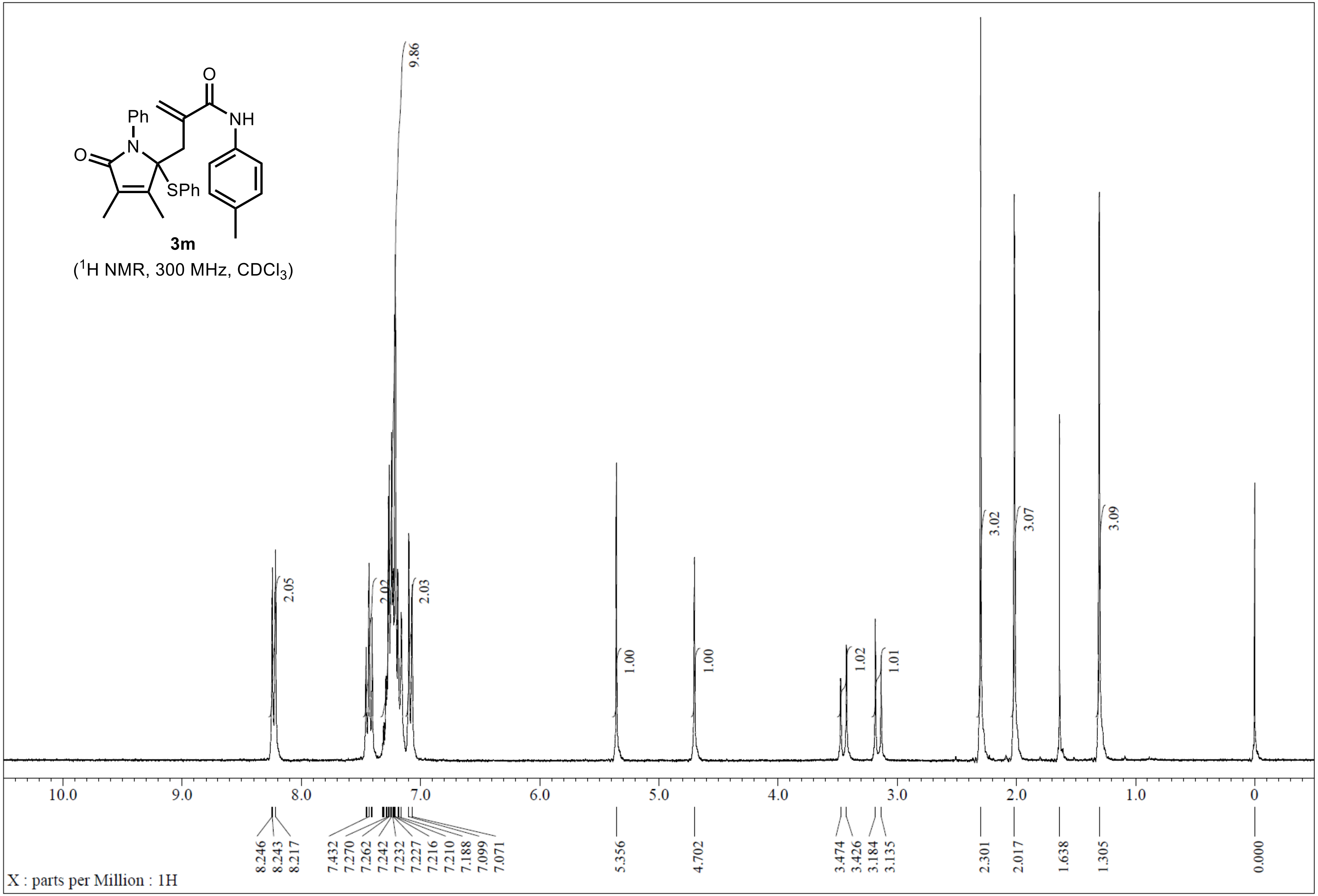
Ph
O
NH
SPh
3m
(^{1}H NMR, 300 MHz, CDCl$_3$)
9.86
2.05
2.02
2.03
1.00
1.00
1.02
1.01
3.02
3.07
3.09
10.0 9.0 8.0 7.0 6.0 5.0 4.0 3.0 2.0 1.0 0
8.246 8.243 8.217
7.432 7.270 7.262 7.242 7.232 7.227 7.216 7.210 7.188 7.099 7.071
5.356
4.702
3.474 3.426 3.184 3.135
2.301
2.017
1.638
1.305
0.000
X : parts per Million : 1H

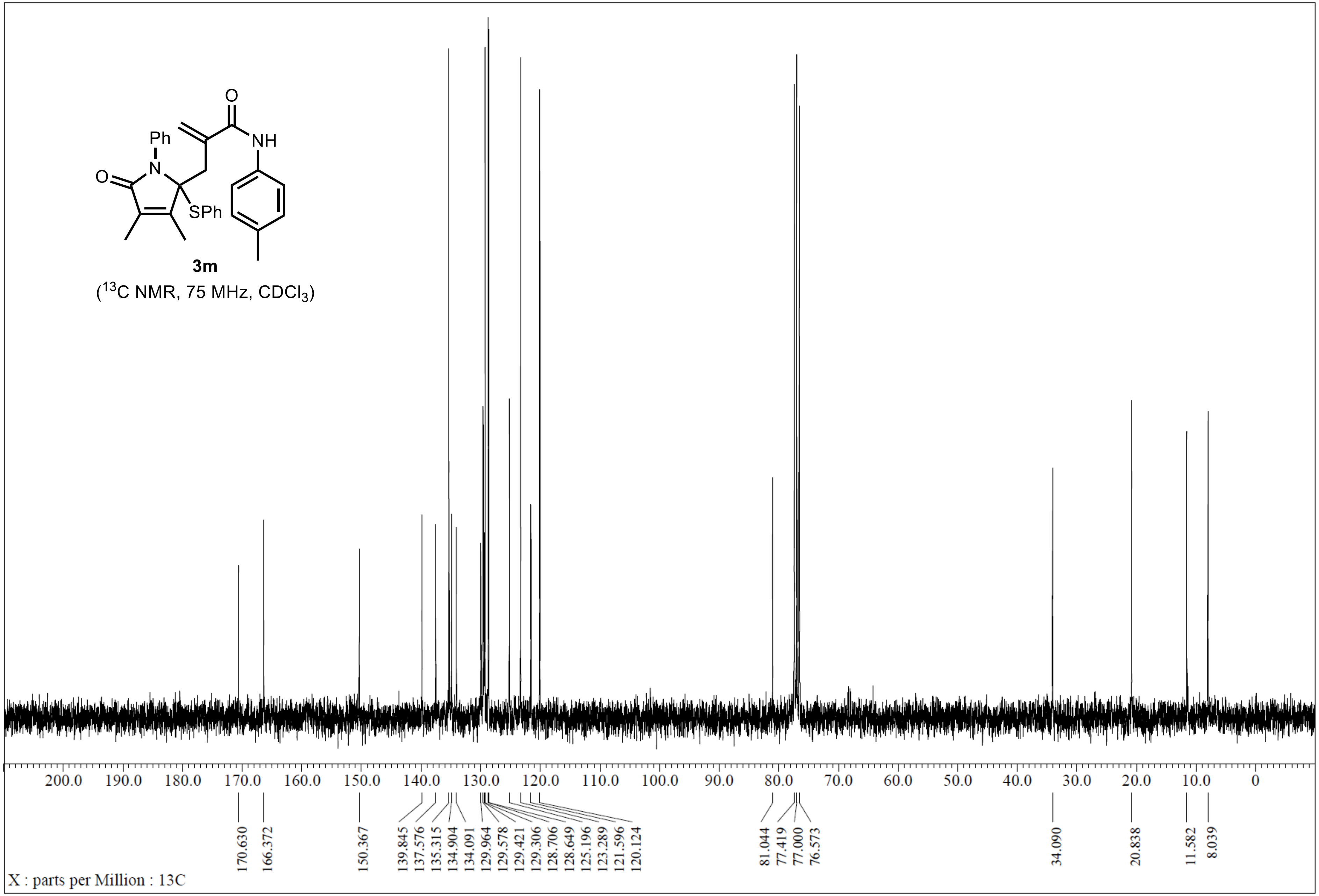
3m
(^{13}C NMR, 75 MHz, CDCl$_3$)
170.630
166.372
150.367
139.845
137.576
135.315
134.904
134.091
129.964
129.578
129.421
129.306
128.706
128.649
125.196
123.289
121.596
120.124
81.044
77.419
77.000
76.573
34.090
20.838
11.582
8.039
X : parts per Million : 13C

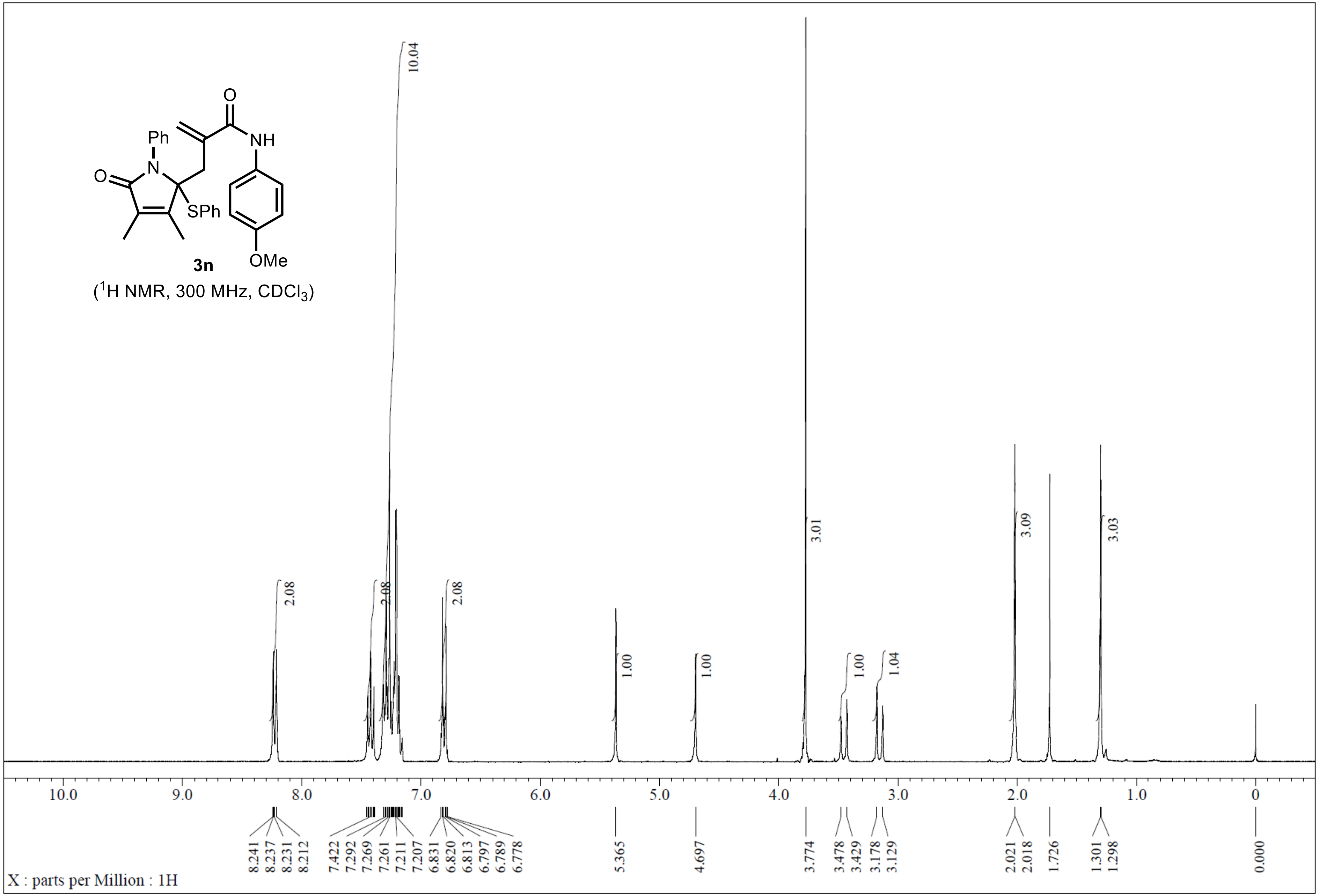
O
Ph
NH
N
O
SPh
OMe
3n
(1H NMR, 300 MHz, CDCl3)
10.04
2.08
2.08
2.08
1.00
1.00
3.01
1.00
1.04
3.09
3.03
10.0
9.0
8.0
7.0
6.0
5.0
4.0
3.0
2.0
1.0
0
8.241
8.237
8.231
8.212
7.422
7.292
7.269
7.261
7.211
7.207
6.831
6.820
6.813
6.797
6.789
6.778
5.365
4.697
3.774
3.478
3.429
3.178
3.129
2.021
2.018
1.726
1.301
1.298
0.000
X : parts per Million : 1H

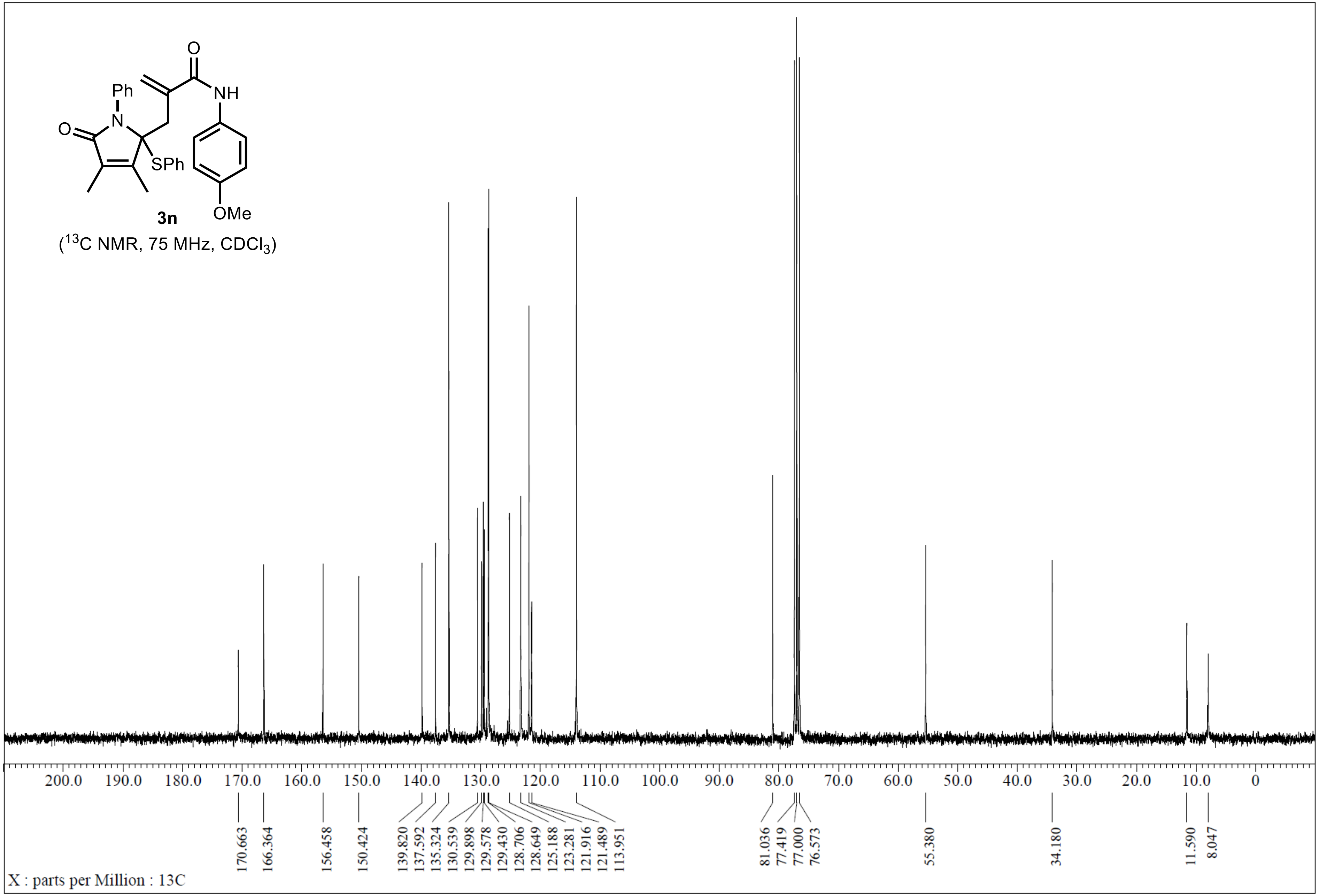
Ph
N
O
O
NH
SPh
OMe
3n
(13C NMR, 75 MHz, CDCl3)
200.0 190.0 180.0 170.0 160.0 150.0 140.0 130.0 120.0 110.0 100.0 90.0 80.0 70.0 60.0 50.0 40.0 30.0 20.0 10.0 0
170.663
166.364
156.458
150.424
139.820
137.592
135.324
130.539
129.898
129.578
129.430
128.706
128.649
125.188
123.281
121.916
121.489
113.951
81.036
77.419
77.000
76.573
55.380
34.180
11.590
8.047
X : parts per Million : 13C

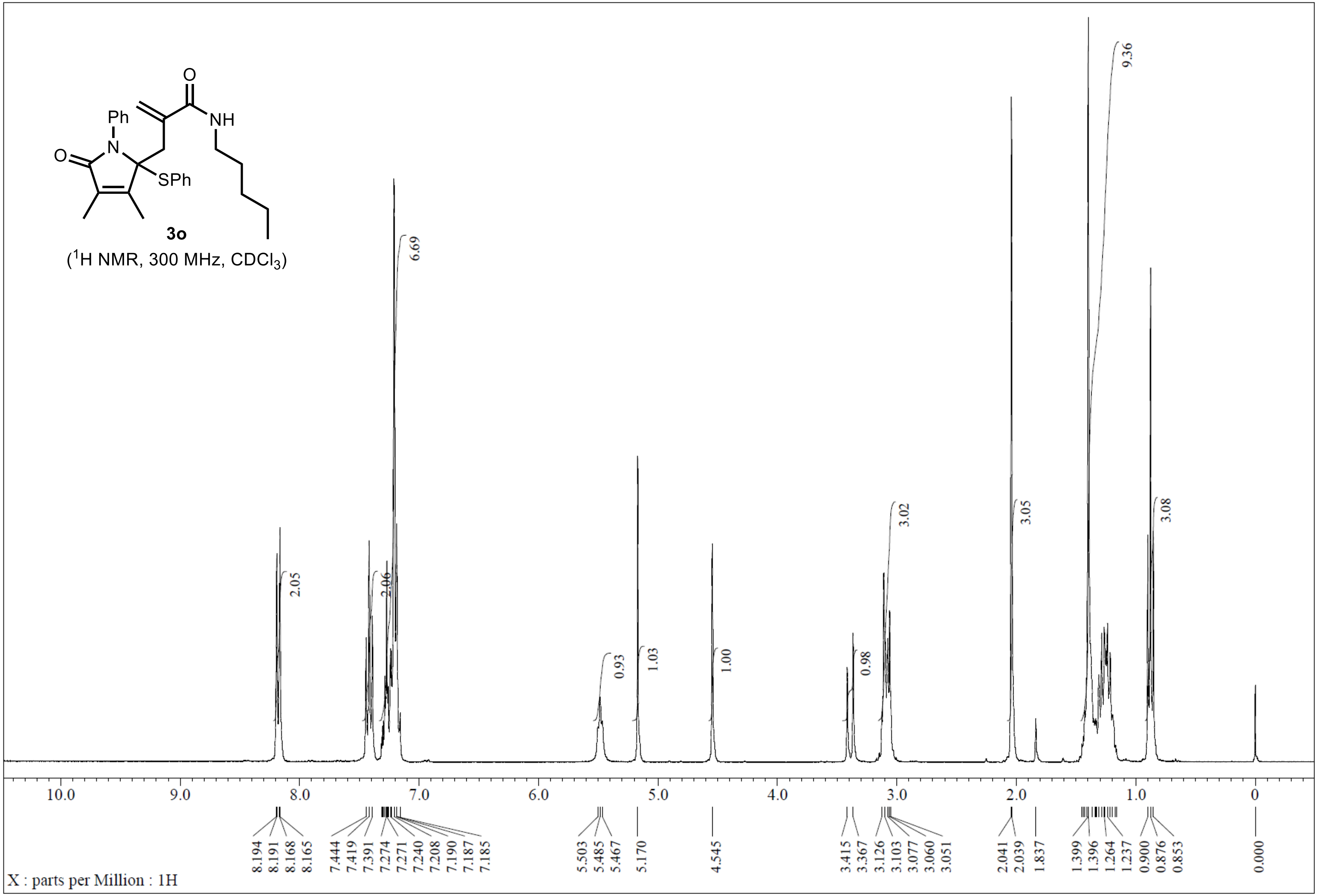
O
Ph
NH
N
O
SPh
3o
(1H NMR, 300 MHz, CDCl3)
9.36
6.69
2.05
2.06
0.93
1.03
1.00
0.98
3.02
3.05
3.08
10.0
9.0
8.0
7.0
6.0
5.0
4.0
3.0
2.0
1.0
0
8.194
8.191
8.168
8.165
7.444
7.419
7.391
7.274
7.271
7.240
7.208
7.190
7.187
7.185
5.503
5.485
5.467
5.170
4.545
3.415
3.367
3.126
3.103
3.077
3.060
3.051
2.041
2.039
1.837
1.399
1.396
1.264
1.237
0.900
0.876
0.853
0.000
X : parts per Million : 1H

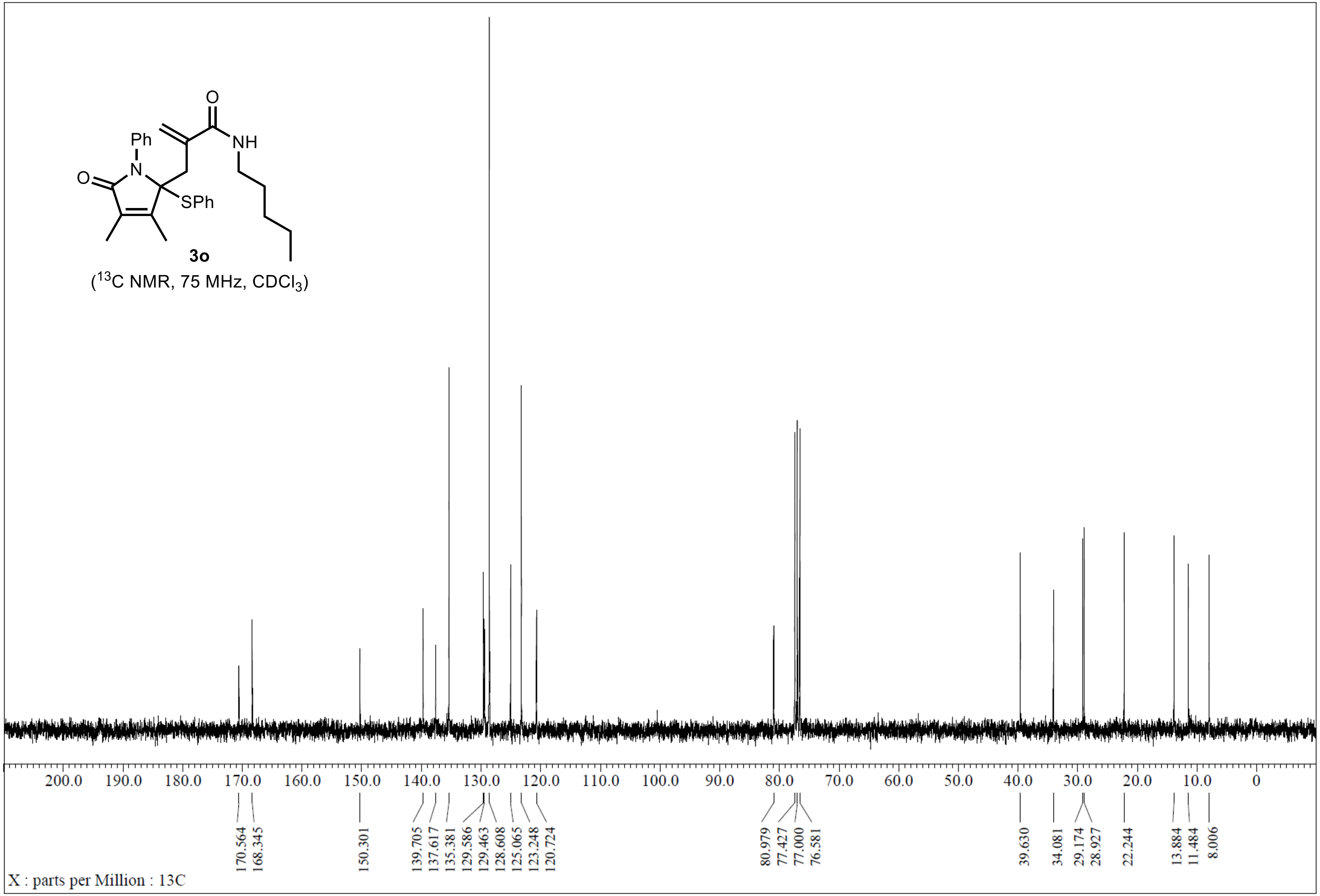
O
Ph
N
NH
O
SPh
3o
(13C NMR, 75 MHz, CDCl3)
200.0 190.0 180.0 170.0 160.0 150.0 140.0 130.0 120.0 110.0 100.0 90.0 80.0 70.0 60.0 50.0 40.0 30.0 20.0 10.0 0
170.564
168.345
150.301
139.705
137.617
135.381
129.586
129.463
128.608
125.065
123.248
120.724
80.979
77.427
77.000
76.581
39.630
34.081
29.174
28.927
22.244
13.884
11.484
8.006
X : parts per Million : 13C

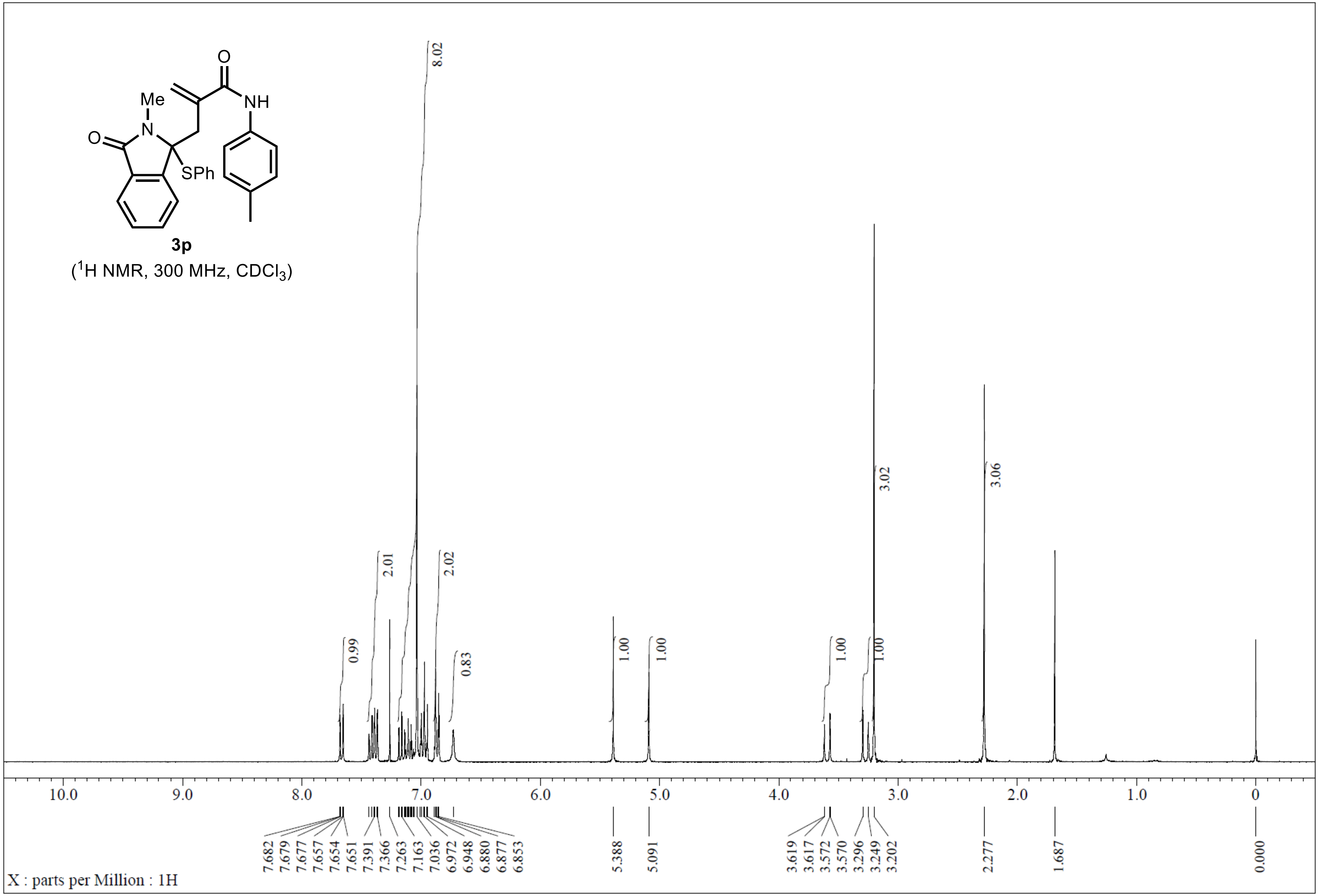

O
Me
N
NH
O
SPh
3p
(¹H NMR, 300 MHz, CDCl₃)
8.02
2.01
2.02
0.99
0.83
1.00
1.00
1.00
1.00
3.02
3.06
10.0
9.0
8.0
7.0
6.0
5.0
4.0
3.0
2.0
1.0
0
7.682
7.679
7.677
7.657
7.654
7.651
7.391
7.366
7.263
7.163
7.036
6.972
6.948
6.880
6.877
6.853
5.388
5.091
3.619
3.617
3.572
3.570
3.296
3.249
3.202
2.277
1.687
0.000
X : parts per Million : 1H

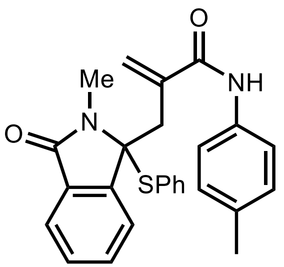

3p

(^{13}C NMR, 75 MHz, $CDCl_3$)

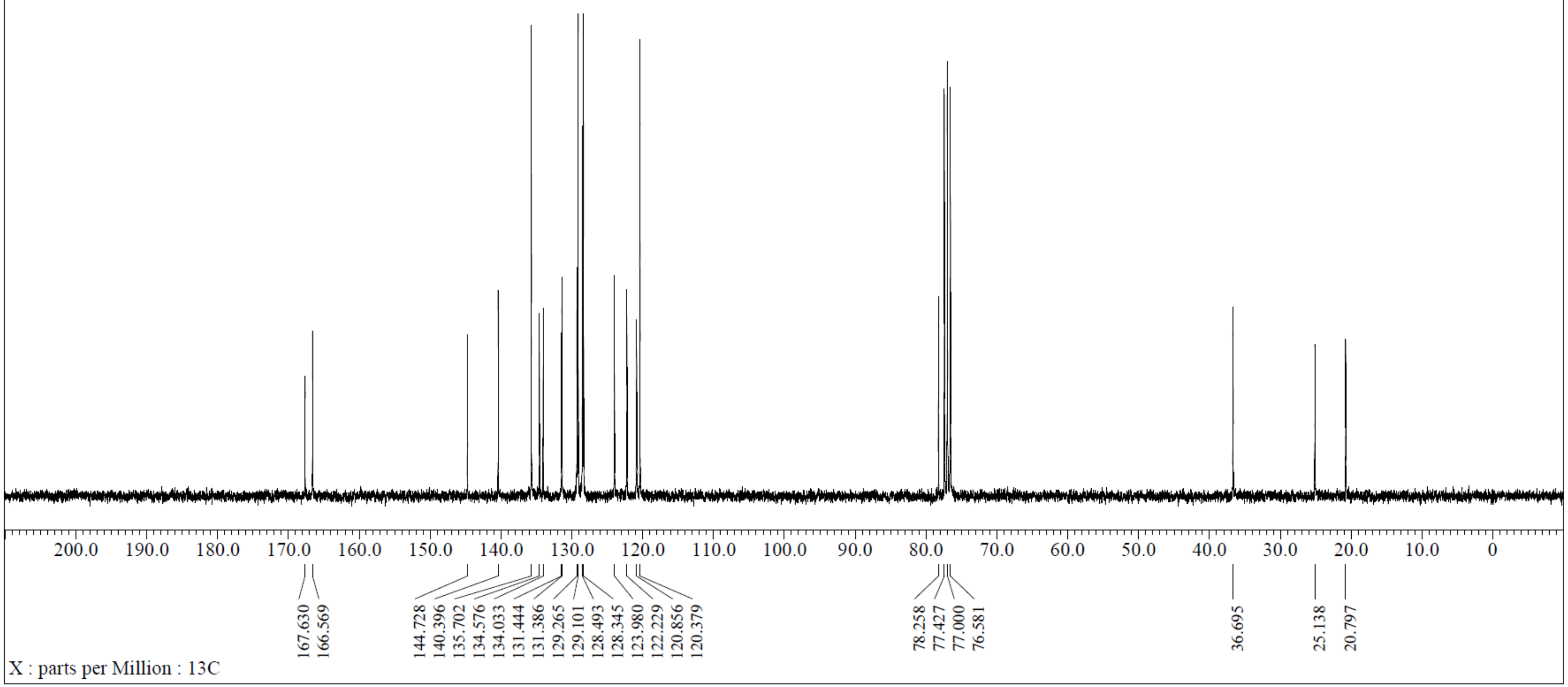

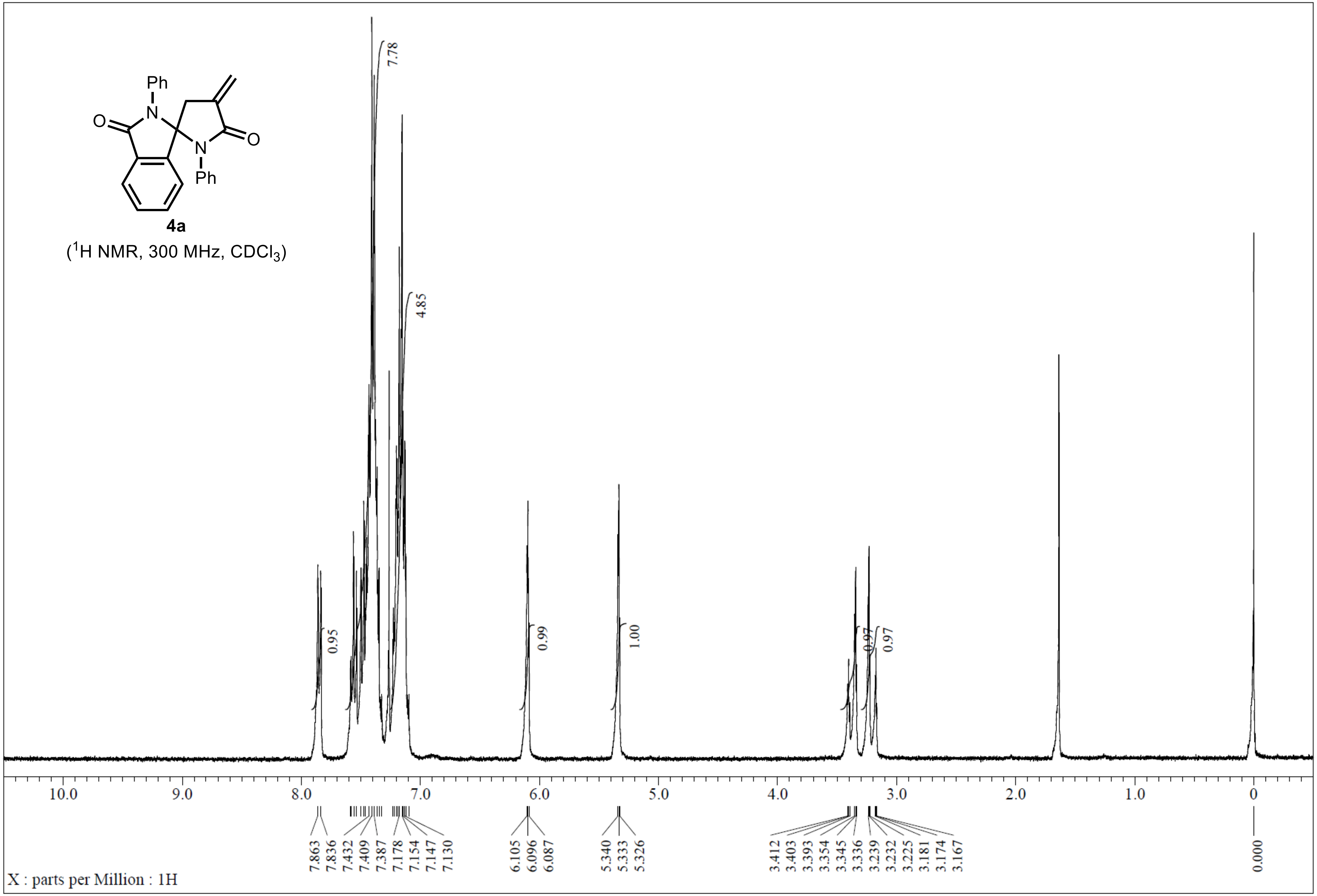

Ph
O
N
N
O
Ph
4a
(¹H NMR, 300 MHz, CDCl₃)
7.78
4.85
0.95
0.99
1.00
0.97
0.97
10.0
9.0
8.0
7.0
6.0
5.0
4.0
3.0
2.0
1.0
0
7.863
7.836
7.432
7.409
7.387
7.178
7.154
7.147
7.130
6.105
6.096
6.087
5.340
5.333
5.326
3.412
3.403
3.393
3.354
3.345
3.336
3.239
3.232
3.225
3.181
3.174
3.167
0.000
X : parts per Million : 1H

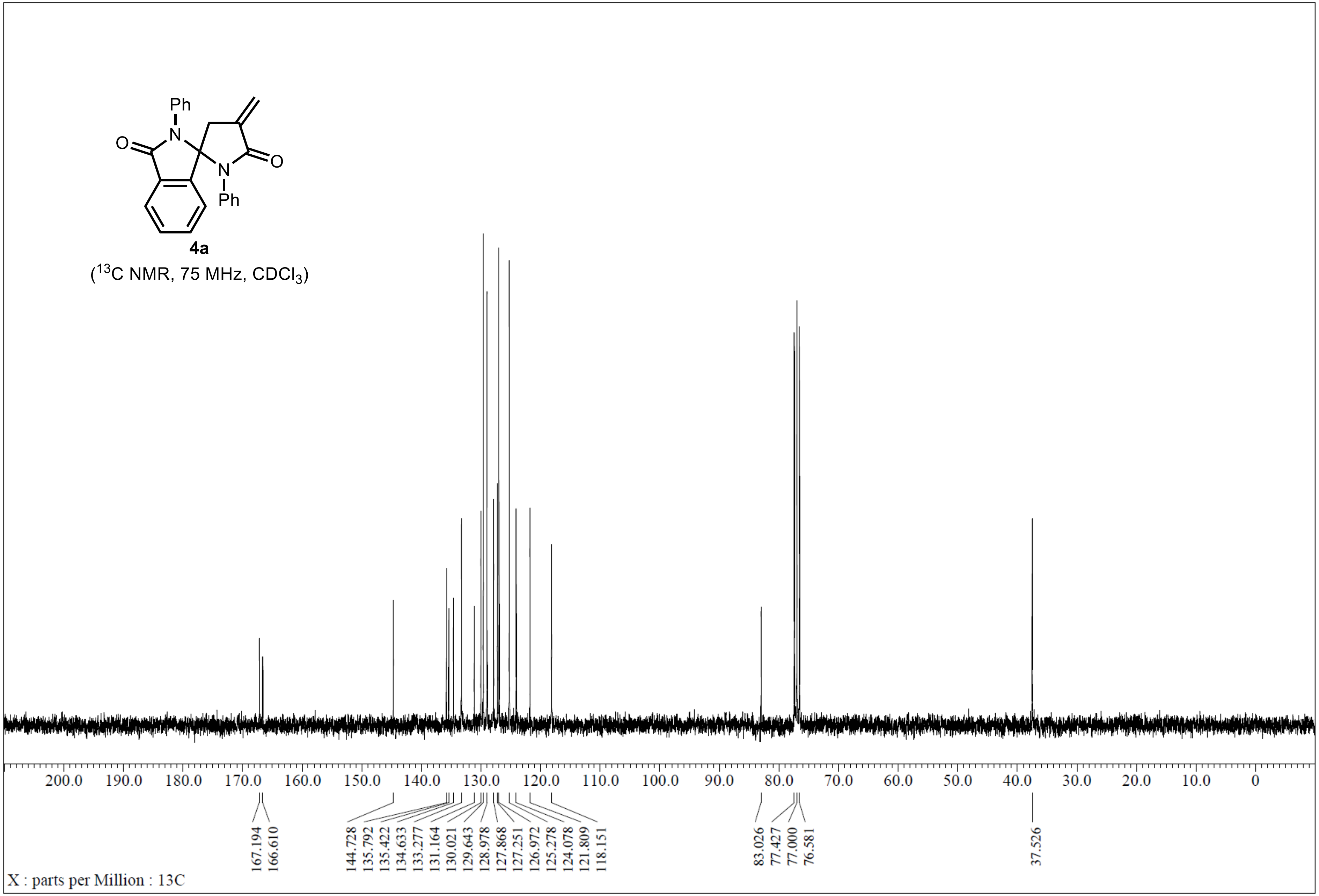
Ph
N
O
N
O
Ph
4a
(13C NMR, 75 MHz, CDCl3)
200.0 190.0 180.0 170.0 160.0 150.0 140.0 130.0 120.0 110.0 100.0 90.0 80.0 70.0 60.0 50.0 40.0 30.0 20.0 10.0 0
167.194
166.610
144.728
135.792
135.422
134.633
133.277
131.164
130.021
129.643
128.978
127.868
127.251
126.972
125.278
124.078
121.809
118.151
83.026
77.427
77.000
76.581
37.526
X : parts per Million : 13C

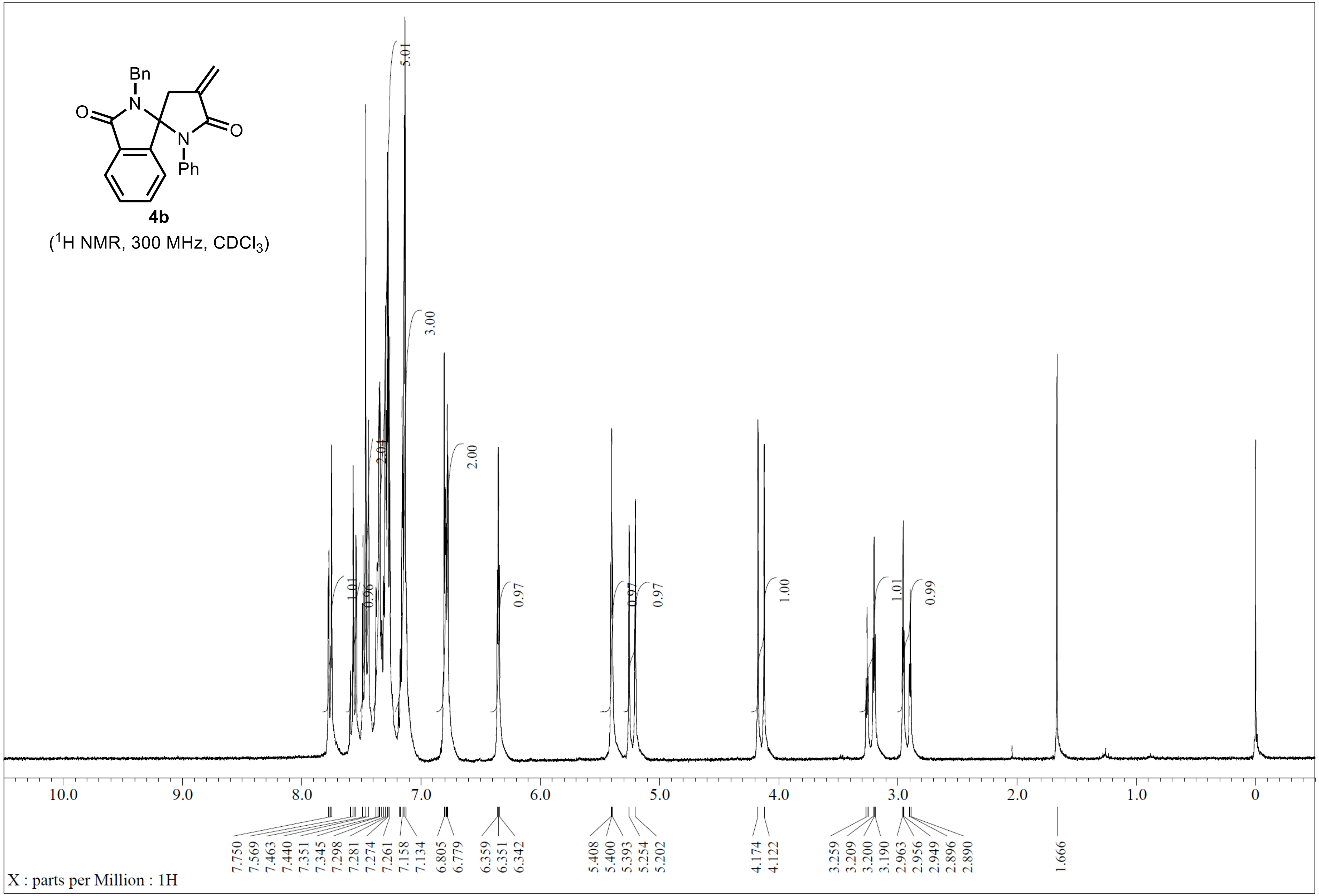
Bn
N
O
N
O
Ph
4b
(1H NMR, 300 MHz, CDCl3)
X : parts per Million : 1H

4b

(^{13}C NMR, 75 MHz, $CDCl_3$)

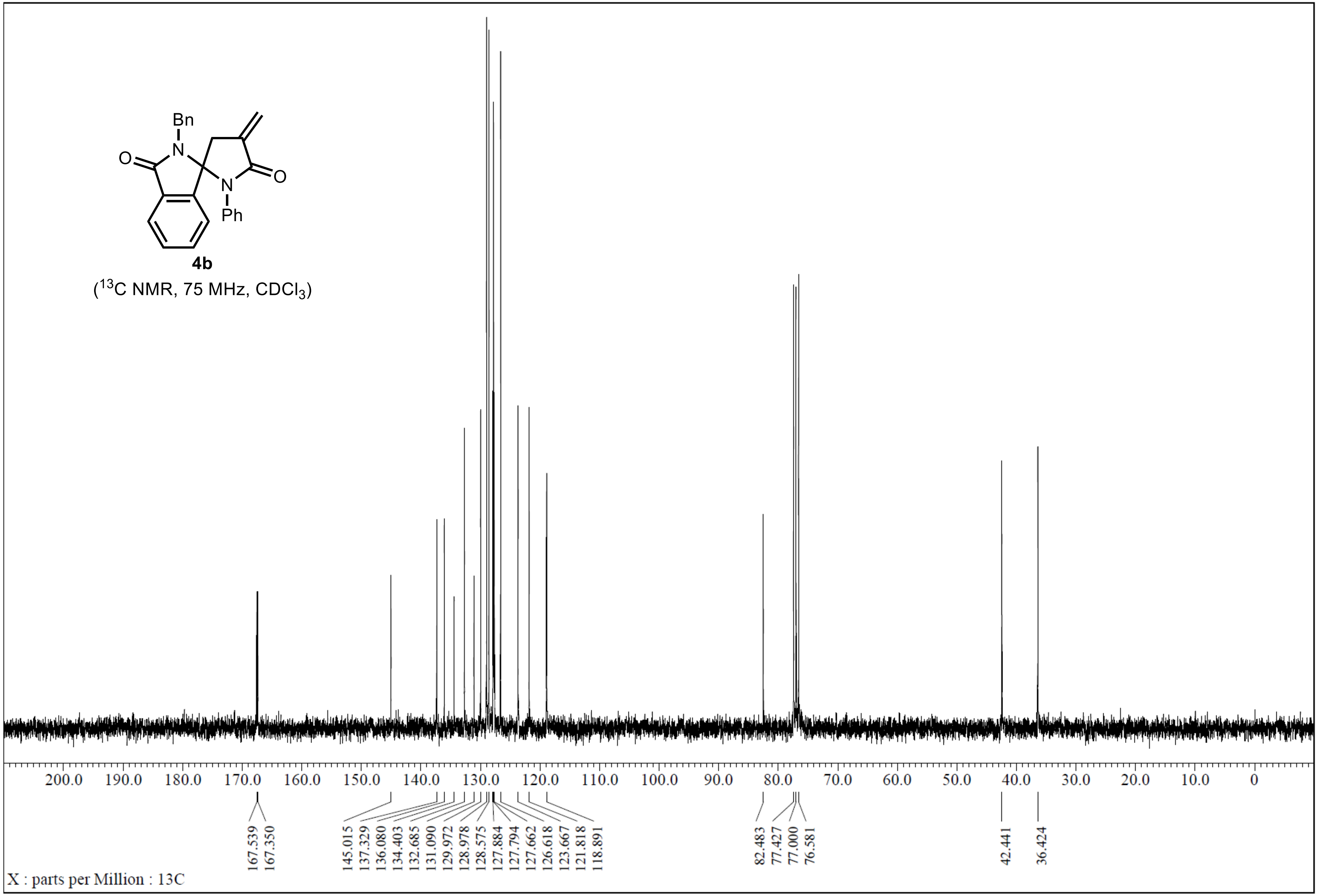

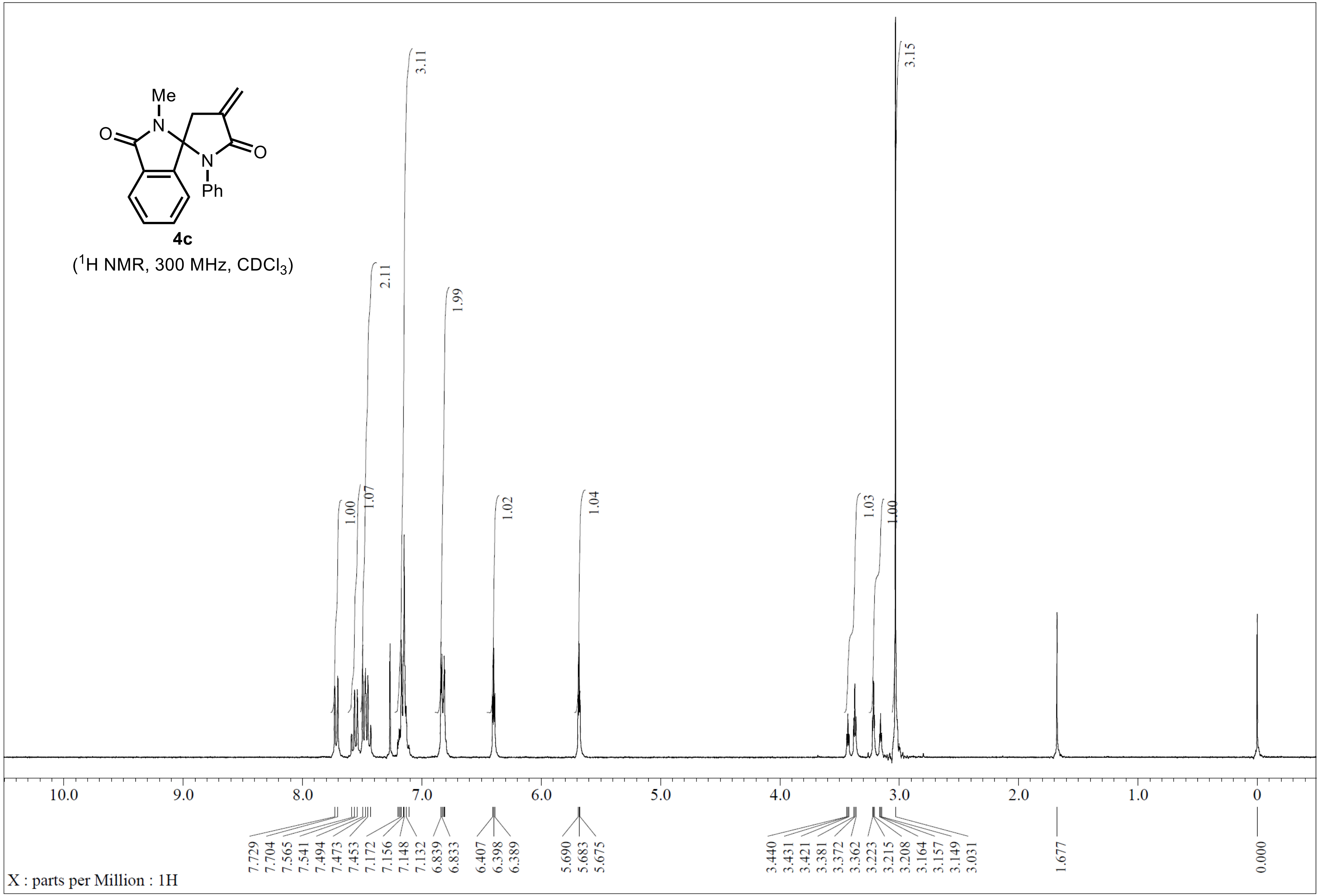
Me
N
O
N
O
Ph
4c
(1H NMR, 300 MHz, CDCl3)
1.00
1.07
2.11
3.11
1.99
1.02
1.04
1.03
1.00
3.15
10.0
9.0
8.0
7.0
6.0
5.0
4.0
3.0
2.0
1.0
0
7.729
7.704
7.565
7.541
7.494
7.473
7.453
7.172
7.156
7.148
7.132
6.839
6.833
6.407
6.398
6.389
5.690
5.683
5.675
3.440
3.431
3.421
3.381
3.372
3.362
3.223
3.215
3.208
3.164
3.157
3.149
3.031
1.677
0.000
X : parts per Million : 1H

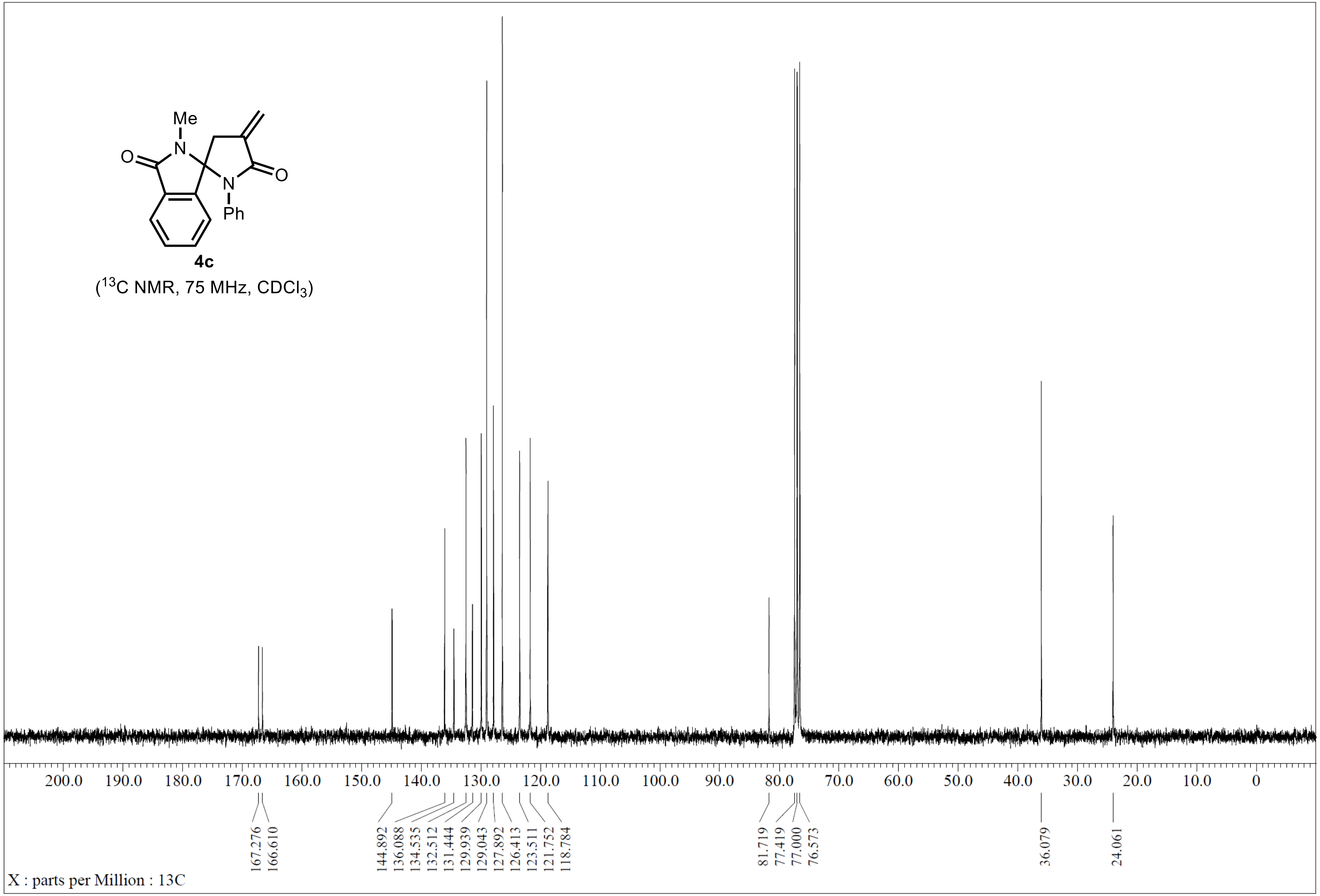
Me
N
O
N
O
Ph
4c
(13C NMR, 75 MHz, CDCl3)
200.0
190.0
180.0
170.0
160.0
150.0
140.0
130.0
120.0
110.0
100.0
90.0
80.0
70.0
60.0
50.0
40.0
30.0
20.0
10.0
0
167.276
166.610
144.892
136.088
134.535
132.512
131.444
129.939
129.043
127.892
126.413
123.511
121.752
118.784
81.719
77.419
77.000
76.573
36.079
24.061
X : parts per Million : 13C

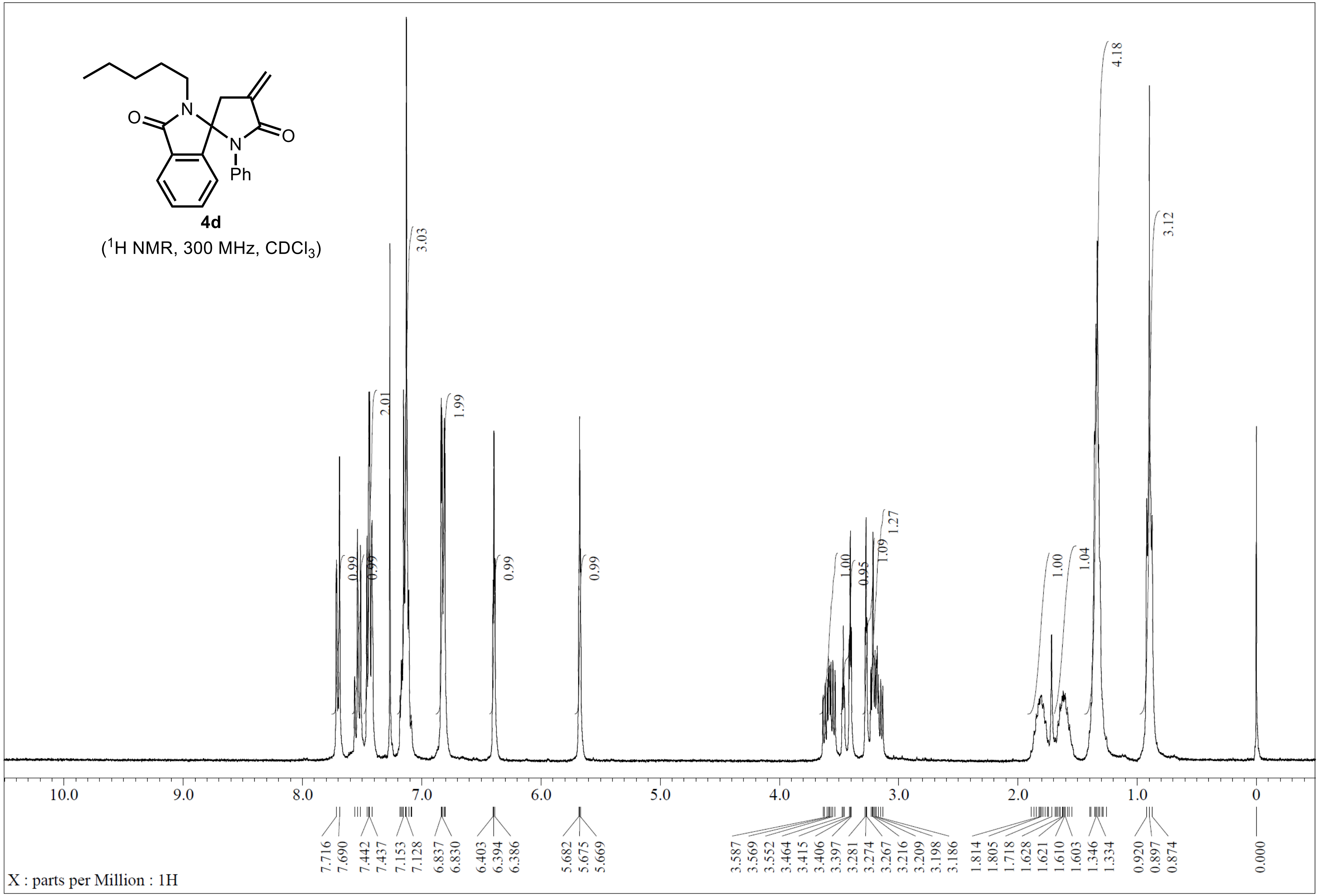
4d
(1H NMR, 300 MHz, CDCl3)
0.99 0.99 2.01 3.03 1.99 0.99 0.99 1.00 0.95 1.09 1.27 1.00 1.04 4.18 3.12
10.0 9.0 8.0 7.0 6.0 5.0 4.0 3.0 2.0 1.0 0
7.716 7.690 7.442 7.437 7.153 7.128 6.837 6.830 6.403 6.394 6.386 5.682 5.675 5.669
3.587 3.569 3.552 3.464 3.415 3.406 3.397 3.281 3.274 3.267 3.216 3.209 3.198 3.186
1.814 1.805 1.718 1.628 1.621 1.610 1.603 1.346 1.334 0.920 0.897 0.874 0.000
X : parts per Million : 1H

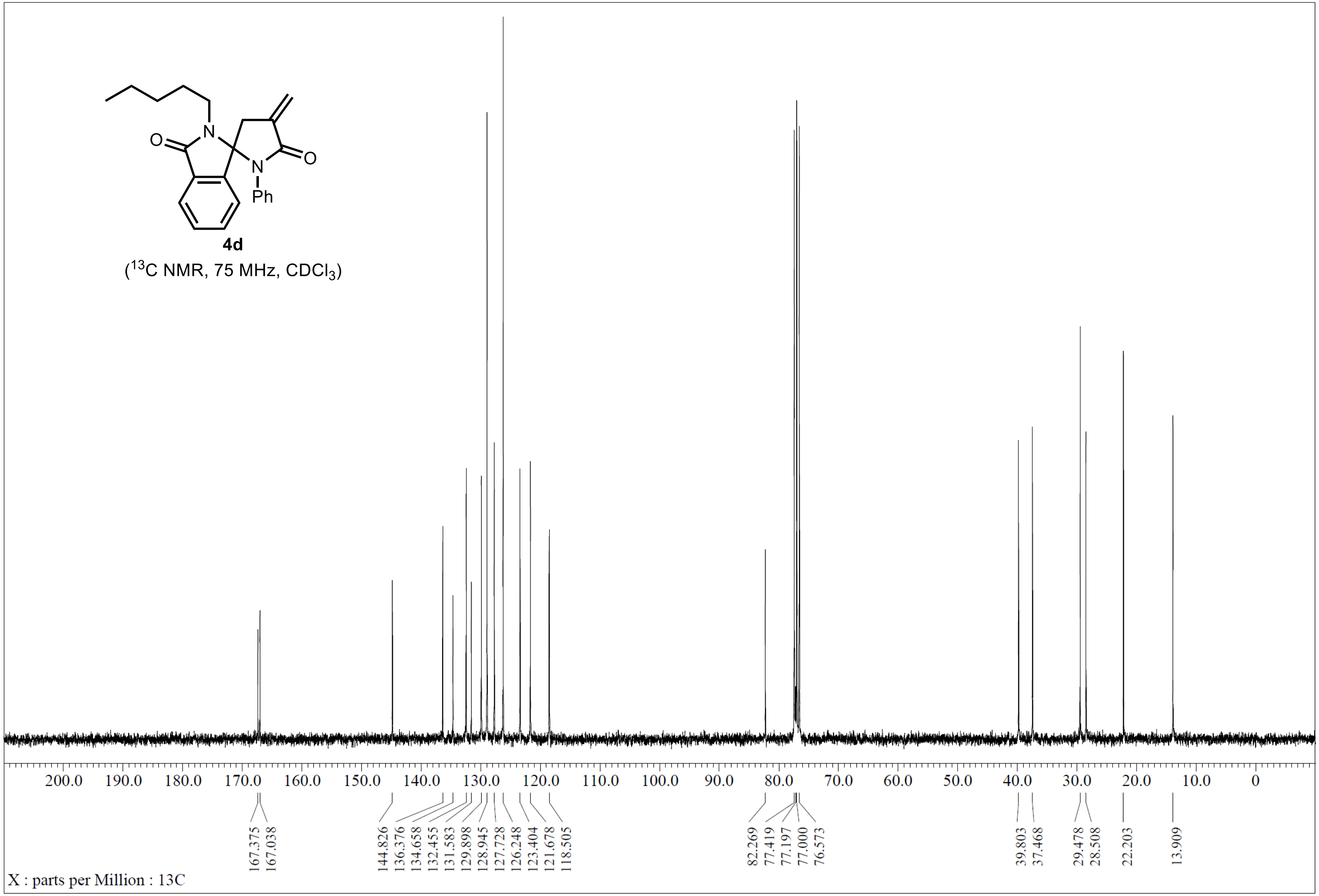
O
N
N
Ph
O
4d
(¹³C NMR, 75 MHz, CDCl₃)
200.0
190.0
180.0
170.0
160.0
150.0
140.0
130.0
120.0
110.0
100.0
90.0
80.0
70.0
60.0
50.0
40.0
30.0
20.0
10.0
0
167.375
167.038
144.826
136.376
134.658
132.455
131.583
129.898
128.945
127.728
126.248
123.404
121.678
118.505
82.269
77.419
77.197
77.000
76.573
39.803
37.468
29.478
28.508
22.203
13.909
X : parts per Million : 13C

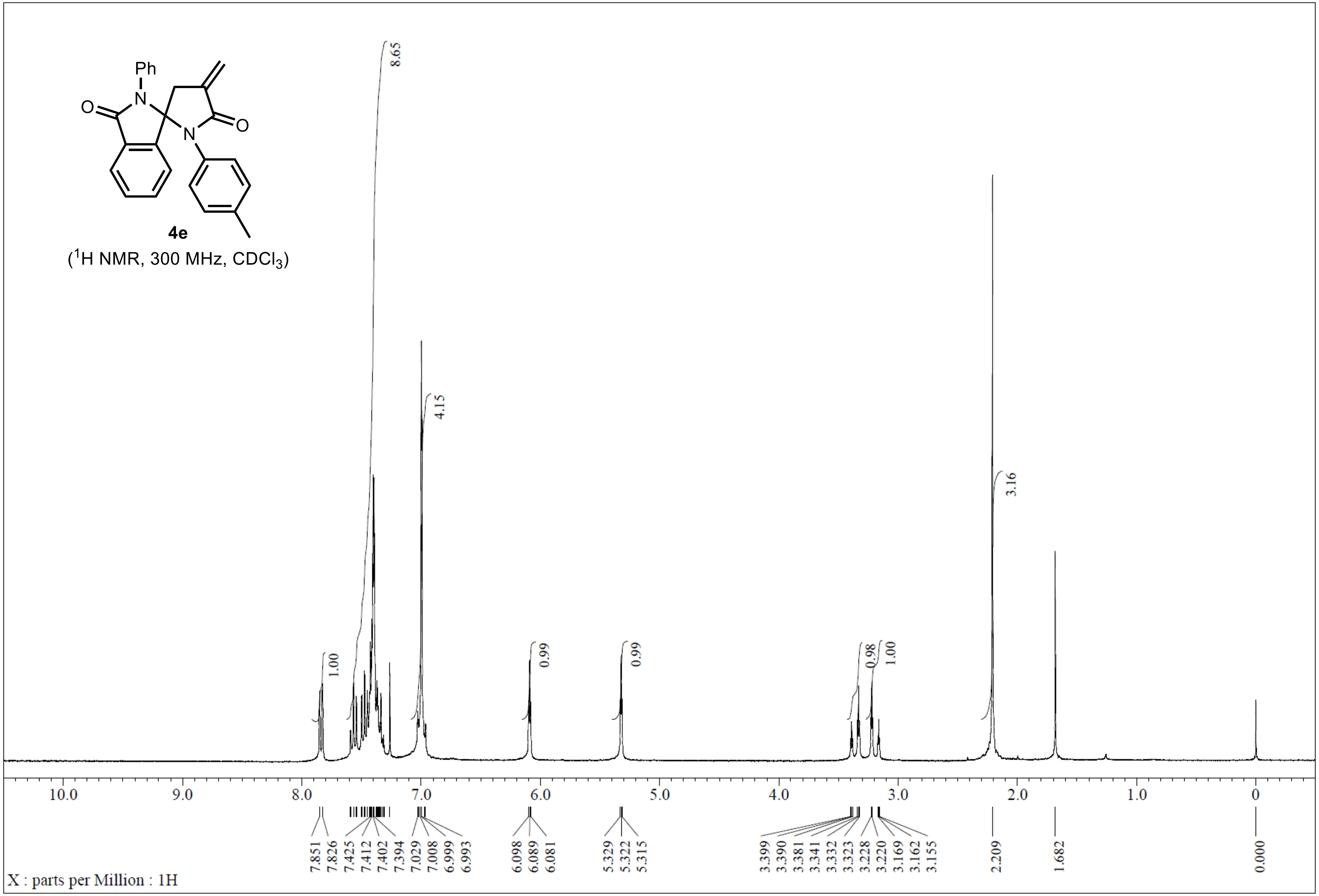
Ph
N
O
N
O
4e
(1H NMR, 300 MHz, CDCl3)
8.65
4.15
3.16
1.00
0.99
0.99
0.98
1.00
10.0
9.0
8.0
7.0
6.0
5.0
4.0
3.0
2.0
1.0
0
7.851
7.826
7.425
7.412
7.402
7.394
7.029
7.008
6.999
6.993
6.098
6.089
6.081
5.329
5.322
5.315
3.399
3.390
3.381
3.341
3.332
3.323
3.228
3.220
3.169
3.162
3.155
2.209
1.682
0.000
X : parts per Million : 1H

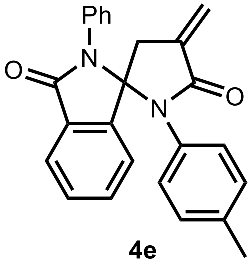

4e

(^{13}C NMR, 75 MHz, $CDCl_3$)

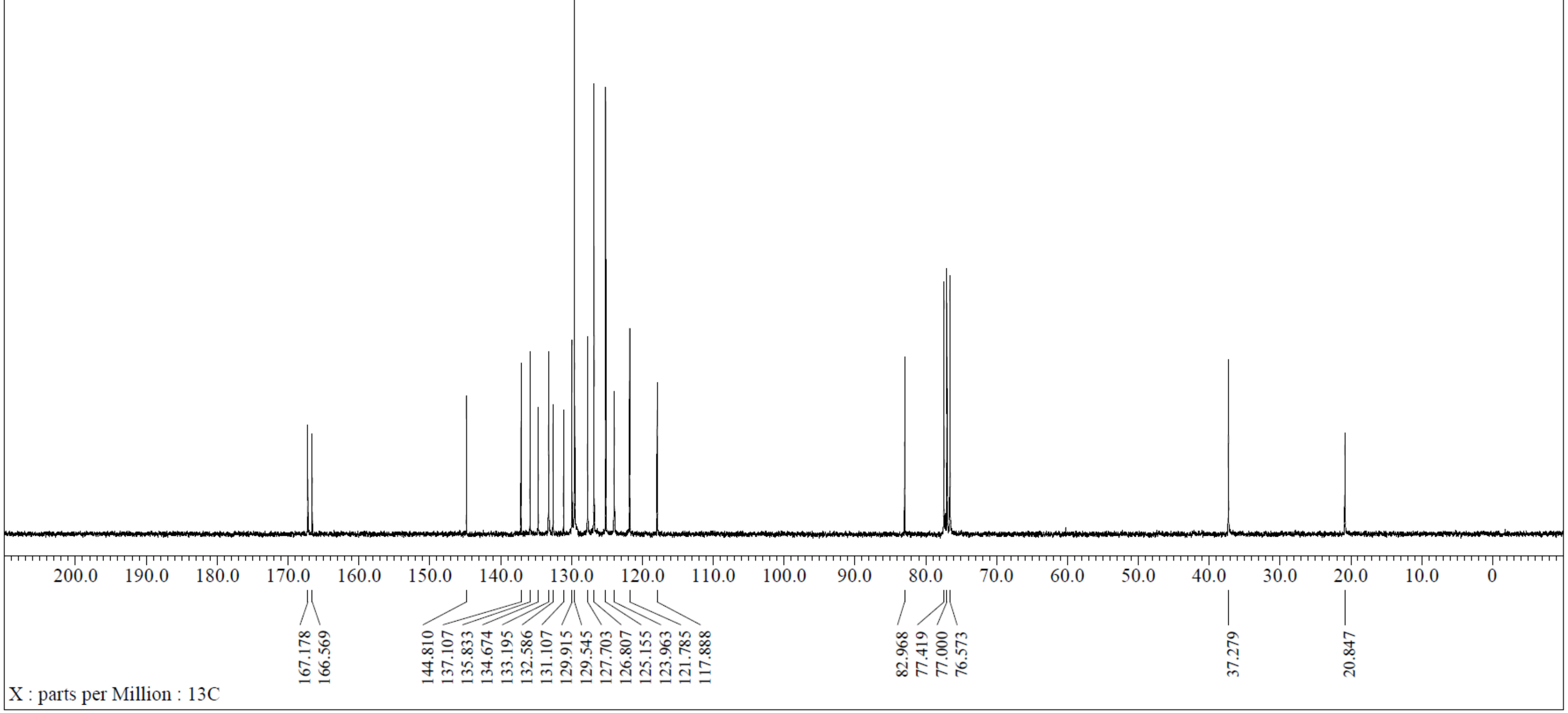

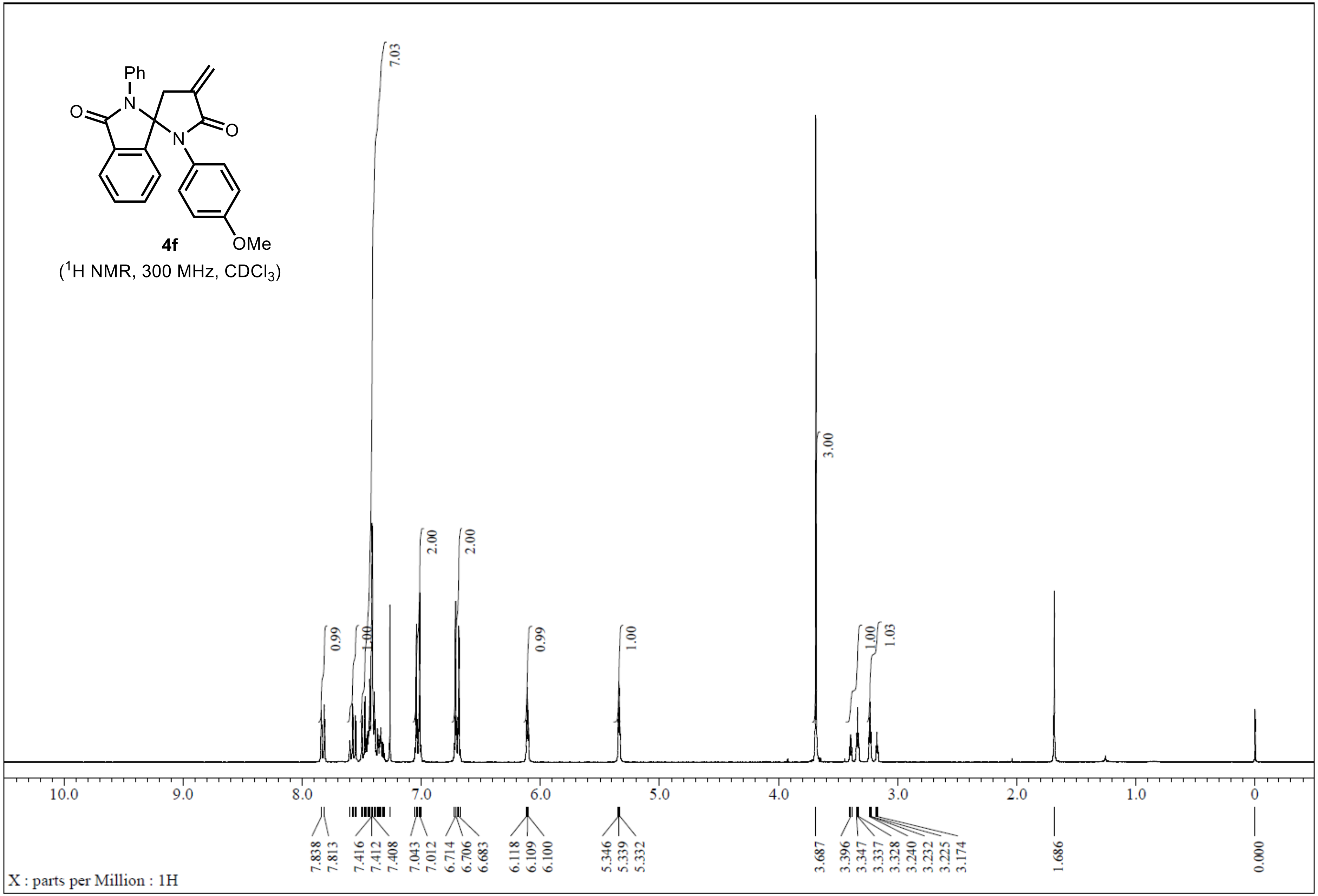
Ph
O
N
N
O
OMe
4f
(1H NMR, 300 MHz, CDCl3)
7.03
3.00
0.99
1.00
2.00
2.00
0.99
1.00
1.00
1.03
10.0
9.0
8.0
7.0
6.0
5.0
4.0
3.0
2.0
1.0
0
7.838
7.813
7.416
7.412
7.408
7.043
7.012
6.714
6.706
6.683
6.118
6.109
6.100
5.346
5.339
5.332
3.687
3.396
3.347
3.337
3.328
3.240
3.232
3.225
3.174
1.686
0.000
X : parts per Million : 1H

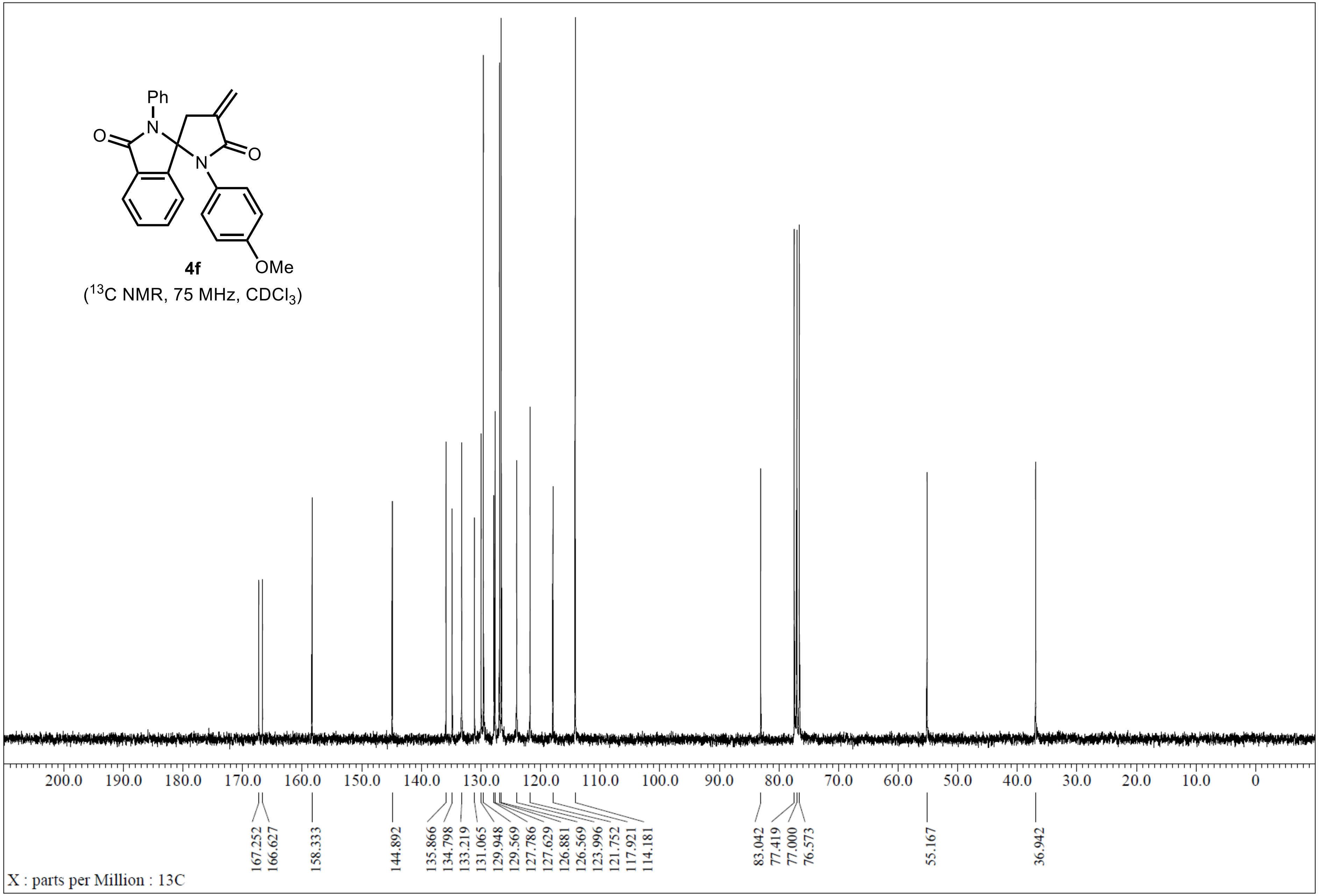
Ph
N
O
N
O
OMe
4f
(13C NMR, 75 MHz, CDCl3)
200.0 190.0 180.0 170.0 160.0 150.0 140.0 130.0 120.0 110.0 100.0 90.0 80.0 70.0 60.0 50.0 40.0 30.0 20.0 10.0 0
167.252
166.627
158.333
144.892
135.866
134.798
133.219
131.065
129.948
129.569
127.786
127.629
126.881
126.569
123.996
121.752
117.921
114.181
83.042
77.419
77.000
76.573
55.167
36.942
X : parts per Million : 13C

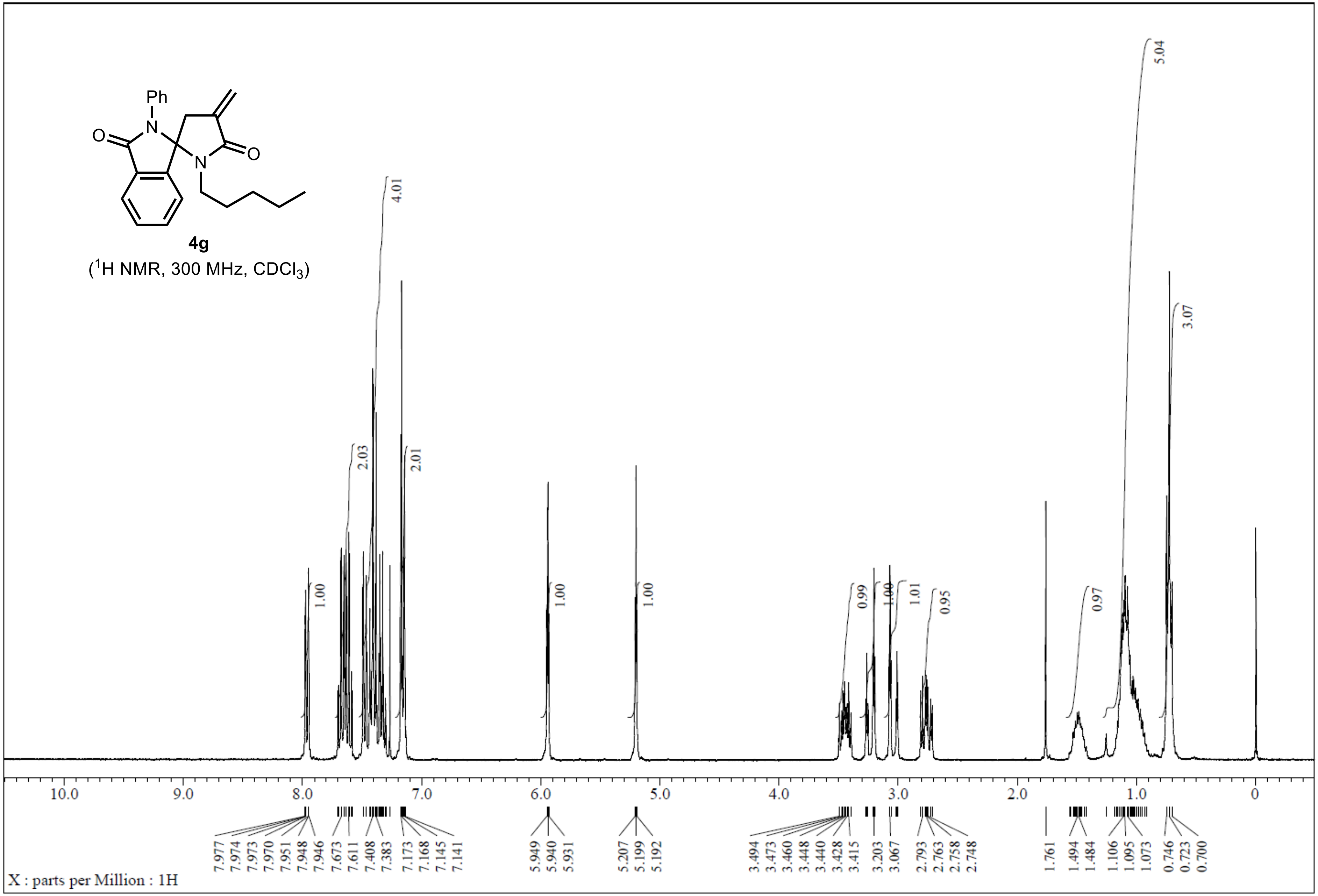
Ph
N
O
N
O
4g
(¹H NMR, 300 MHz, CDCl3)
1.00
2.03
4.01
2.01
1.00
1.00
0.99
1.00
1.01
0.95
0.97
5.04
3.07
10.0
9.0
8.0
7.0
6.0
5.0
4.0
3.0
2.0
1.0
0
7.977
7.974
7.973
7.970
7.951
7.948
7.946
7.673
7.611
7.408
7.383
7.173
7.168
7.145
7.141
5.949
5.940
5.931
5.207
5.199
5.192
3.494
3.473
3.460
3.448
3.440
3.428
3.415
3.203
3.067
2.793
2.763
2.758
2.748
1.761
1.494
1.484
1.106
1.095
1.073
0.746
0.723
0.700
X : parts per Million : 1H

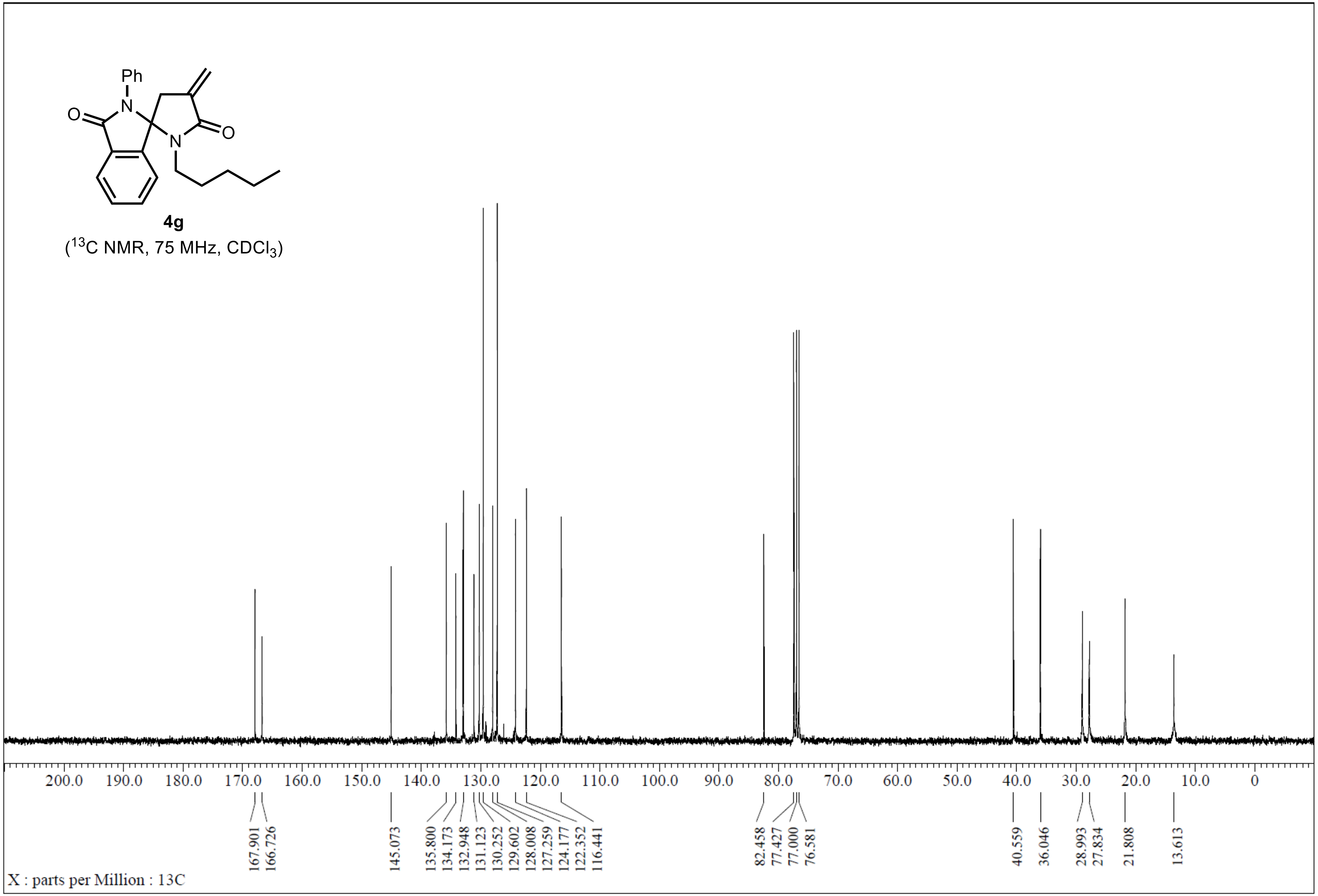

4g

(^{13}C NMR, 75 MHz, $CDCl_3$)

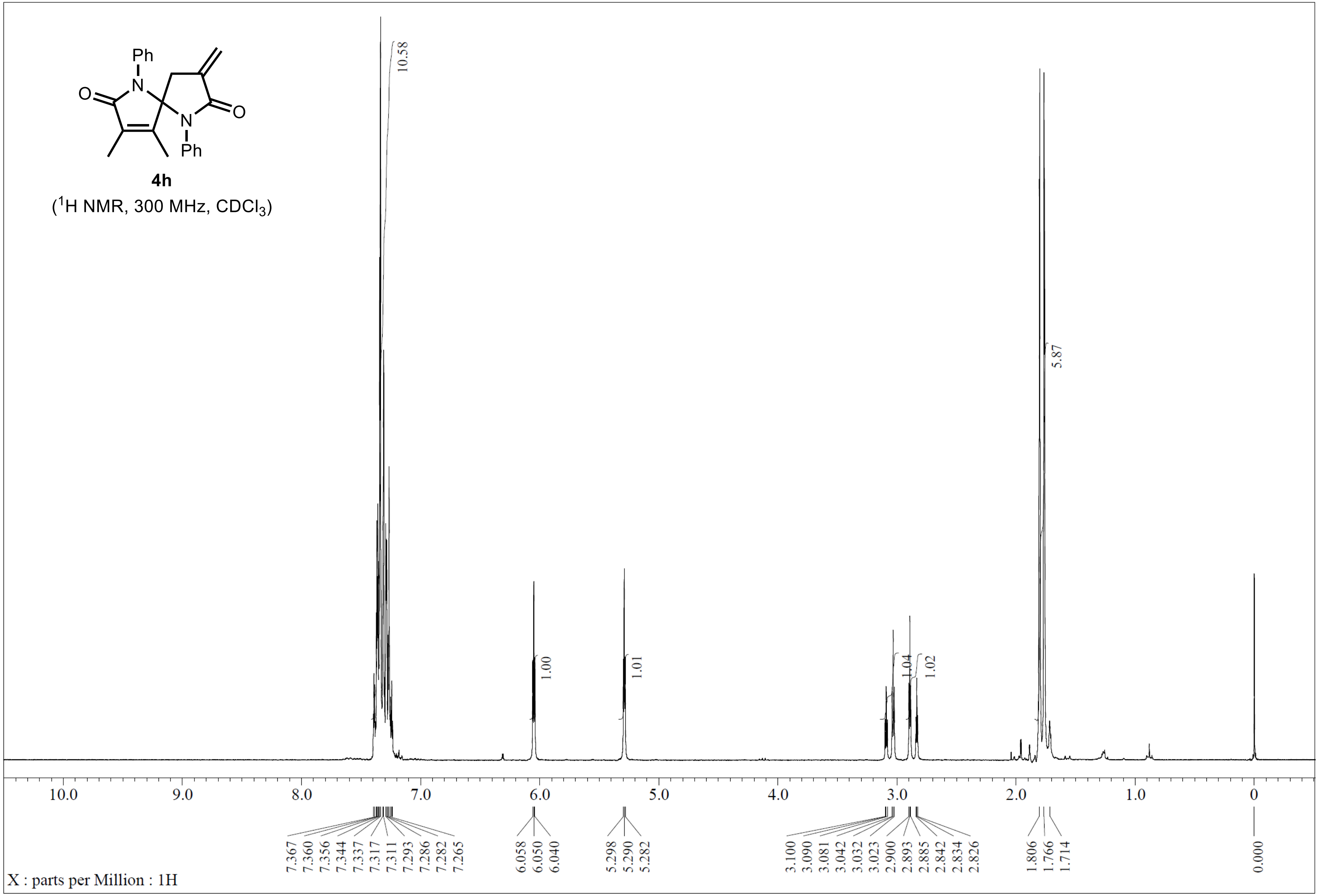
Ph
O
N
N
O
Ph
4h
(1H NMR, 300 MHz, CDCl3)
10.58
5.87
1.00
1.01
1.04
1.02
10.0
9.0
8.0
7.0
6.0
5.0
4.0
3.0
2.0
1.0
0
7.367
7.360
7.356
7.344
7.337
7.317
7.311
7.293
7.286
7.282
7.265
6.058
6.050
6.040
5.298
5.290
5.282
3.100
3.090
3.081
3.042
3.032
3.023
2.900
2.893
2.885
2.842
2.834
2.826
1.806
1.766
1.714
0.000
X : parts per Million : 1H

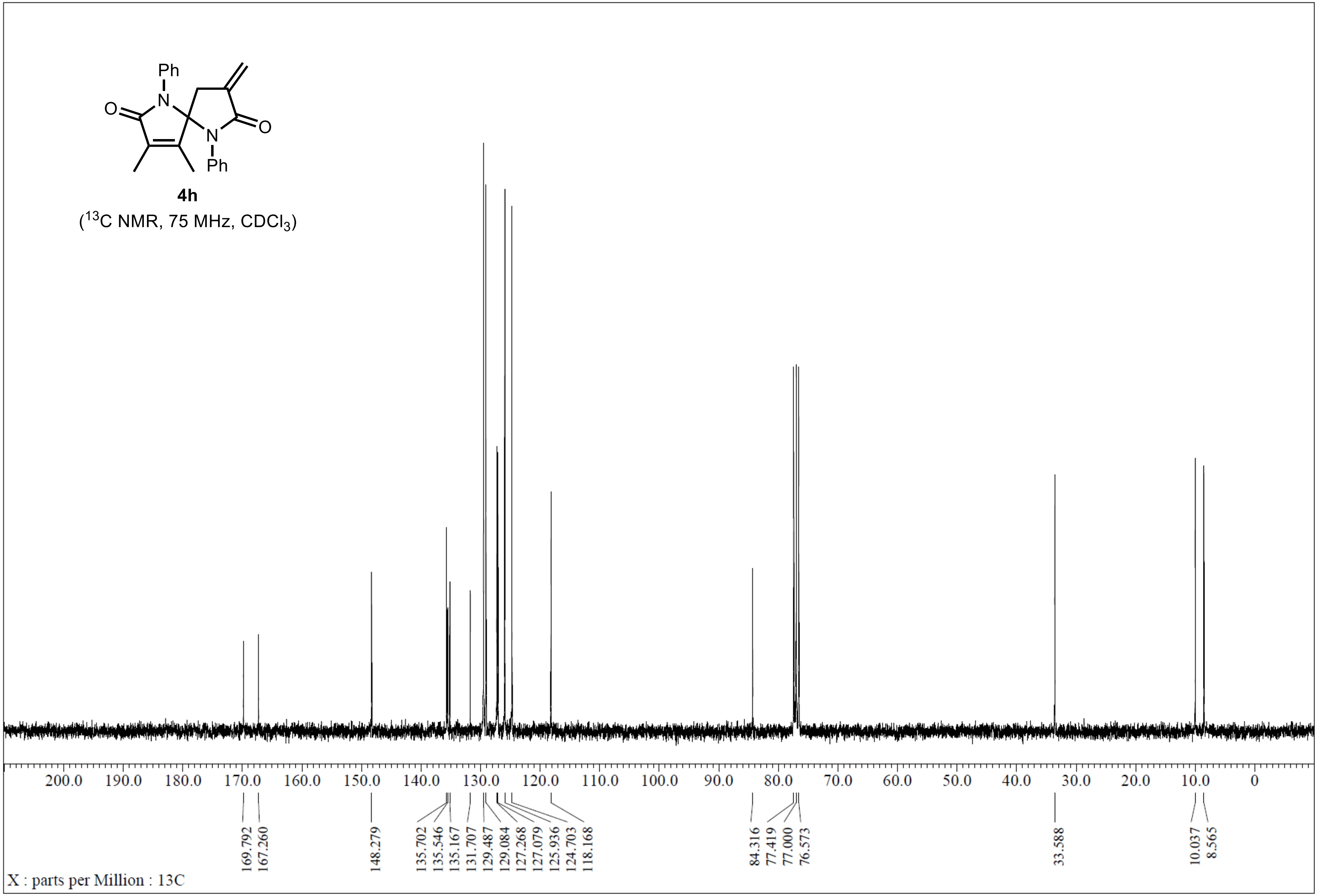
Ph
N
O
N
O
Ph
4h
(13C NMR, 75 MHz, CDCl3)
200.0
190.0
180.0
170.0
160.0
150.0
140.0
130.0
120.0
110.0
100.0
90.0
80.0
70.0
60.0
50.0
40.0
30.0
20.0
10.0
0
169.792
167.260
148.279
135.702
135.546
135.167
131.707
129.487
129.084
127.268
127.079
125.936
124.703
118.168
84.316
77.419
77.000
76.573
33.588
10.037
8.565
X : parts per Million : 13C

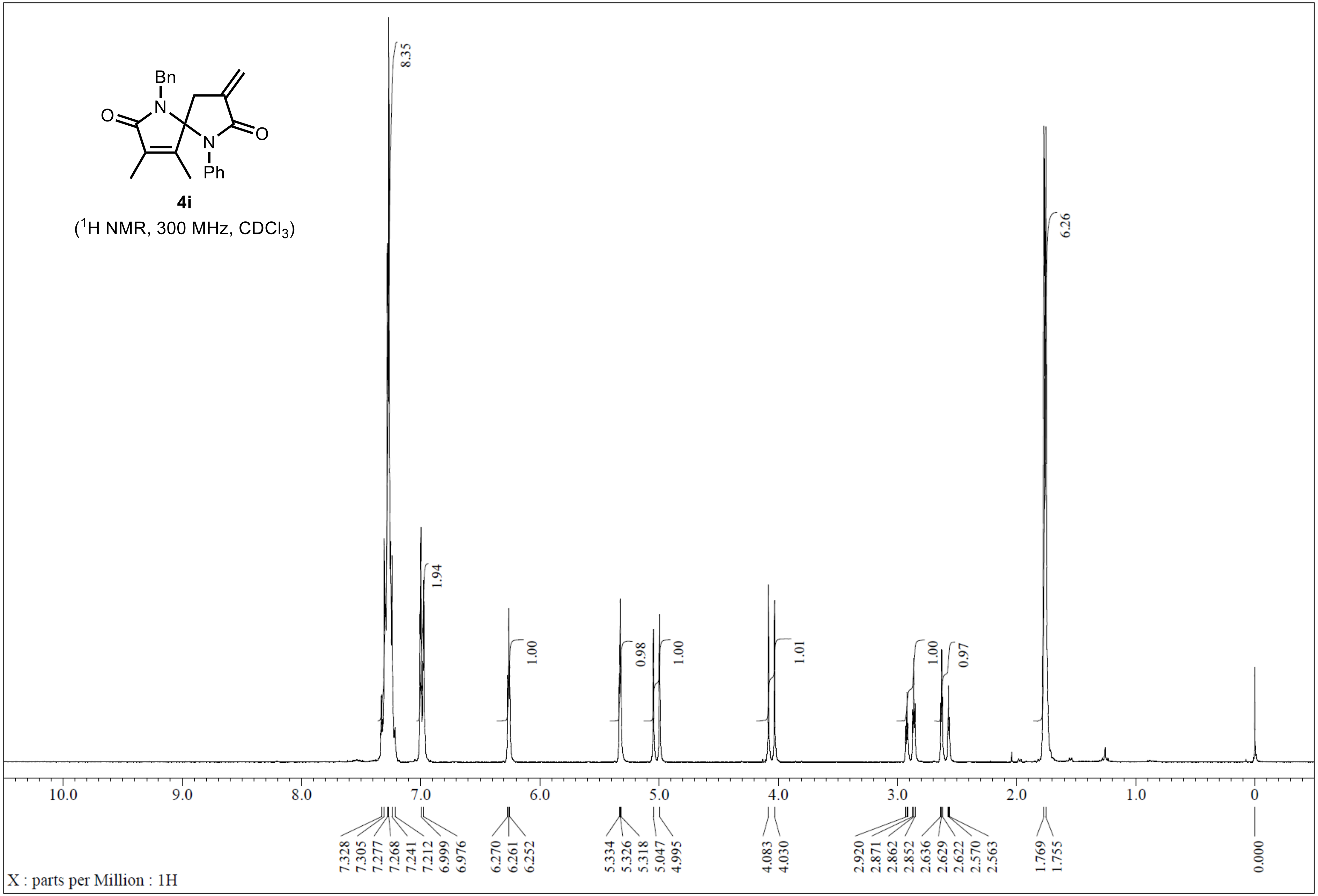
Bn
N
O
N
O
Ph
4i
(1H NMR, 300 MHz, CDCl3)
8.35
1.94
1.00
0.98
1.00
1.01
1.00
0.97
6.26
10.0
9.0
8.0
7.0
6.0
5.0
4.0
3.0
2.0
1.0
0
7.328
7.305
7.277
7.268
7.241
7.212
6.999
6.976
6.270
6.261
6.252
5.334
5.326
5.318
5.047
4.995
4.083
4.030
2.920
2.871
2.862
2.852
2.636
2.629
2.622
2.570
2.563
1.769
1.755
0.000
X : parts per Million : 1H

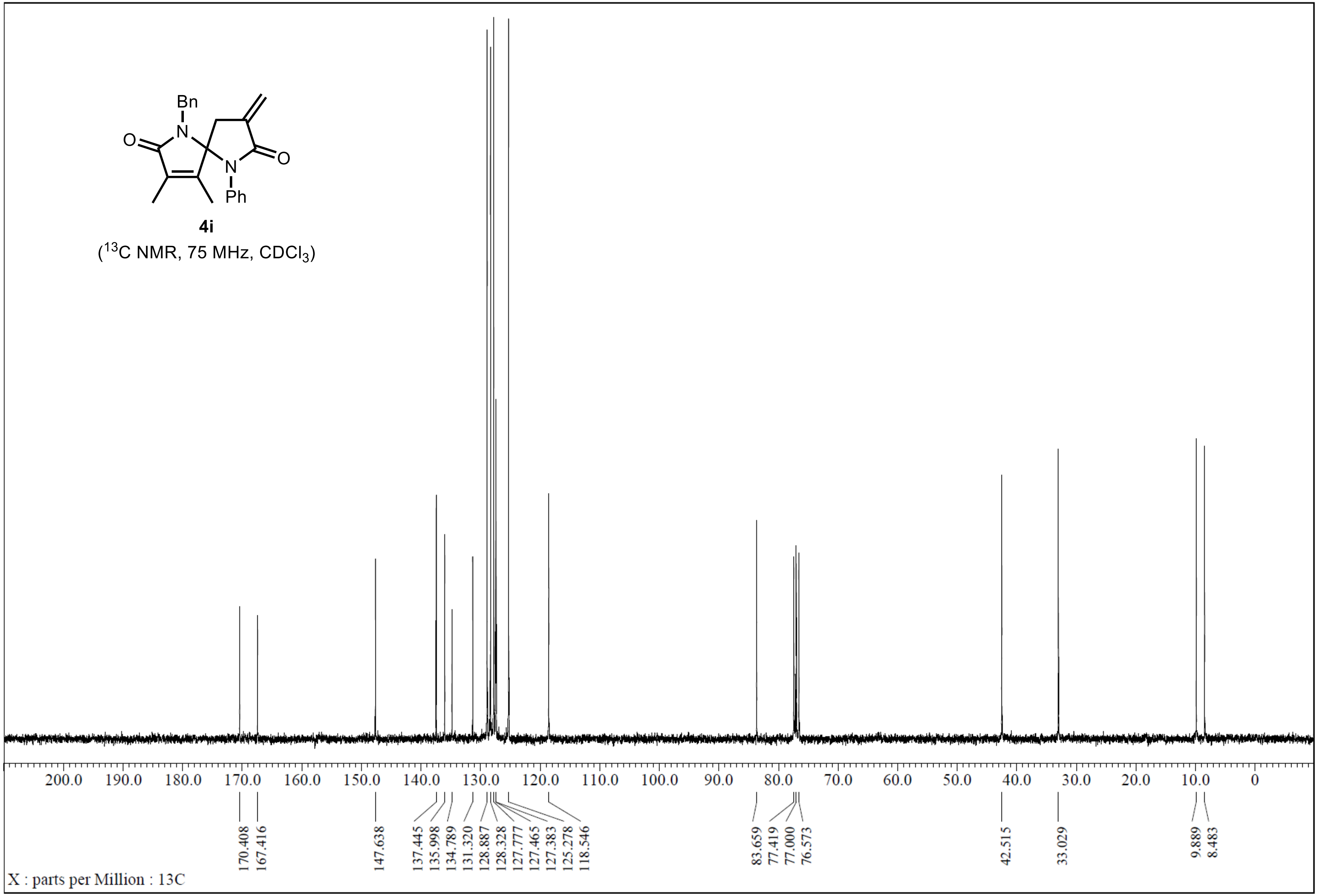
Bn
N
O
N
O
Ph
4i
(^{13}C NMR, 75 MHz, CDCl$_3$)
200.0 190.0 180.0 170.0 160.0 150.0 140.0 130.0 120.0 110.0 100.0 90.0 80.0 70.0 60.0 50.0 40.0 30.0 20.0 10.0 0
170.408
167.416
147.638
137.445
135.998
134.789
131.320
128.887
128.328
127.777
127.465
127.383
125.278
118.546
83.659
77.419
77.000
76.573
42.515
33.029
9.889
8.483
X : parts per Million : 13C

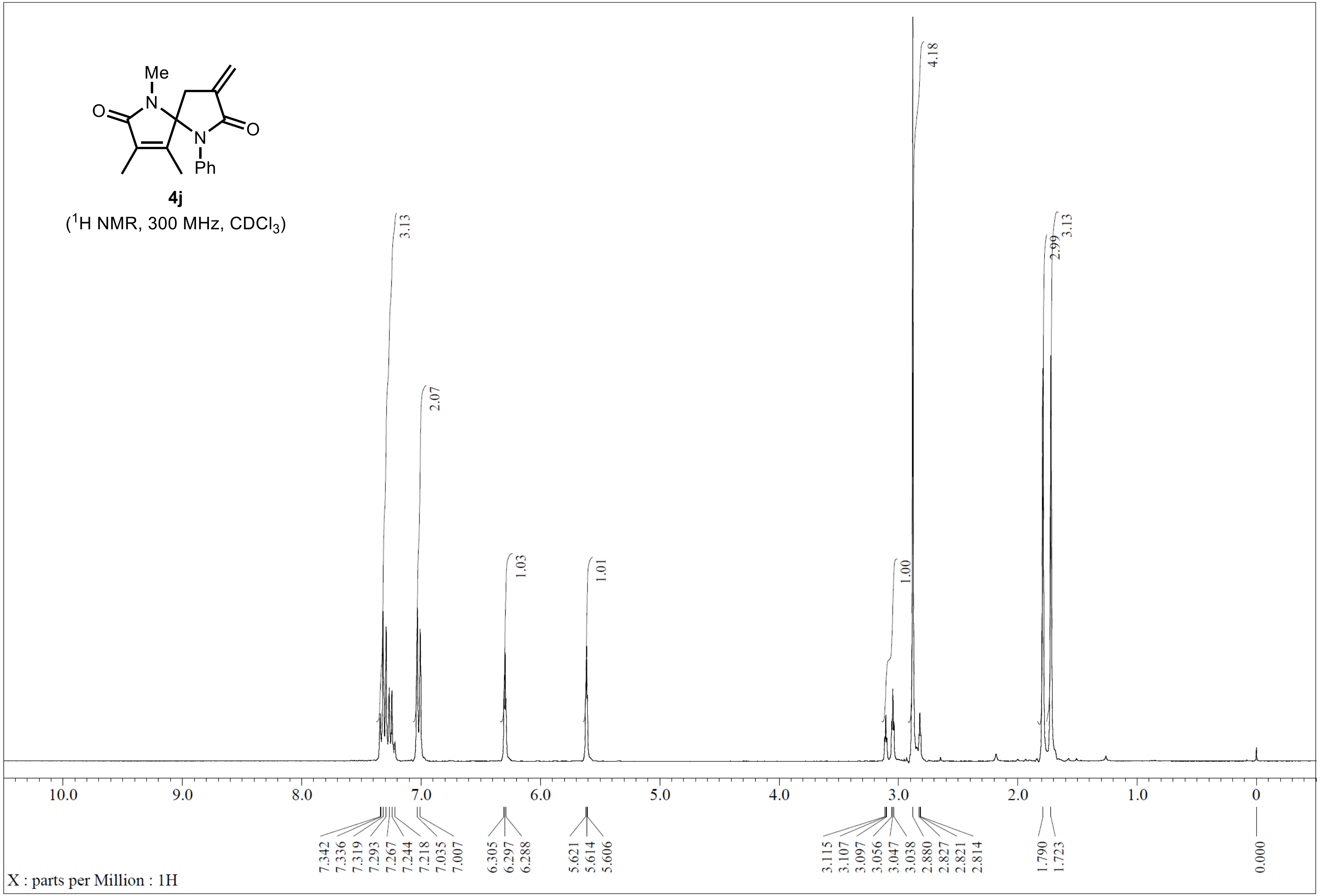
Me
N
O
N
O
Ph
4j
(¹H NMR, 300 MHz, CDCl3)
3.13
2.07
1.03
1.01
1.00
4.18
2.99
3.13
10.0
9.0
8.0
7.0
6.0
5.0
4.0
3.0
2.0
1.0
0
7.342
7.336
7.319
7.293
7.267
7.244
7.218
7.035
7.007
6.305
6.297
6.288
5.621
5.614
5.606
3.115
3.107
3.097
3.056
3.047
3.038
2.880
2.827
2.821
2.814
1.790
1.723
0.000
X : parts per Million : 1H

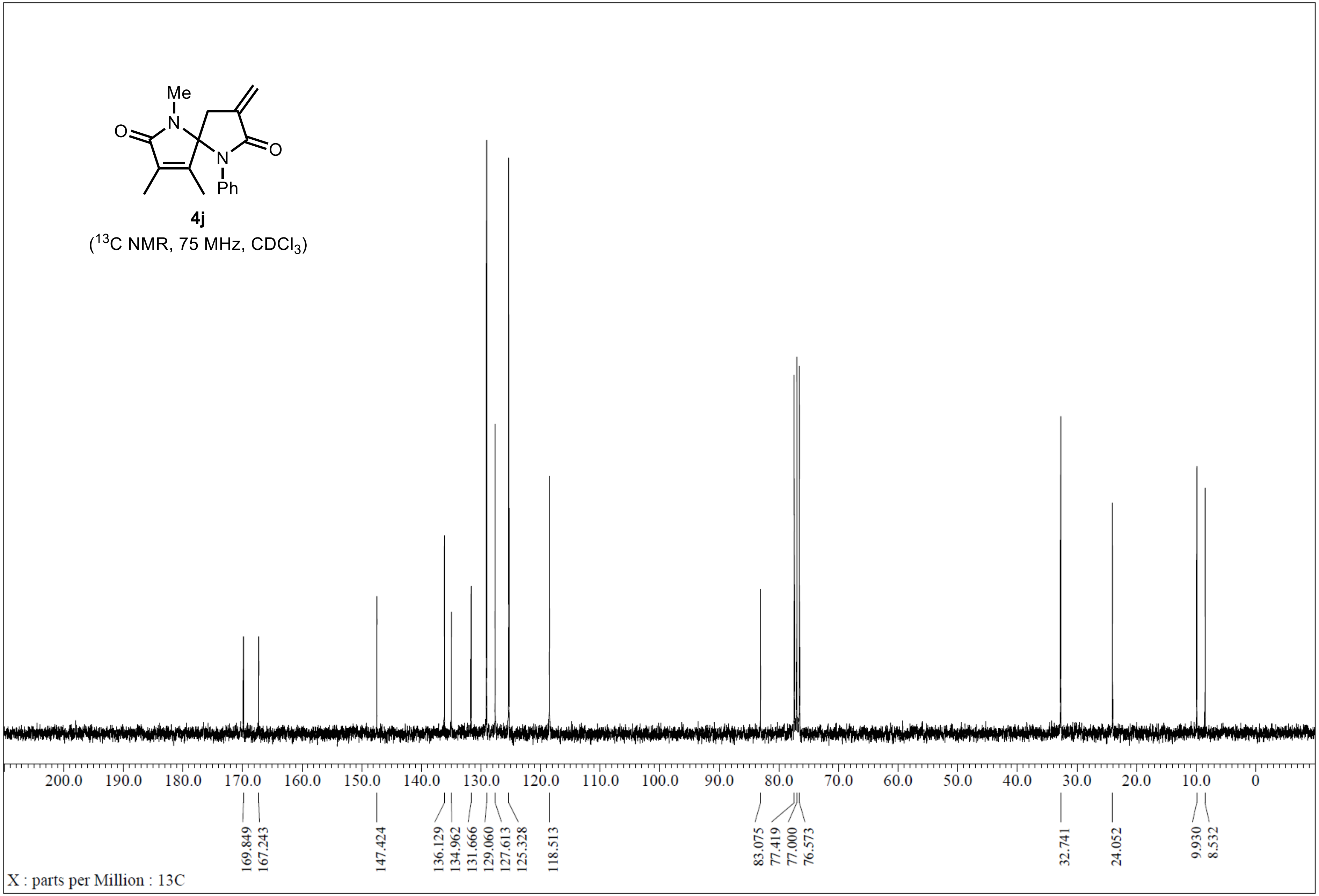
Me
O
N
O
N
Ph
4j
(^{13}C NMR, 75 MHz, $CDCl_3$)
200.0
190.0
180.0
170.0
160.0
150.0
140.0
130.0
120.0
110.0
100.0
90.0
80.0
70.0
60.0
50.0
40.0
30.0
20.0
10.0
0
169.849
167.243
147.424
136.129
134.962
131.666
129.060
127.613
125.328
118.513
83.075
77.419
77.000
76.573
32.741
24.052
9.930
8.532
X : parts per Million : 13C

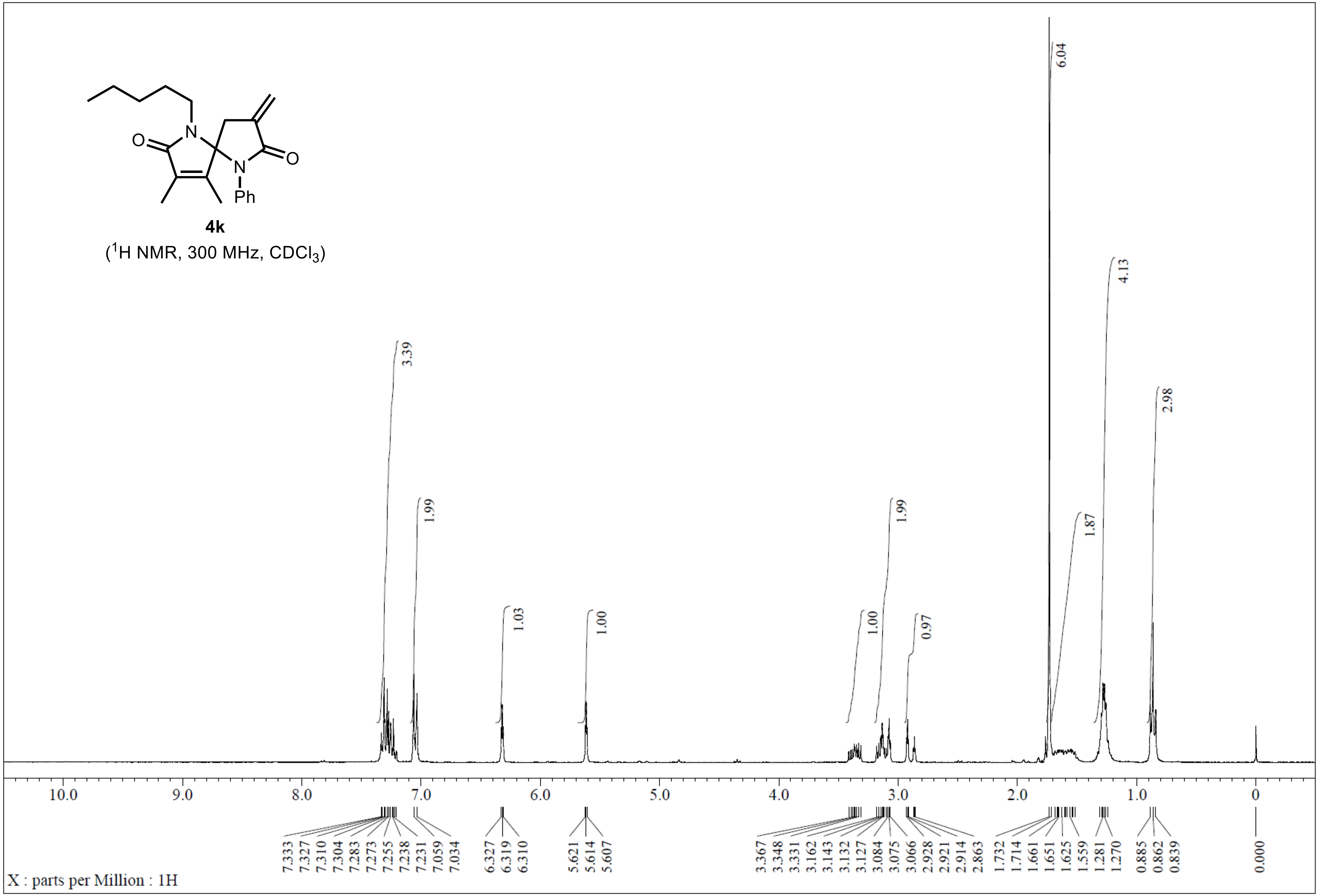
4k
(^{1}H NMR, 300 MHz, CDCl$_3$)
Ph
3.39
1.99
1.03
1.00
1.00
1.99
0.97
6.04
1.87
4.13
2.98
10.0 9.0 8.0 7.0 6.0 5.0 4.0 3.0 2.0 1.0 0
7.333 7.327 7.310 7.304 7.283 7.273 7.255 7.238 7.231 7.059 7.034
6.327 6.319 6.310
5.621 5.614 5.607
3.367 3.348 3.331 3.162 3.143 3.132 3.127 3.084 3.075 3.066 2.928 2.921 2.914 2.863
1.732 1.714 1.661 1.651 1.625 1.559 1.281 1.270
0.885 0.862 0.839
0.000
X : parts per Million : 1H

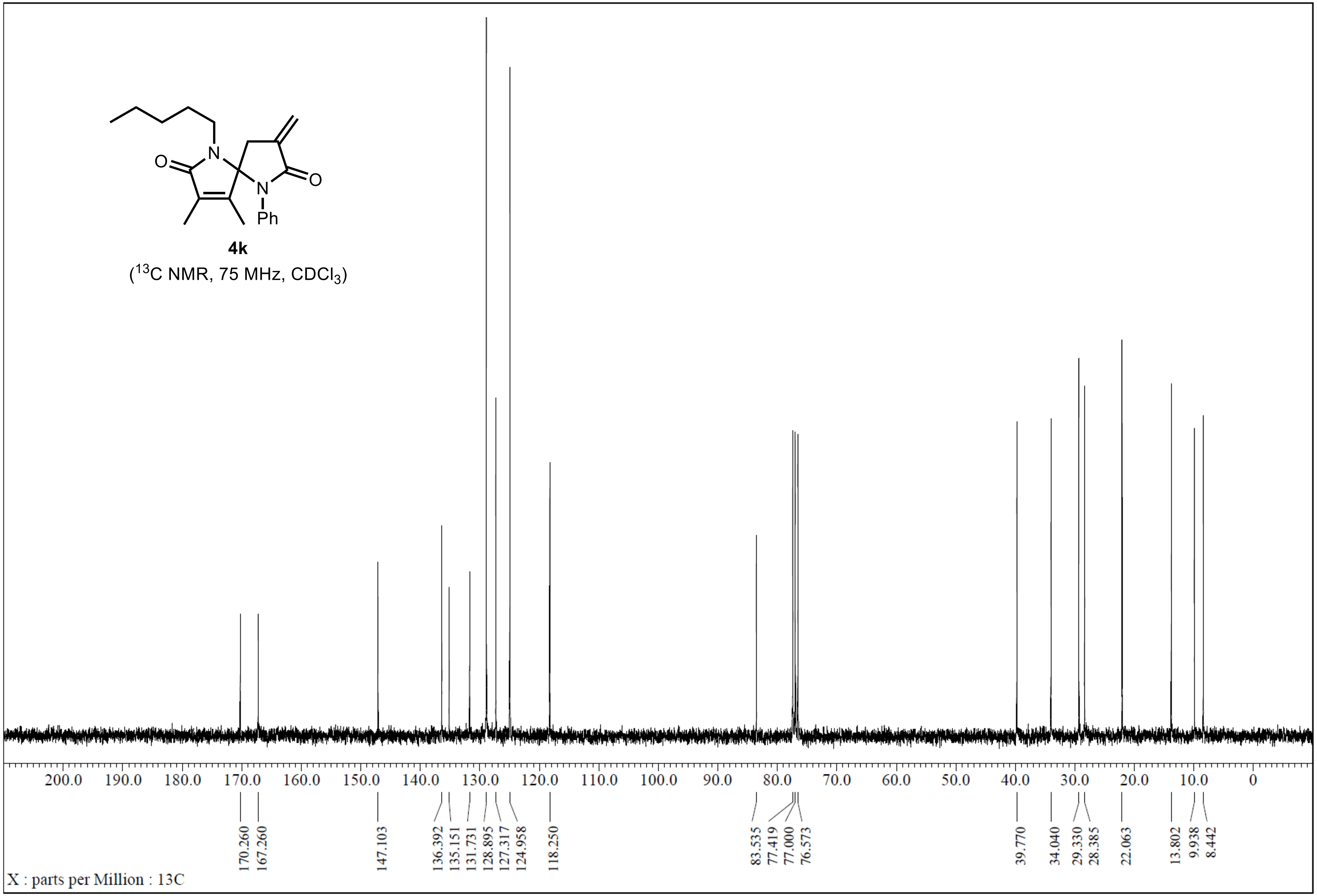

4k

(^{13}C NMR, 75 MHz, $CDCl_3$)

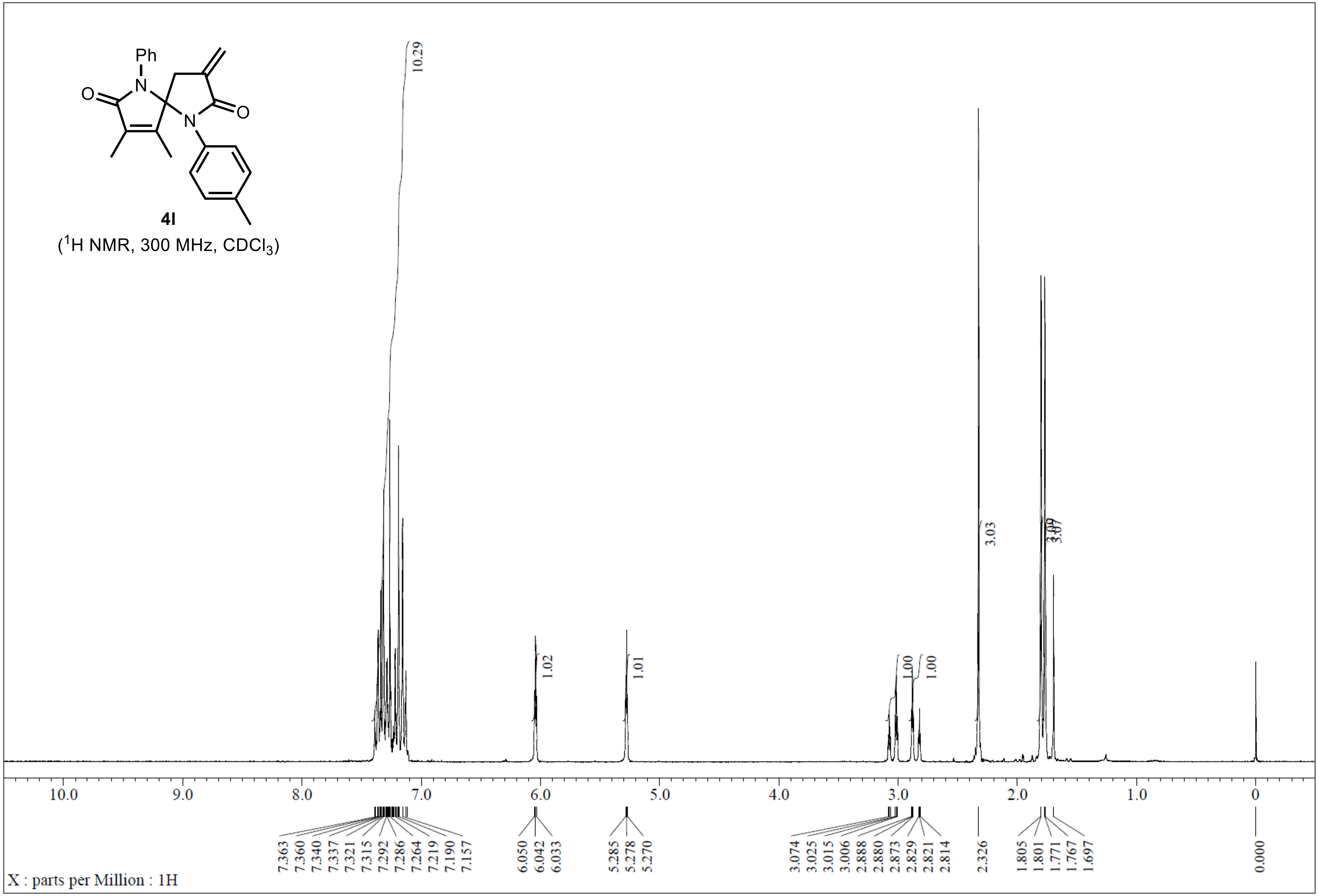
Ph
N
O
N
O
4l
(1H NMR, 300 MHz, CDCl3)
10.29
3.03
1.02
1.01
1.00
1.00
10.0
9.0
8.0
7.0
6.0
5.0
4.0
3.0
2.0
1.0
0
7.363
7.360
7.340
7.337
7.321
7.315
7.292
7.286
7.264
7.219
7.190
7.157
6.050
6.042
6.033
5.285
5.278
5.270
3.074
3.025
3.015
3.006
2.888
2.880
2.873
2.829
2.821
2.814
2.326
1.805
1.801
1.771
1.767
1.697
0.000
X : parts per Million : 1H

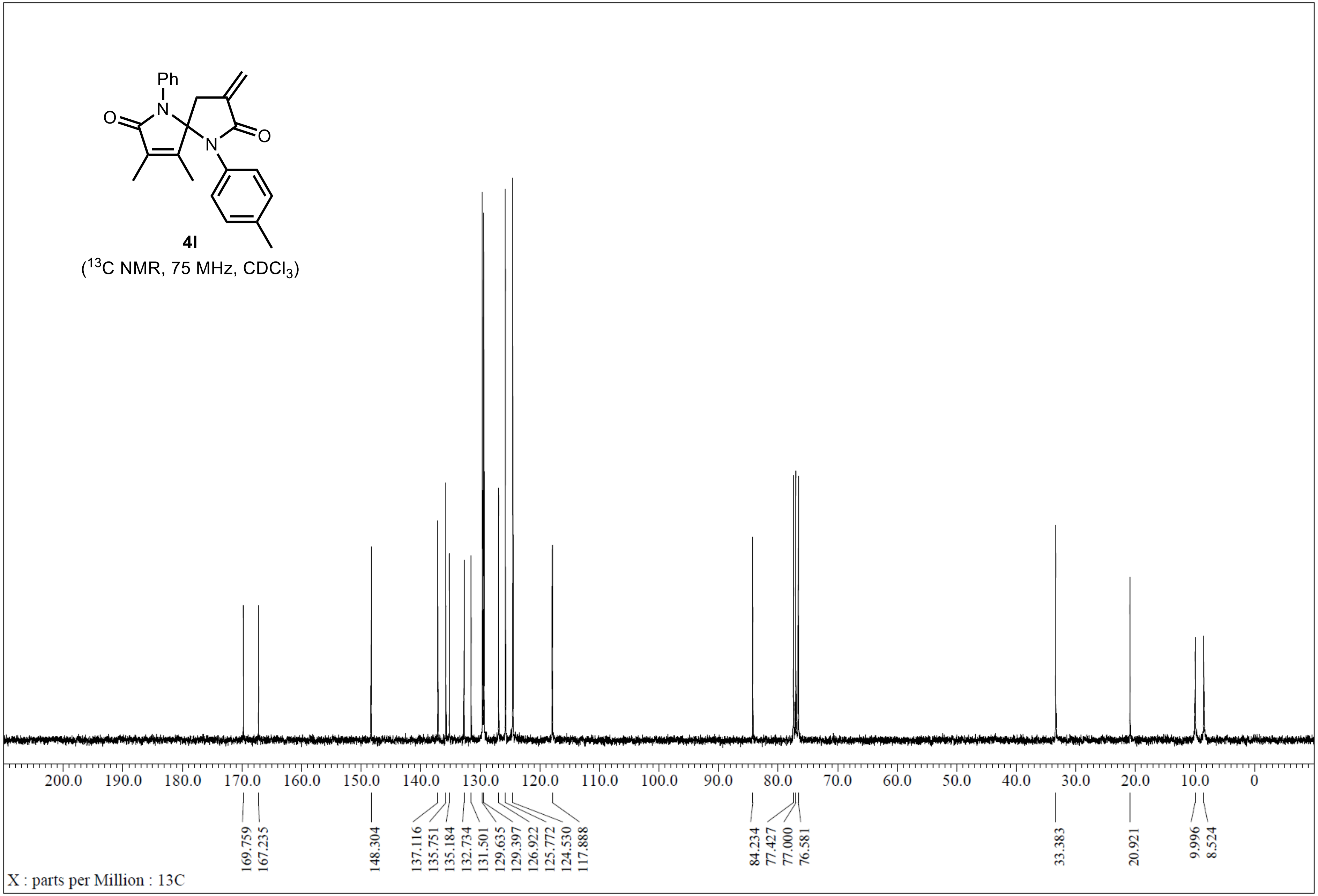
Ph
N
O
N
O
4l
(13C NMR, 75 MHz, CDCl3)
200.0 190.0 180.0 170.0 160.0 150.0 140.0 130.0 120.0 110.0 100.0 90.0 80.0 70.0 60.0 50.0 40.0 30.0 20.0 10.0 0
169.759
167.235
148.304
137.116
135.751
135.184
132.734
131.501
129.635
129.397
126.922
125.772
124.530
117.888
84.234
77.427
77.000
76.581
33.383
20.921
9.996
8.524
X : parts per Million : 13C

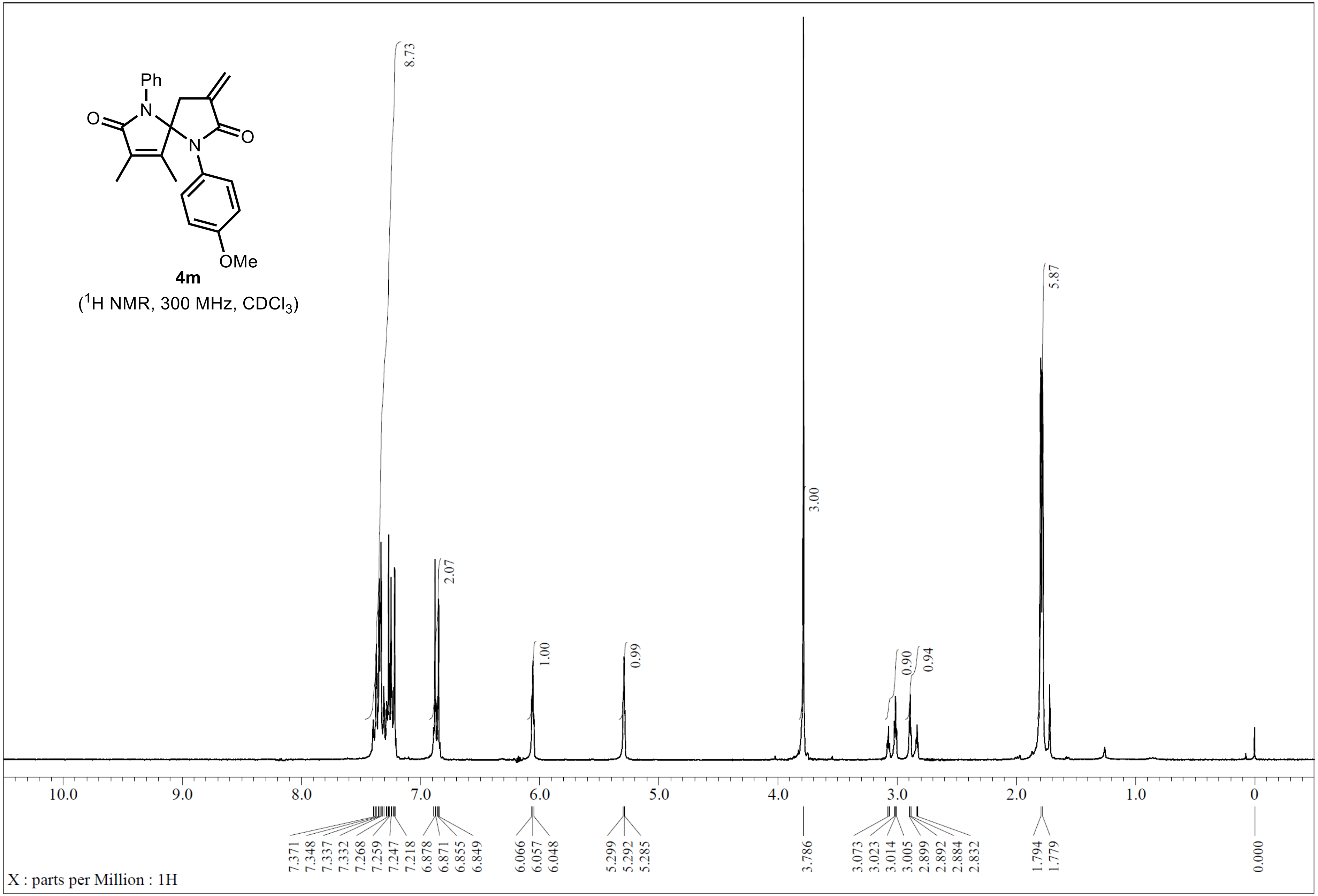
Ph
N
O
N
O
OMe
4m
(1H NMR, 300 MHz, CDCl3)
8.73
2.07
1.00
0.99
3.00
0.90
0.94
5.87
10.0
9.0
8.0
7.0
6.0
5.0
4.0
3.0
2.0
1.0
0
7.371 7.348 7.337 7.332 7.268 7.259 7.247 7.218 6.878 6.871 6.855 6.849
6.066 6.057 6.048
5.299 5.292 5.285
3.786
3.073 3.023 3.014 3.005 2.899 2.892 2.884 2.832
1.794 1.779
0.000
X : parts per Million : 1H

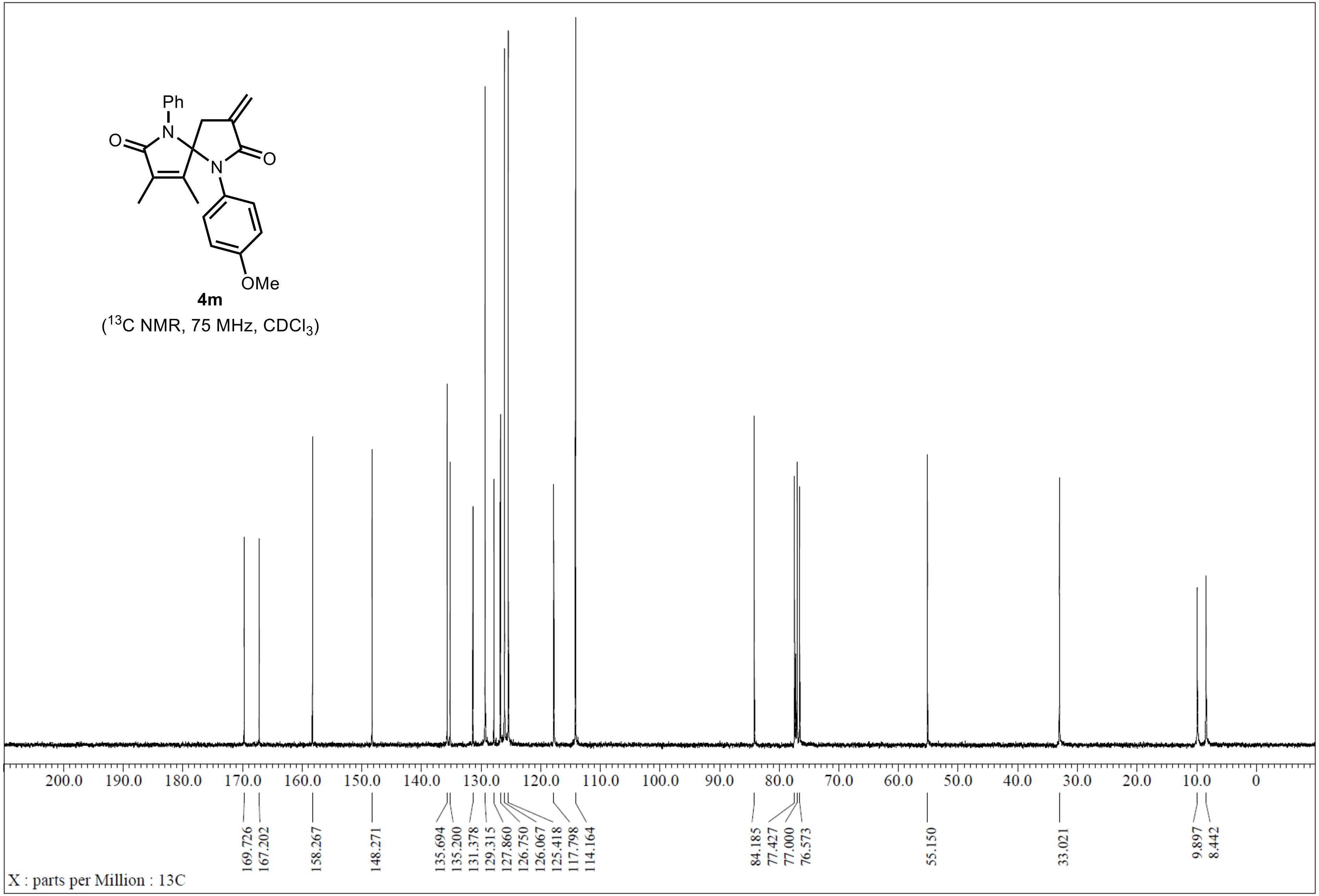
Ph
N
O
N
O
OMe
4m
(13C NMR, 75 MHz, CDCl3)
200.0 190.0 180.0 170.0 160.0 150.0 140.0 130.0 120.0 110.0 100.0 90.0 80.0 70.0 60.0 50.0 40.0 30.0 20.0 10.0 0
169.726
167.202
158.267
148.271
135.694
135.200
131.378
129.315
127.860
126.750
126.067
125.418
117.798
114.164
84.185
77.427
77.000
76.573
55.150
33.021
9.897
8.442
X : parts per Million : 13C

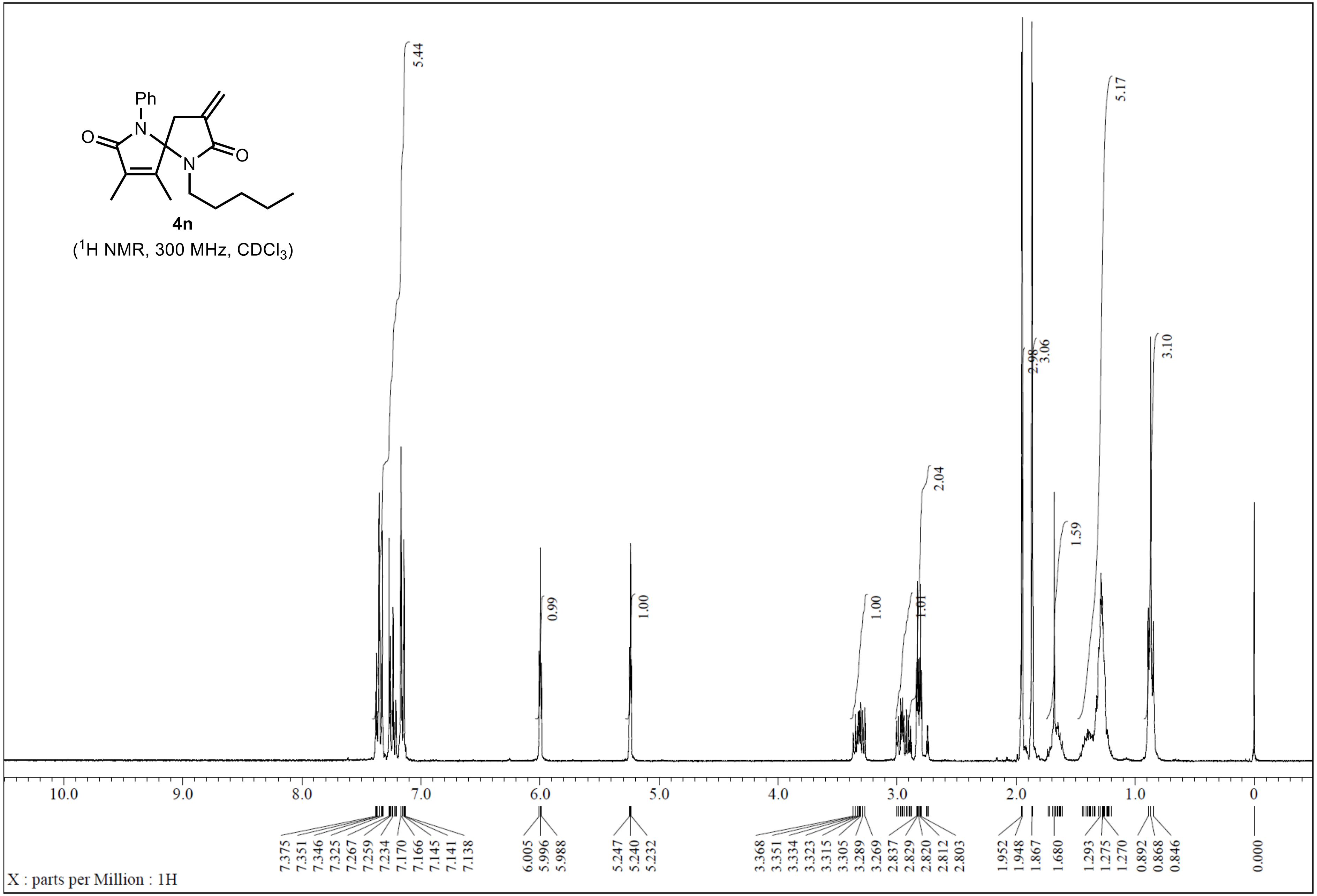
Ph
N
O
O
N
4n
(1H NMR, 300 MHz, CDCl3)
5.44
0.99
1.00
1.00
1.01
2.04
2.98
3.06
1.59
5.17
3.10
10.0
9.0
8.0
7.0
6.0
5.0
4.0
3.0
2.0
1.0
0
7.375
7.351
7.346
7.325
7.267
7.259
7.234
7.170
7.166
7.145
7.141
7.138
6.005
5.996
5.988
5.247
5.240
5.232
3.368
3.351
3.334
3.323
3.315
3.305
3.289
3.269
2.837
2.829
2.820
2.812
2.803
1.952
1.948
1.867
1.680
1.293
1.275
1.270
0.892
0.868
0.846
0.000
X : parts per Million : 1H

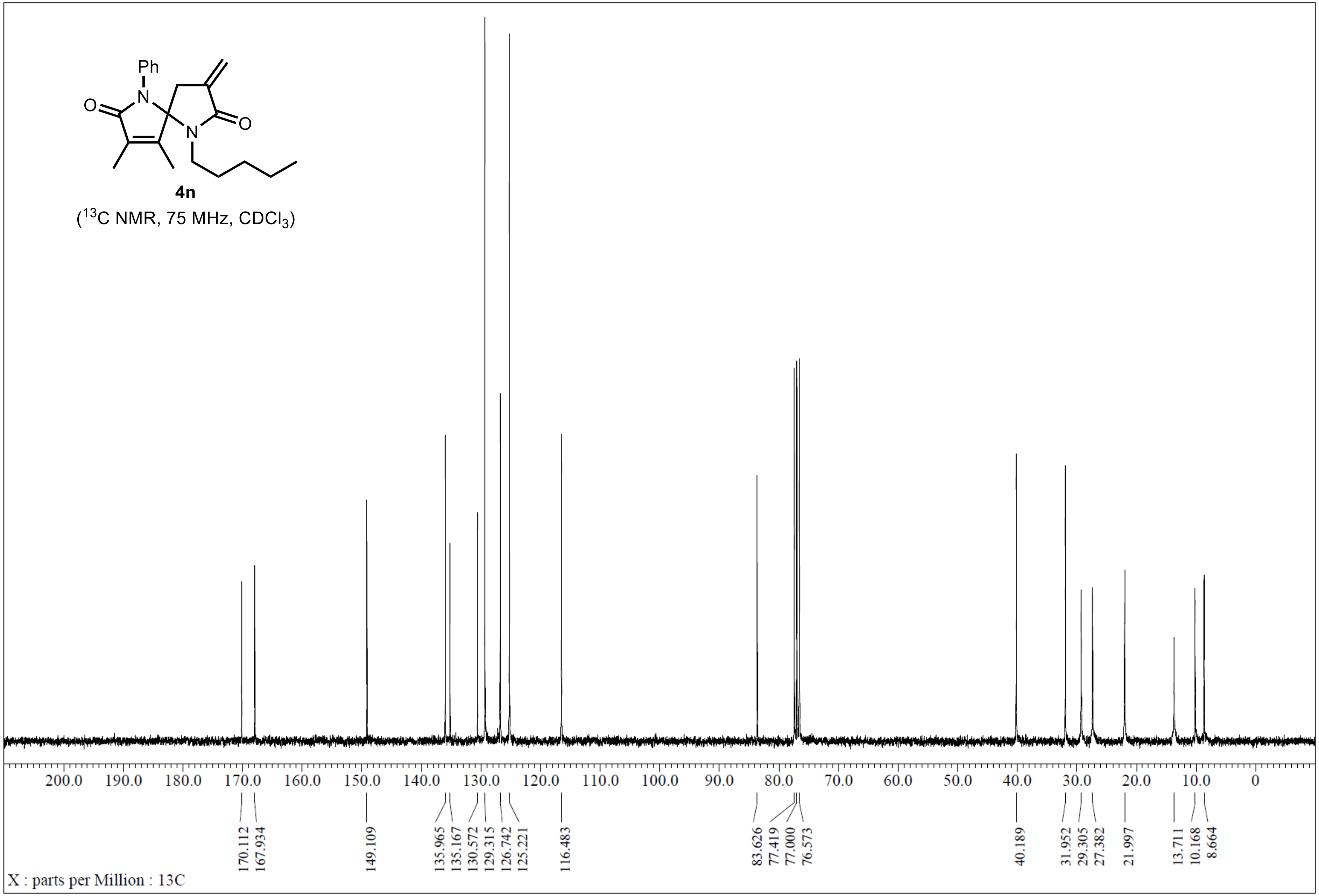
Ph
N
O
N
O
4n
(13C NMR, 75 MHz, CDCl3)
200.0 190.0 180.0 170.0 160.0 150.0 140.0 130.0 120.0 110.0 100.0 90.0 80.0 70.0 60.0 50.0 40.0 30.0 20.0 10.0 0
170.112
167.934
149.109
135.965
135.167
130.572
129.315
126.742
125.221
116.483
83.626
77.419
77.000
76.573
40.189
31.952
29.305
27.382
21.997
13.711
10.168
8.664
X : parts per Million : 13C

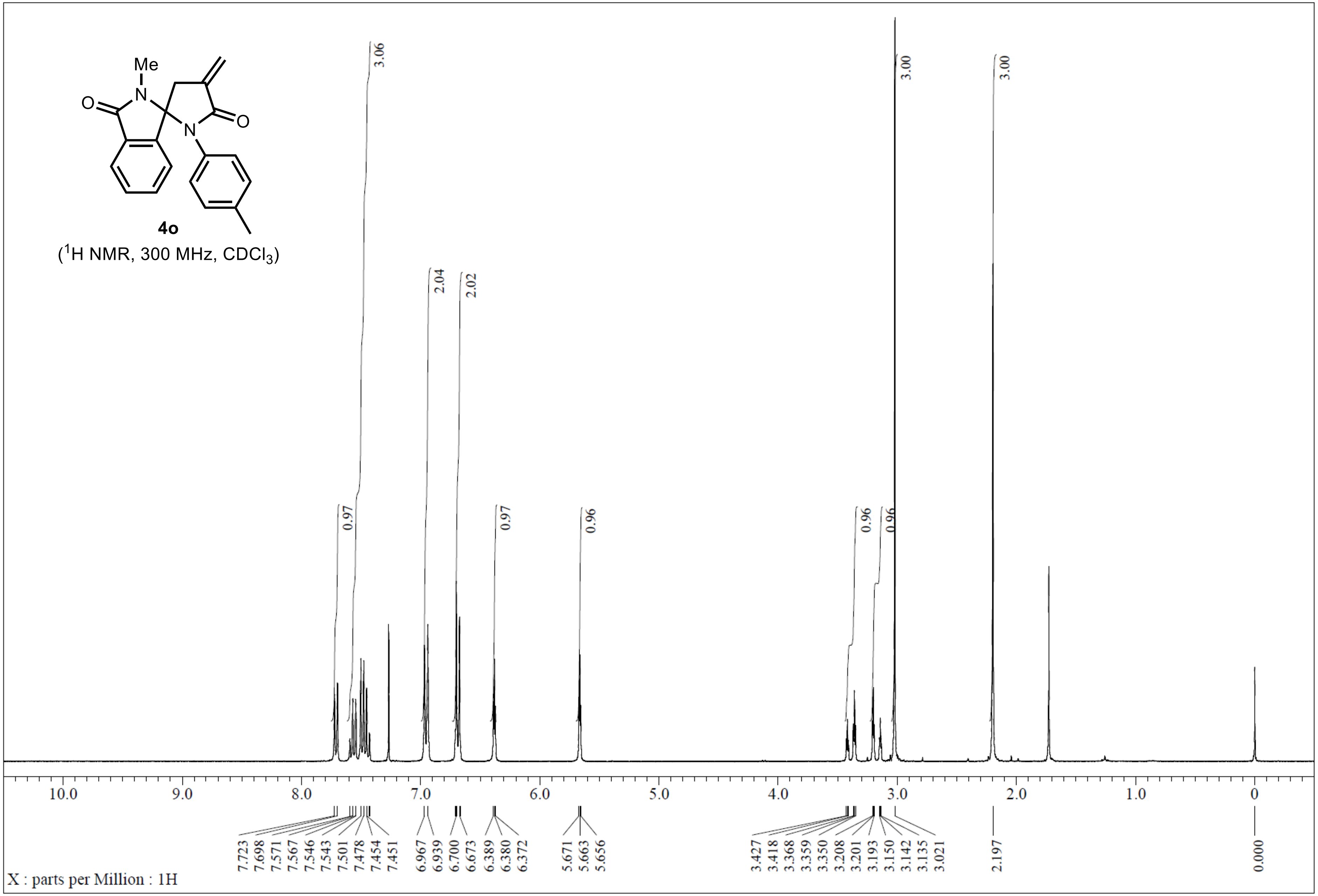

Me
N
O
N
O
4o
(1H NMR, 300 MHz, CDCl3)
3.06
0.97
2.04
2.02
0.97
0.96
0.96
0.96
3.00
3.00
10.0
9.0
8.0
7.0
6.0
5.0
4.0
3.0
2.0
1.0
0
7.723
7.698
7.571
7.567
7.546
7.543
7.501
7.478
7.454
7.451
6.967
6.939
6.700
6.673
6.389
6.380
6.372
5.671
5.663
5.656
3.427
3.418
3.368
3.359
3.350
3.208
3.201
3.193
3.150
3.142
3.135
3.021
2.197
0.000
X : parts per Million : 1H

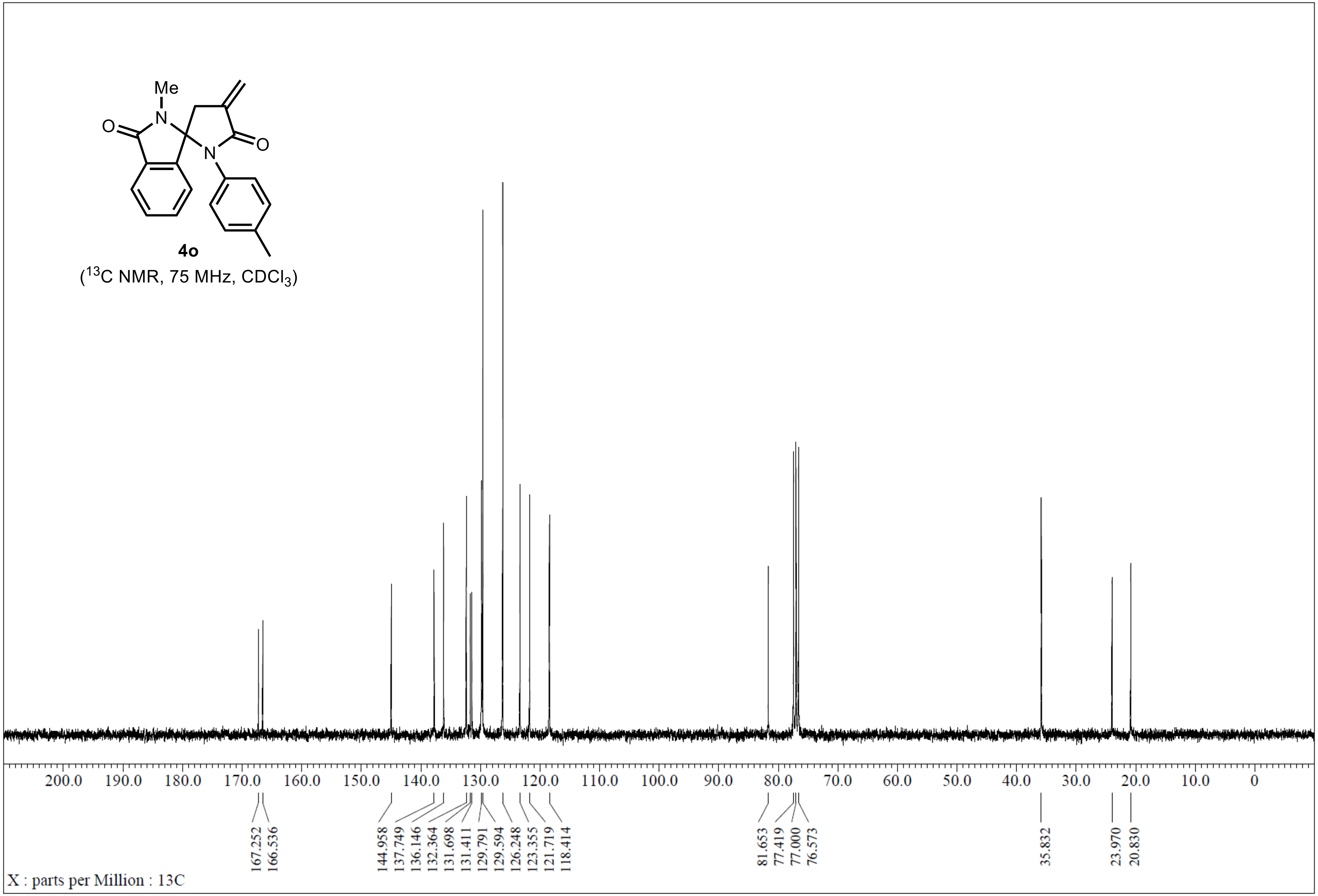
Me
N
O
N
O
4o
(13C NMR, 75 MHz, CDCl3)
200.0
190.0
180.0
170.0
160.0
150.0
140.0
130.0
120.0
110.0
100.0
90.0
80.0
70.0
60.0
50.0
40.0
30.0
20.0
10.0
0
167.252
166.536
144.958
137.749
136.146
132.364
131.698
131.411
129.791
129.594
126.248
123.355
121.719
118.414
81.653
77.419
77.000
76.573
35.832
23.970
20.830
X : parts per Million : 13C

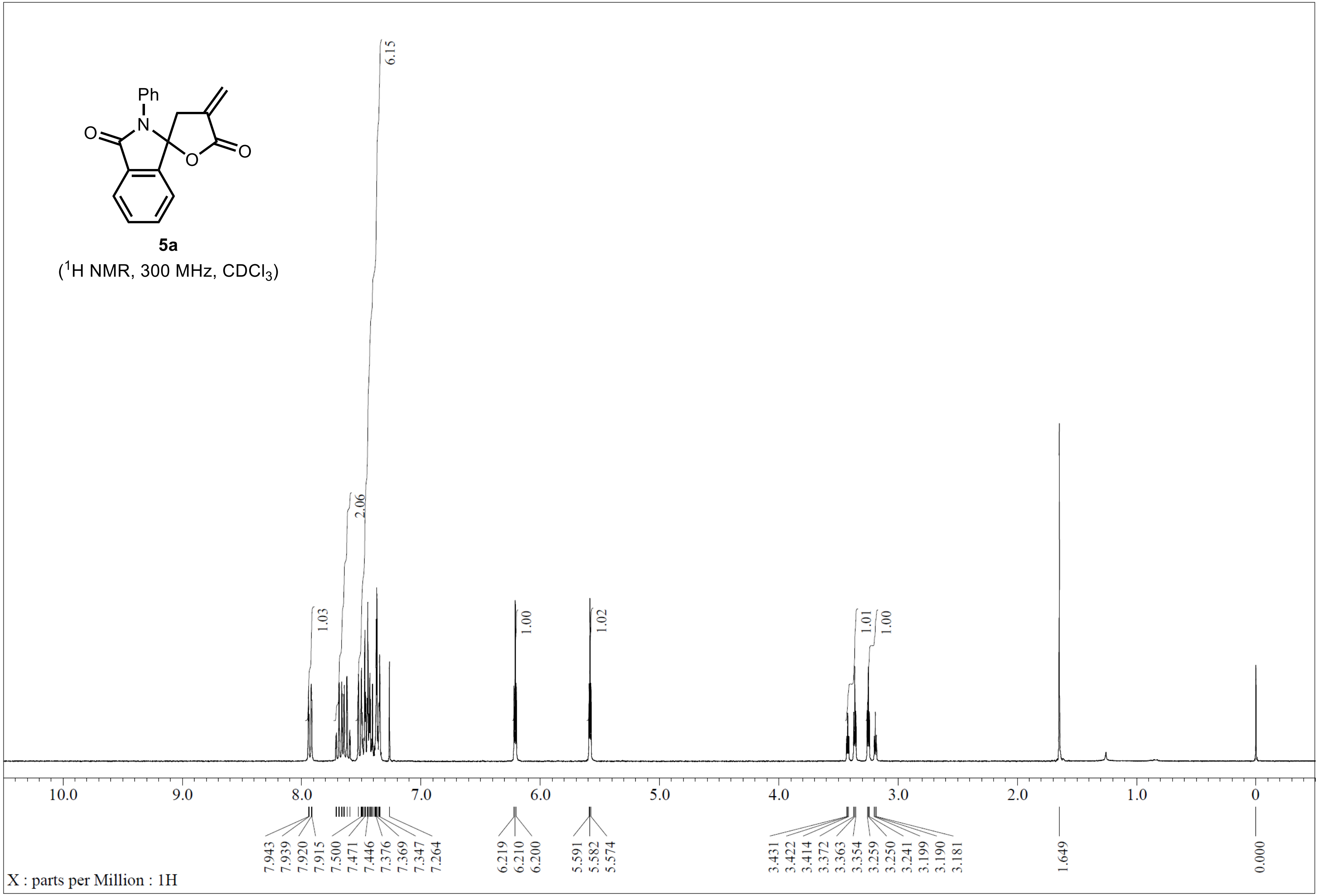
Ph
N
O
O
O
5a
(¹H NMR, 300 MHz, CDCl₃)
6.15
2.06
1.03
1.00
1.02
1.01
1.00
10.0
9.0
8.0
7.0
6.0
5.0
4.0
3.0
2.0
1.0
0
7.943
7.939
7.920
7.915
7.500
7.471
7.446
7.376
7.369
7.347
7.264
6.219
6.210
6.200
5.591
5.582
5.574
3.431
3.422
3.414
3.372
3.363
3.354
3.259
3.250
3.241
3.199
3.190
3.181
1.649
0.000
X : parts per Million : 1H

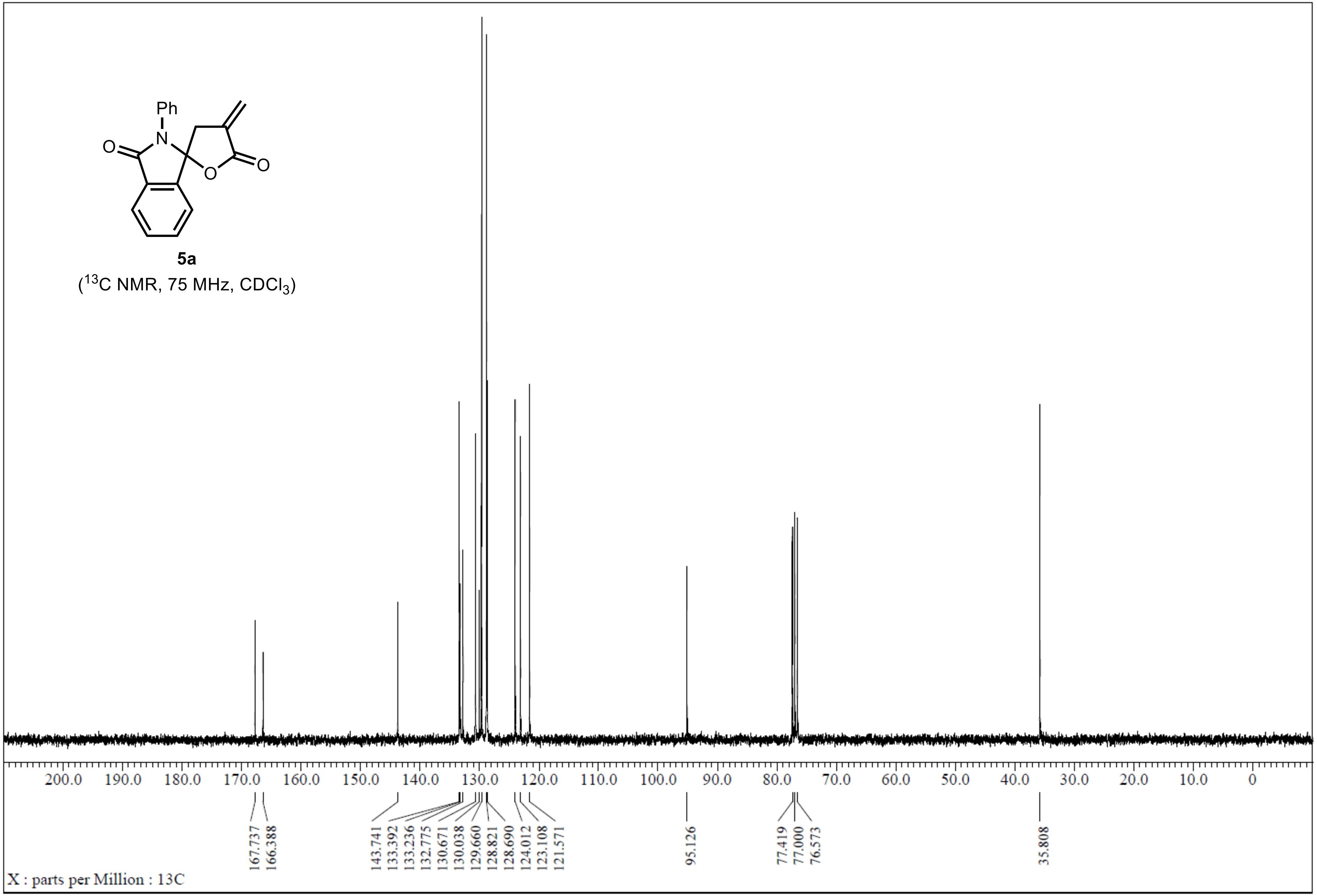

Ph
N
O
O
O
5a
(13C NMR, 75 MHz, CDCl3)
200.0
190.0
180.0
170.0
160.0
150.0
140.0
130.0
120.0
110.0
100.0
90.0
80.0
70.0
60.0
50.0
40.0
30.0
20.0
10.0
0
167.737
166.388
143.741
133.392
133.236
132.775
130.671
130.038
129.660
128.821
128.690
124.012
123.108
121.571
95.126
77.419
77.000
76.573
35.808
X : parts per Million : 13C

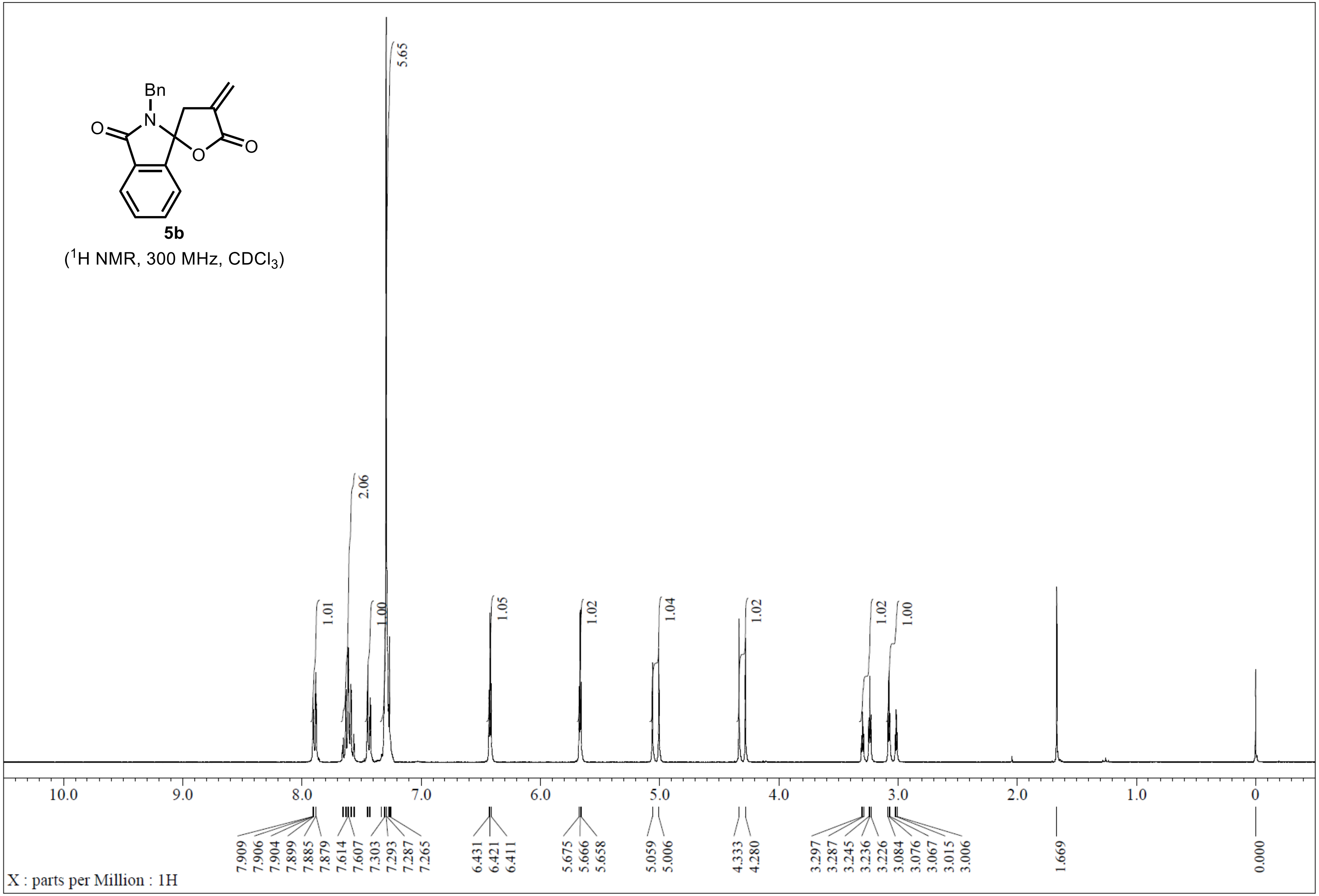
Bn
N
O
O
O
5b
(1H NMR, 300 MHz, CDCl3)
5.65
2.06
1.01
1.00
1.05
1.02
1.04
1.02
1.02
1.00
10.0
9.0
8.0
7.0
6.0
5.0
4.0
3.0
2.0
1.0
0
7.909
7.906
7.904
7.899
7.885
7.879
7.614
7.607
7.303
7.293
7.287
7.265
6.431
6.421
6.411
5.675
5.666
5.658
5.059
5.006
4.333
4.280
3.297
3.287
3.245
3.236
3.226
3.084
3.076
3.067
3.015
3.006
1.669
0.000
X : parts per Million : 1H

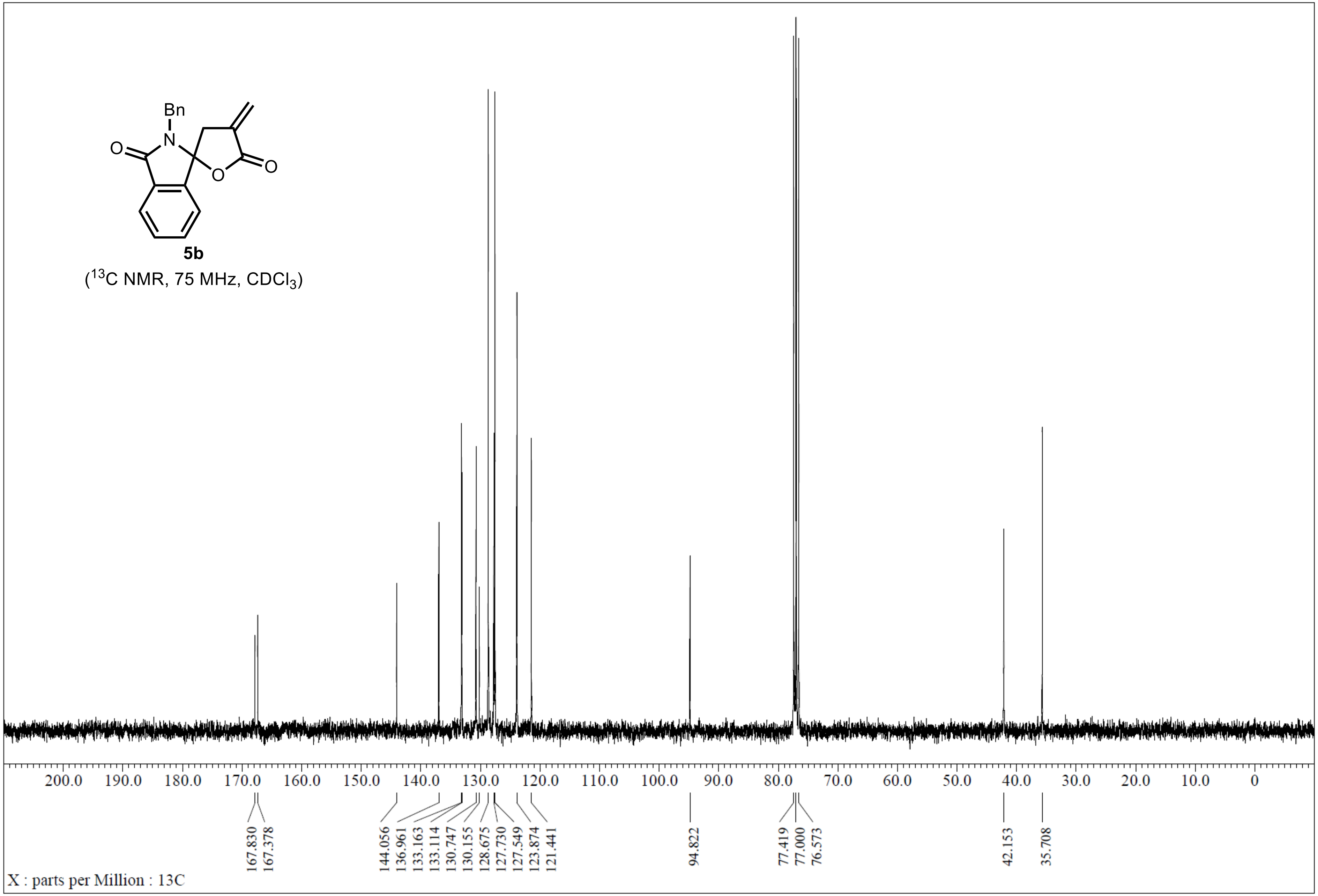
Bn
N
O
O
O
5b
(13C NMR, 75 MHz, CDCl3)
200.0 190.0 180.0 170.0 160.0 150.0 140.0 130.0 120.0 110.0 100.0 90.0 80.0 70.0 60.0 50.0 40.0 30.0 20.0 10.0 0
167.830
167.378
144.056
136.961
133.163
133.114
130.747
130.155
128.675
127.730
127.549
123.874
121.441
94.822
77.419
77.000
76.573
42.153
35.708
X : parts per Million : 13C

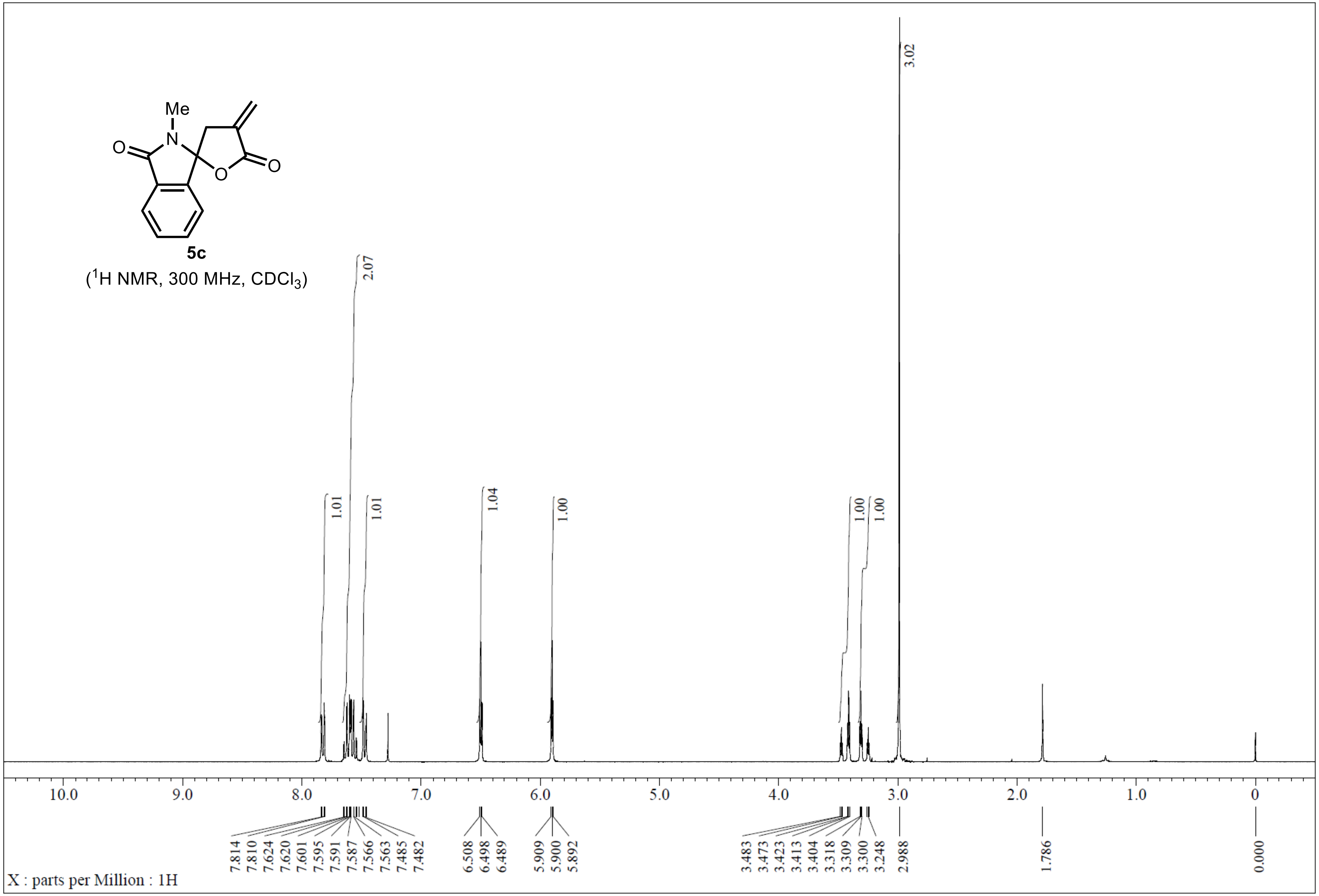
Me
N
O
O
O
5c
(1H NMR, 300 MHz, CDCl3)
3.02
2.07
1.01
1.01
1.04
1.00
1.00
1.00
10.0
9.0
8.0
7.0
6.0
5.0
4.0
3.0
2.0
1.0
0
7.814
7.810
7.624
7.620
7.601
7.595
7.591
7.587
7.566
7.563
7.485
7.482
6.508
6.498
6.489
5.909
5.900
5.892
3.483
3.473
3.423
3.413
3.404
3.318
3.309
3.300
3.248
2.988
1.786
0.000
X : parts per Million : 1H

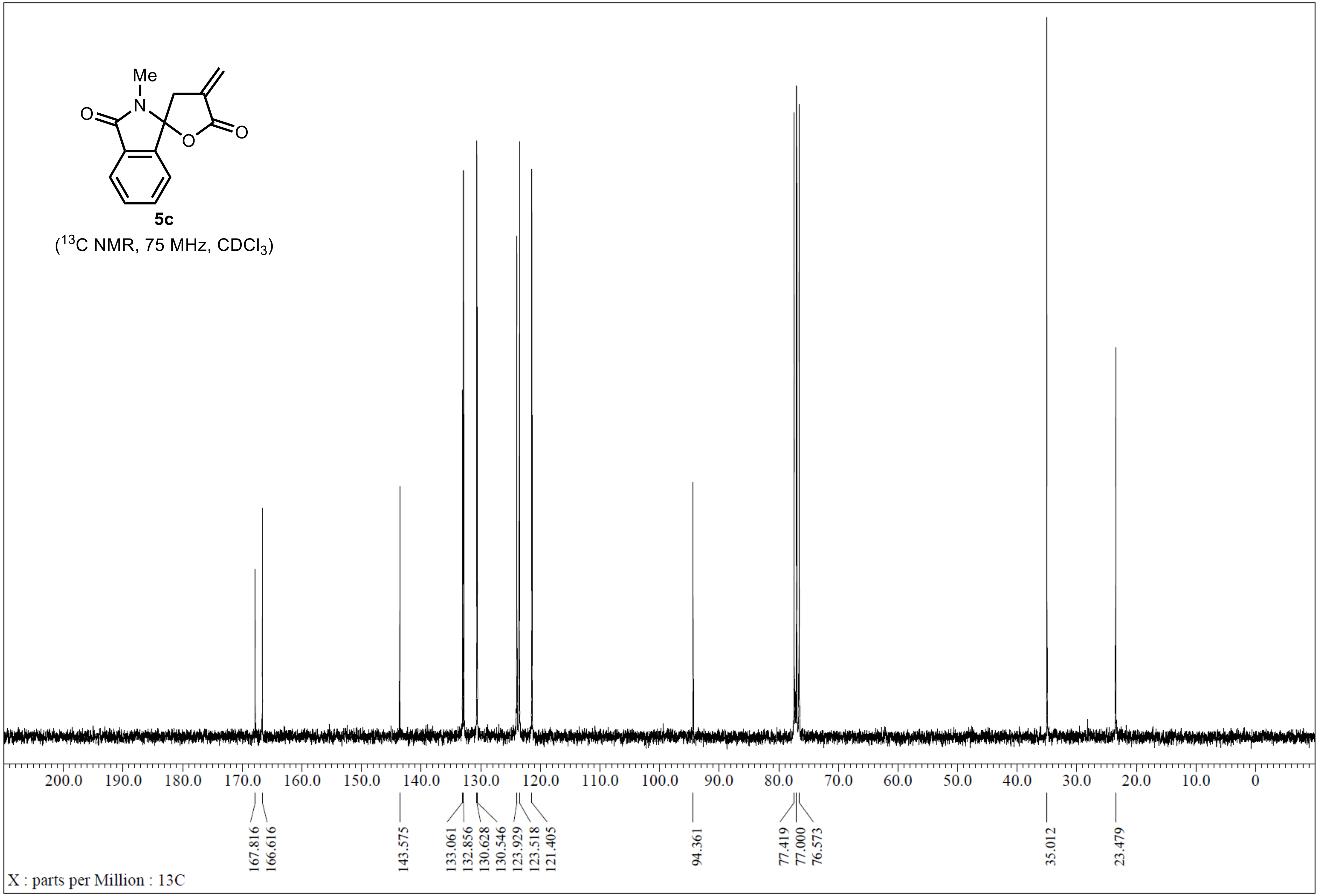
Me
N
O
O
O
5c
(13C NMR, 75 MHz, CDCl3)
200.0 190.0 180.0 170.0 160.0 150.0 140.0 130.0 120.0 110.0 100.0 90.0 80.0 70.0 60.0 50.0 40.0 30.0 20.0 10.0 0
167.816
166.616
143.575
133.061
132.856
130.628
130.546
123.929
123.518
121.405
94.361
77.419
77.000
76.573
35.012
23.479
X : parts per Million : 13C

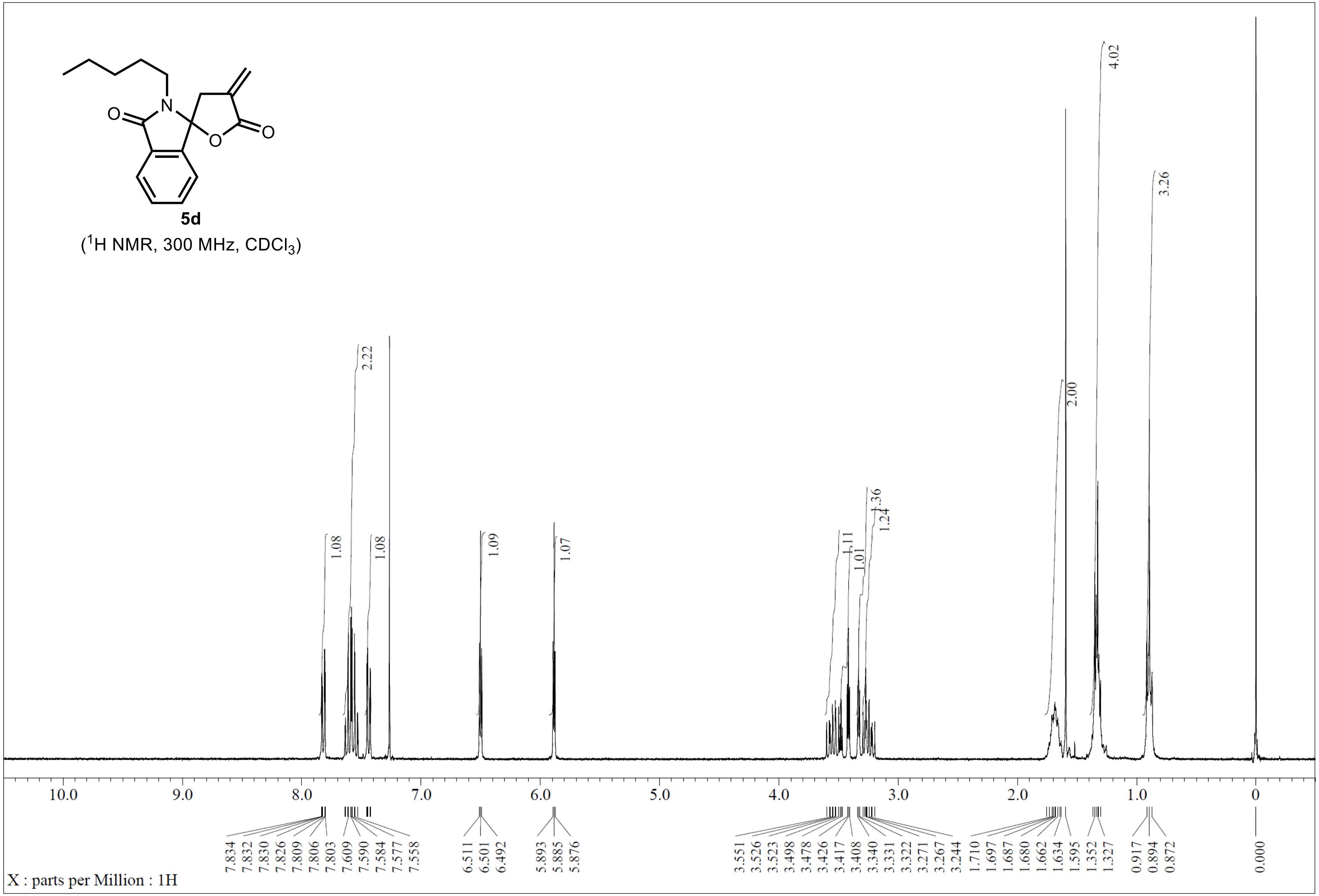

5d
(^{1}H NMR, 300 MHz, $CDCl_3$)
4.02
3.26
2.22
2.00
1.08
1.08
1.09
1.07
1.11
1.01
1.36
1.24
10.0
9.0
8.0
7.0
6.0
5.0
4.0
3.0
2.0
1.0
0
7.834
7.832
7.830
7.826
7.809
7.806
7.803
7.609
7.590
7.584
7.577
7.558
6.511
6.501
6.492
5.893
5.885
5.876
3.551
3.526
3.523
3.498
3.478
3.426
3.417
3.408
3.340
3.331
3.322
3.271
3.267
3.244
1.710
1.697
1.687
1.680
1.662
1.634
1.595
1.352
1.327
0.917
0.894
0.872
0.000
X : parts per Million : 1H

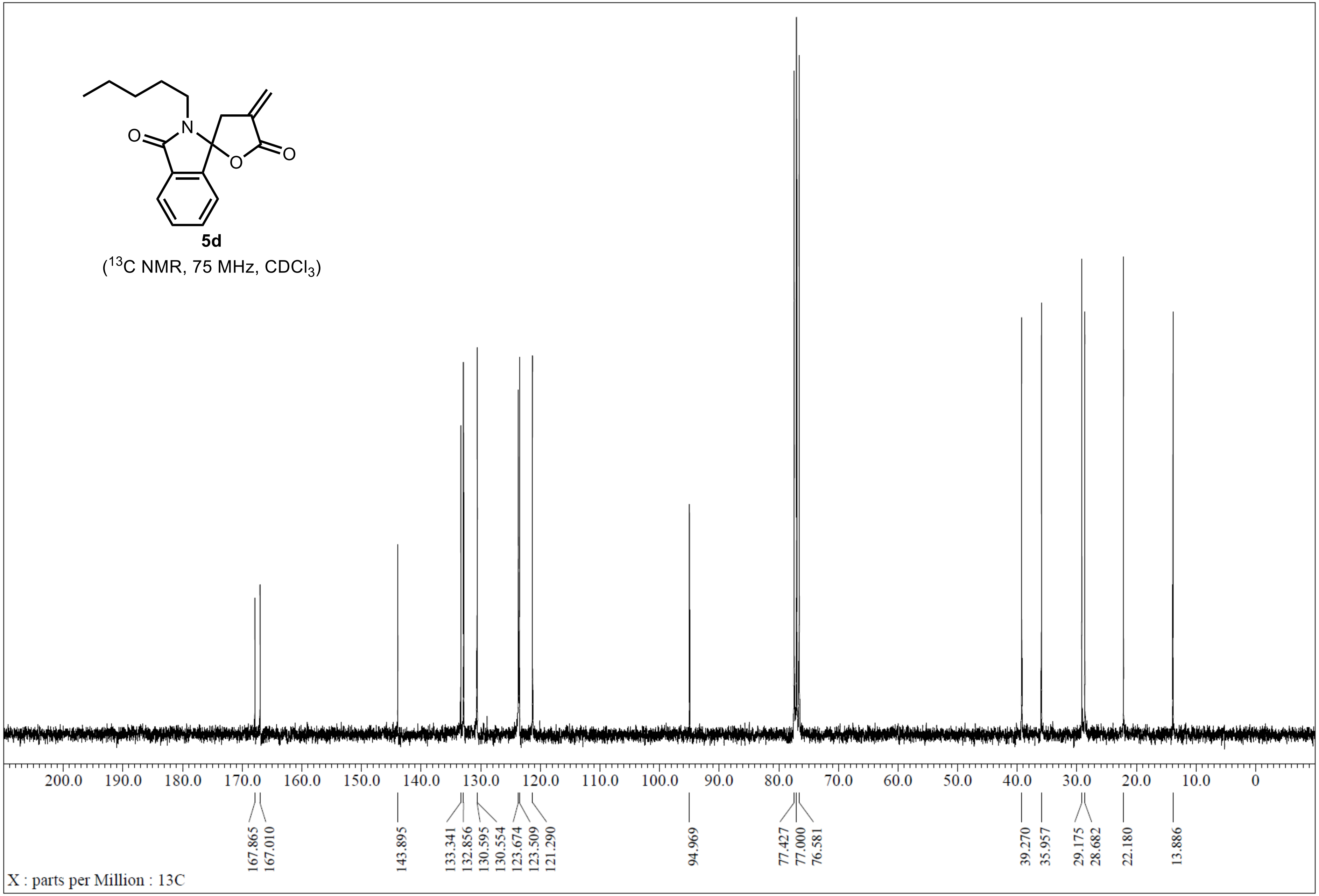
5d
(^{13}C NMR, 75 MHz, CDCl$_3$)
200.0
190.0
180.0
170.0
160.0
150.0
140.0
130.0
120.0
110.0
100.0
90.0
80.0
70.0
60.0
50.0
40.0
30.0
20.0
10.0
0
167.865
167.010
143.895
133.341
132.856
130.595
130.554
123.674
123.509
121.290
94.969
77.427
77.000
76.581
39.270
35.957
29.175
28.682
22.180
13.886
X : parts per Million : 13C

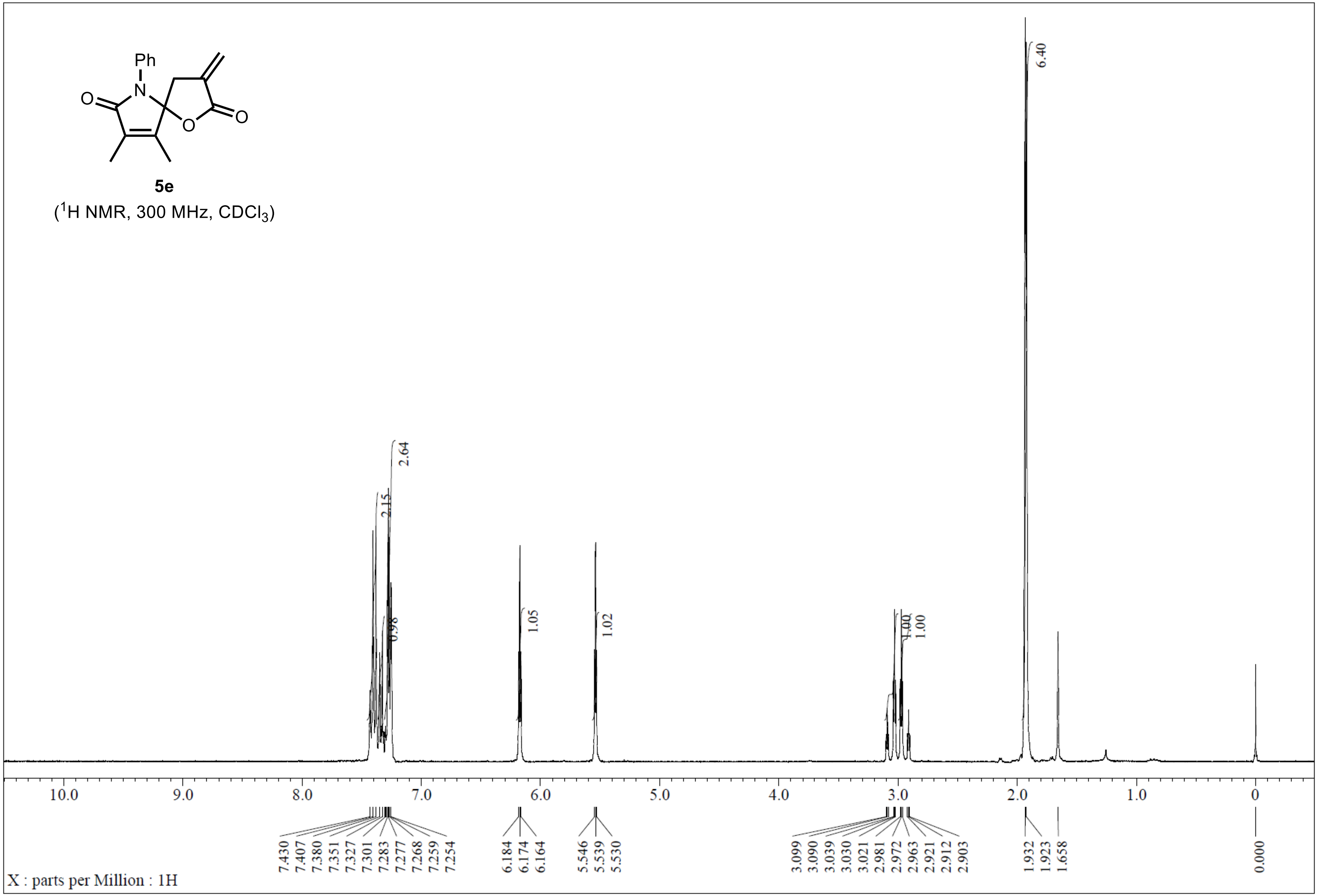
Ph
N
O
O
O
5e
(^{1}H NMR, 300 MHz, $CDCl_3$)
6.40
2.64
2.15
0.98
1.05
1.02
1.00
1.00
10.0
9.0
8.0
7.0
6.0
5.0
4.0
3.0
2.0
1.0
0
7.430
7.407
7.380
7.351
7.327
7.301
7.283
7.277
7.268
7.259
7.254
6.184
6.174
6.164
5.546
5.539
5.530
3.099
3.090
3.039
3.030
3.021
2.981
2.972
2.963
2.921
2.912
2.903
1.932
1.923
1.658
0.000
X : parts per Million : 1H

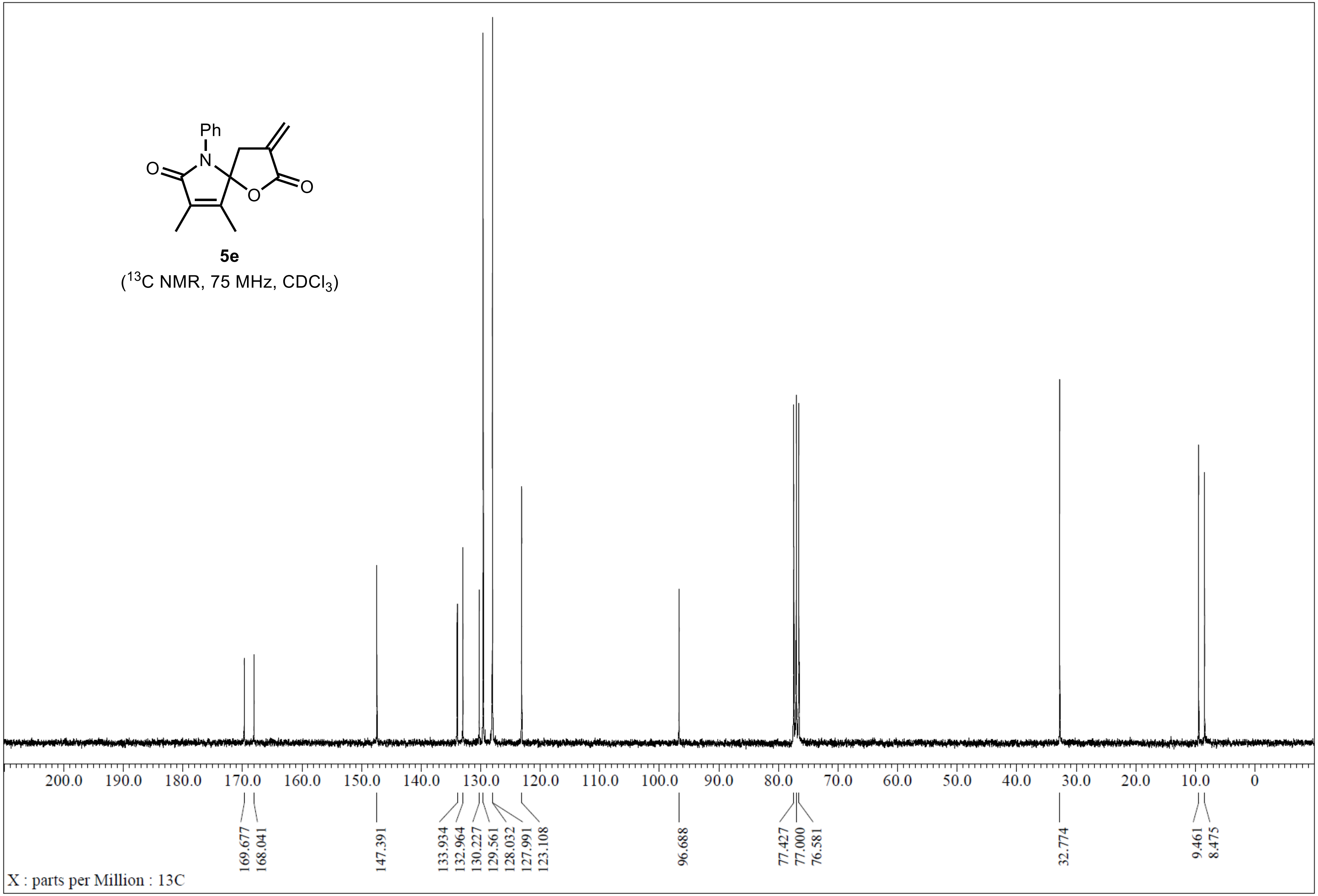

Ph
N
O
O
O
5e
(13C NMR, 75 MHz, CDCl3)
169.677
168.041
147.391
133.934
132.964
130.227
129.561
128.032
127.991
123.108
96.688
77.427
77.000
76.581
32.774
9.461
8.475
X : parts per Million : 13C

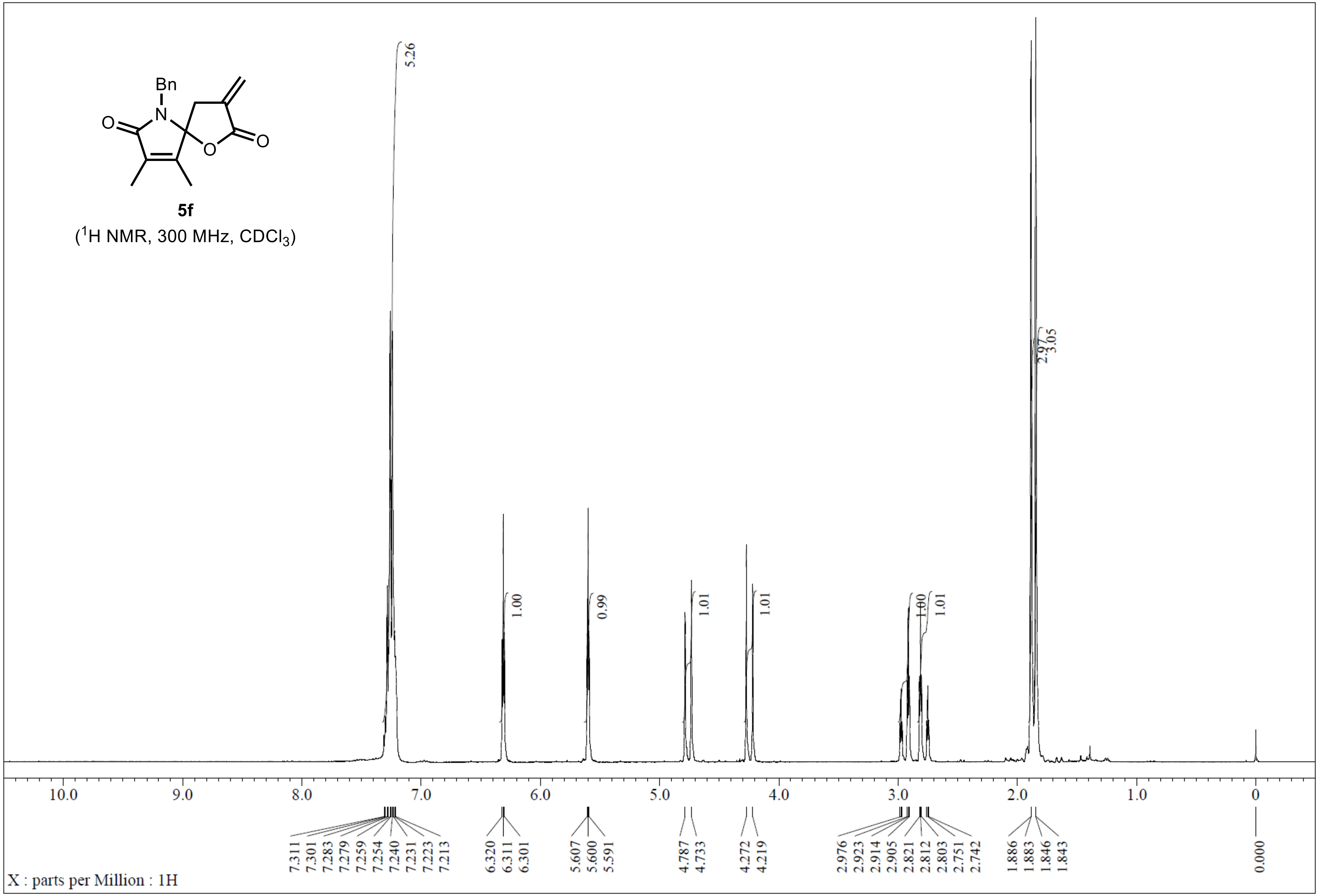
Bn
N
O
O
O
5f
(1H NMR, 300 MHz, CDCl3)
5.26
1.00
0.99
1.01
1.01
1.00
1.01
2.97
3.05
10.0
9.0
8.0
7.0
6.0
5.0
4.0
3.0
2.0
1.0
0
7.311
7.301
7.283
7.279
7.259
7.254
7.240
7.231
7.223
7.213
6.320
6.311
6.301
5.607
5.600
5.591
4.787
4.733
4.272
4.219
2.976
2.923
2.914
2.905
2.821
2.812
2.803
2.751
2.742
1.886
1.883
1.846
1.843
0.000
X : parts per Million : 1H

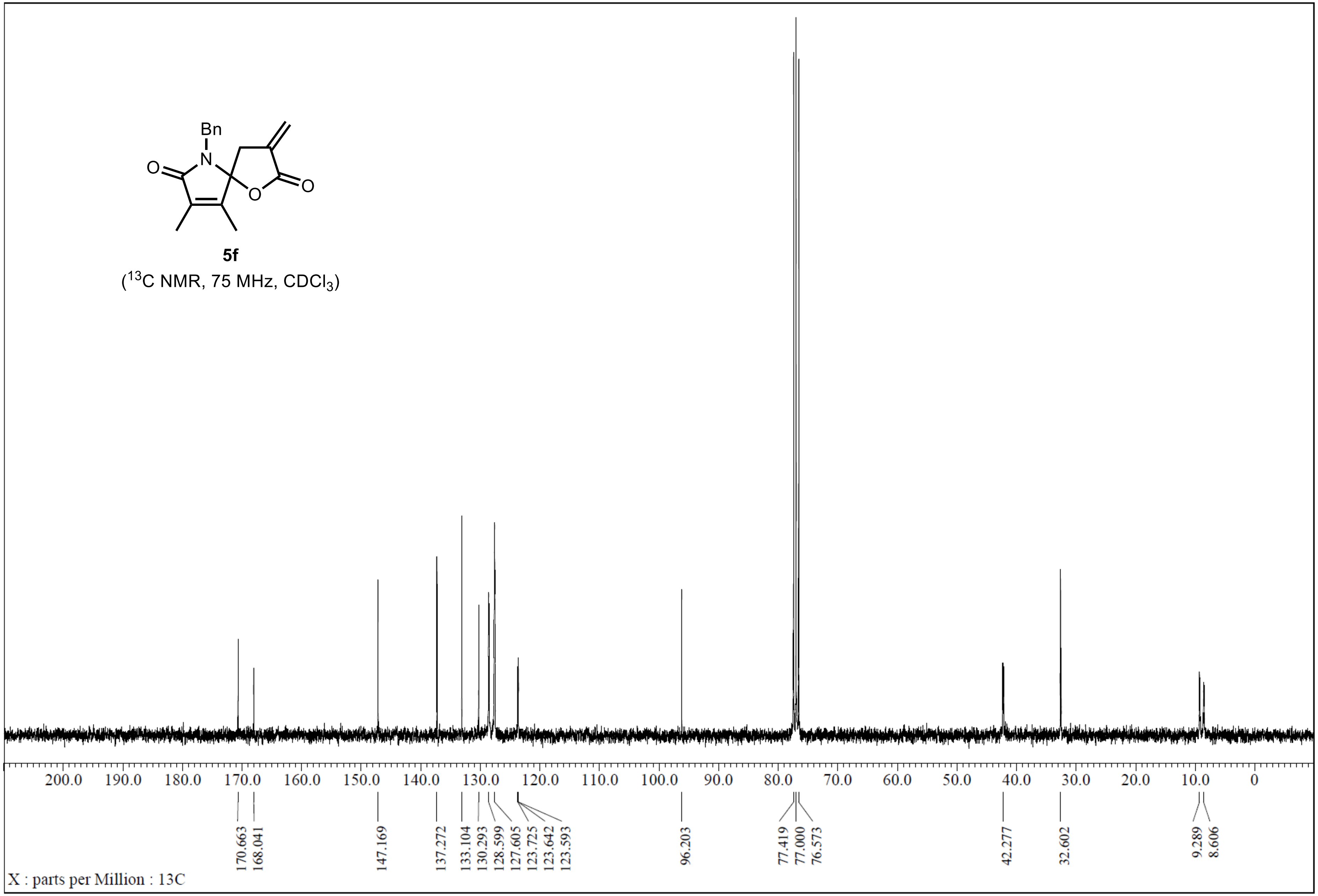
Bn
N
O
O
O
5f
(^{13}C NMR, 75 MHz, $CDCl_3$)
200.0 190.0 180.0 170.0 160.0 150.0 140.0 130.0 120.0 110.0 100.0 90.0 80.0 70.0 60.0 50.0 40.0 30.0 20.0 10.0 0
170.663
168.041
147.169
137.272
133.104
130.293
128.599
127.605
123.725
123.642
123.593
96.203
77.419
77.000
76.573
42.277
32.602
9.289
8.606
X : parts per Million : 13C

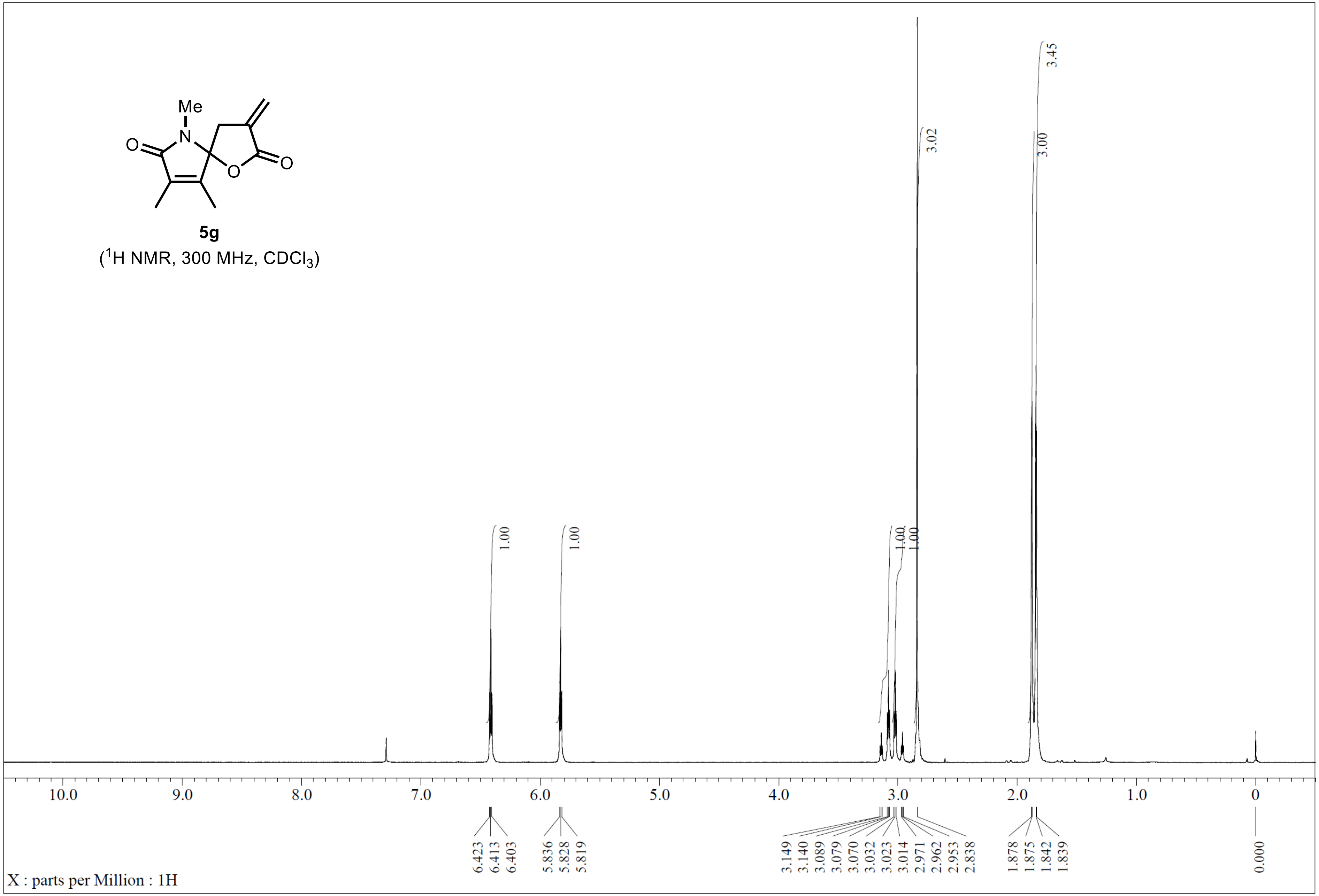
Me
N
O
O
O
5g
(1H NMR, 300 MHz, CDCl3)
3.45
3.02
3.00
1.00
1.00
1.00
1.00
10.0
9.0
8.0
7.0
6.0
5.0
4.0
3.0
2.0
1.0
0
6.423
6.413
6.403
5.836
5.828
5.819
3.149
3.140
3.089
3.079
3.070
3.032
3.023
3.014
2.971
2.962
2.953
2.838
1.878
1.875
1.842
1.839
0.000
X : parts per Million : 1H

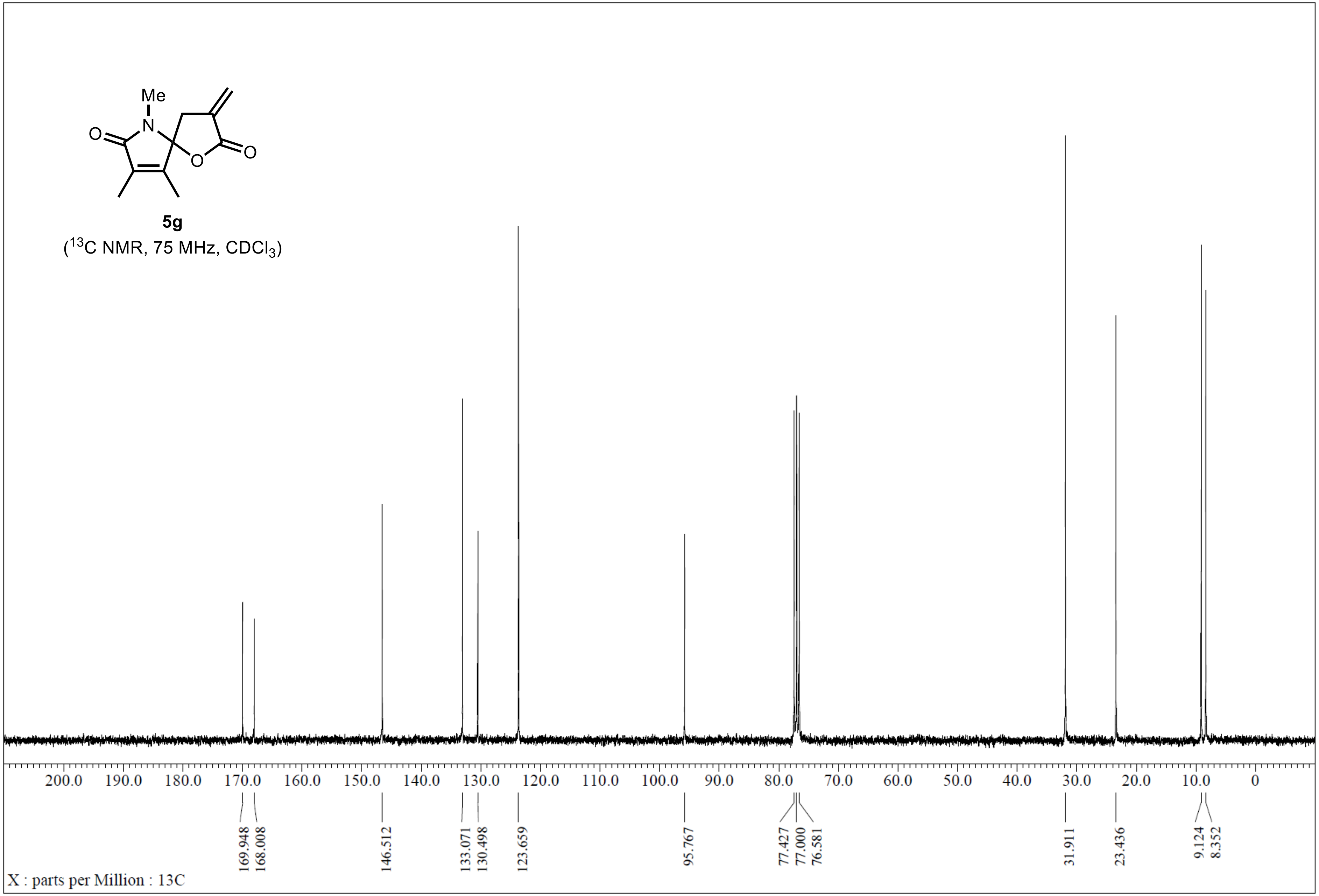
Me
N
O
O
O
5g
(^{13}C NMR, 75 MHz, CDCl$_3$)
200.0 190.0 180.0 170.0 160.0 150.0 140.0 130.0 120.0 110.0 100.0 90.0 80.0 70.0 60.0 50.0 40.0 30.0 20.0 10.0 0
169.948
168.008
146.512
133.071
130.498
123.659
95.767
77.427
77.000
76.581
31.911
23.436
9.124
8.352
X : parts per Million : 13C

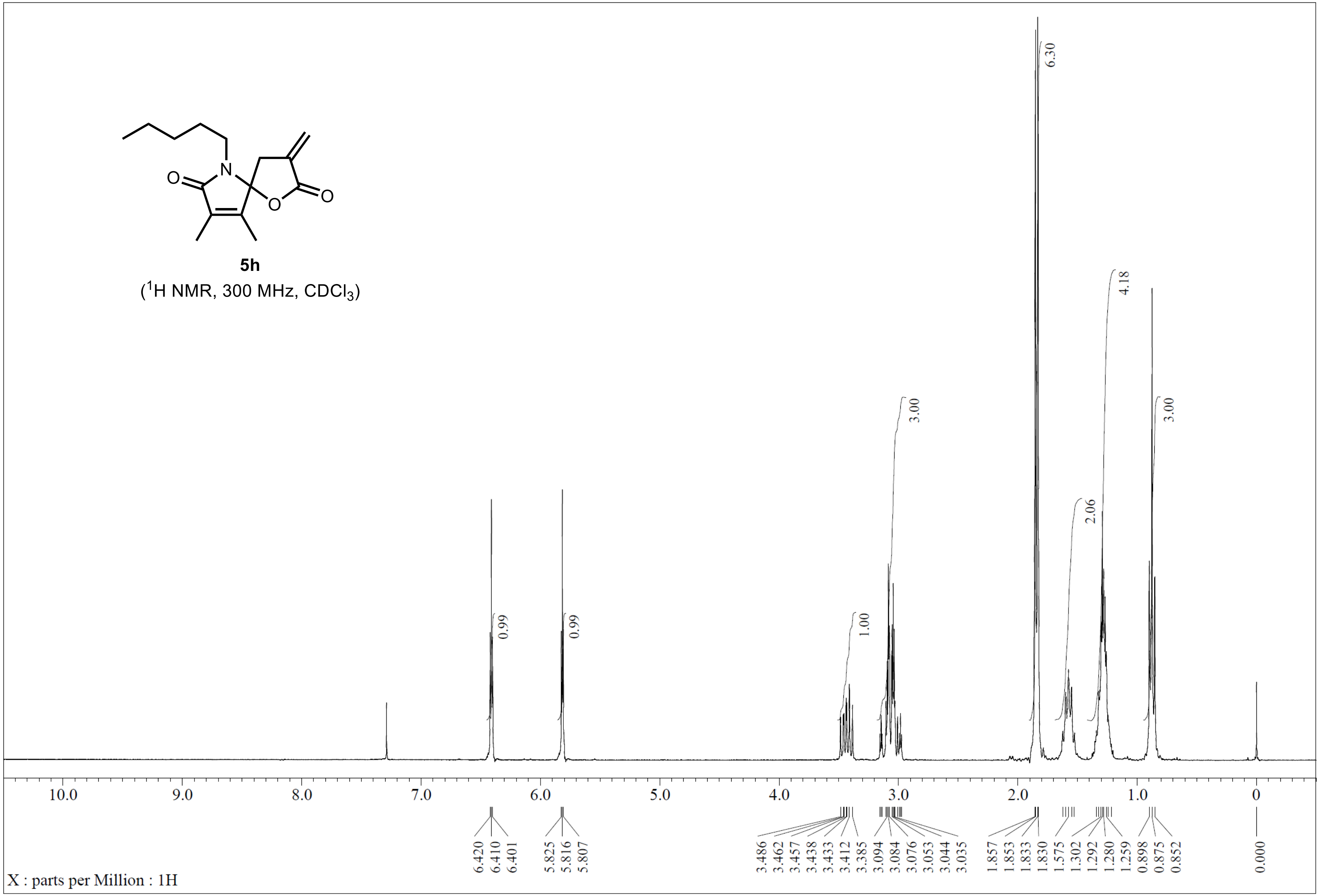
O
N
O
O
5h
(1H NMR, 300 MHz, CDCl3)
6.30
4.18
3.00
3.00
2.06
1.00
0.99
0.99
10.0
9.0
8.0
7.0
6.0
5.0
4.0
3.0
2.0
1.0
0
6.420
6.410
6.401
5.825
5.816
5.807
3.486
3.462
3.457
3.438
3.433
3.412
3.385
3.094
3.084
3.076
3.053
3.044
3.035
1.857
1.853
1.833
1.830
1.575
1.302
1.292
1.280
1.259
0.898
0.875
0.852
0.000
X : parts per Million : 1H

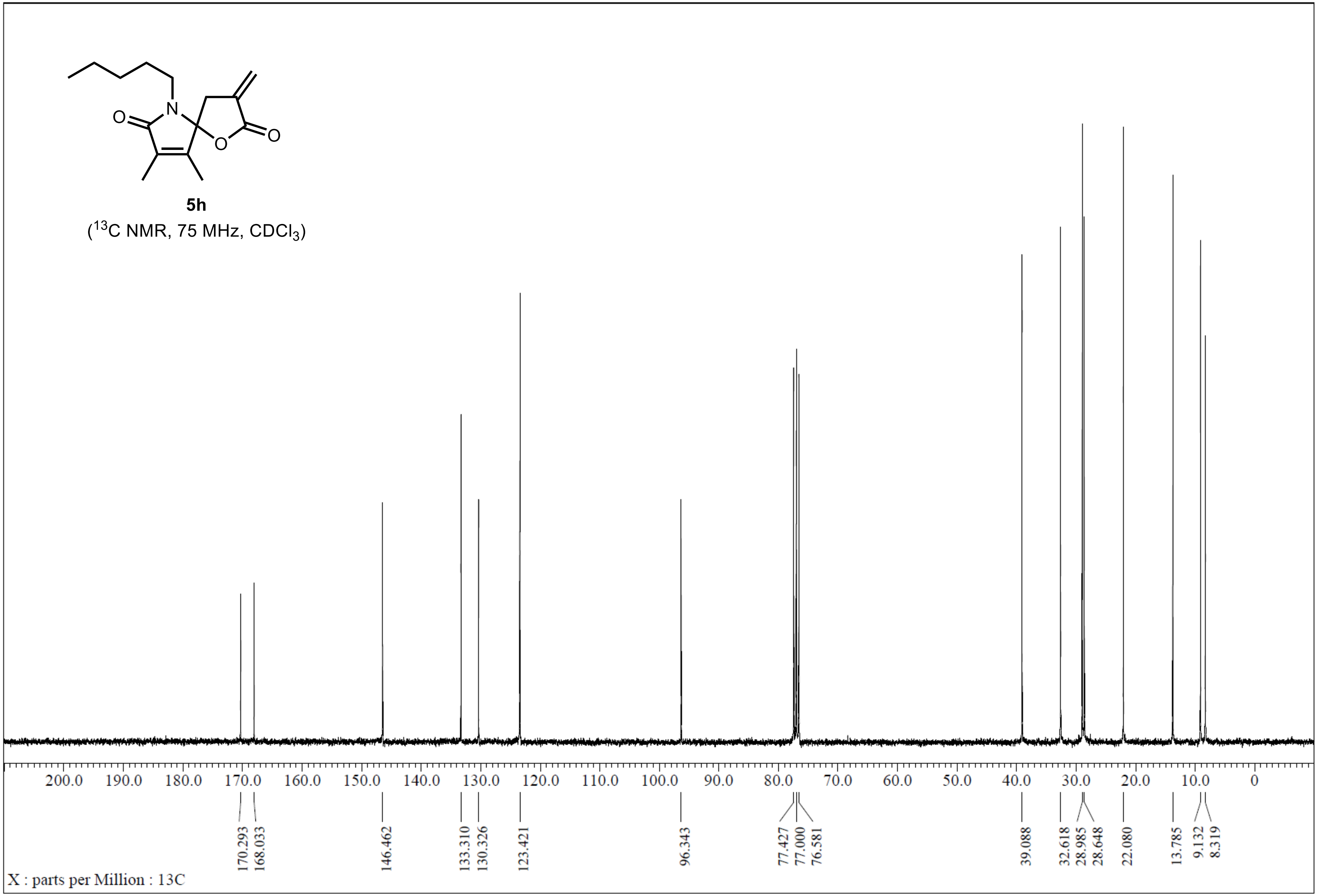
O
N
O
O
5h
(13C NMR, 75 MHz, CDCl3)
200.0 190.0 180.0 170.0 160.0 150.0 140.0 130.0 120.0 110.0 100.0 90.0 80.0 70.0 60.0 50.0 40.0 30.0 20.0 10.0 0
170.293
168.033
146.462
133.310
130.326
123.421
96.343
77.427
77.000
76.581
39.088
32.618
28.985
28.648
22.080
13.785
9.132
8.319
X : parts per Million : 13C

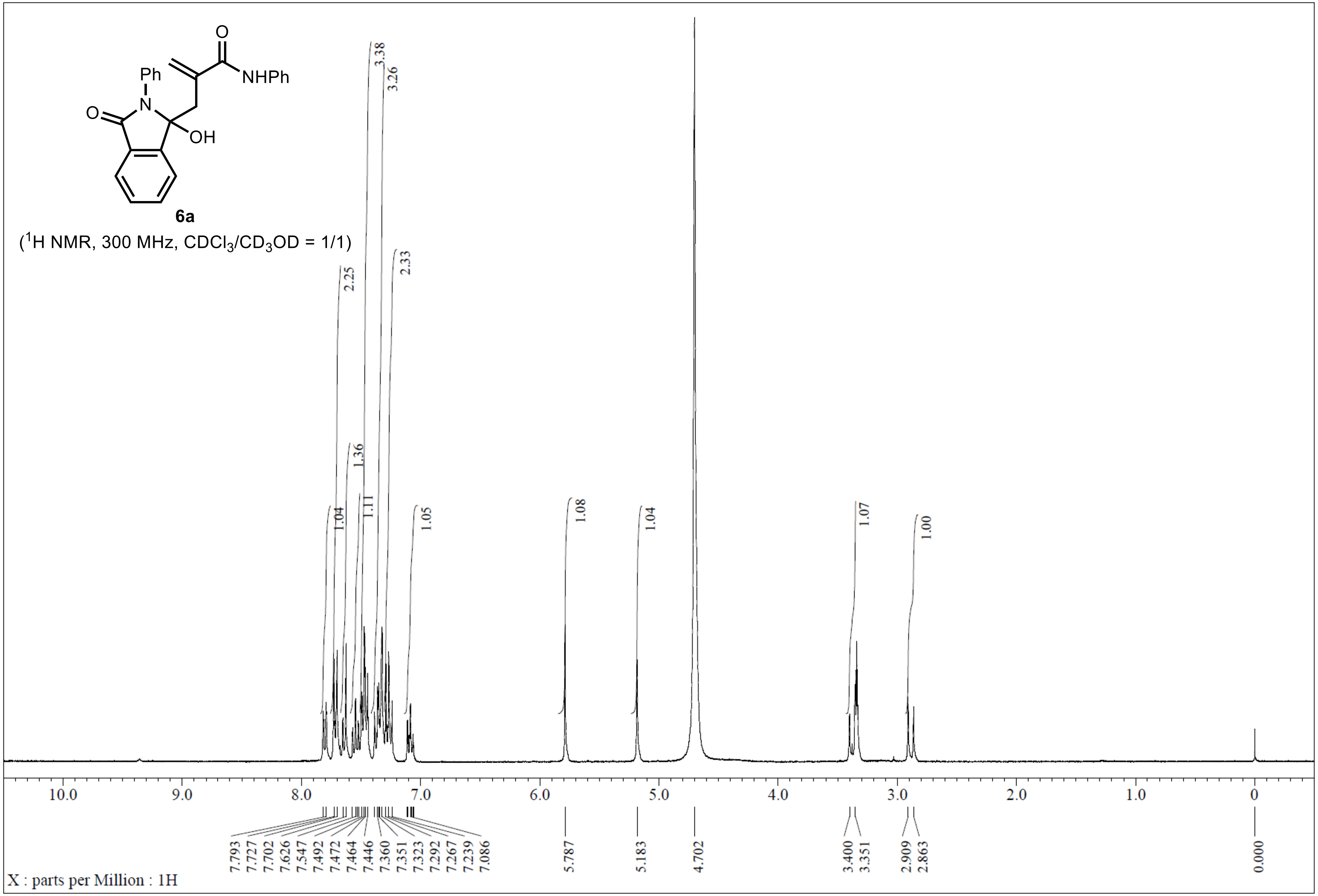
O
Ph
NHPh
O
N
OH
6a
(1H NMR, 300 MHz, CDCl3/CD3OD = 1/1)
3.38
3.26
2.25
2.33
1.36
1.04
1.11
1.05
1.08
1.04
1.07
1.00
10.0
9.0
8.0
7.0
6.0
5.0
4.0
3.0
2.0
1.0
0
7.793
7.727
7.702
7.626
7.547
7.492
7.472
7.464
7.446
7.360
7.351
7.323
7.292
7.267
7.239
7.086
5.787
5.183
4.702
3.400
3.351
2.909
2.863
0.000
X : parts per Million : 1H

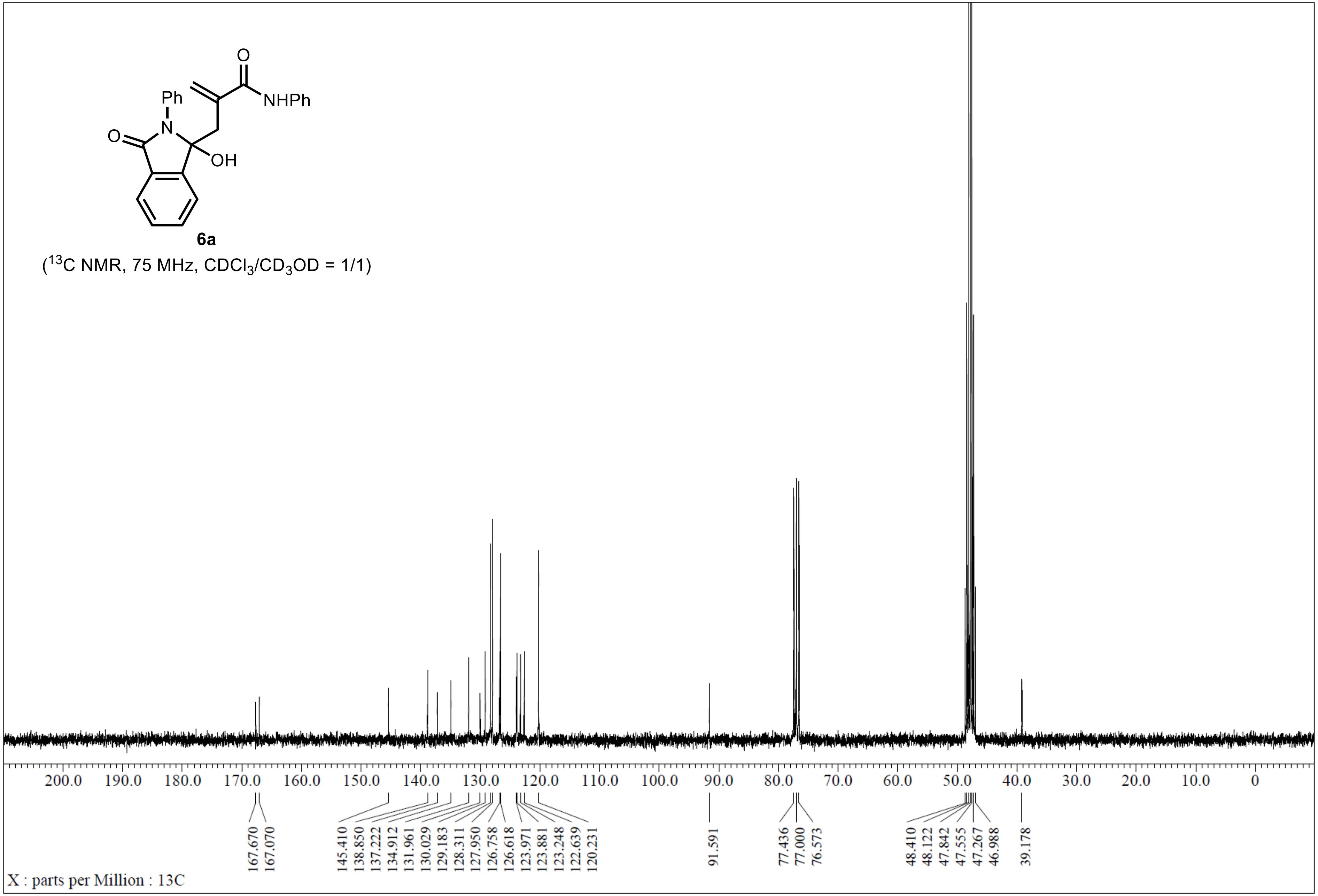
Ph
N
O
OH
O
NHPh
6a
(13C NMR, 75 MHz, CDCl3/CD3OD = 1/1)
200.0 190.0 180.0 170.0 160.0 150.0 140.0 130.0 120.0 110.0 100.0 90.0 80.0 70.0 60.0 50.0 40.0 30.0 20.0 10.0 0
167.670
167.070
145.410
138.850
137.222
134.912
131.961
130.029
129.183
128.311
127.950
126.758
126.618
123.971
123.881
123.248
122.639
120.231
91.591
77.436
77.000
76.573
48.410
48.122
47.842
47.555
47.267
46.988
39.178
X : parts per Million : 13C

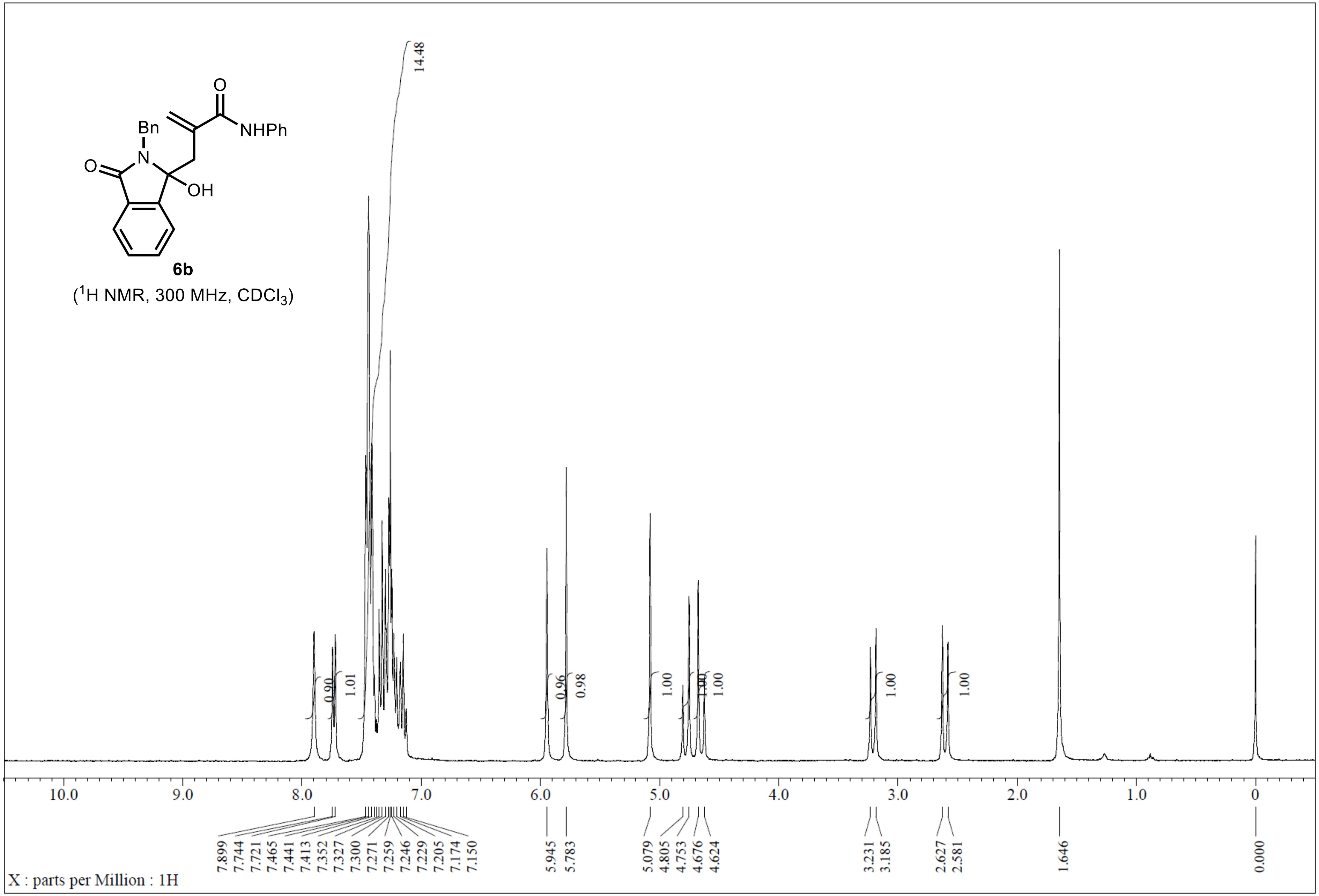
Bn
O
NHPh
OH
6b
(1H NMR, 300 MHz, CDCl3)
14.48
0.90
1.01
0.96
0.98
1.00
1.00
1.00
1.00
1.00
10.0
9.0
8.0
7.0
6.0
5.0
4.0
3.0
2.0
1.0
0
7.899
7.744
7.721
7.465
7.441
7.413
7.352
7.327
7.300
7.271
7.259
7.246
7.229
7.205
7.174
7.150
5.945
5.783
5.079
4.805
4.753
4.676
4.624
3.231
3.185
2.627
2.581
1.646
0.000
X : parts per Million : 1H

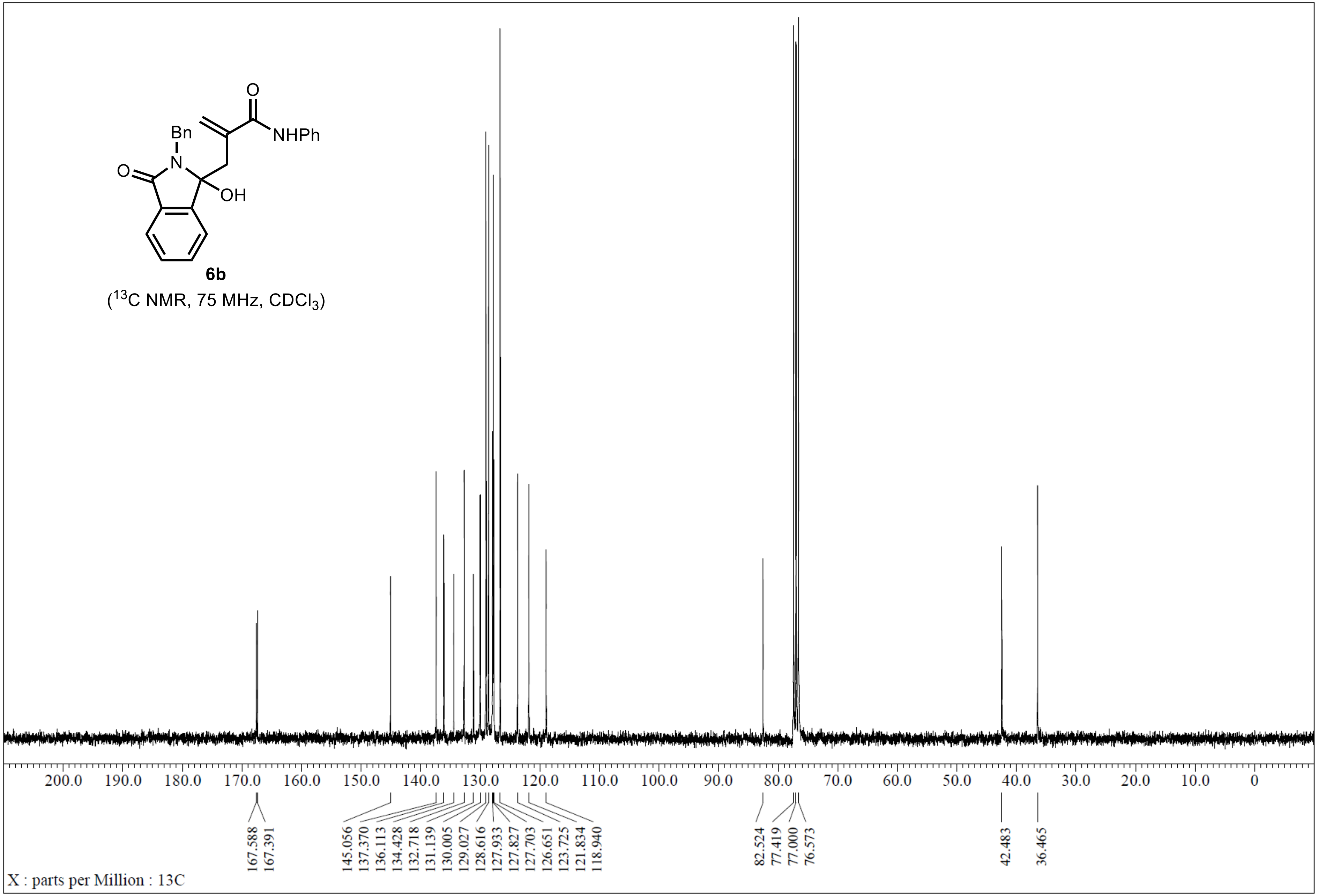
O
Bn
NHPh
O
N
OH
6b
(13C NMR, 75 MHz, CDCl3)
200.0 190.0 180.0 170.0 160.0 150.0 140.0 130.0 120.0 110.0 100.0 90.0 80.0 70.0 60.0 50.0 40.0 30.0 20.0 10.0 0
167.588
167.391
145.056
137.370
136.113
134.428
132.718
131.139
130.005
129.027
128.616
127.933
127.827
127.703
126.651
123.725
121.834
118.940
82.524
77.419
77.000
76.573
42.483
36.465
X : parts per Million : 13C

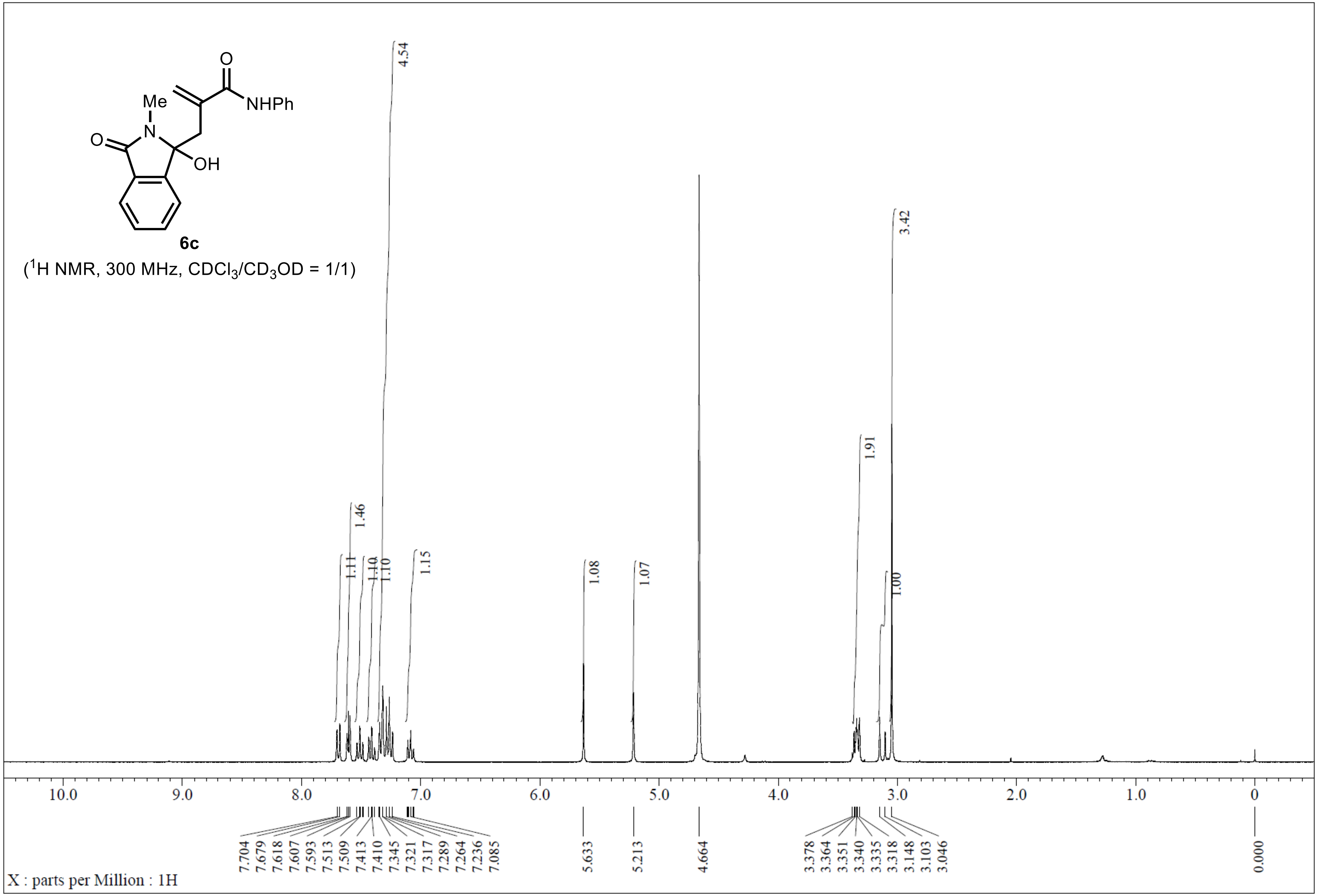
O
Me
N
NHPh
O
OH
6c
(1H NMR, 300 MHz, CDCl3/CD3OD = 1/1)
4.54
3.42
1.91
1.46
1.11
1.10
1.10
1.15
1.08
1.07
1.00
10.0
9.0
8.0
7.0
6.0
5.0
4.0
3.0
2.0
1.0
0
7.704
7.679
7.618
7.607
7.593
7.513
7.509
7.413
7.410
7.345
7.321
7.317
7.289
7.264
7.236
7.085
5.633
5.213
4.664
3.378
3.364
3.351
3.340
3.335
3.318
3.148
3.103
3.046
0.000
X : parts per Million : 1H

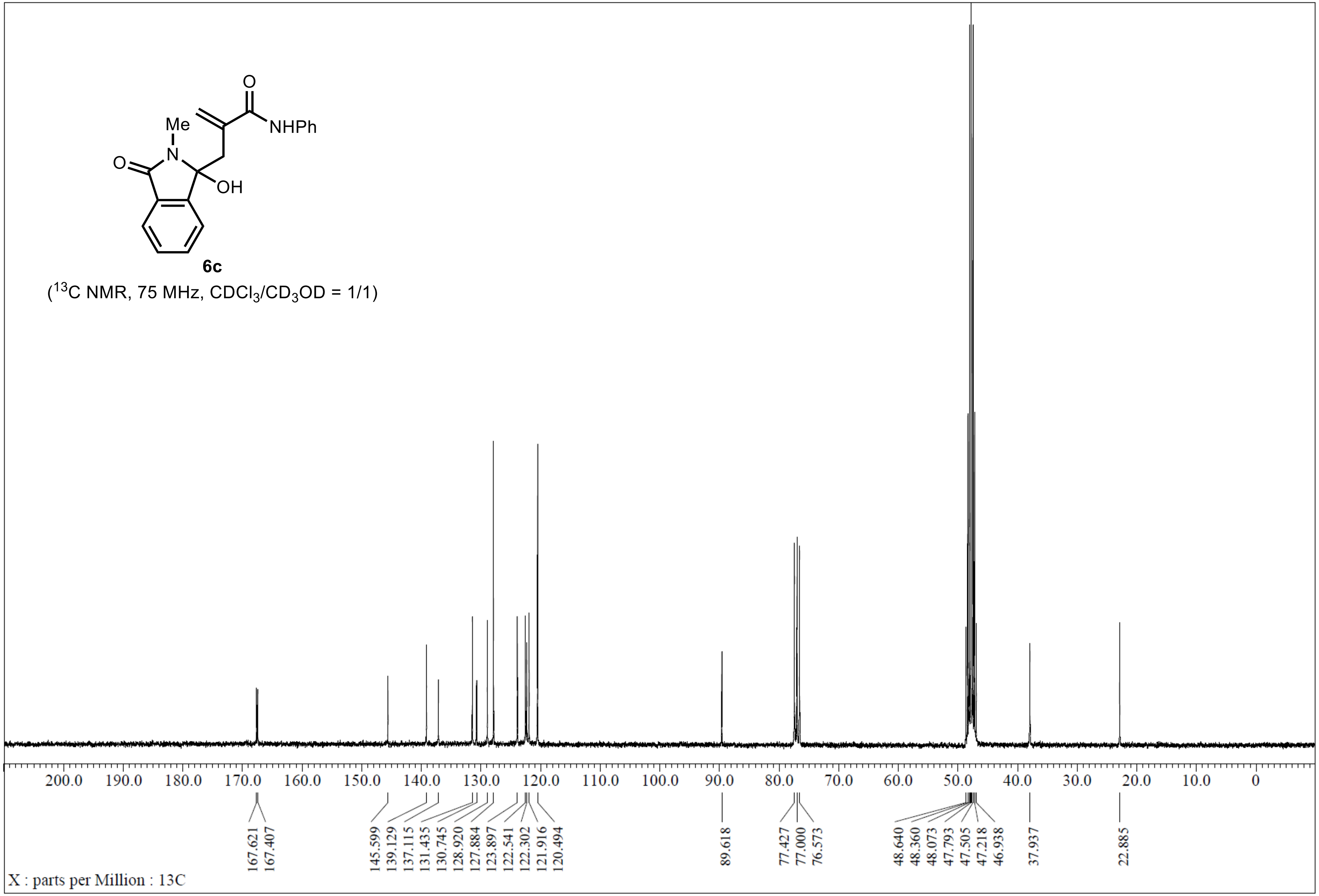
O
Me
NHPh
O
N
OH
6c
(^{13}C NMR, 75 MHz, $CDCl_3/CD_3OD$ = 1/1)
200.0 190.0 180.0 170.0 160.0 150.0 140.0 130.0 120.0 110.0 100.0 90.0 80.0 70.0 60.0 50.0 40.0 30.0 20.0 10.0 0
167.621
167.407
145.599
139.129
137.115
131.435
130.745
128.920
127.884
123.897
122.541
122.302
121.916
120.494
89.618
77.427
77.000
76.573
48.640
48.360
48.073
47.793
47.505
47.218
46.938
37.937
22.885
X : parts per Million : 13C

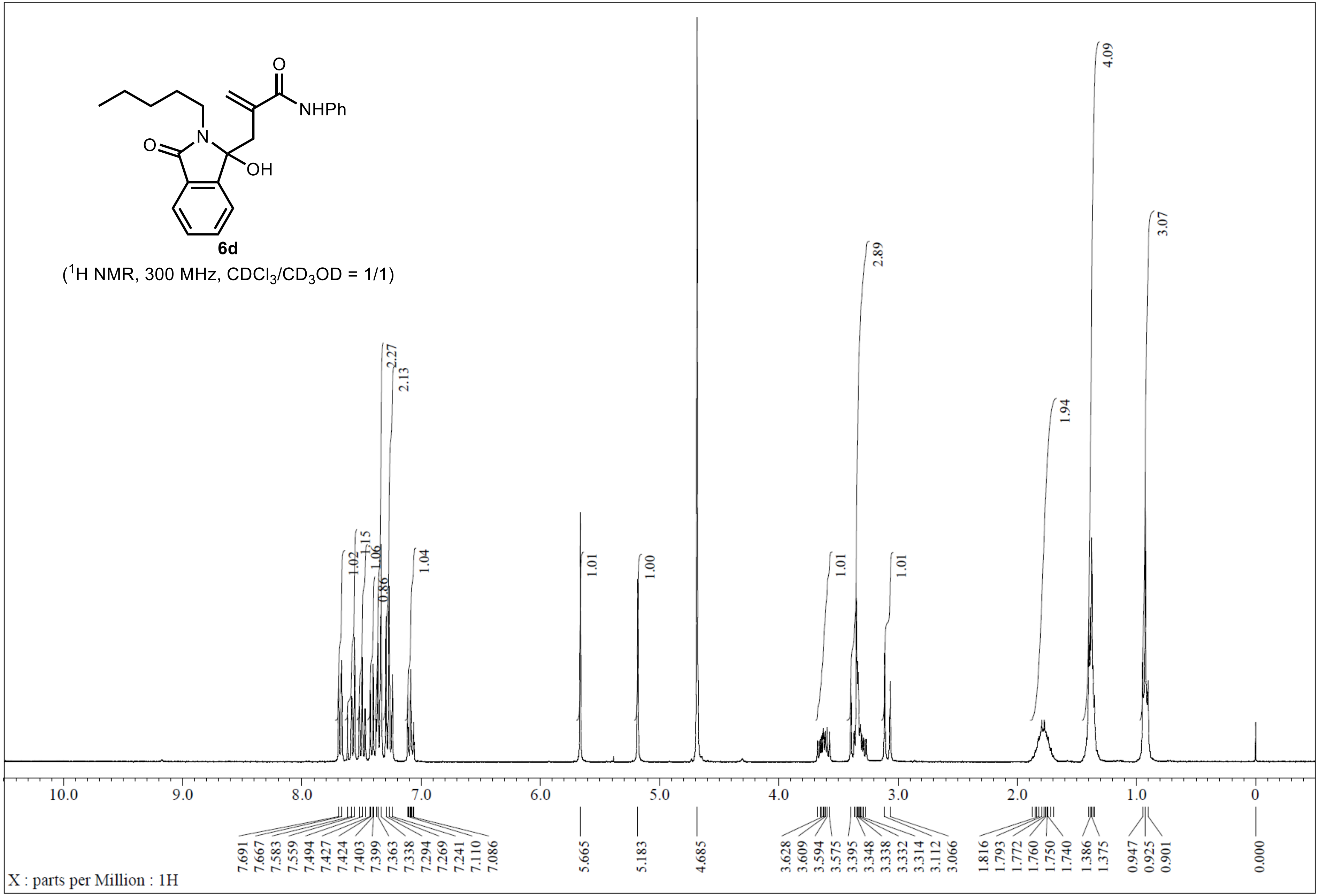
O
NHPh
O
N
OH
6d
(¹H NMR, 300 MHz, CDCl₃/CD₃OD = 1/1)
X : parts per Million : 1H
10.0
9.0
8.0
7.0
6.0
5.0
4.0
3.0
2.0
1.0
0
1.02
1.15
1.06
0.86
2.27
2.13
1.04
1.01
1.00
1.01
2.89
1.01
1.94
4.09
3.07
7.691
7.667
7.583
7.559
7.494
7.427
7.424
7.403
7.399
7.363
7.338
7.294
7.269
7.241
7.110
7.086
5.665
5.183
4.685
3.628
3.609
3.594
3.575
3.395
3.348
3.338
3.332
3.314
3.112
3.066
1.816
1.793
1.772
1.760
1.750
1.740
1.386
1.375
0.947
0.925
0.901
0.000

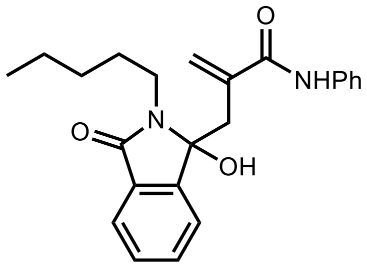

6d

(^{13}C NMR, 75 MHz, $CDCl_3/CD_3OD$ = 1/1)

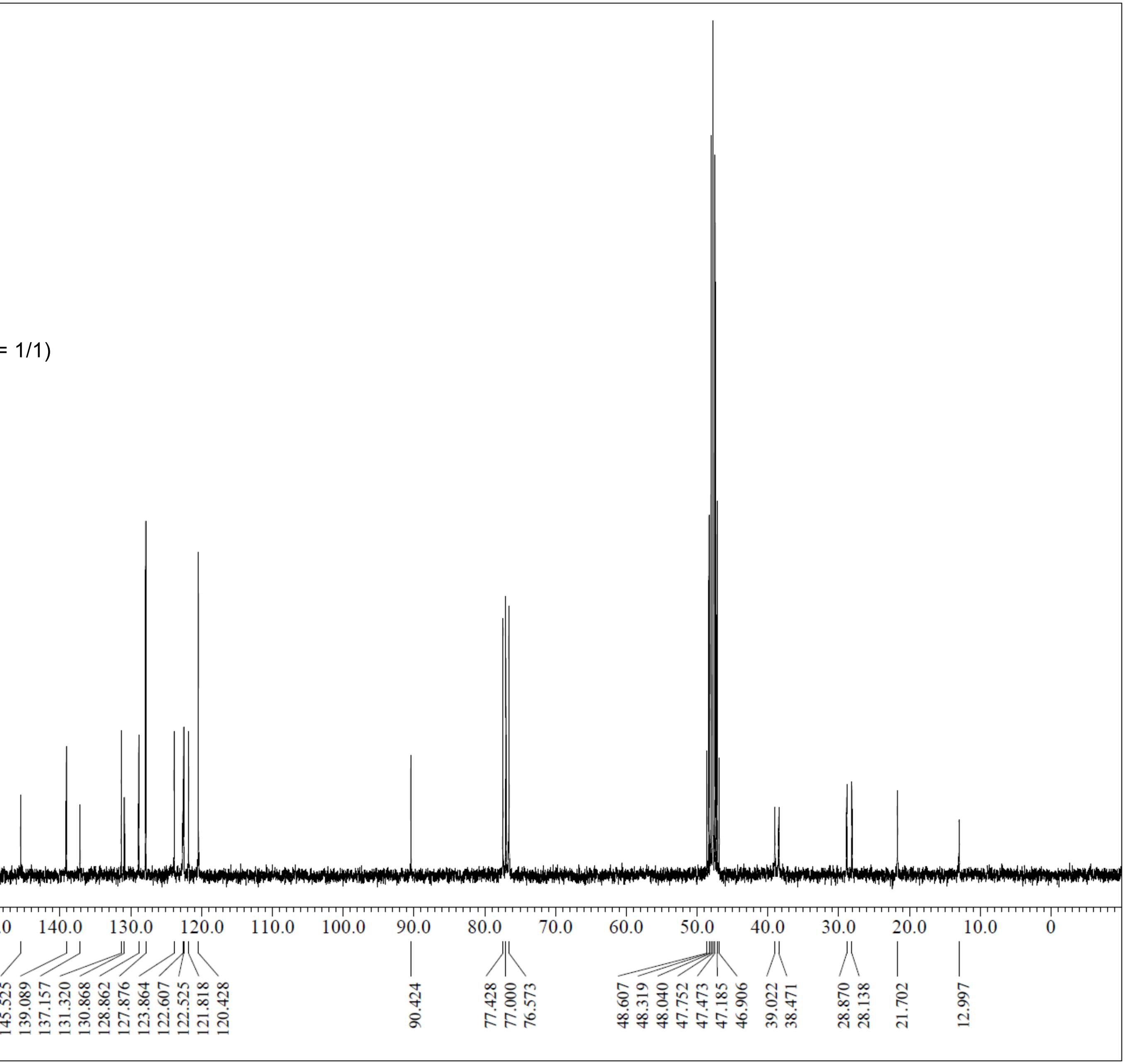

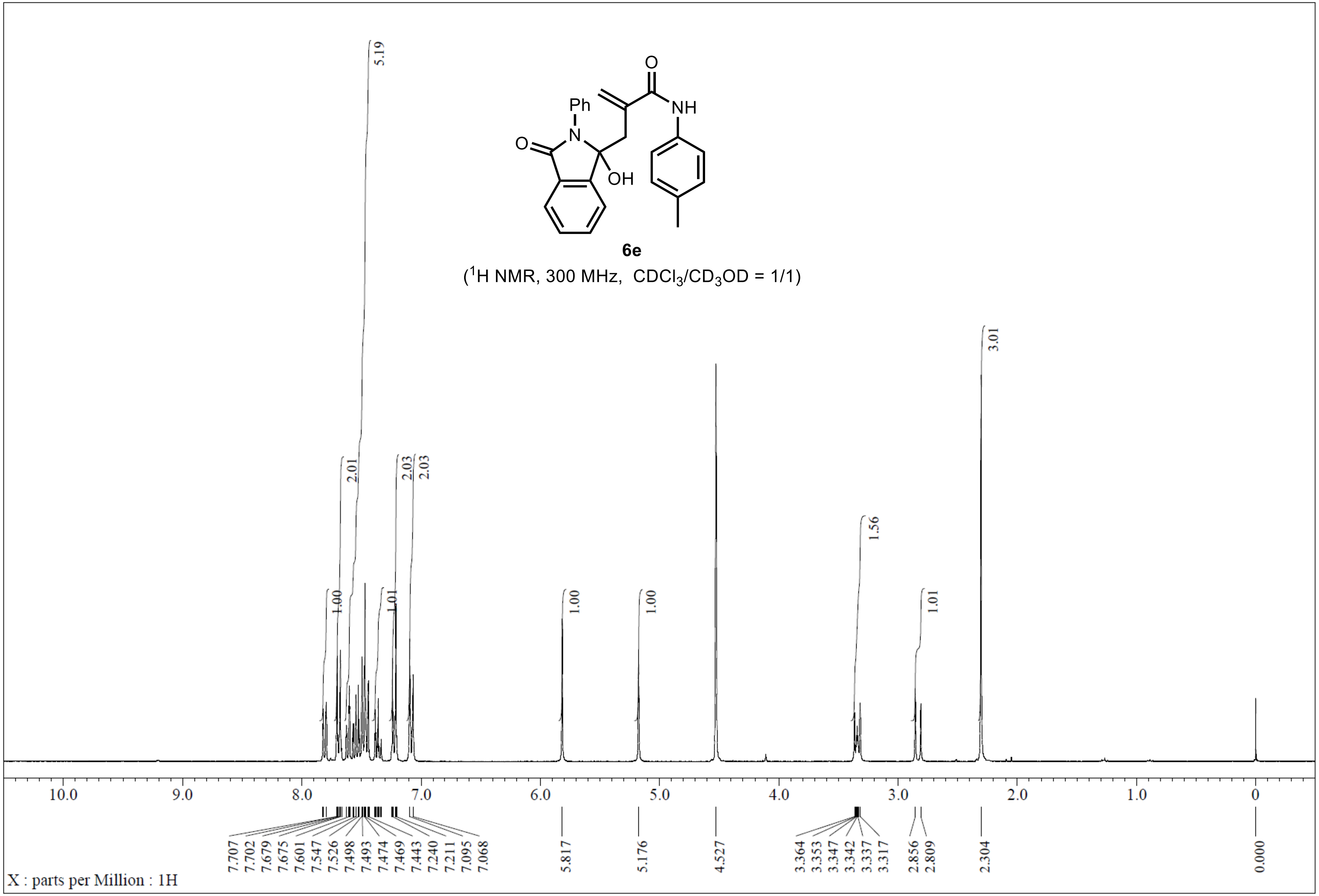
Ph
N
O
OH
O
NH
6e
(1H NMR, 300 MHz, CDCl3/CD3OD = 1/1)
5.19
2.01
1.00
1.01
2.03
2.03
1.00
1.00
1.56
1.01
3.01
10.0
9.0
8.0
7.0
6.0
5.0
4.0
3.0
2.0
1.0
0
7.707
7.702
7.679
7.675
7.601
7.547
7.526
7.498
7.493
7.474
7.469
7.443
7.240
7.211
7.095
7.068
5.817
5.176
4.527
3.364
3.353
3.347
3.342
3.337
3.317
2.856
2.809
2.304
0.000
X : parts per Million : 1H

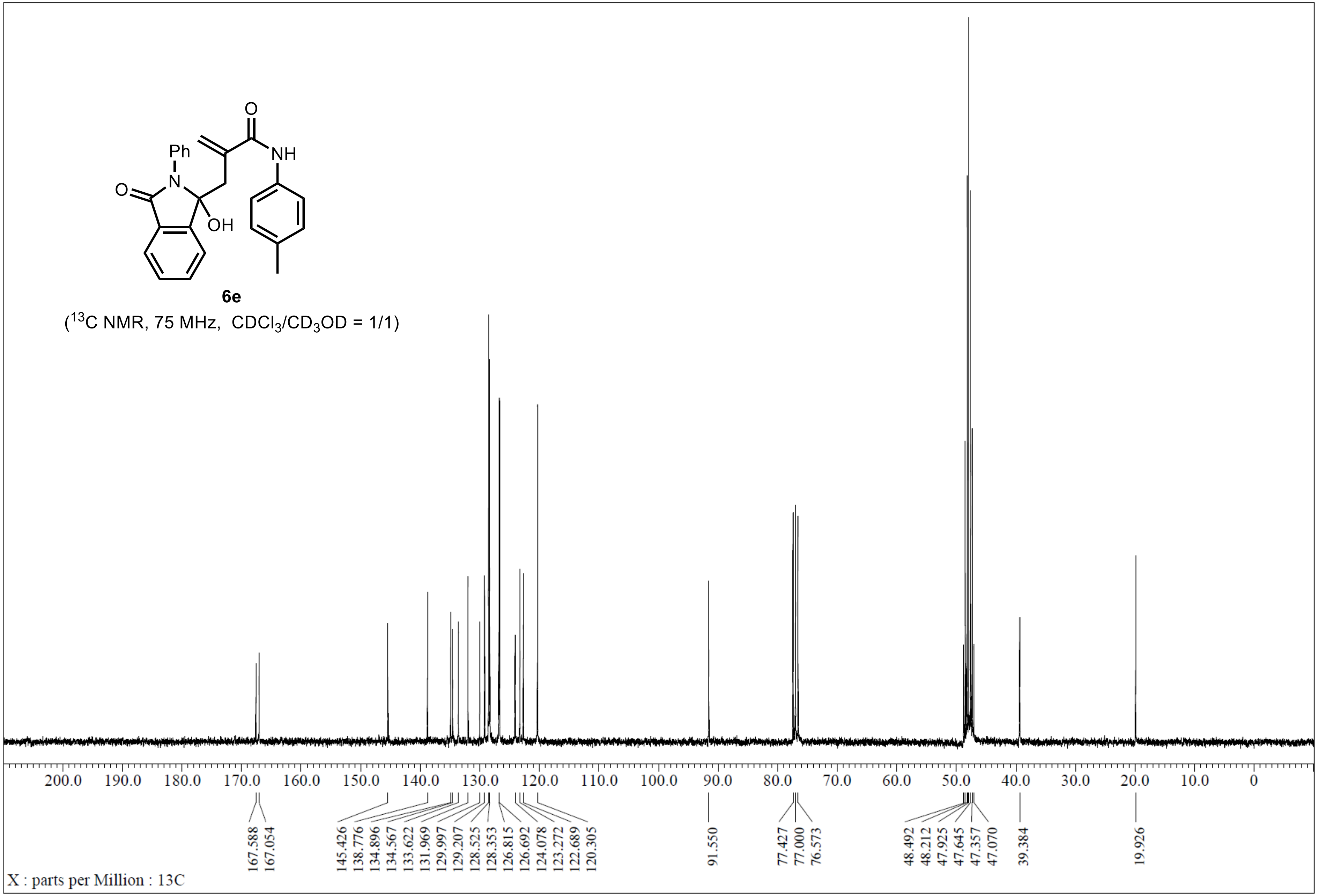
O
Ph
NH
N
O
OH
6e
(^{13}C NMR, 75 MHz, $CDCl_3/CD_3OD$ = 1/1)
200.0 190.0 180.0 170.0 160.0 150.0 140.0 130.0 120.0 110.0 100.0 90.0 80.0 70.0 60.0 50.0 40.0 30.0 20.0 10.0 0
167.588
167.054
145.426
138.776
134.896
134.567
133.622
131.969
129.997
129.207
128.525
128.353
126.815
126.692
124.078
123.272
122.689
120.305
91.550
77.427
77.000
76.573
48.492
48.212
47.925
47.645
47.357
47.070
39.384
19.926
X : parts per Million : 13C

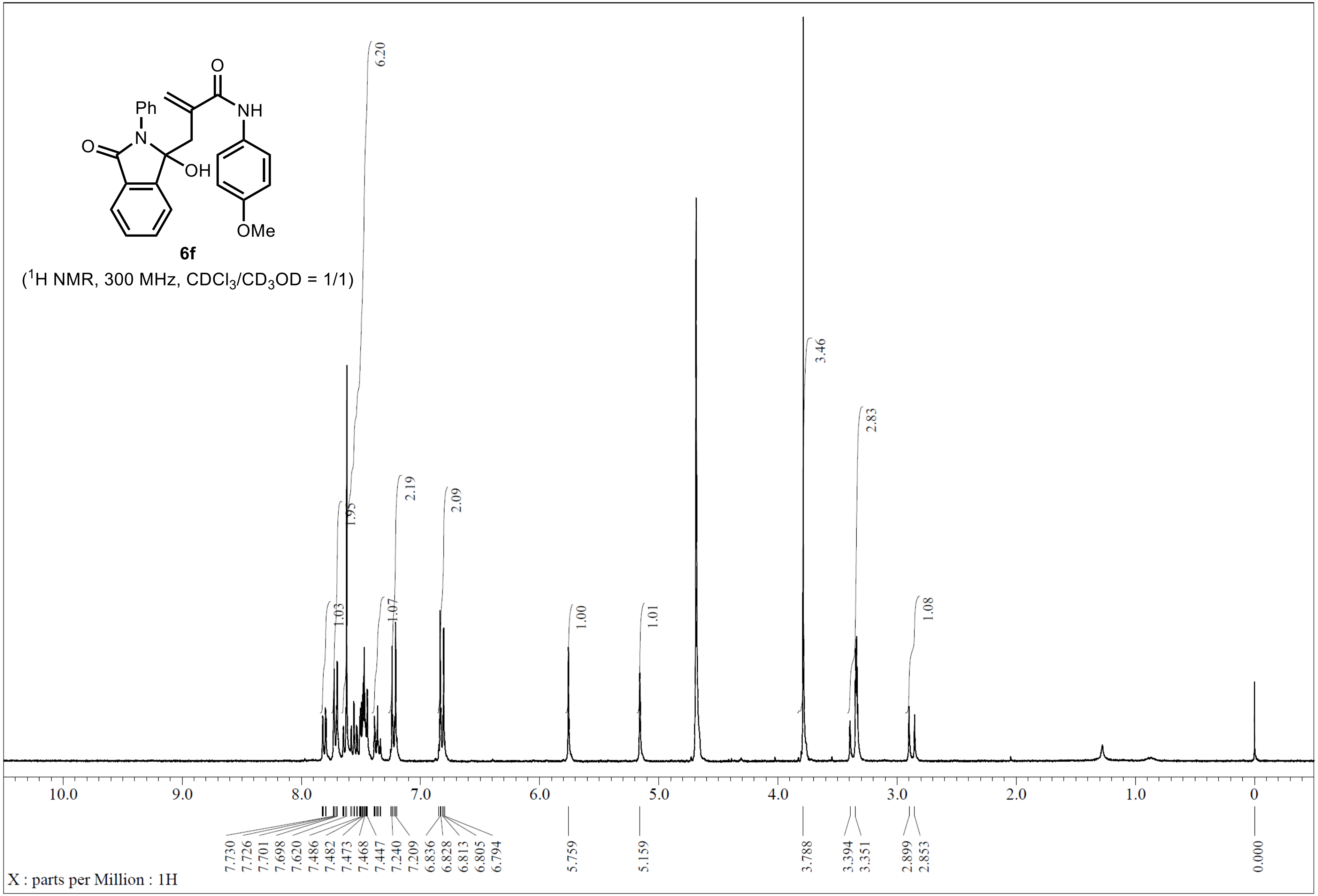

6f

(^{1}H NMR, 300 MHz, $CDCl_3/CD_3OD$ = 1/1)

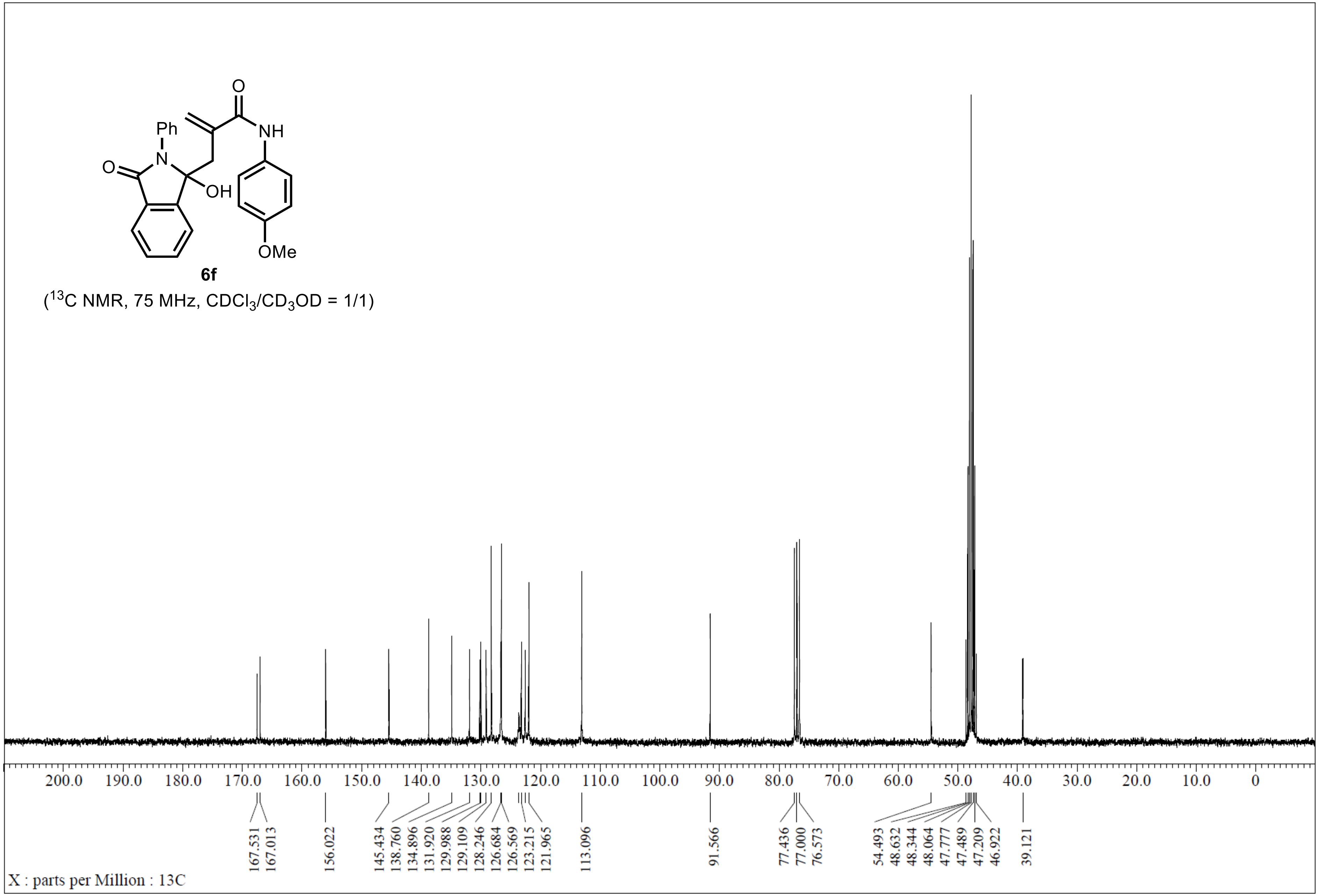
Ph
N
O
OH
O
NH
OMe
6f
(^{13}C NMR, 75 MHz, $CDCl_3/CD_3OD$ = 1/1)
200.0
190.0
180.0
170.0
160.0
150.0
140.0
130.0
120.0
110.0
100.0
90.0
80.0
70.0
60.0
50.0
40.0
30.0
20.0
10.0
0
167.531
167.013
156.022
145.434
138.760
134.896
131.920
129.988
129.109
128.246
126.684
126.569
123.215
121.965
113.096
91.566
77.436
77.000
76.573
54.493
48.632
48.344
48.064
47.777
47.489
47.209
46.922
39.121
X : parts per Million : 13C

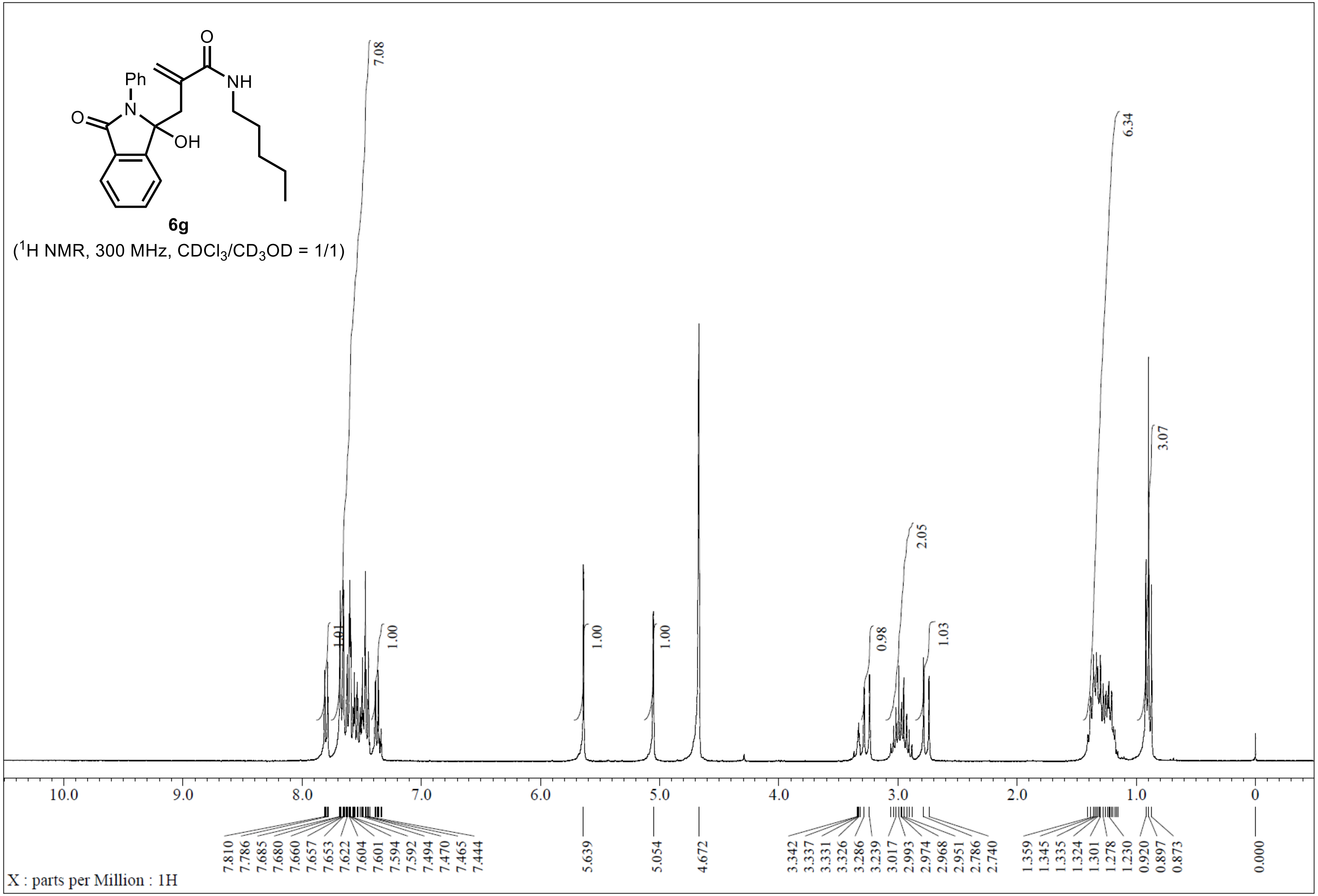
Ph
N
O
OH
O
NH
6g
(¹H NMR, 300 MHz, CDCl3/CD3OD = 1/1)
7.08
1.01
1.00
1.00
1.00
0.98
2.05
1.03
6.34
3.07
10.0
9.0
8.0
7.0
6.0
5.0
4.0
3.0
2.0
1.0
0
7.810
7.786
7.685
7.680
7.660
7.657
7.653
7.622
7.604
7.601
7.594
7.592
7.494
7.470
7.465
7.444
5.639
5.054
4.672
3.342
3.337
3.331
3.326
3.286
3.239
3.017
2.993
2.974
2.968
2.951
2.786
2.740
1.359
1.345
1.335
1.324
1.301
1.278
1.230
0.920
0.897
0.873
0.000
X : parts per Million : 1H

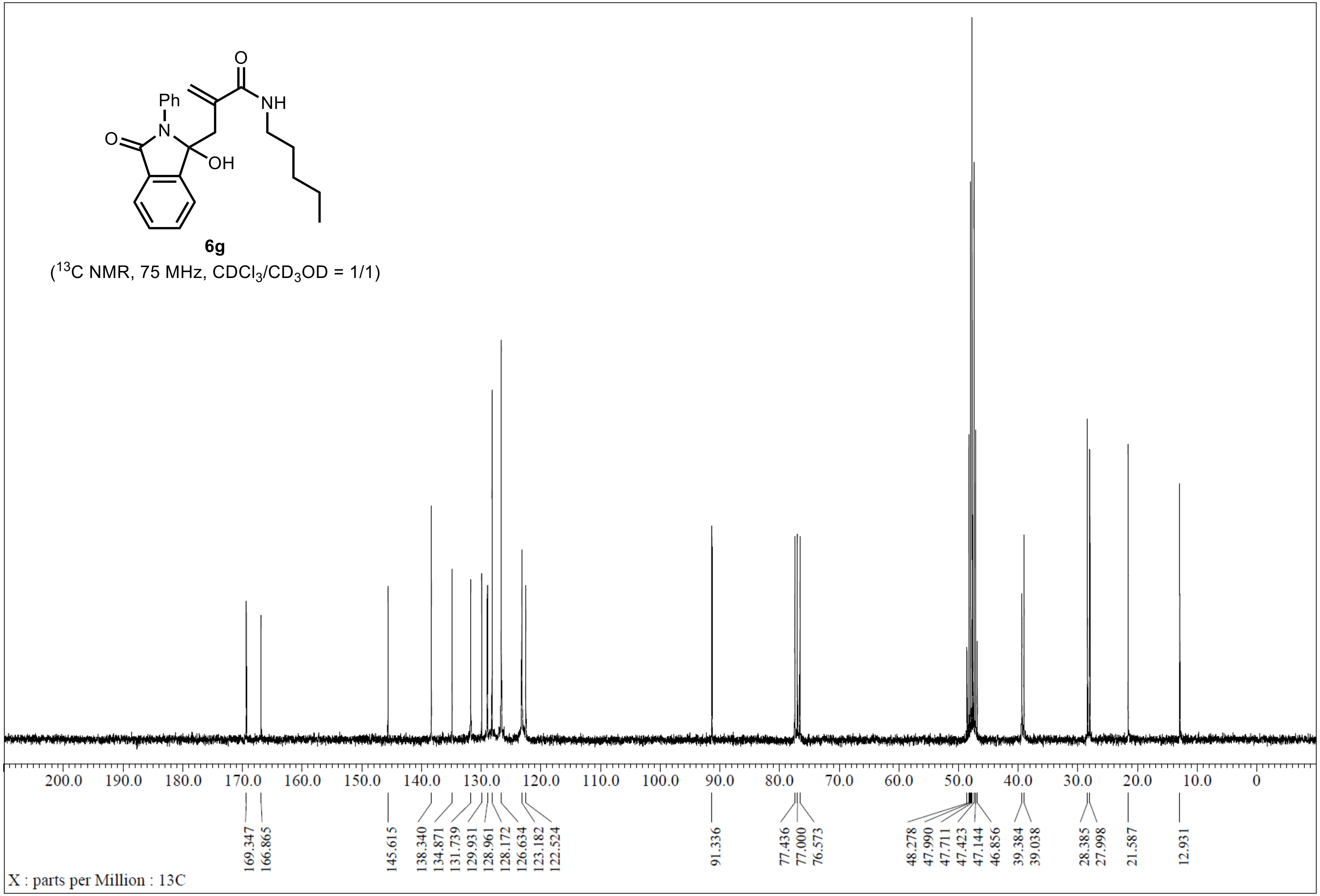
O
Ph
N
NH
O
OH
6g
(^{13}C NMR, 75 MHz, $CDCl_3/CD_3OD$ = 1/1)
200.0
190.0
180.0
170.0
160.0
150.0
140.0
130.0
120.0
110.0
100.0
90.0
80.0
70.0
60.0
50.0
40.0
30.0
20.0
10.0
0
169.347
166.865
145.615
138.340
134.871
131.739
129.931
128.961
128.172
126.634
123.182
122.524
91.336
77.436
77.000
76.573
48.278
47.990
47.711
47.423
47.144
46.856
39.384
39.038
28.385
27.998
21.587
12.931
X : parts per Million : 13C

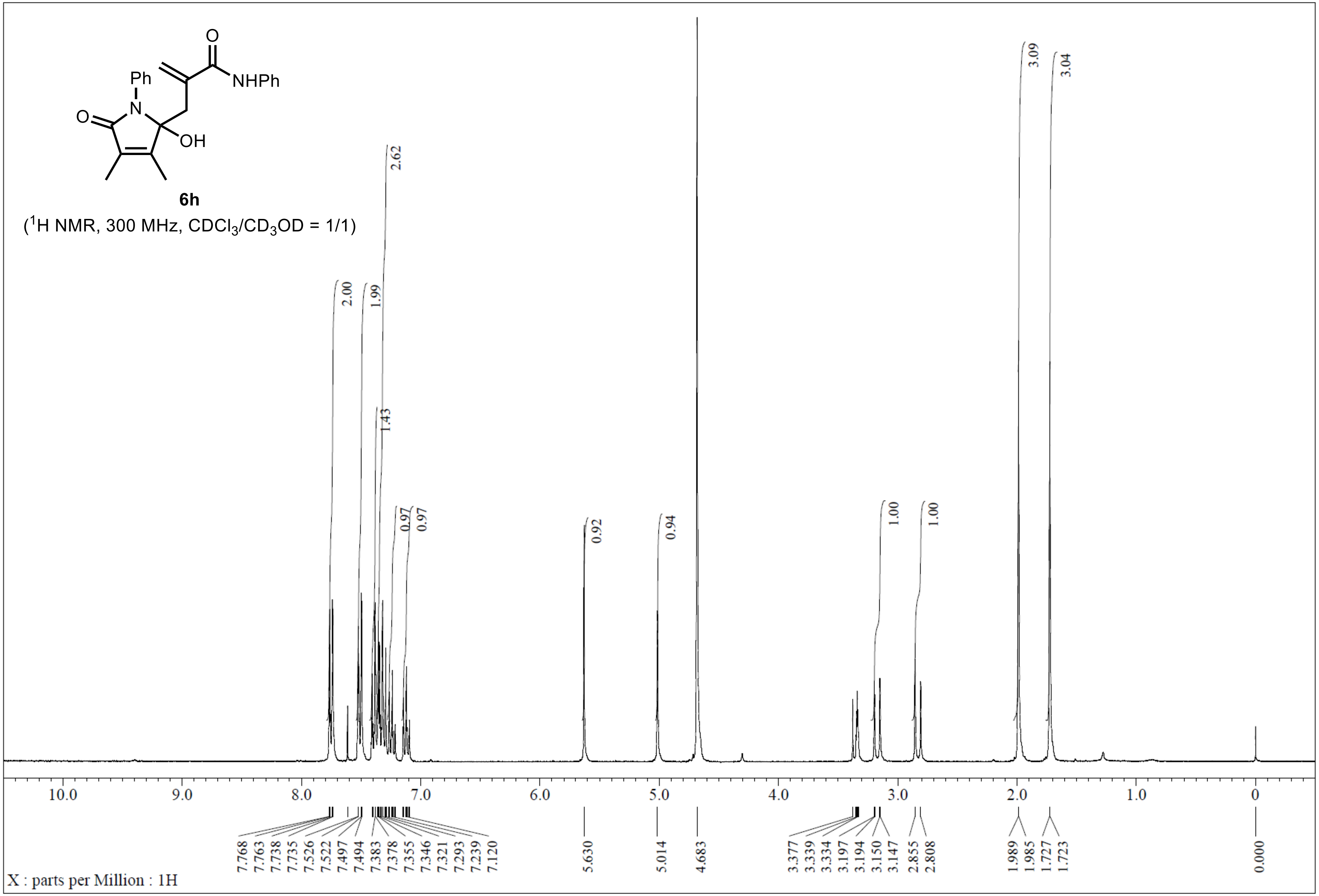

Ph
O
N
OH
NHPh
O
6h
(1H NMR, 300 MHz, CDCl3/CD3OD = 1/1)
2.00
1.99
2.62
1.43
0.97
0.97
0.92
0.94
1.00
1.00
3.09
3.04
10.0
9.0
8.0
7.0
6.0
5.0
4.0
3.0
2.0
1.0
0
7.768
7.763
7.738
7.735
7.526
7.522
7.497
7.494
7.383
7.378
7.355
7.346
7.321
7.293
7.239
7.120
5.630
5.014
4.683
3.377
3.339
3.334
3.197
3.194
3.150
3.147
2.855
2.808
1.989
1.985
1.727
1.723
0.000
X : parts per Million : 1H

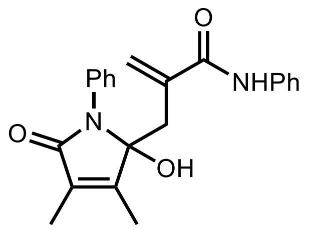

6h

(^{13}C NMR, 75 MHz, $CDCl_3/CD_3OD$ = 1/1)

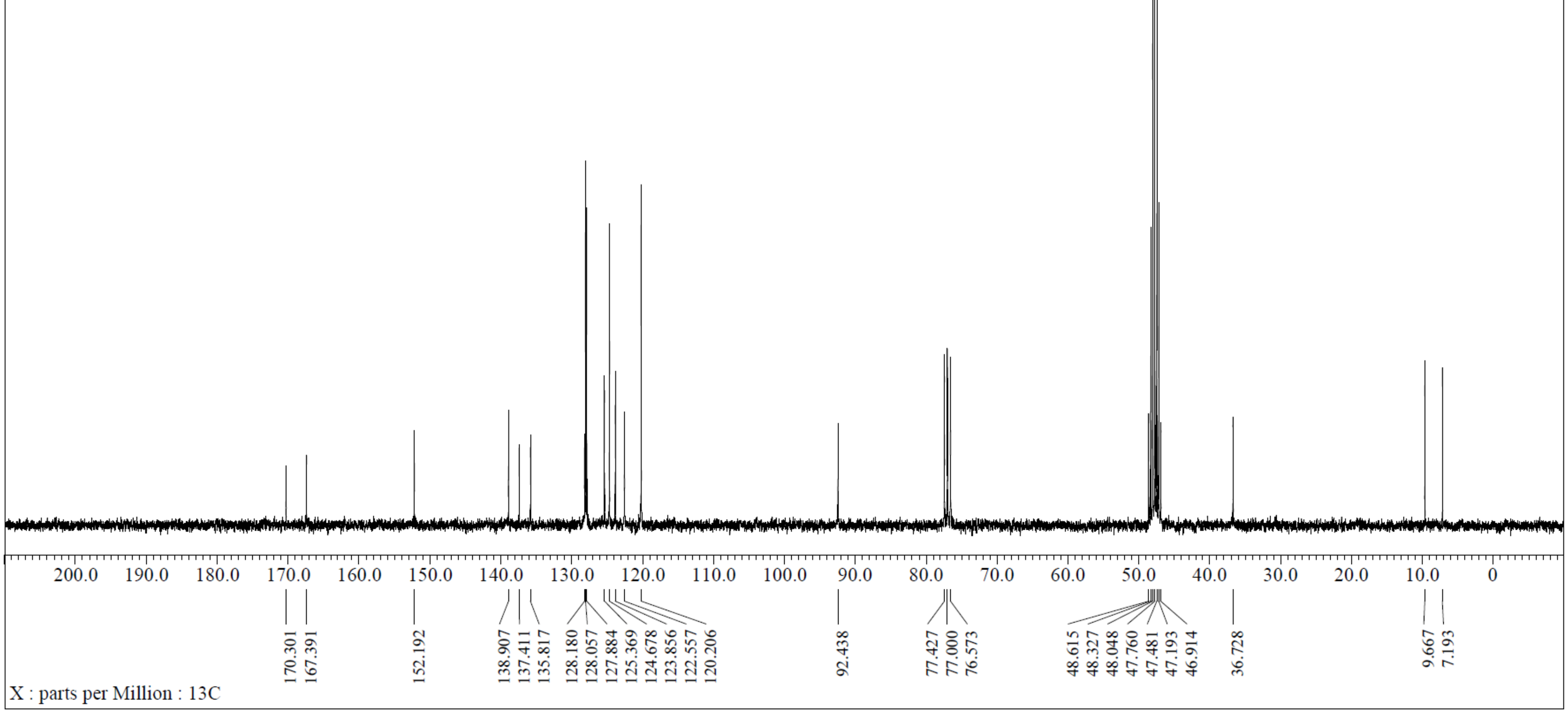

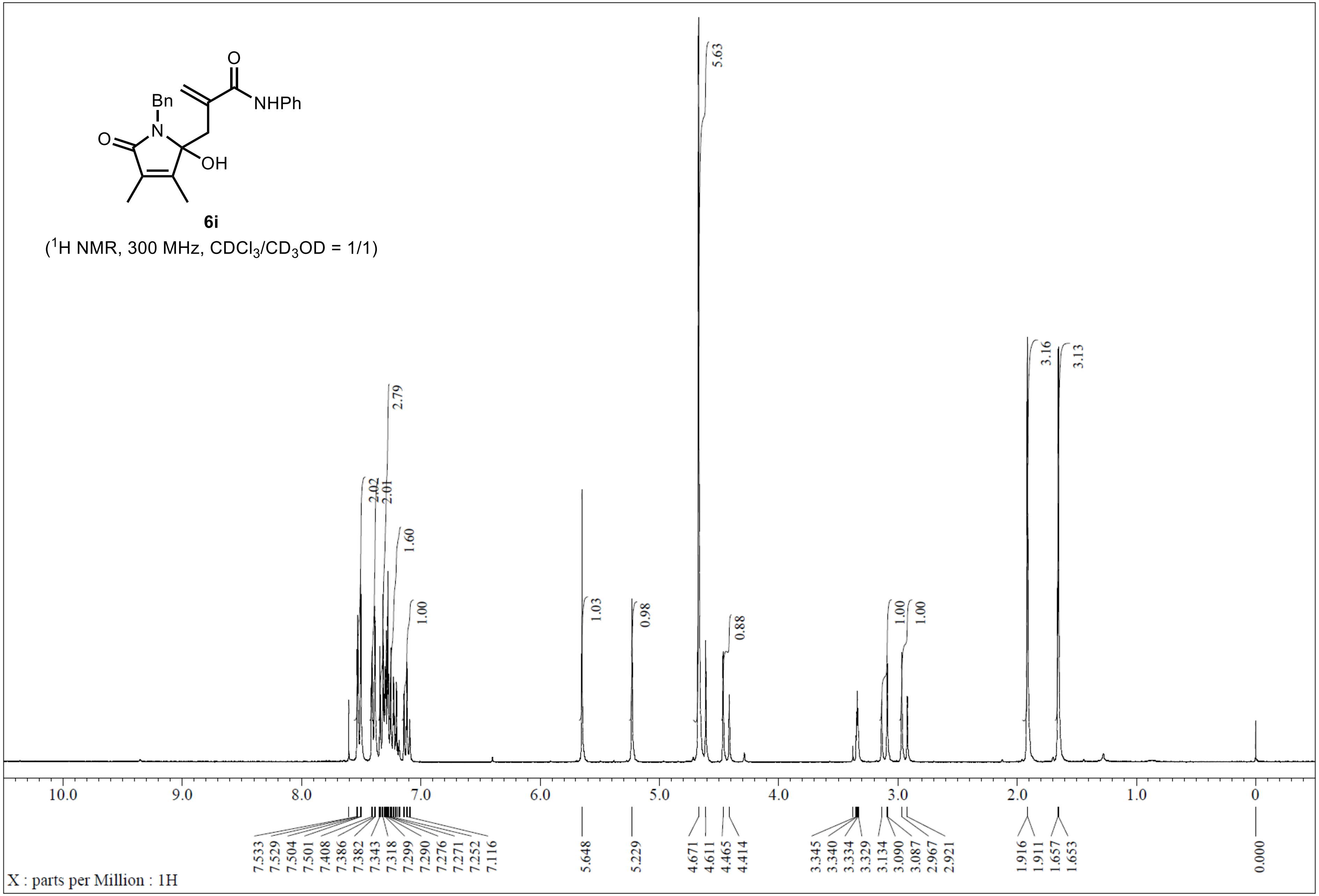
O
Bn
NHPh
O
N
OH
6i
(¹H NMR, 300 MHz, CDCl3/CD3OD = 1/1)
5.63
3.16
3.13
2.79
2.02
2.01
1.60
1.00
1.03
0.98
0.88
1.00
1.00
10.0
9.0
8.0
7.0
6.0
5.0
4.0
3.0
2.0
1.0
0
7.533 7.529 7.504 7.501 7.408 7.386 7.382 7.343 7.318 7.299 7.290 7.276 7.271 7.252 7.116
5.648
5.229
4.671 4.611 4.465 4.414
3.345 3.340 3.334 3.329 3.134 3.090 3.087 2.967 2.921
1.916 1.911 1.657 1.653
0.000
X : parts per Million : 1H

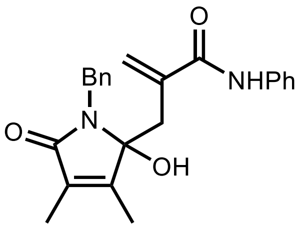

6i

(^{13}C NMR, 75 MHz, $CDCl_3/CD_3OD$ = 1/1)

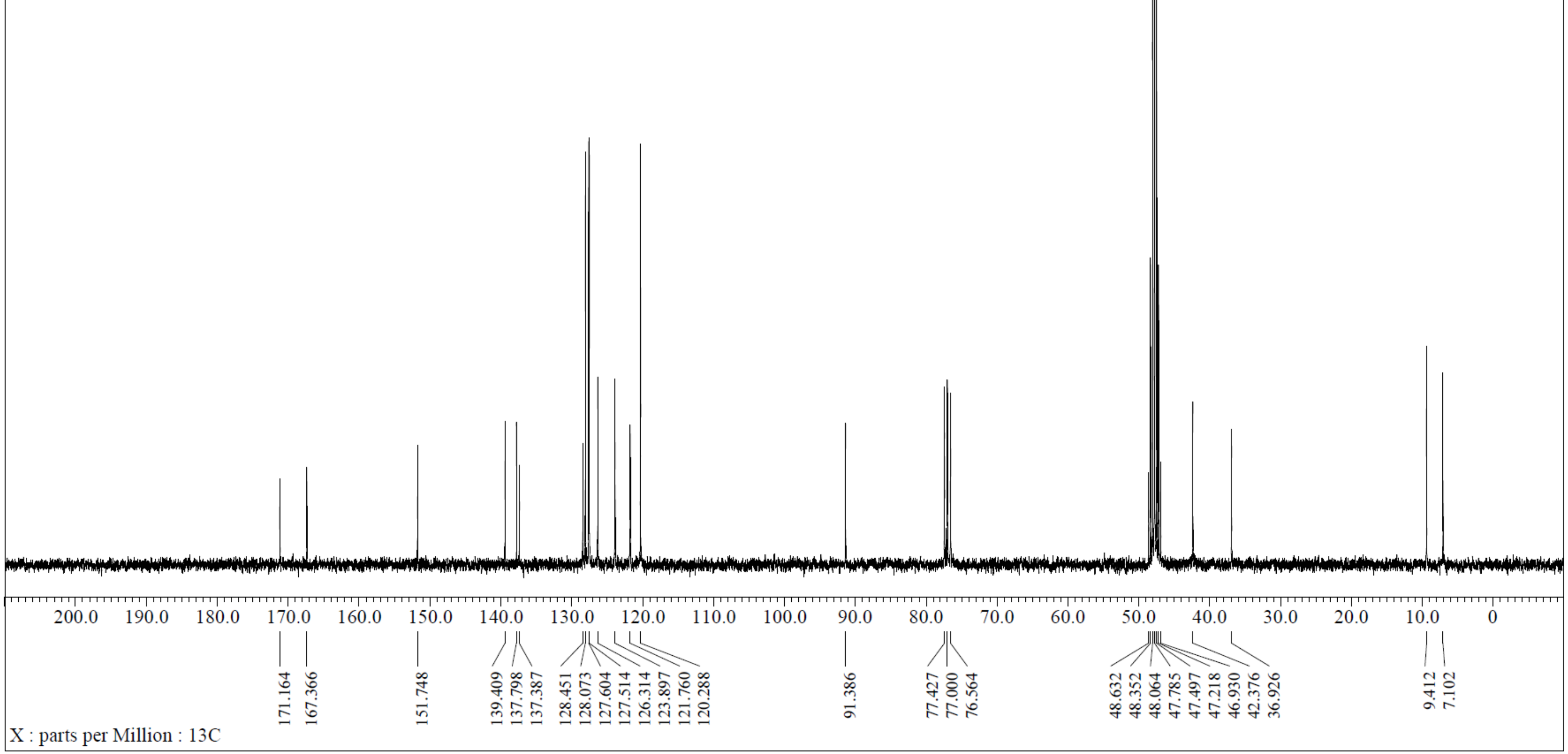

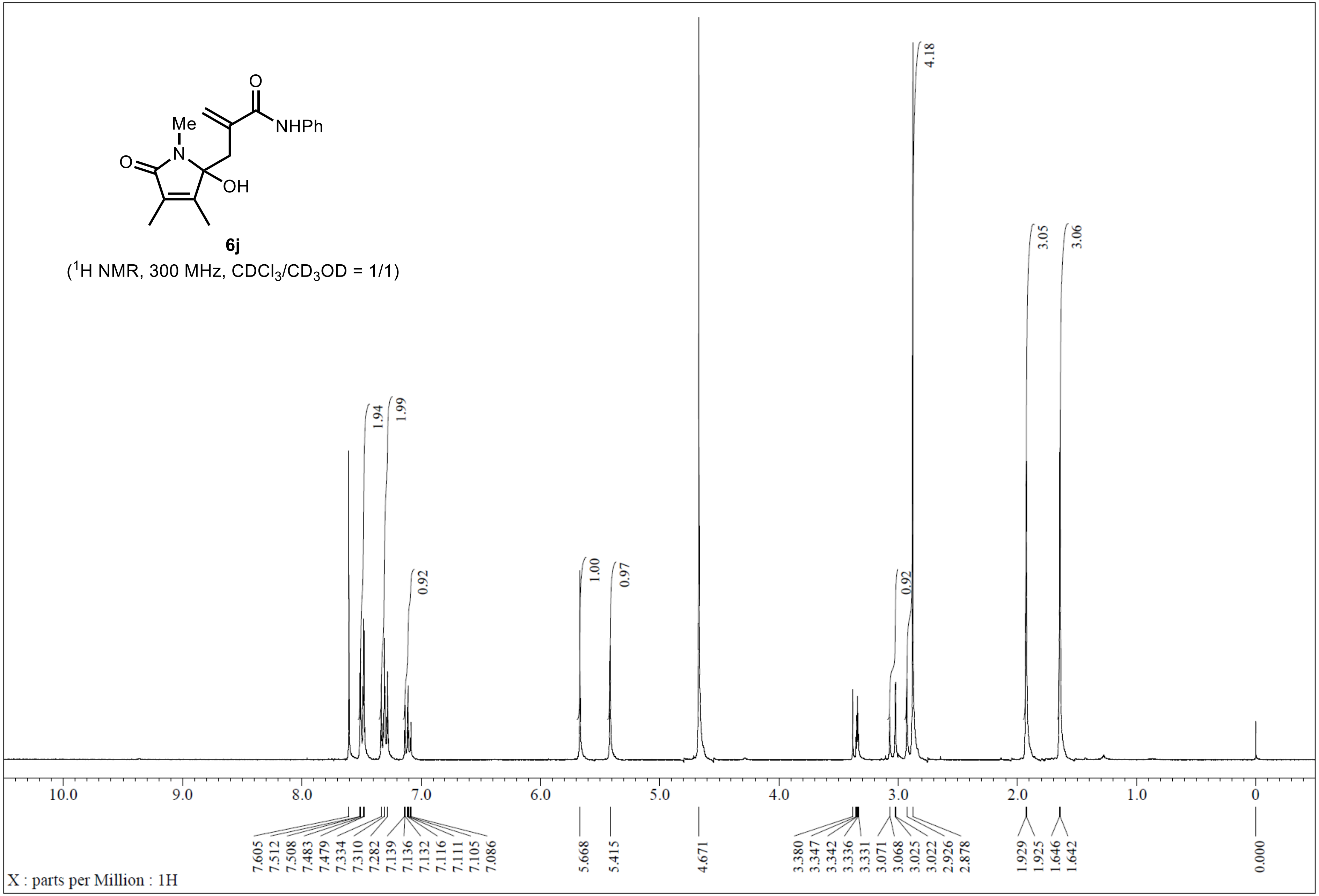

Me
N
O
OH
O
NHPh
6j
(1H NMR, 300 MHz, CDCl3/CD3OD = 1/1)
X : parts per Million : 1H

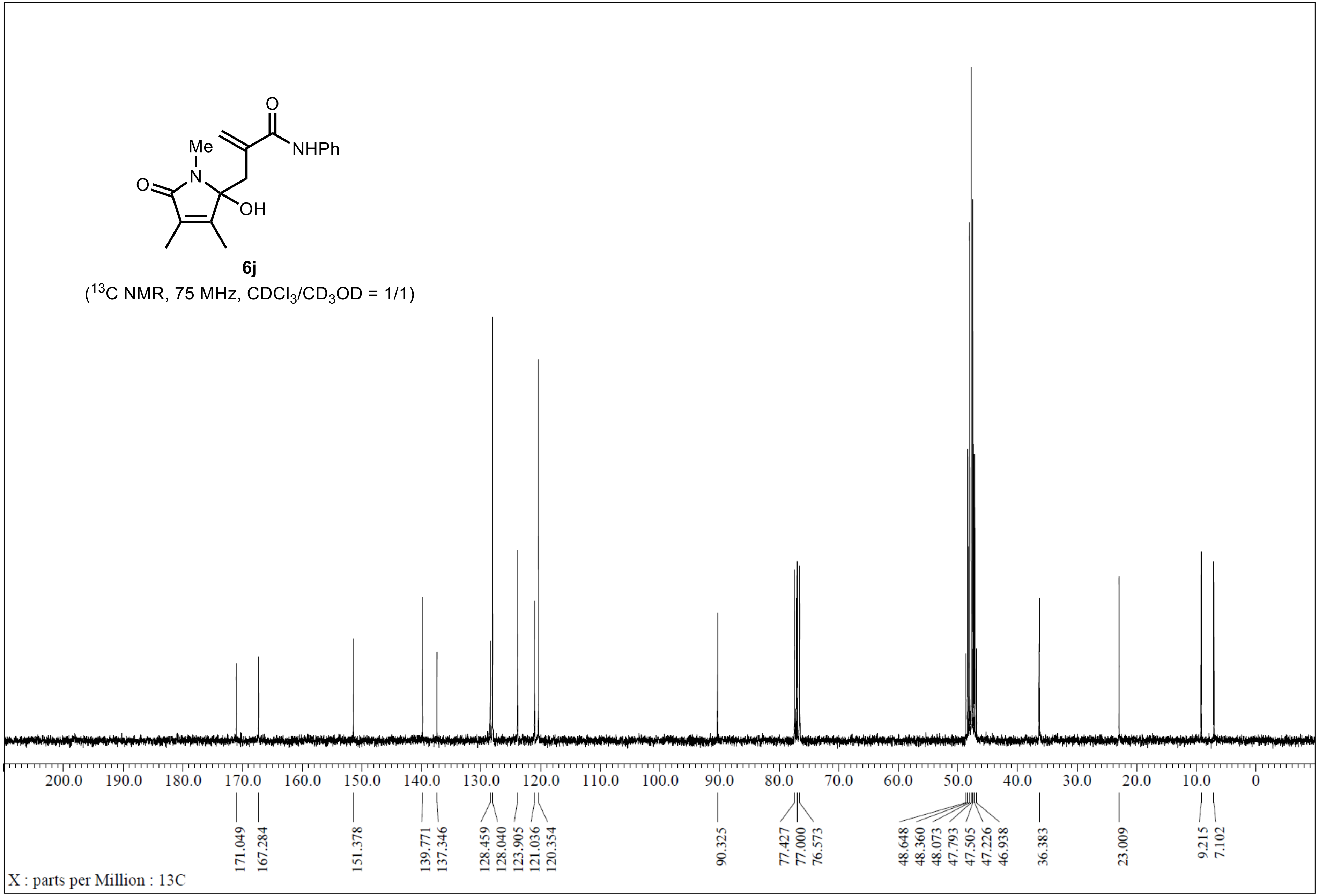
O
Me
N
NHPh
O
OH
6j
(13C NMR, 75 MHz, CDCl3/CD3OD = 1/1)
200.0
190.0
180.0
170.0
160.0
150.0
140.0
130.0
120.0
110.0
100.0
90.0
80.0
70.0
60.0
50.0
40.0
30.0
20.0
10.0
0
171.049
167.284
151.378
139.771
137.346
128.459
128.040
123.905
121.036
120.354
90.325
77.427
77.000
76.573
48.648
48.360
48.073
47.793
47.505
47.226
46.938
36.383
23.009
9.215
7.102
X : parts per Million : 13C

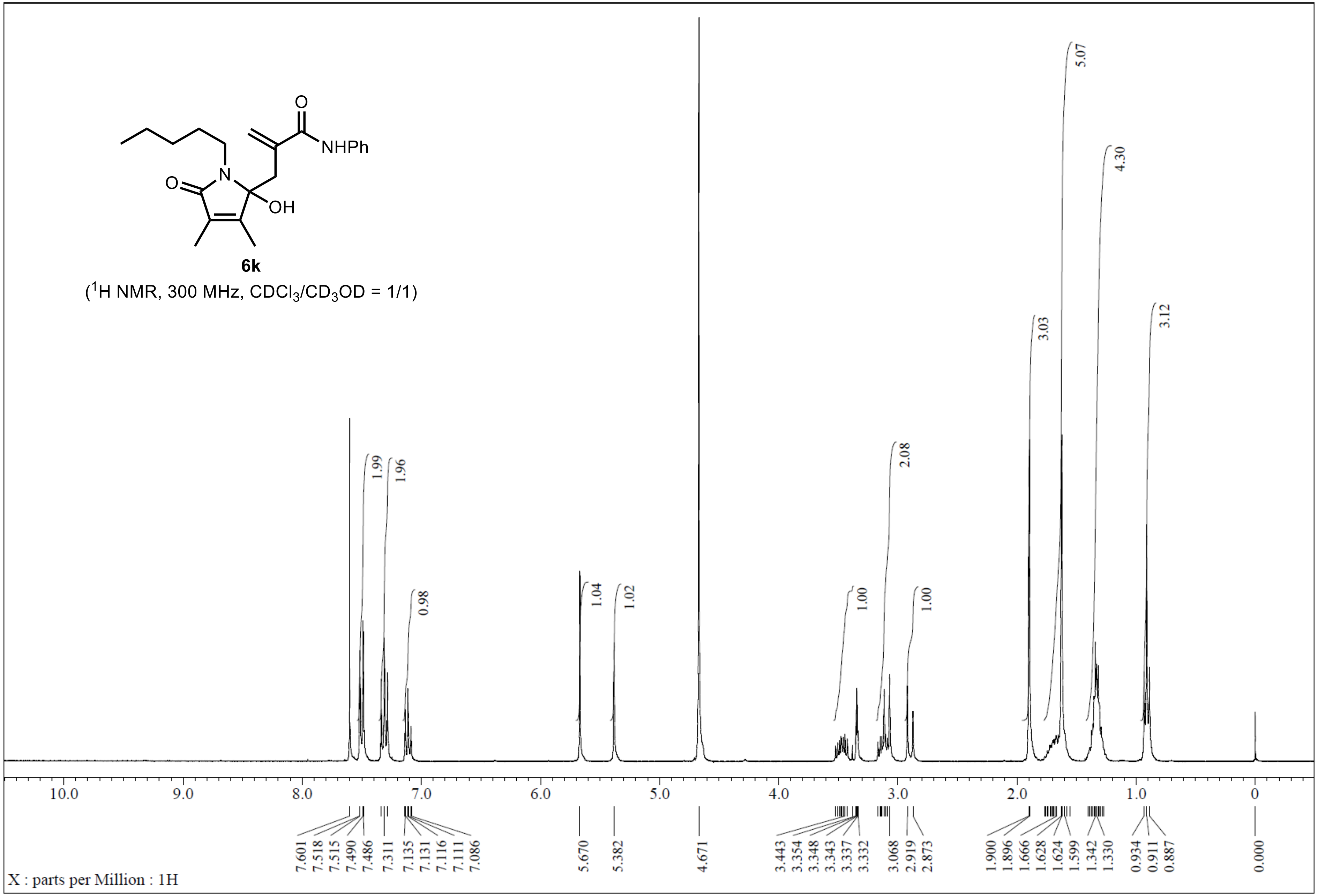
O
NHPh
O
N
OH
6k
(1H NMR, 300 MHz, CDCl3/CD3OD = 1/1)
1.99
1.96
0.98
1.04
1.02
1.00
2.08
1.00
3.03
5.07
4.30
3.12
10.0
9.0
8.0
7.0
6.0
5.0
4.0
3.0
2.0
1.0
0
7.601
7.518
7.515
7.490
7.486
7.311
7.135
7.131
7.116
7.111
7.086
5.670
5.382
4.671
3.443
3.354
3.348
3.343
3.337
3.332
3.068
2.919
2.873
1.900
1.896
1.666
1.628
1.624
1.599
1.342
1.330
0.934
0.911
0.887
0.000
X : parts per Million : 1H

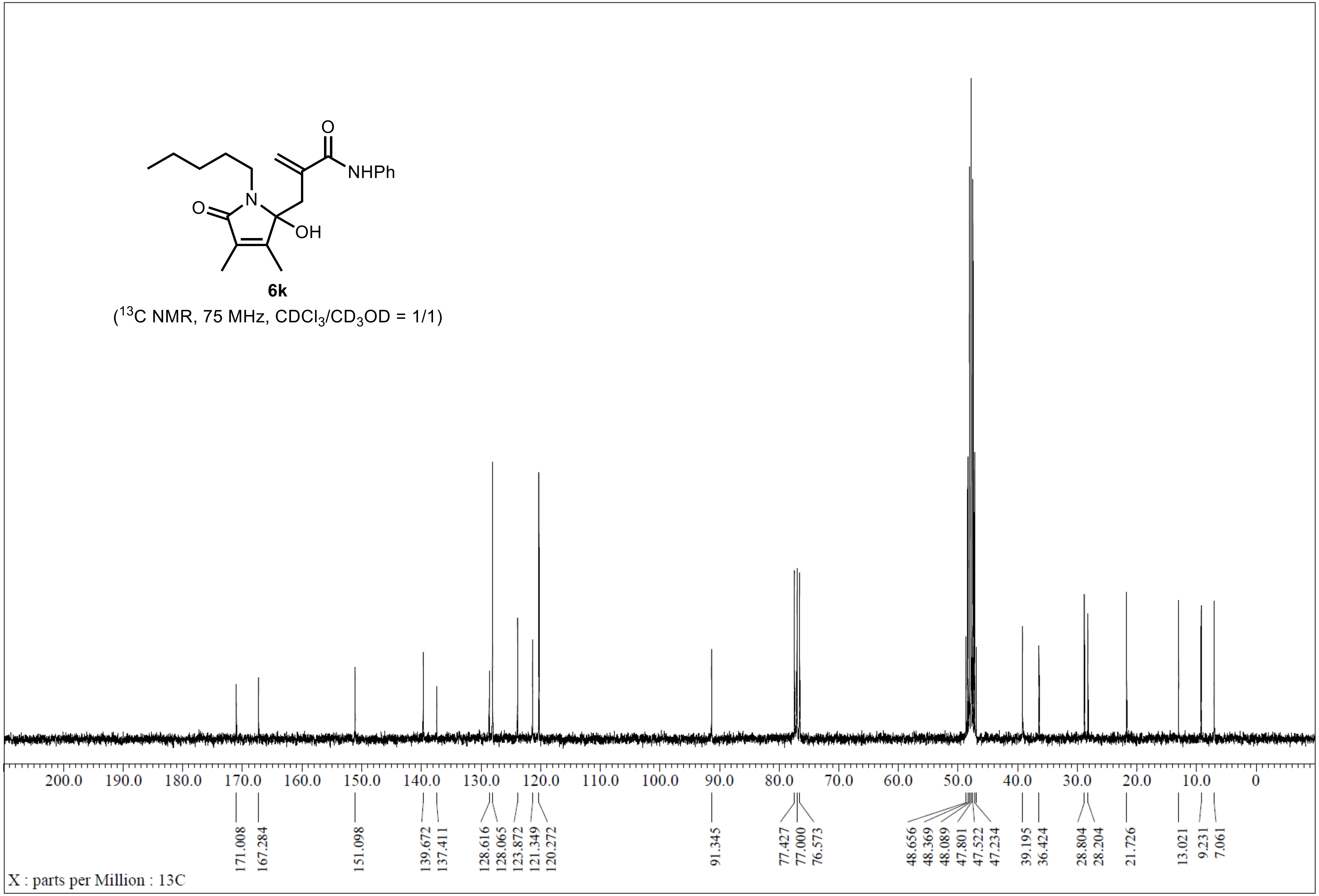

6k

(^{13}C NMR, 75 MHz, $CDCl_3/CD_3OD$ = 1/1)

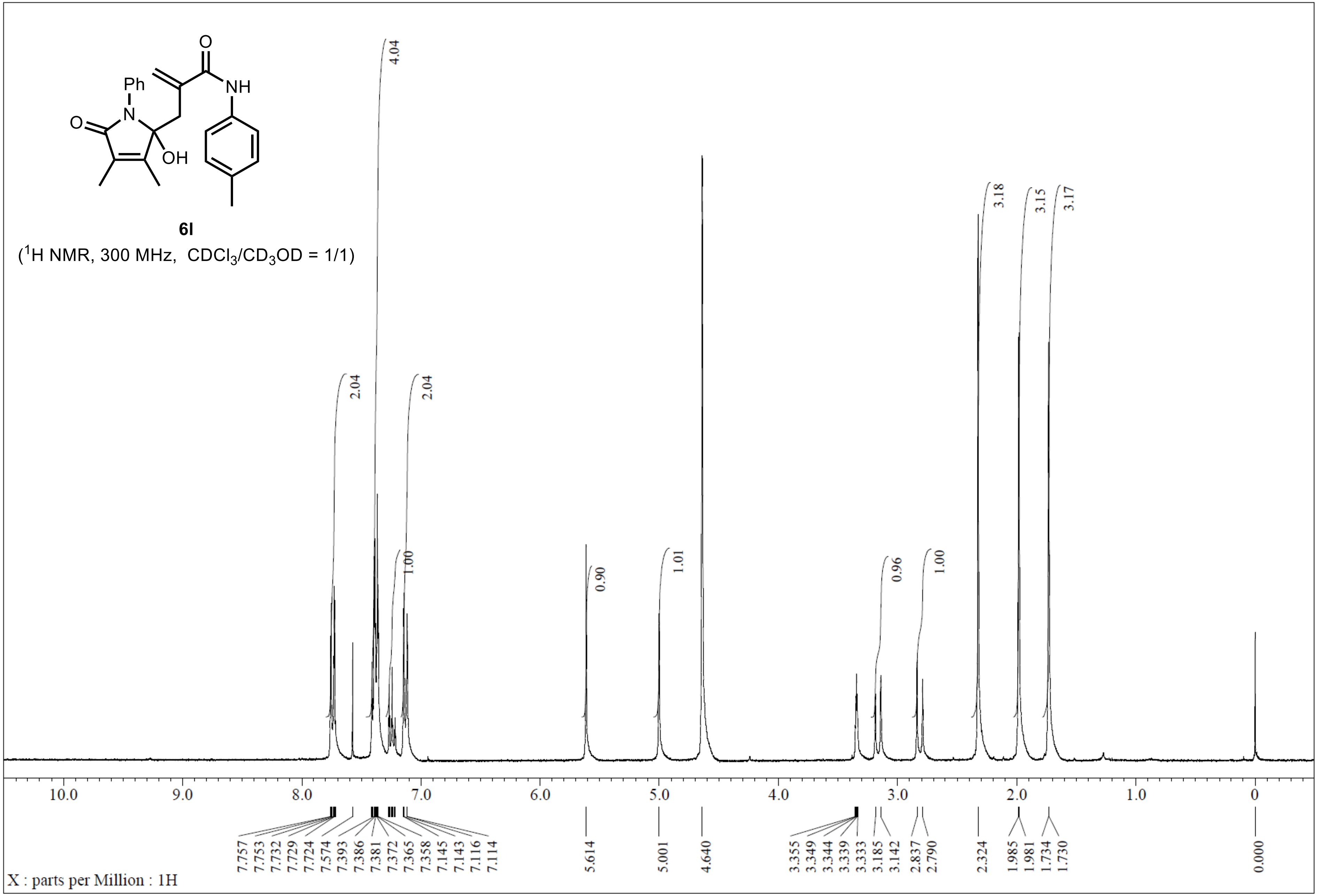

Ph
N
O
OH
NH
6l
(^{1}H NMR, 300 MHz, CDCl$_3$/CD$_3$OD = 1/1)
2.04
4.04
1.00
2.04
0.90
1.01
0.96
1.00
3.18
3.15
3.17
10.0
9.0
8.0
7.0
6.0
5.0
4.0
3.0
2.0
1.0
0
7.757 7.753 7.732 7.729 7.724 7.574 7.393 7.386 7.381 7.372 7.365 7.358 7.145 7.143 7.116 7.114
5.614
5.001
4.640
3.355 3.349 3.344 3.339 3.333 3.185 3.142 2.837 2.790
2.324
1.985 1.981 1.734 1.730
0.000
X : parts per Million : 1H

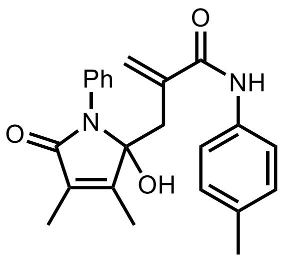

6l

(^{13}C NMR, 75 MHz, $CDCl_3/CD_3OD$ = 1/1)

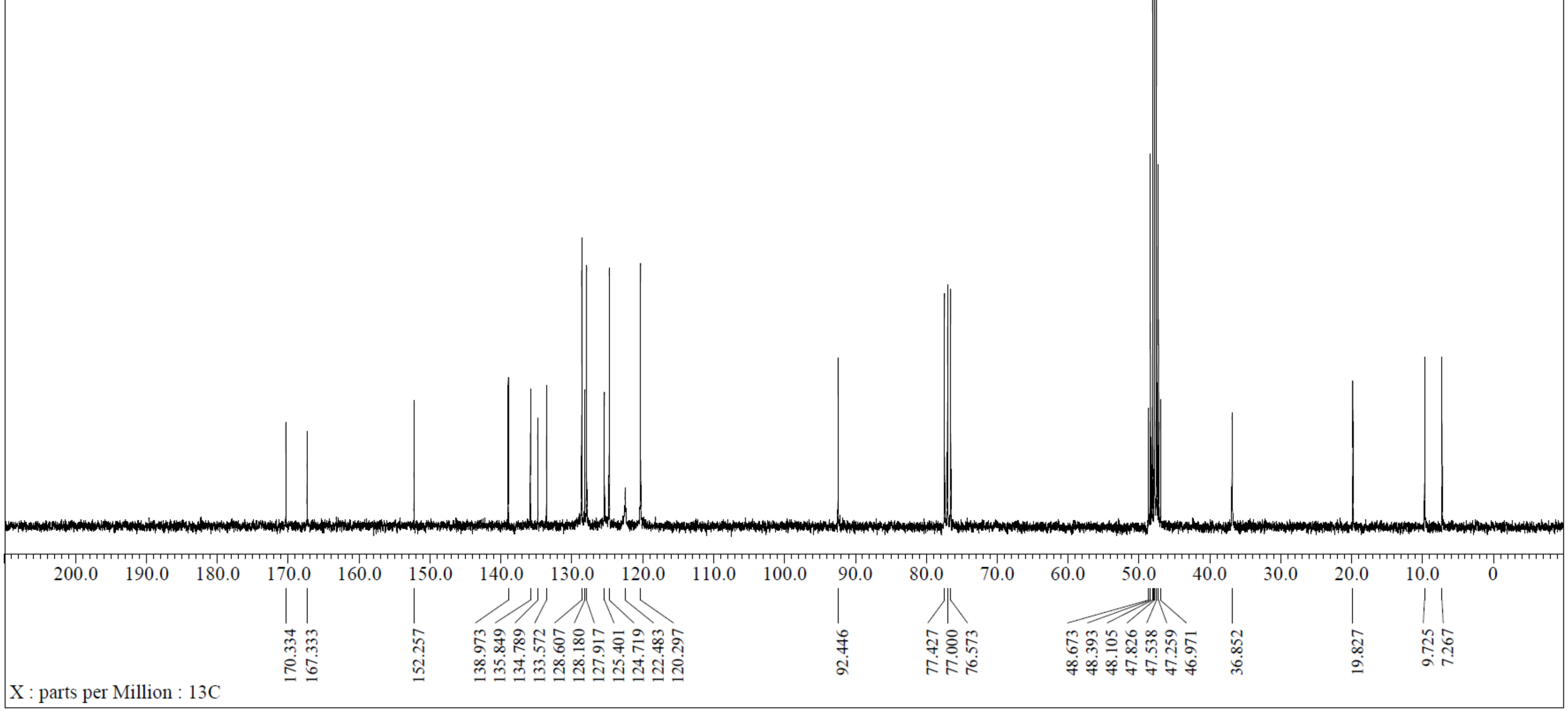

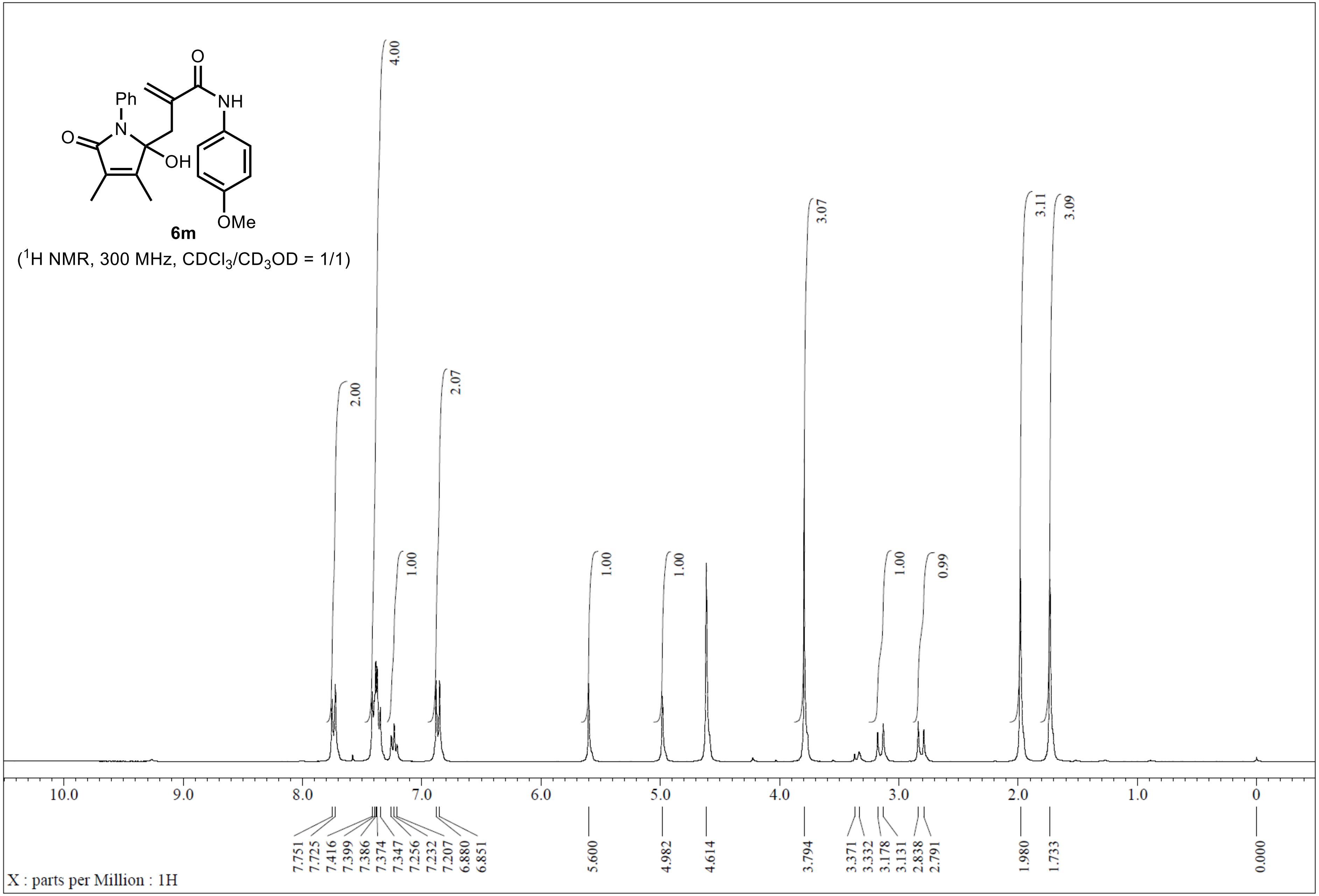
Ph
N
O
OH
O
NH
OMe
6m
(1H NMR, 300 MHz, CDCl3/CD3OD = 1/1)
2.00
4.00
1.00
2.07
1.00
1.00
3.07
1.00
0.99
3.11
3.09
10.0
9.0
8.0
7.0
6.0
5.0
4.0
3.0
2.0
1.0
0
7.751
7.725
7.416
7.399
7.386
7.374
7.347
7.256
7.232
7.207
6.880
6.851
5.600
4.982
4.614
3.794
3.371
3.332
3.178
3.131
2.838
2.791
1.980
1.733
0.000
X : parts per Million : 1H

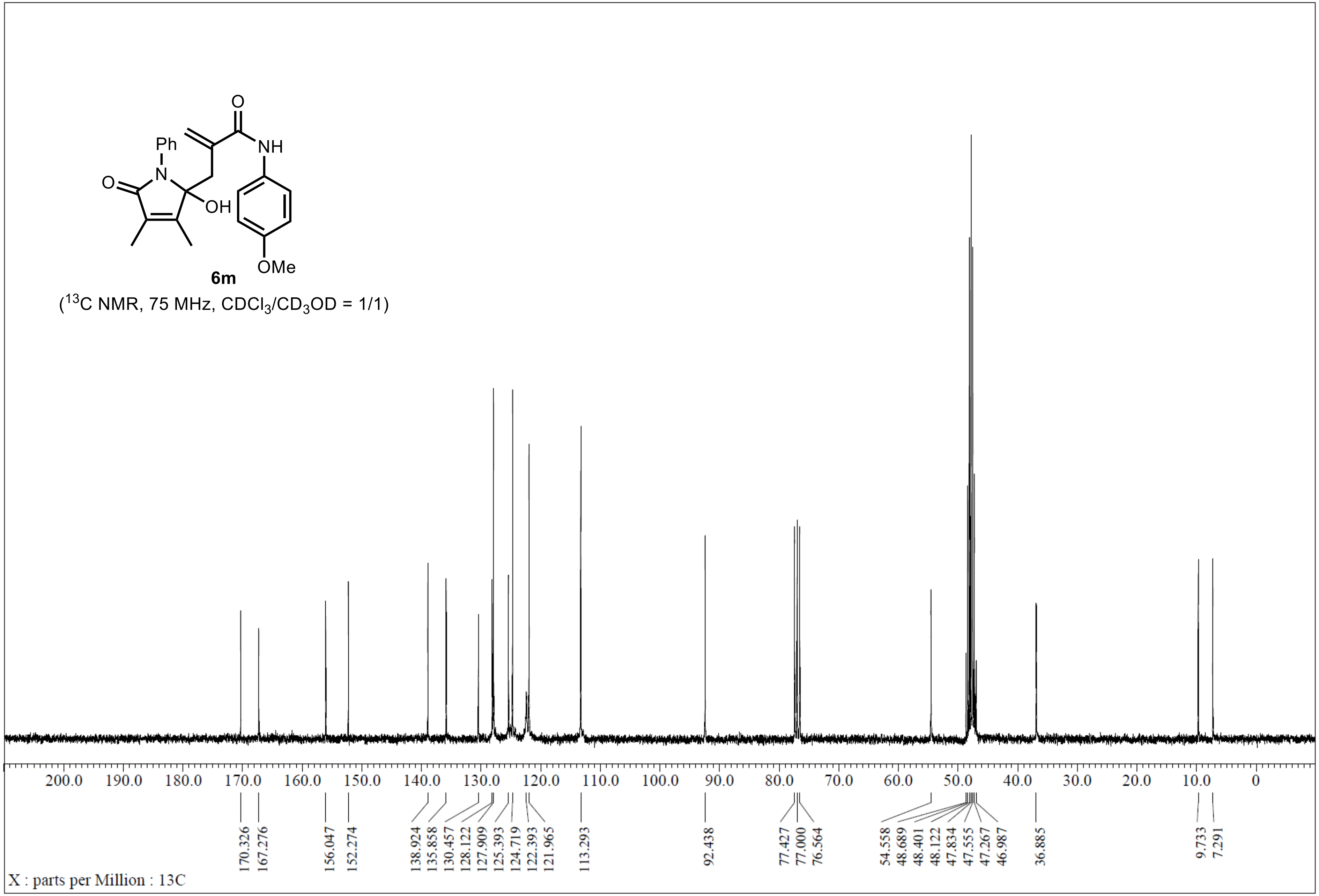

Ph
N
O
OH
O
NH
OMe
6m
(13C NMR, 75 MHz, CDCl3/CD3OD = 1/1)
200.0
190.0
180.0
170.0
160.0
150.0
140.0
130.0
120.0
110.0
100.0
90.0
80.0
70.0
60.0
50.0
40.0
30.0
20.0
10.0
0
170.326
167.276
156.047
152.274
138.924
135.858
130.457
128.122
127.909
125.393
124.719
122.393
121.965
113.293
92.438
77.427
77.000
76.564
54.558
48.689
48.401
48.122
47.834
47.555
47.267
46.987
36.885
9.733
7.291
X : parts per Million : 13C

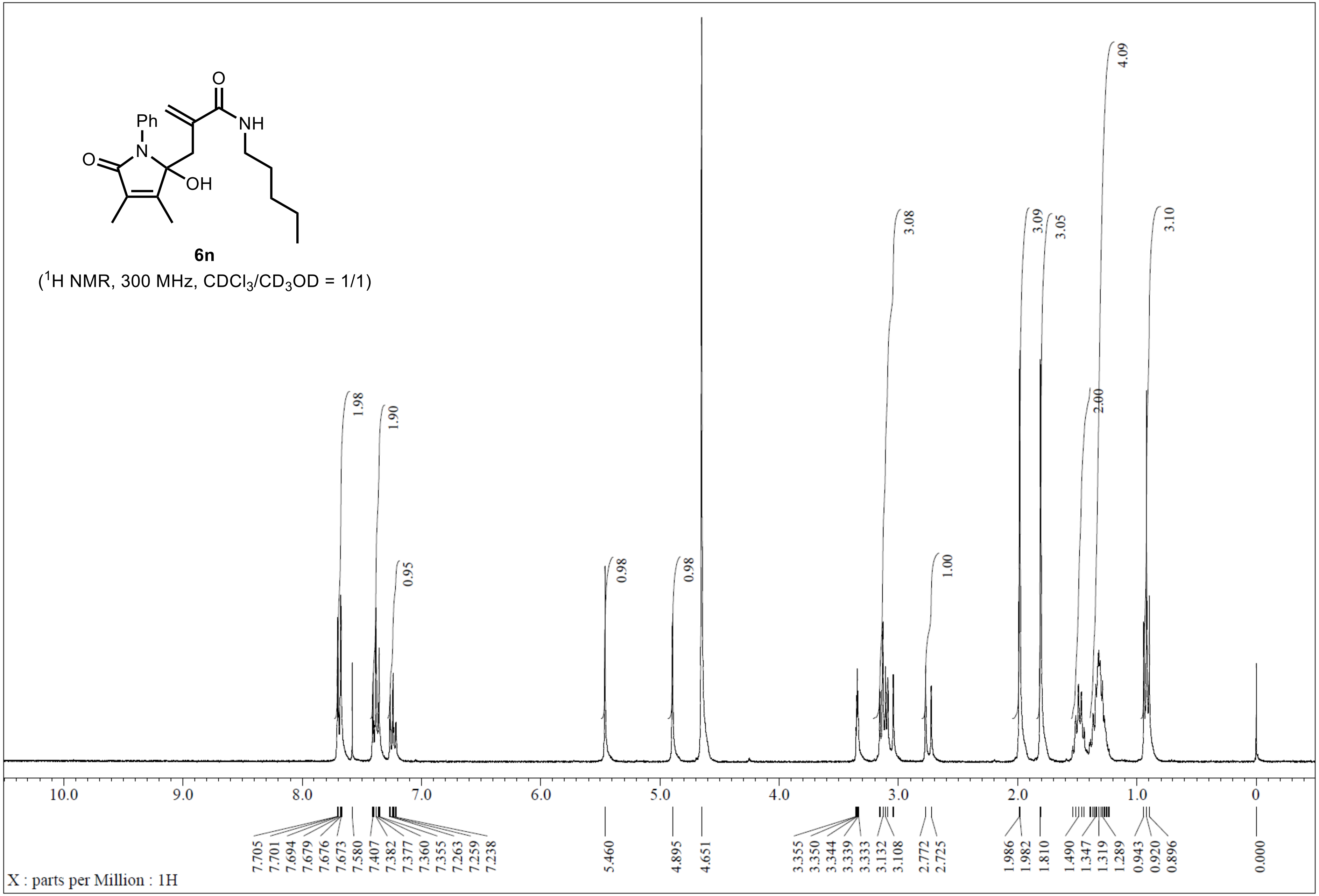
6n
(1H NMR, 300 MHz, CDCl3/CD3OD = 1/1)
X : parts per Million : 1H
10.0
9.0
8.0
7.0
6.0
5.0
4.0
3.0
2.0
1.0
0
1.98
1.90
0.95
0.98
0.98
3.08
1.00
3.09
3.05
2.00
4.09
3.10
7.705
7.701
7.694
7.679
7.676
7.673
7.580
7.407
7.382
7.377
7.360
7.355
7.263
7.259
7.238
5.460
4.895
4.651
3.355
3.350
3.344
3.339
3.333
3.132
3.108
2.772
2.725
1.986
1.982
1.810
1.490
1.347
1.319
1.289
0.943
0.920
0.896
0.000

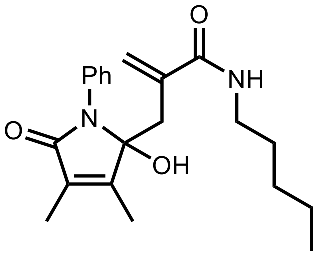

6n

(^{13}C NMR, 75 MHz, $CDCl_3/CD_3OD$ = 1/1)

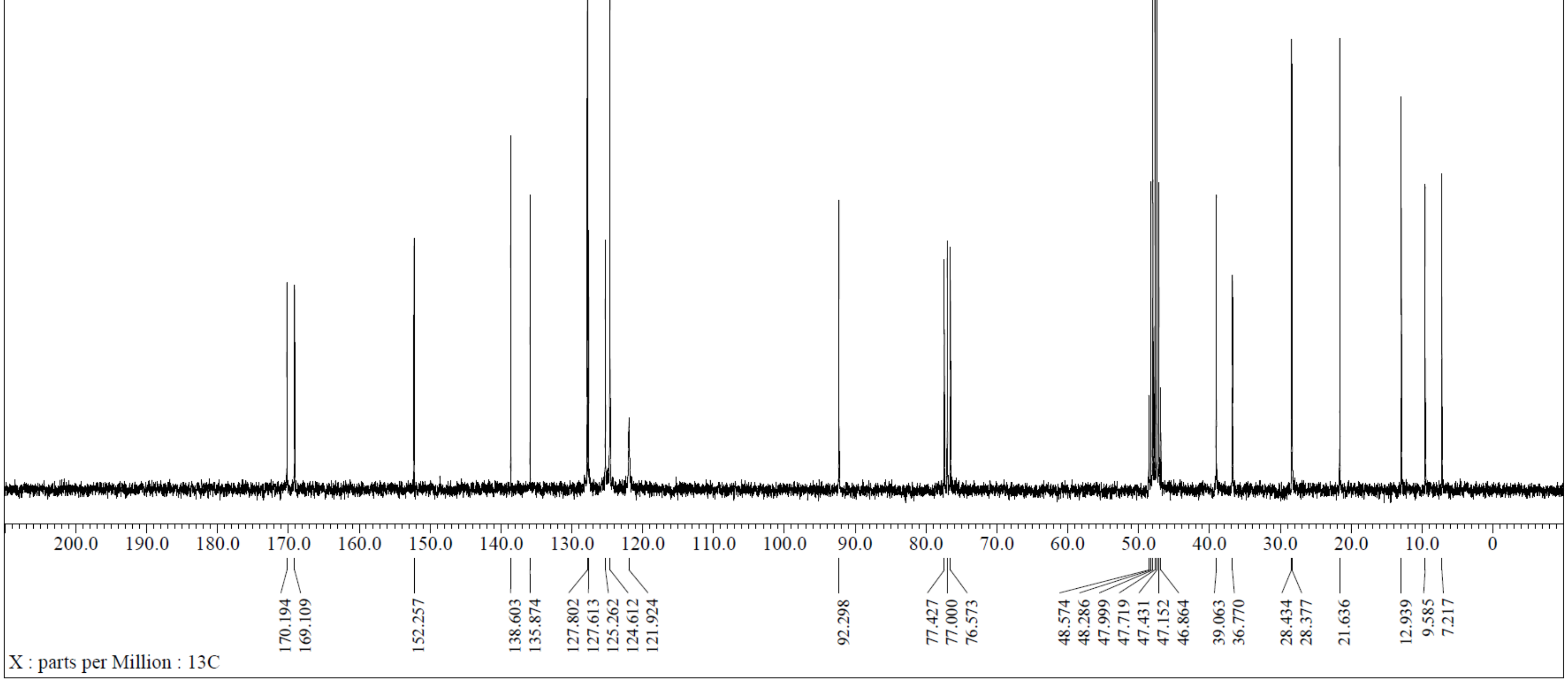

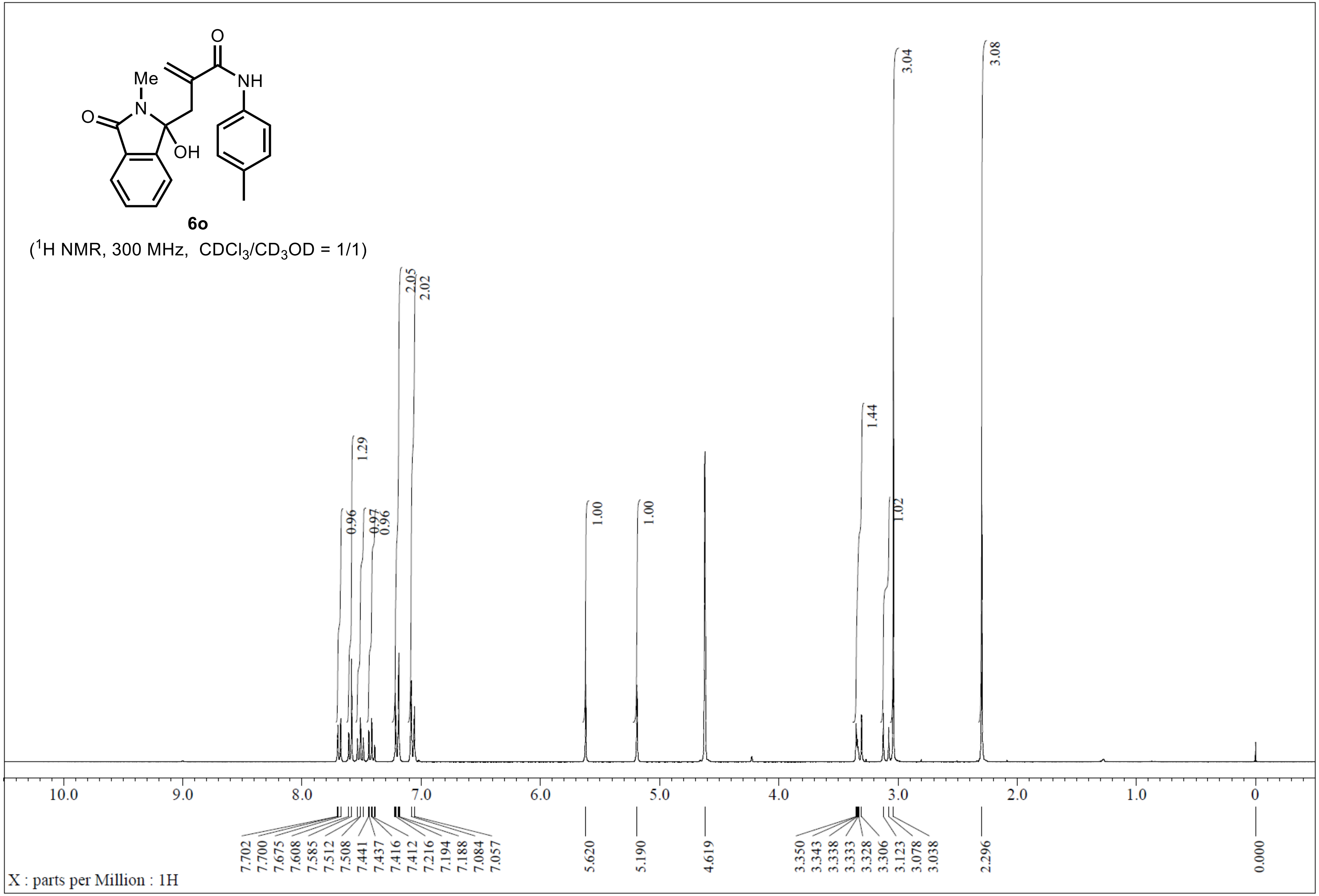
O
Me
NH
O
N
OH
6o
(1H NMR, 300 MHz, CDCl3/CD3OD = 1/1)
3.04
3.08
2.05
2.02
1.29
0.96
0.97
0.96
1.00
1.00
1.44
1.02
10.0
9.0
8.0
7.0
6.0
5.0
4.0
3.0
2.0
1.0
0
7.702
7.700
7.675
7.608
7.585
7.512
7.508
7.441
7.437
7.416
7.412
7.216
7.194
7.188
7.084
7.057
5.620
5.190
4.619
3.350
3.343
3.338
3.333
3.328
3.306
3.123
3.078
3.038
2.296
0.000
X : parts per Million : 1H

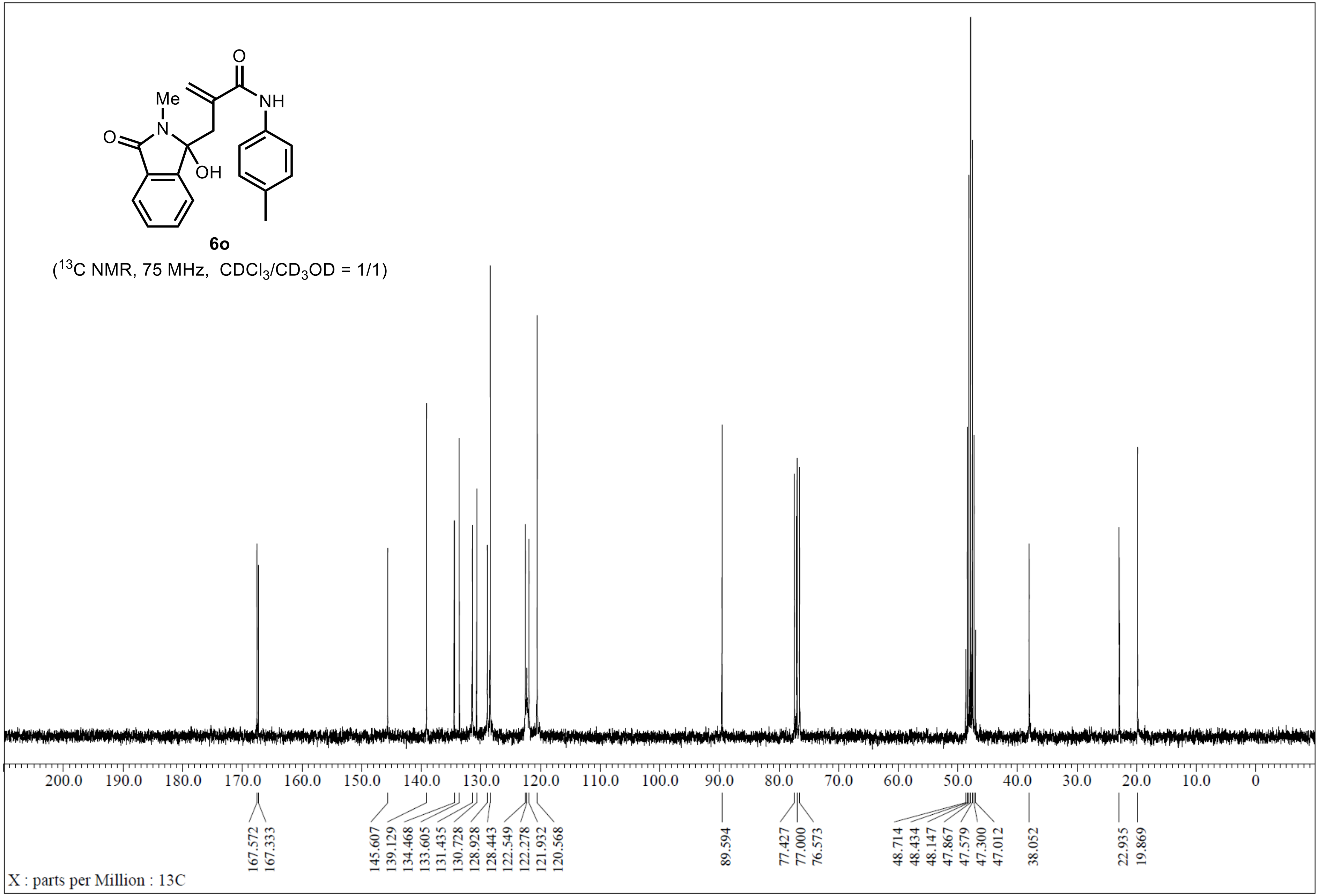

O
Me
N
NH
O
OH
6o
(13C NMR, 75 MHz, CDCl3/CD3OD = 1/1)
200.0
190.0
180.0
170.0
160.0
150.0
140.0
130.0
120.0
110.0
100.0
90.0
80.0
70.0
60.0
50.0
40.0
30.0
20.0
10.0
0
167.572
167.333
145.607
139.129
134.468
133.605
131.435
130.728
128.928
128.443
122.549
122.278
121.932
120.568
89.594
77.427
77.000
76.573
48.714
48.434
48.147
47.867
47.579
47.300
47.012
38.052
22.935
19.869
X : parts per Million : 13C

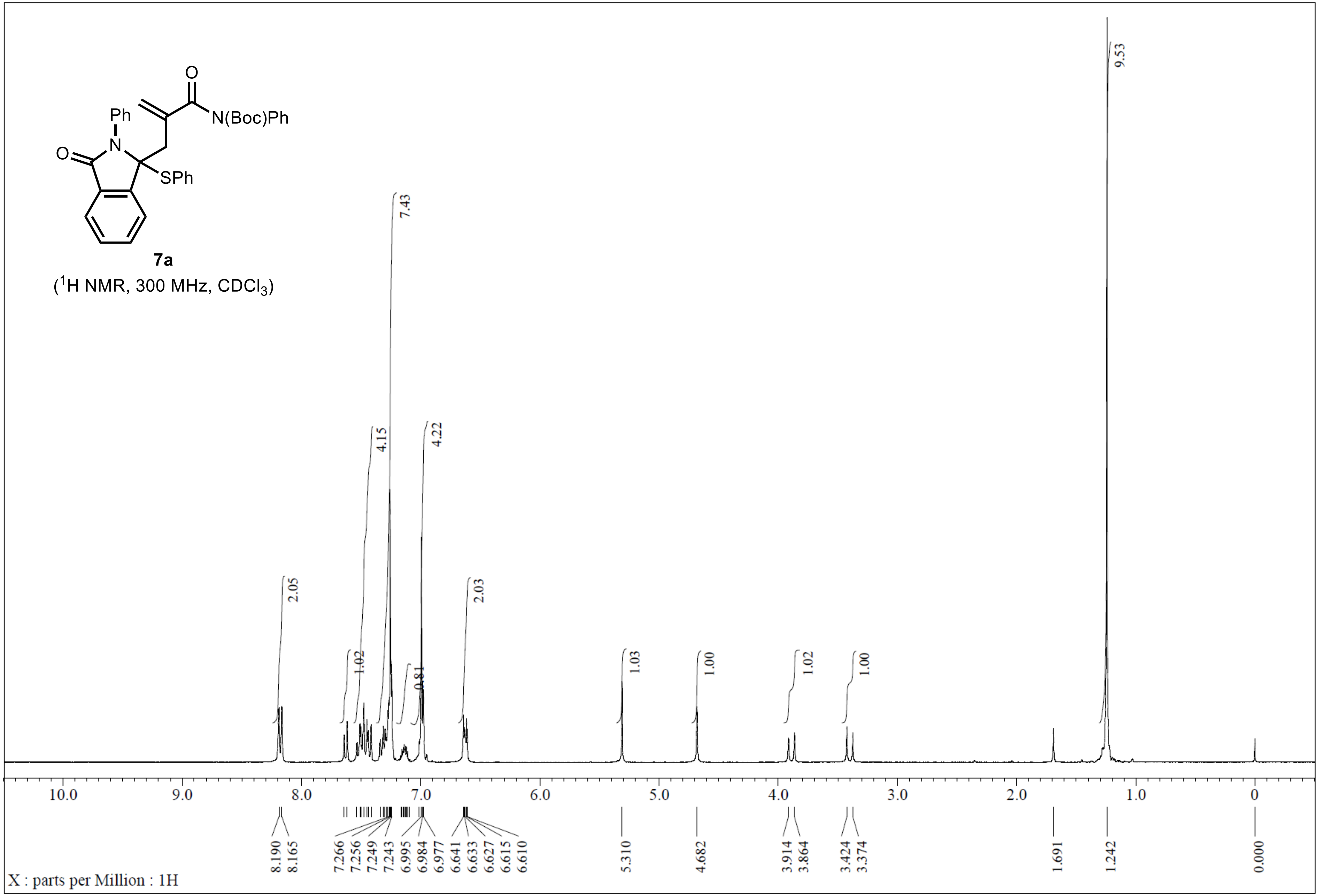
O
Ph
N
N(Boc)Ph
O
SPh
7a
(^{1}H NMR, 300 MHz, $CDCl_3$)
9.53
7.43
4.15
4.22
2.05
1.02
0.81
2.03
1.03
1.00
1.02
1.00
10.0
9.0
8.0
7.0
6.0
5.0
4.0
3.0
2.0
1.0
0
8.190
8.165
7.266
7.256
7.249
7.243
6.995
6.984
6.977
6.641
6.633
6.627
6.615
6.610
5.310
4.682
3.914
3.864
3.424
3.374
1.691
1.242
0.000
X : parts per Million : 1H

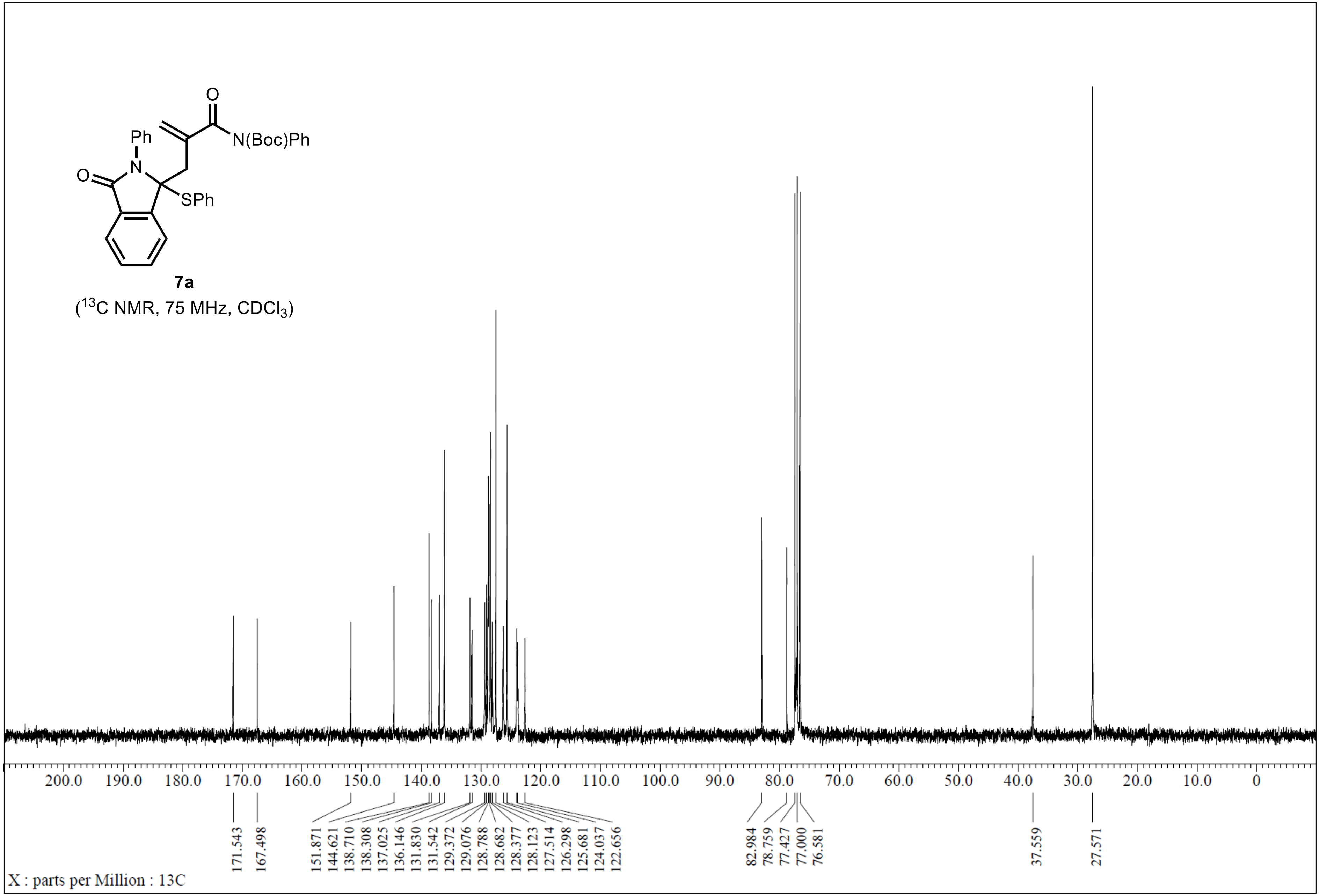
O
Ph
N(Boc)Ph
O
N
SPh
7a
(13C NMR, 75 MHz, CDCl3)
200.0 190.0 180.0 170.0 160.0 150.0 140.0 130.0 120.0 110.0 100.0 90.0 80.0 70.0 60.0 50.0 40.0 30.0 20.0 10.0 0
171.543
167.498
151.871
144.621
138.710
138.308
137.025
136.146
131.830
131.542
129.372
129.076
128.788
128.682
128.377
128.123
127.514
126.298
125.681
124.037
122.656
82.984
78.759
77.427
77.000
76.581
37.559
27.571
X : parts per Million : 13C

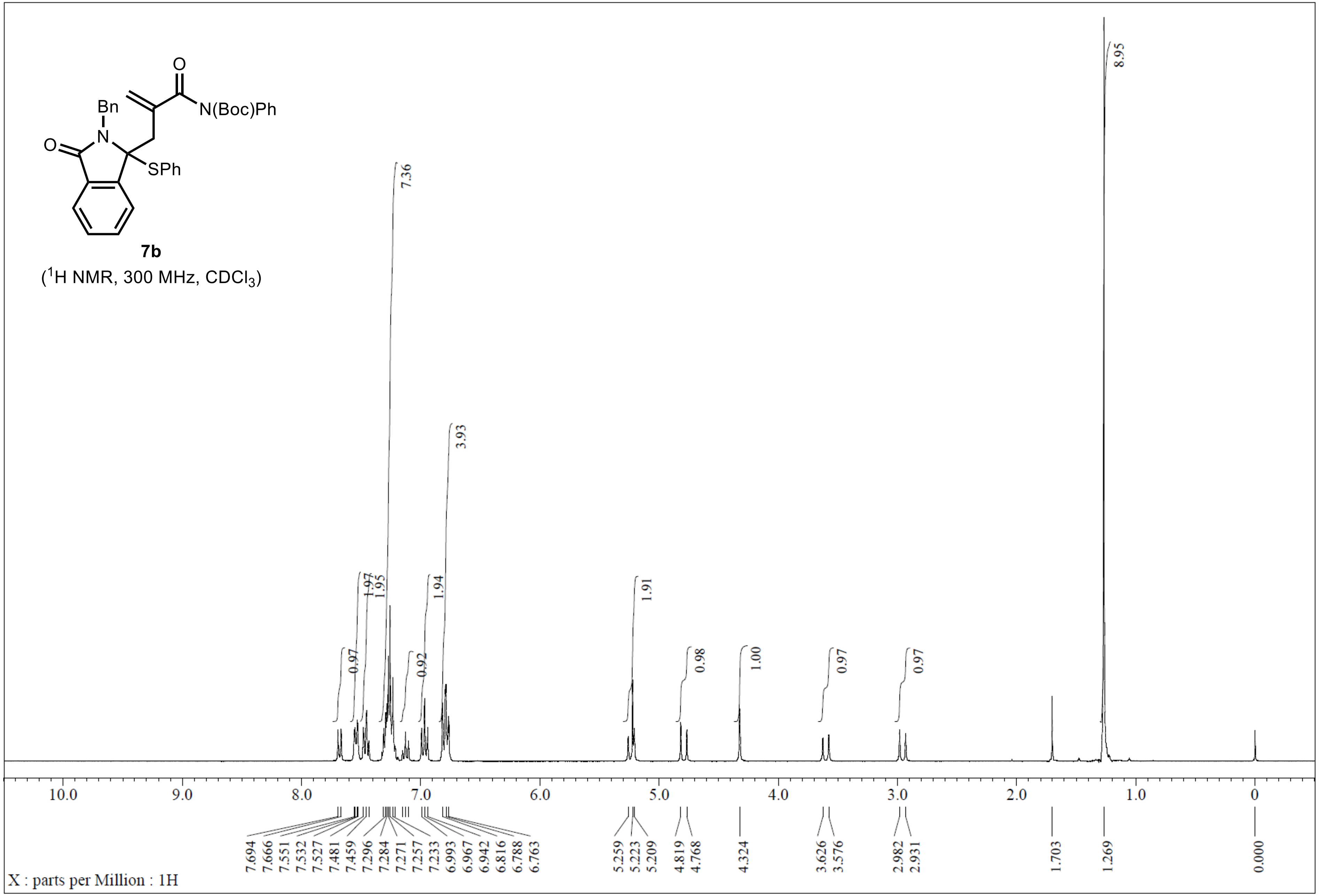
O
Bn
N(Boc)Ph
O
N
SPh
7b
(^{1}H NMR, 300 MHz, $CDCl_3$)
7.36
3.93
1.97
1.95
1.94
1.91
0.97
0.92
0.98
1.00
0.97
0.97
8.95
10.0
9.0
8.0
7.0
6.0
5.0
4.0
3.0
2.0
1.0
0
7.694
7.666
7.551
7.532
7.527
7.481
7.459
7.296
7.284
7.271
7.257
7.233
6.993
6.967
6.942
6.816
6.788
6.763
5.259
5.223
5.209
4.819
4.768
4.324
3.626
3.576
2.982
2.931
1.703
1.269
0.000
X : parts per Million : 1H

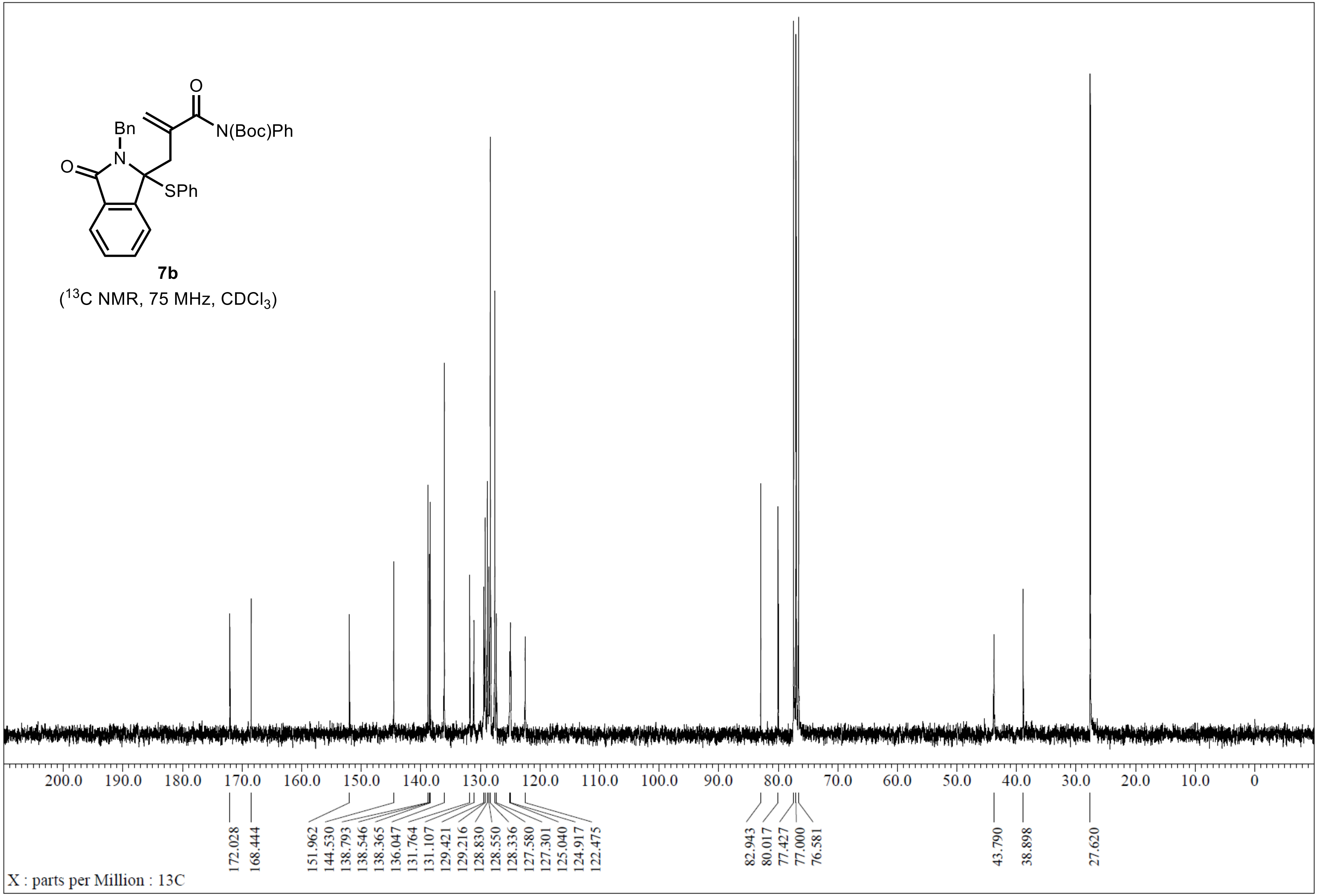
Bn
O
N
SPh
N(Boc)Ph
7b
(13C NMR, 75 MHz, CDCl3)
200.0
190.0
180.0
170.0
160.0
150.0
140.0
130.0
120.0
110.0
100.0
90.0
80.0
70.0
60.0
50.0
40.0
30.0
20.0
10.0
0
172.028
168.444
151.962
144.530
138.793
138.546
138.365
136.047
131.764
131.107
129.421
129.216
128.830
128.550
128.336
127.580
127.301
125.040
124.917
122.475
82.943
80.017
77.427
77.000
76.581
43.790
38.898
27.620
X : parts per Million : 13C

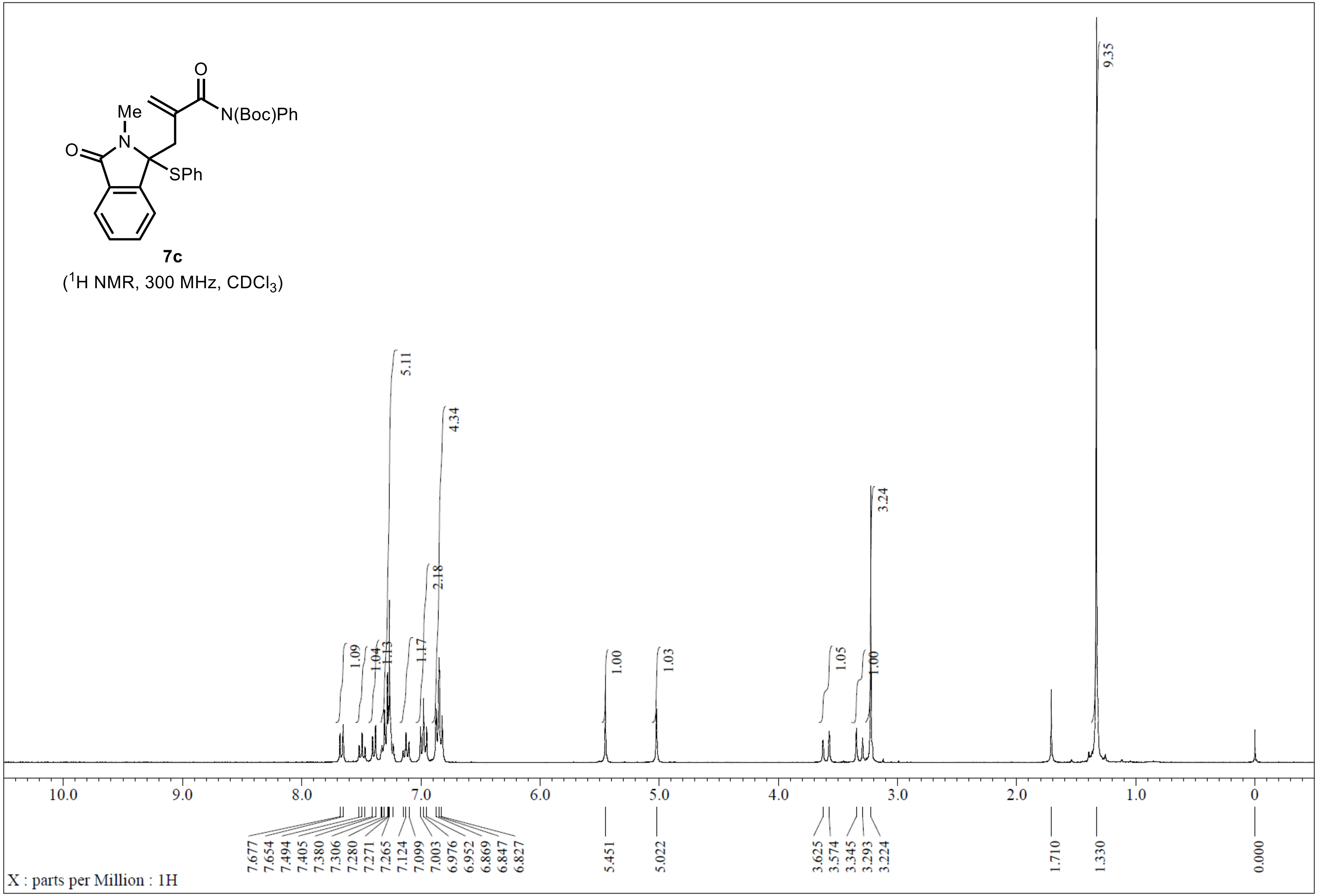
O
Me
N(Boc)Ph
O
N
SPh
7c
(1H NMR, 300 MHz, CDCl3)
X : parts per Million : 1H
10.0
9.0
8.0
7.0
6.0
5.0
4.0
3.0
2.0
1.0
0
1.09
1.04
1.13
5.11
1.17
2.18
4.34
1.00
1.03
1.05
1.00
3.24
9.35
7.677
7.654
7.494
7.405
7.380
7.306
7.280
7.271
7.265
7.124
7.099
7.003
6.976
6.952
6.869
6.847
6.827
5.451
5.022
3.625
3.574
3.345
3.293
3.224
1.710
1.330
0.000

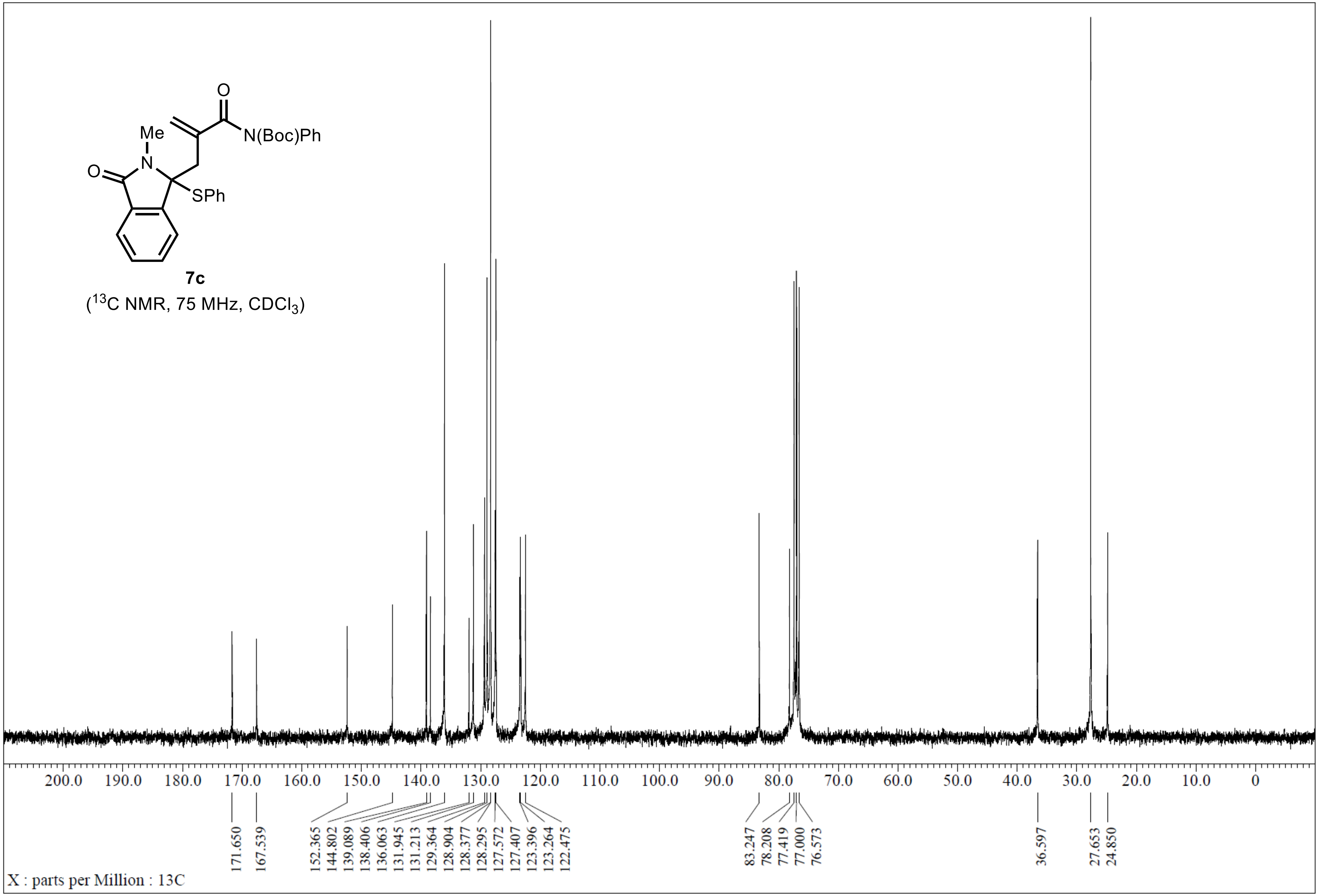
Me
O
N(Boc)Ph
SPh
7c
(13C NMR, 75 MHz, CDCl3)
200.0 190.0 180.0 170.0 160.0 150.0 140.0 130.0 120.0 110.0 100.0 90.0 80.0 70.0 60.0 50.0 40.0 30.0 20.0 10.0 0
171.650
167.539
152.365
144.802
139.089
138.406
136.063
131.945
131.213
129.364
128.904
128.377
128.295
127.572
127.407
123.396
123.264
122.475
83.247
78.208
77.419
77.000
76.573
36.597
27.653
24.850
X : parts per Million : 13C

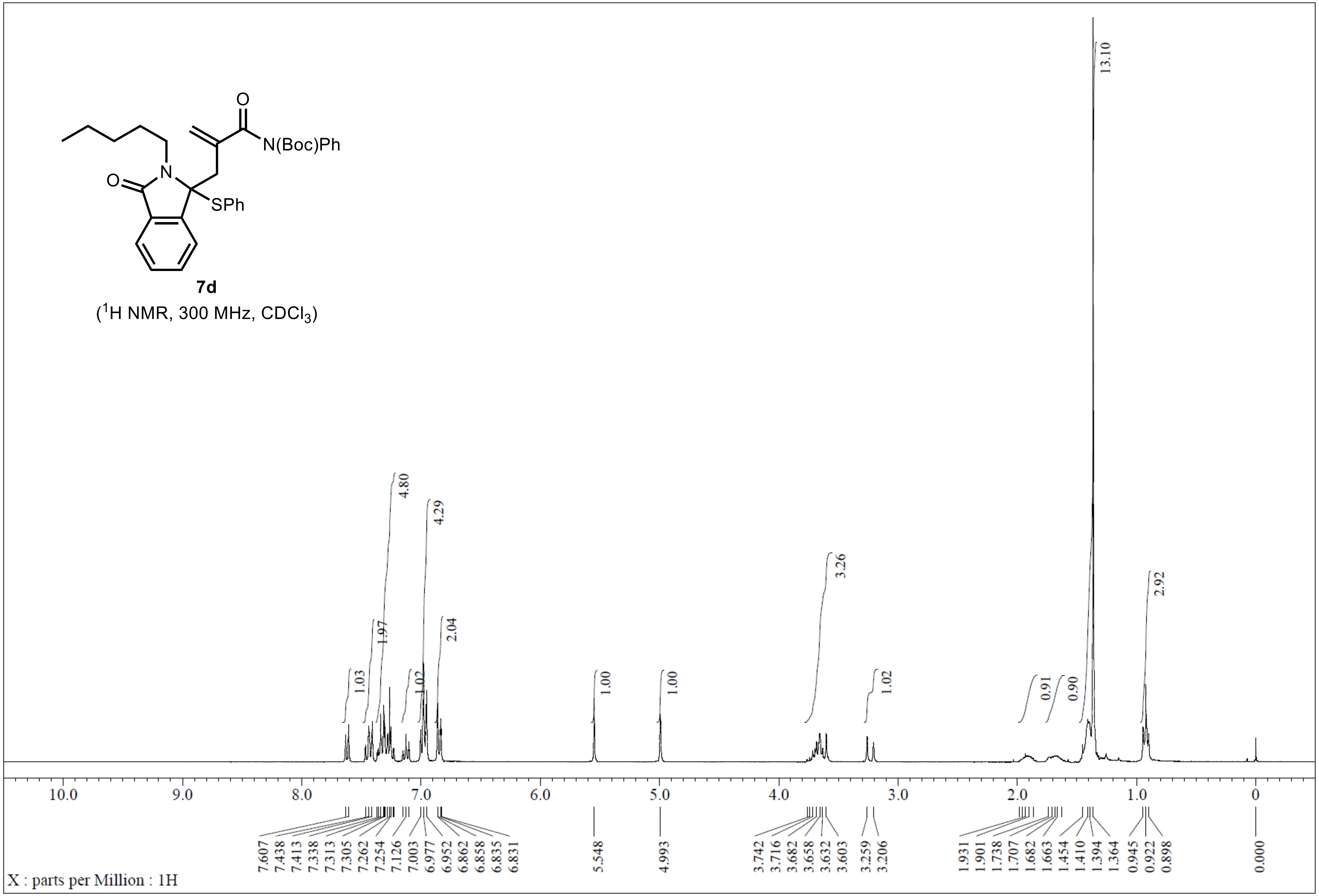

O
N(Boc)Ph
O
N
SPh
7d
(1H NMR, 300 MHz, CDCl3)
X : parts per Million : 1H
10.0
9.0
8.0
7.0
6.0
5.0
4.0
3.0
2.0
1.0
0
1.03
1.97
4.80
1.02
4.29
2.04
1.00
1.00
3.26
1.02
0.91
0.90
13.10
2.92
7.607
7.438
7.413
7.338
7.313
7.305
7.262
7.254
7.126
7.003
6.977
6.952
6.862
6.858
6.835
6.831
5.548
4.993
3.742
3.716
3.682
3.658
3.632
3.603
3.259
3.206
1.931
1.901
1.738
1.707
1.682
1.663
1.454
1.410
1.394
1.364
0.945
0.922
0.898
0.000

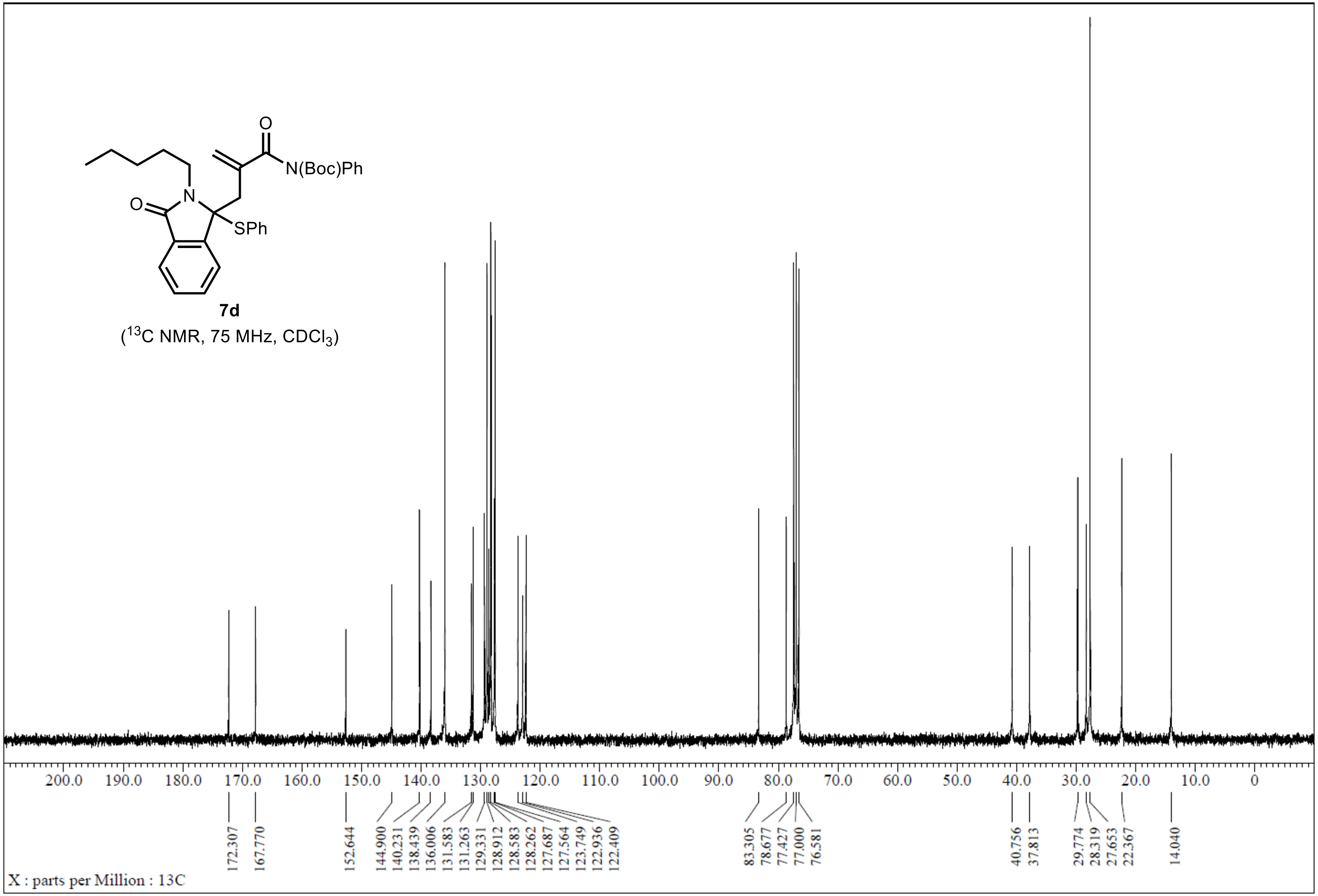
O
N(Boc)Ph
O
N
SPh
7d
(13C NMR, 75 MHz, CDCl3)
200.0 190.0 180.0 170.0 160.0 150.0 140.0 130.0 120.0 110.0 100.0 90.0 80.0 70.0 60.0 50.0 40.0 30.0 20.0 10.0 0
172.307
167.770
152.644
144.900
140.231
138.439
136.006
131.583
131.263
129.331
128.912
128.583
128.262
127.687
127.564
123.749
122.936
122.409
83.305
78.677
77.427
77.000
76.581
40.756
37.813
29.774
28.319
27.653
22.367
14.040
X : parts per Million : 13C

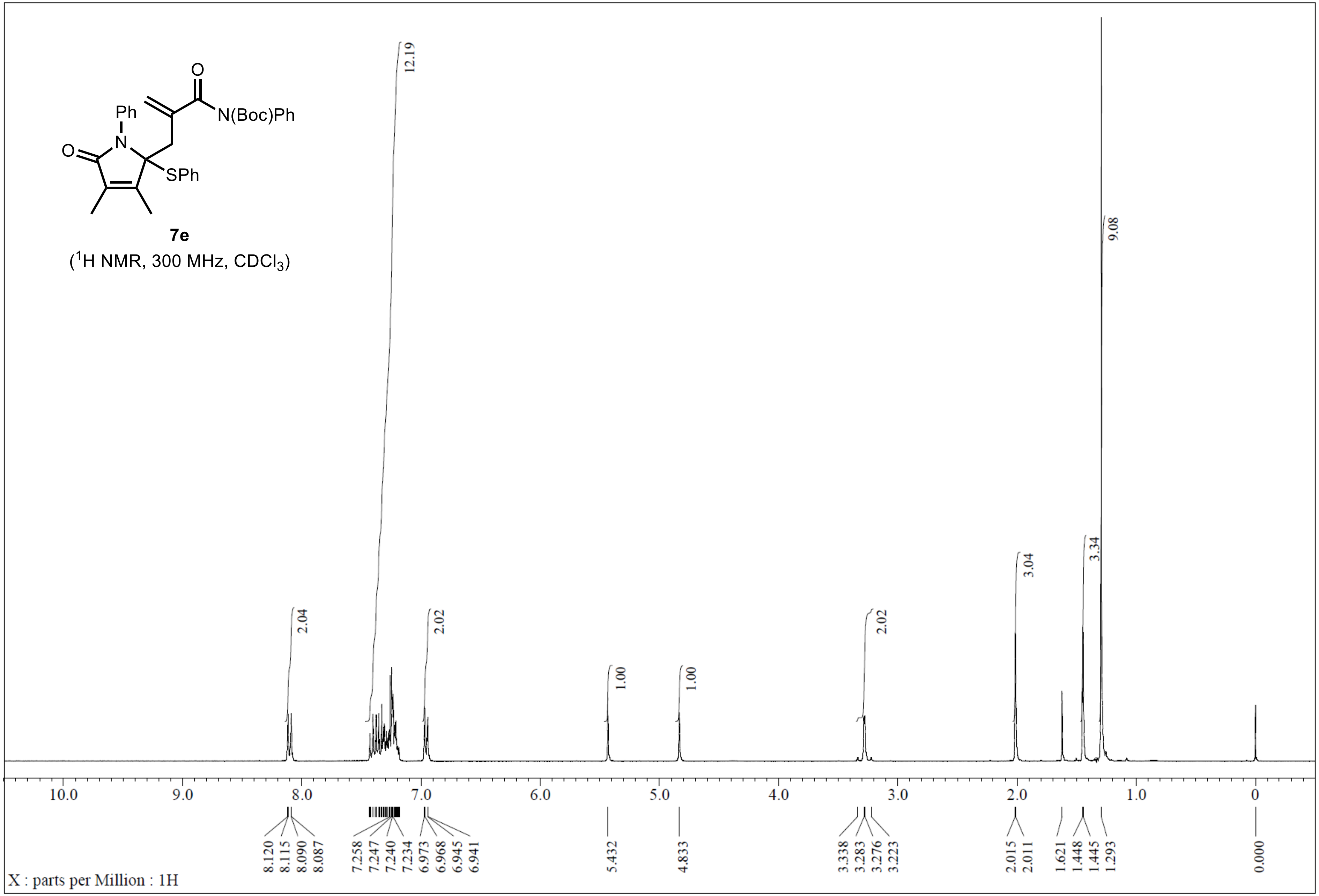
Ph
N
O
O
N(Boc)Ph
SPh
7e
(1H NMR, 300 MHz, CDCl3)
12.19
9.08
3.34
3.04
2.04
2.02
2.02
1.00
1.00
10.0
9.0
8.0
7.0
6.0
5.0
4.0
3.0
2.0
1.0
0
8.120
8.115
8.090
8.087
7.258
7.247
7.240
7.234
6.973
6.968
6.945
6.941
5.432
4.833
3.338
3.283
3.276
3.223
2.015
2.011
1.621
1.448
1.445
1.293
0.000
X : parts per Million : 1H

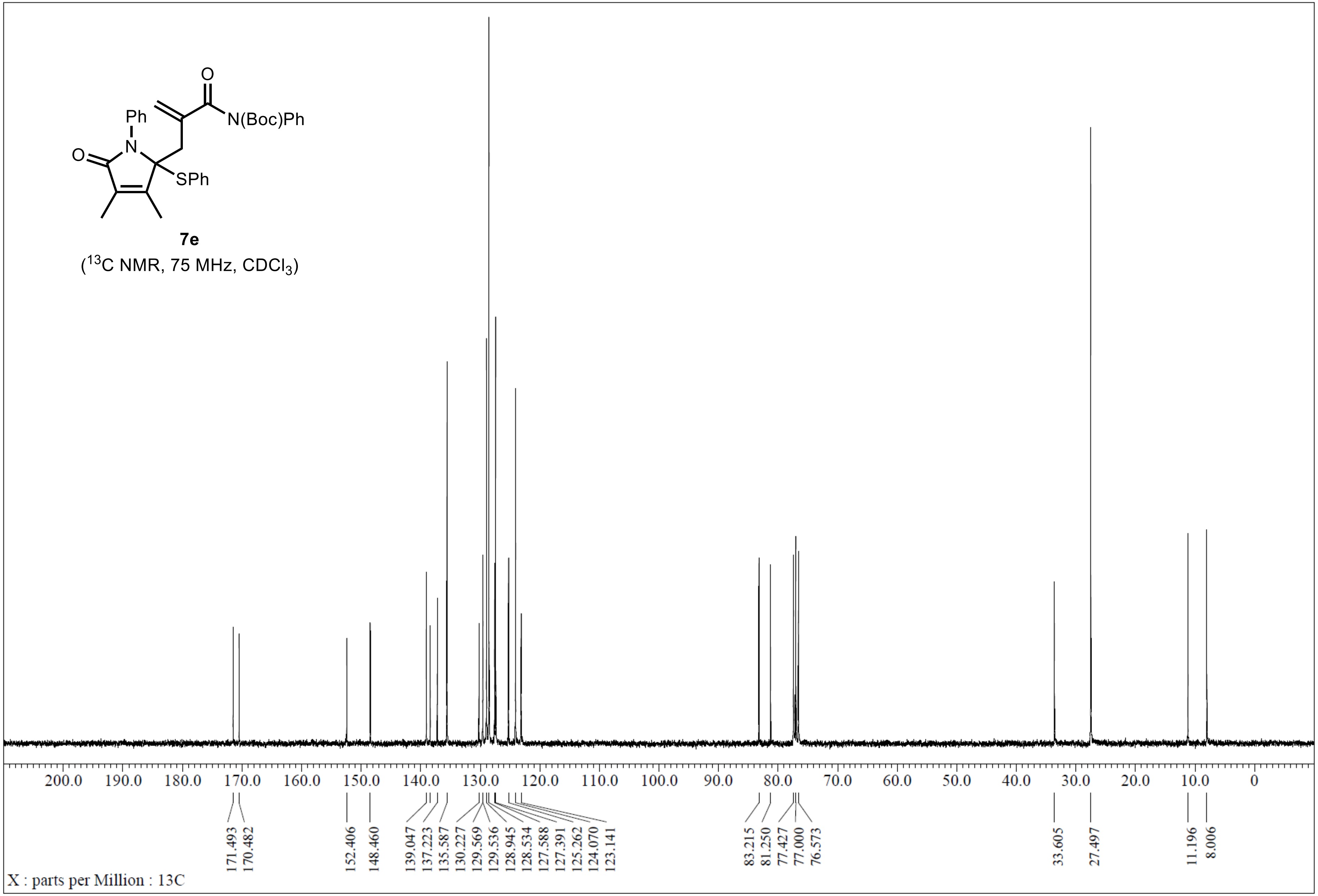
Ph
N
O
O
N(Boc)Ph
SPh
7e
(13C NMR, 75 MHz, CDCl3)
200.0 190.0 180.0 170.0 160.0 150.0 140.0 130.0 120.0 110.0 100.0 90.0 80.0 70.0 60.0 50.0 40.0 30.0 20.0 10.0 0
171.493
170.482
152.406
148.460
139.047
137.223
135.587
130.227
129.569
129.536
128.945
128.534
127.588
127.391
125.262
124.070
123.141
83.215
81.250
77.427
77.000
76.573
33.605
27.497
11.196
8.006
X : parts per Million : 13C

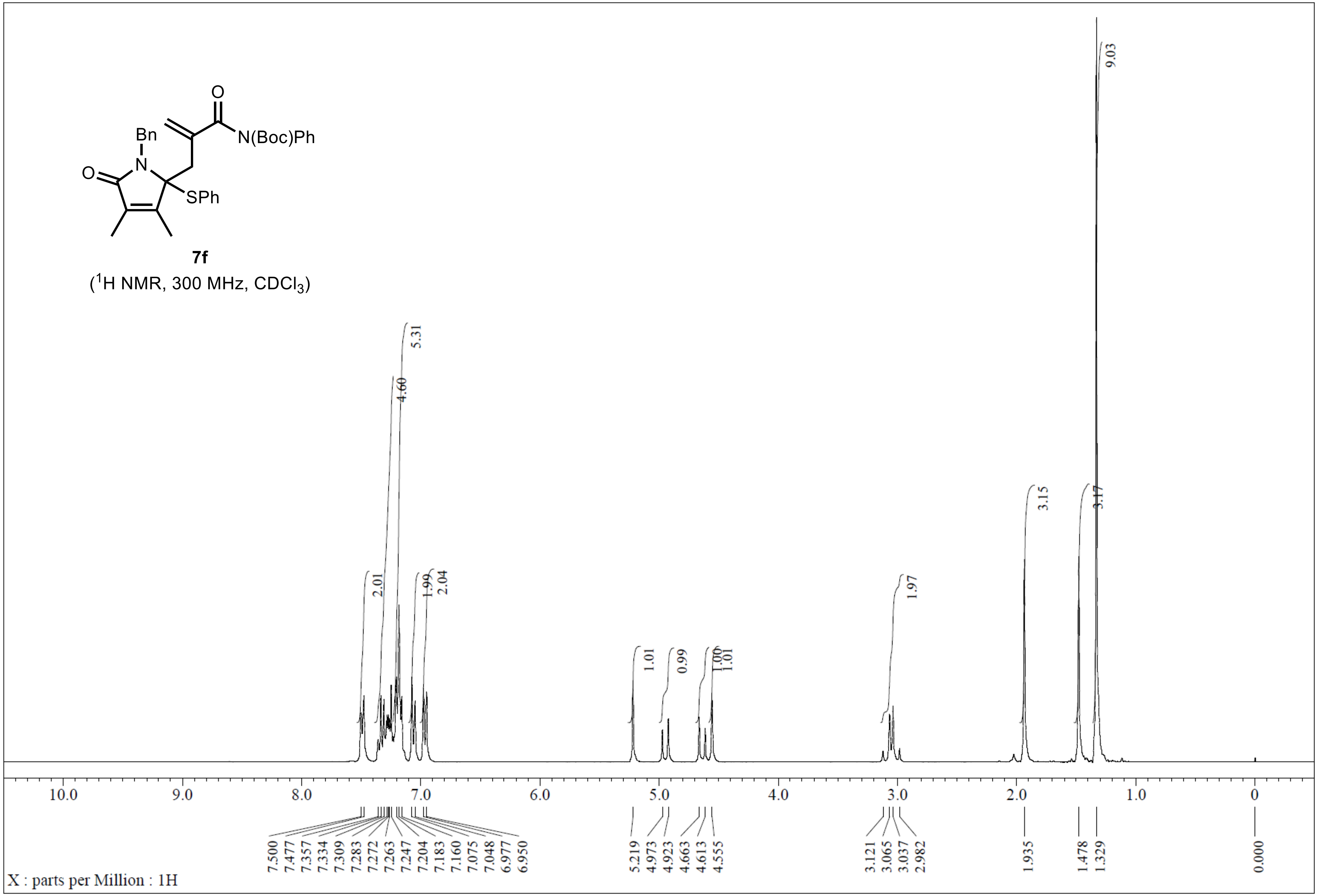
Bn
N(Boc)Ph
SPh
O
7f
(1H NMR, 300 MHz, CDCl3)
X : parts per Million : 1H
10.0
9.0
8.0
7.0
6.0
5.0
4.0
3.0
2.0
1.0
0
2.01
4.60
5.31
1.99
2.04
1.01
0.99
1.00
1.01
1.97
3.15
3.17
9.03
7.500
7.477
7.357
7.334
7.309
7.283
7.272
7.263
7.247
7.204
7.183
7.160
7.075
7.048
6.977
6.950
5.219
4.973
4.923
4.663
4.613
4.555
3.121
3.065
3.037
2.982
1.935
1.478
1.329
0.000

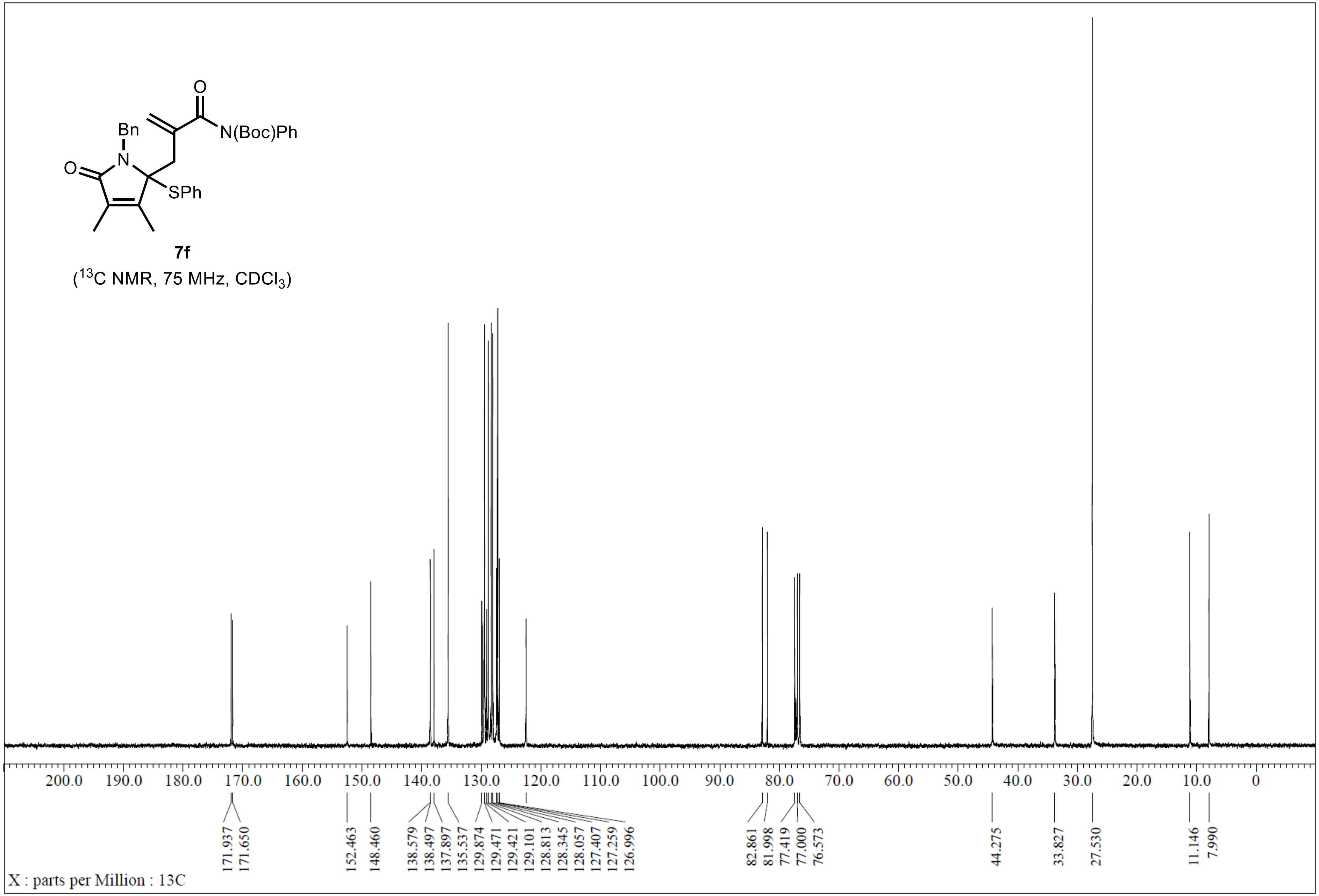
Bn
N
O
SPh
O
N(Boc)Ph
7f
(13C NMR, 75 MHz, CDCl3)
171.937
171.650
152.463
148.460
138.579
138.497
137.897
135.537
129.874
129.471
129.421
129.101
128.813
128.345
128.057
127.407
127.259
126.996
82.861
81.998
77.419
77.000
76.573
44.275
33.827
27.530
11.146
7.990
X : parts per Million : 13C

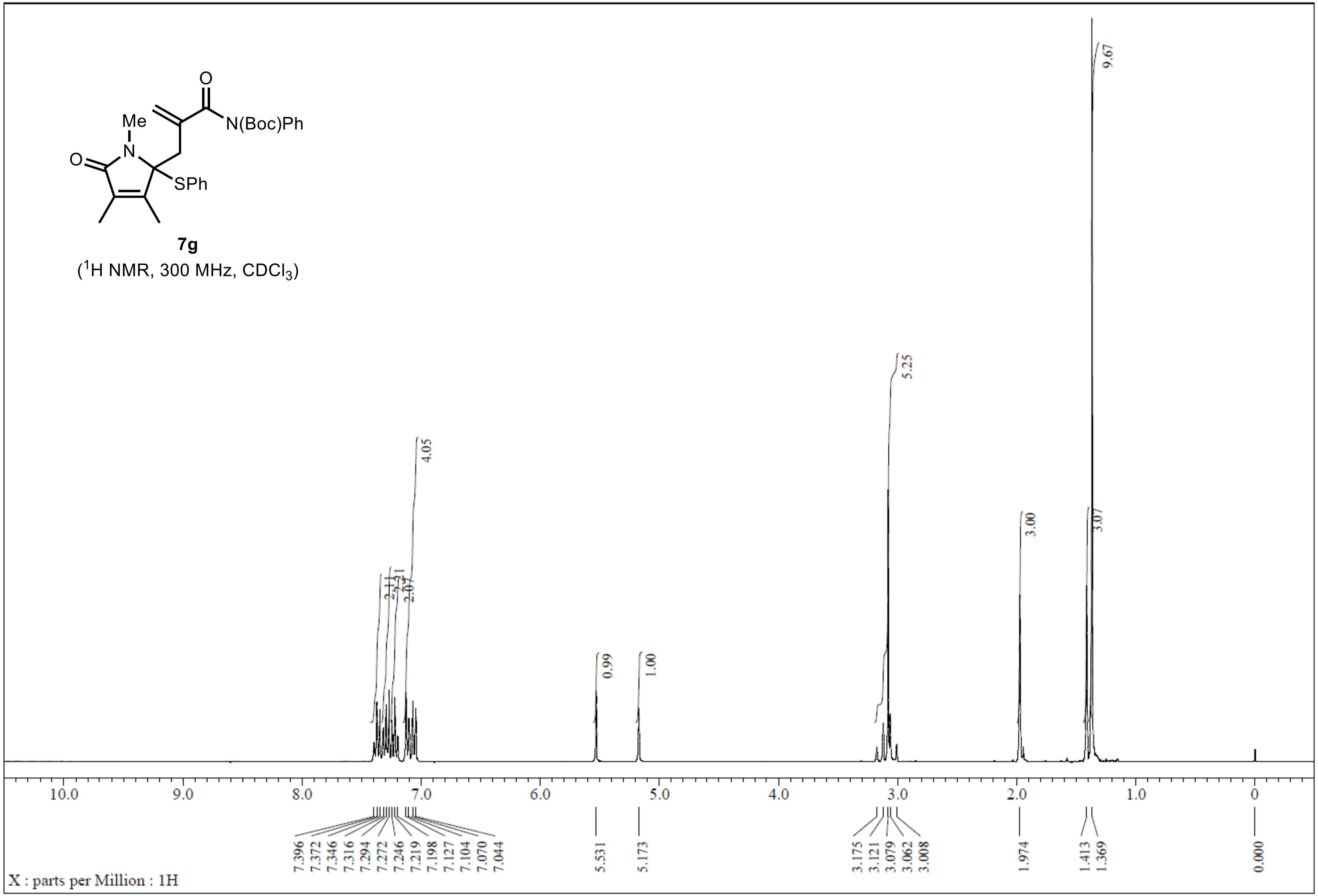

Me
O
N(Boc)Ph
SPh
7g
(1H NMR, 300 MHz, CDCl3)
9.67
5.25
4.05
3.00
3.07
2.11
2.21
2.07
0.99
1.00
10.0
9.0
8.0
7.0
6.0
5.0
4.0
3.0
2.0
1.0
0
7.396
7.372
7.346
7.316
7.294
7.272
7.246
7.219
7.198
7.127
7.104
7.070
7.044
5.531
5.173
3.175
3.121
3.079
3.062
3.008
1.974
1.413
1.369
0.000
X : parts per Million : 1H

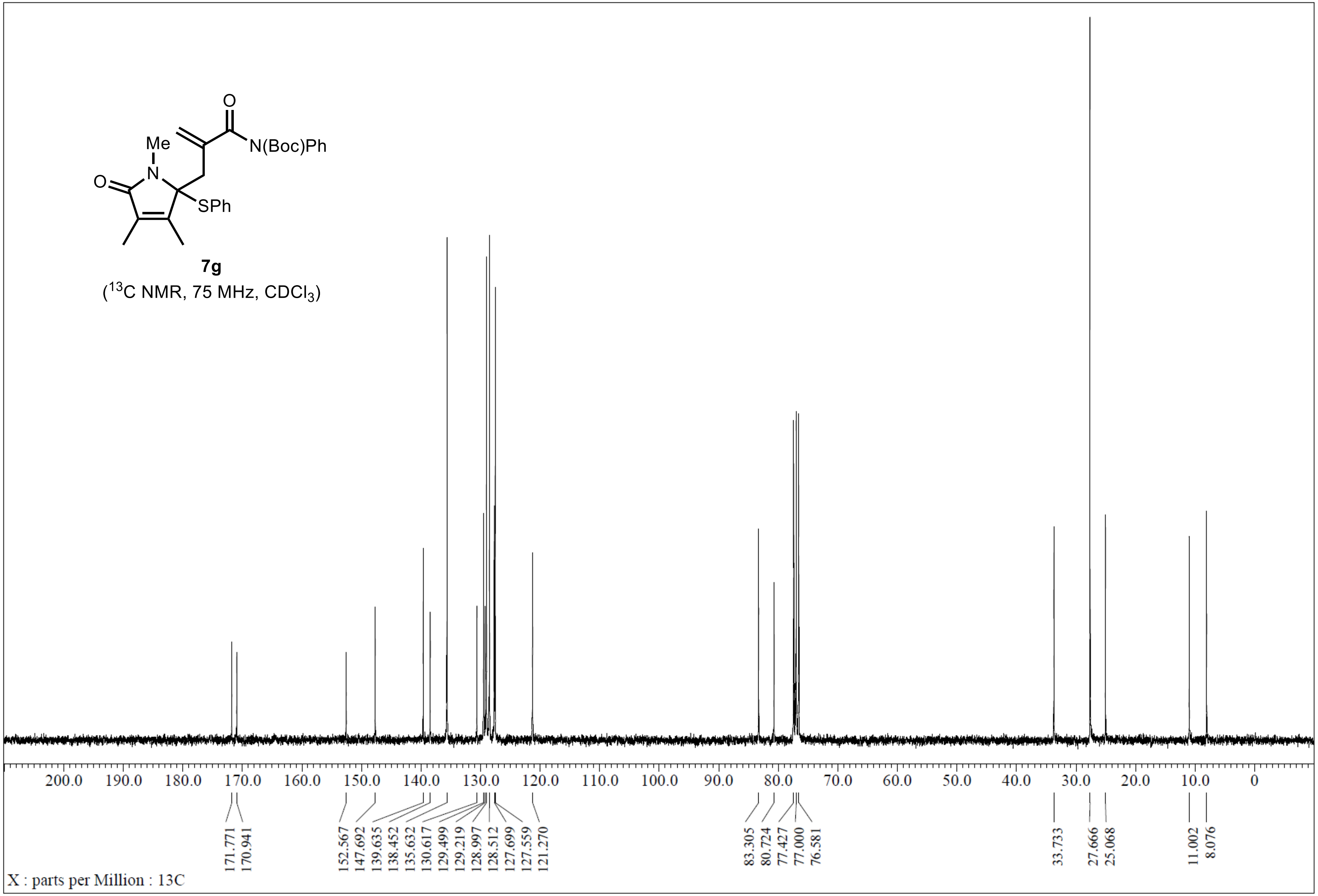

Me
O
N
N(Boc)Ph
O
SPh
7g
(13C NMR, 75 MHz, CDCl3)
171.771
170.941
152.567
147.692
139.635
138.452
135.632
130.617
129.499
129.219
128.997
128.512
127.699
127.559
121.270
83.305
80.724
77.427
77.000
76.581
33.733
27.666
25.068
11.002
8.076
200.0 190.0 180.0 170.0 160.0 150.0 140.0 130.0 120.0 110.0 100.0 90.0 80.0 70.0 60.0 50.0 40.0 30.0 20.0 10.0 0
X : parts per Million : 13C

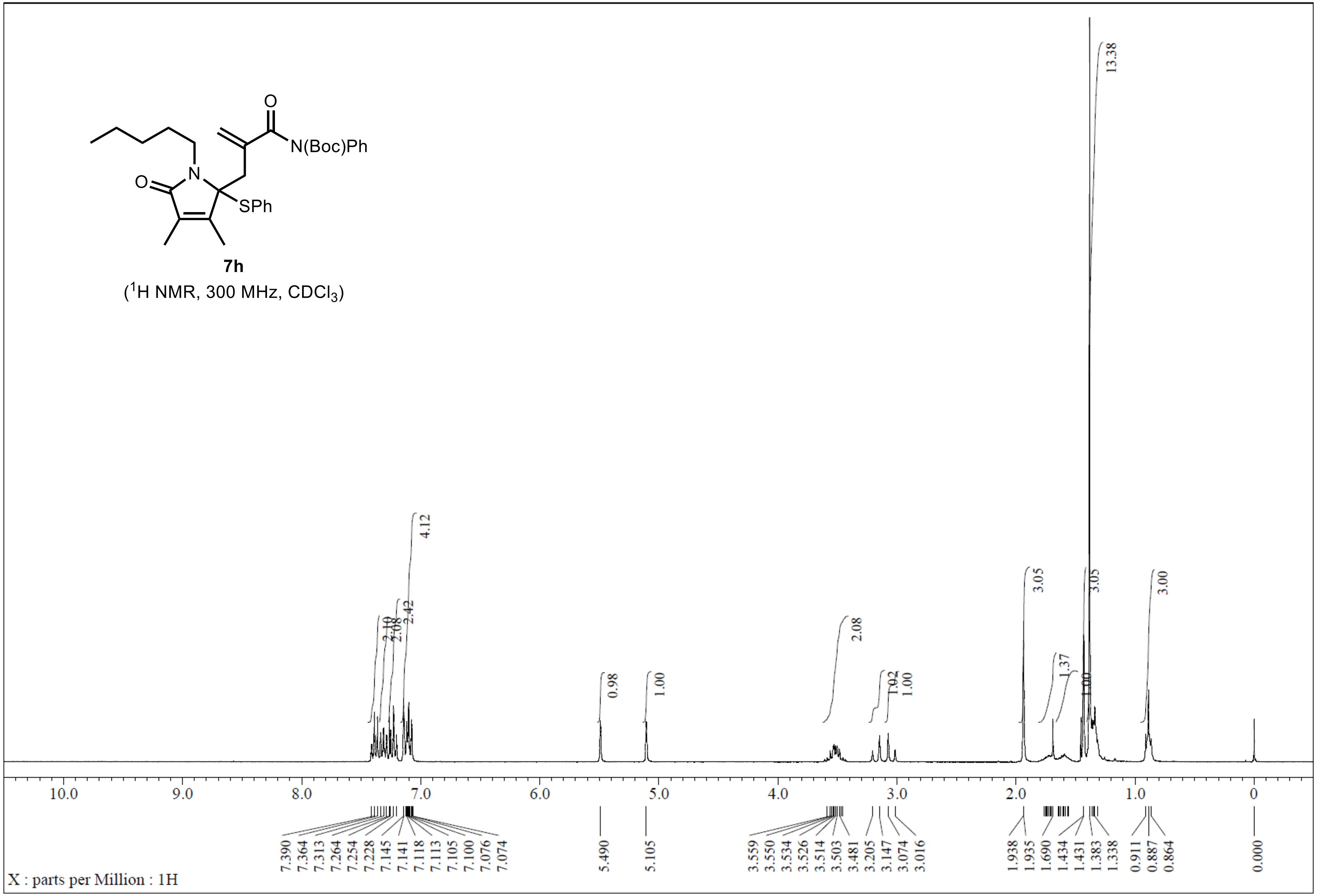
O
N(Boc)Ph
O
N
SPh
7h
(1H NMR, 300 MHz, CDCl3)
13.38
4.12
2.10
2.08
2.42
0.98
1.00
2.08
1.92
1.00
3.05
1.37
1.00
3.05
3.00
10.0
9.0
8.0
7.0
6.0
5.0
4.0
3.0
2.0
1.0
0
7.390
7.364
7.313
7.264
7.254
7.228
7.145
7.141
7.118
7.113
7.105
7.100
7.076
7.074
5.490
5.105
3.559
3.550
3.534
3.526
3.514
3.503
3.481
3.205
3.147
3.074
3.016
1.938
1.935
1.690
1.434
1.431
1.383
1.338
0.911
0.887
0.864
0.000
X : parts per Million : 1H

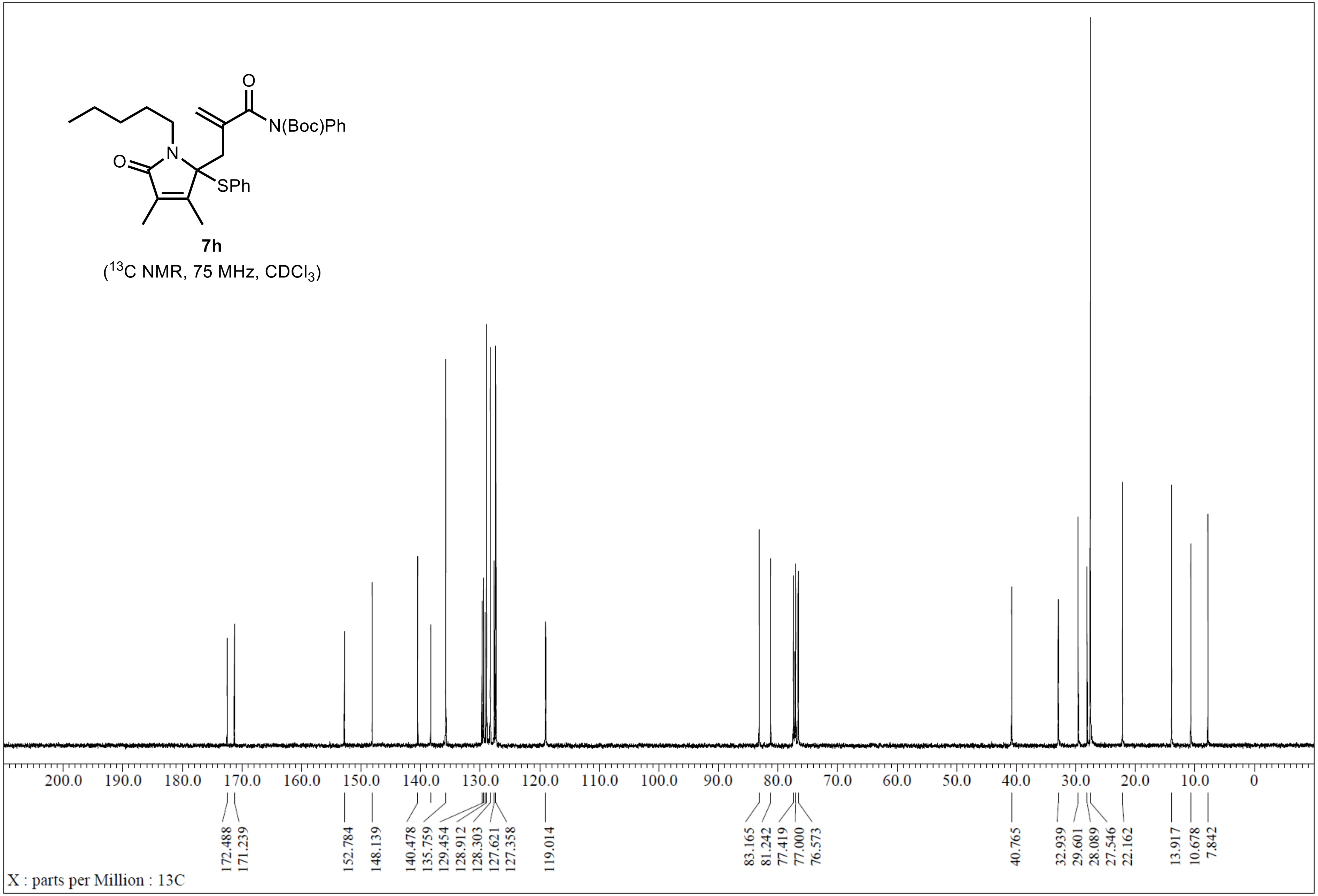

O
N(Boc)Ph
O
N
SPh
7h
(^{13}C NMR, 75 MHz, CDCl$_3$)
200.0 190.0 180.0 170.0 160.0 150.0 140.0 130.0 120.0 110.0 100.0 90.0 80.0 70.0 60.0 50.0 40.0 30.0 20.0 10.0 0
172.488
171.239
152.784
148.139
140.478
135.759
129.454
128.912
128.303
127.621
127.358
119.014
83.165
81.242
77.419
77.000
76.573
40.765
32.939
29.601
28.089
27.546
22.162
13.917
10.678
7.842
X : parts per Million : 13C
